# Tunable Anion Recognition at the Lower Rim of Resorcin[4]arenes: Strength, Selectivity, and Transport

**DOI:** 10.1021/jacsau.5c01041

**Published:** 2025-09-29

**Authors:** Deepshikha Priyadarshini, Ronedy Naorem, Marek P. Szymański, Oksana Danylyuk, Michał J. Chmielewski, Agnieszka Szumna

**Affiliations:** † Institute of Organic Chemistry, 49559 Polish Academy of Sciences, Kasprzaka 44/52, Warsaw 01-224, Poland; ‡ University of Warsaw, Faculty of Chemistry, Biological and Chemical Research Centre, Żwirki i Wigury 101, Warsaw 02-089, Poland; § Institute of Physical Chemistry, 119463 Polish Academy of Sciences, Kasprzaka 44/52, Warsaw 01-224, Poland

**Keywords:** anion receptors, calixarenes, anion transporters, CH-bonding receptors, substituent effects

## Abstract

Selective anion binding and transport are crucial in many chemical and biological settings. CH-bonding receptors–which rely on nonclassical CH···anion hydrogen bonds, offer a pH-independent alternative to conventional hosts; however, their design is challenged by the inherently weak nature of CH···anion interactions. In this study, we present modified resorcin[4]­arenes as versatile scaffolds to address this challenge. By introducing electron-withdrawing groups (EWGs) at the upper rim, we convert π–electron-rich resorcin­[4]­arenes into potent anion receptors. A series of resorcin[4]­arenes bearing −Br, −CHO, −NO_2_, and −CN substituents exhibit a systematic enhancement in anion binding affinity, reaching the highest value in the series for the CN-substituted receptor: *K*_a_(Cl^–^, THF) = 7 × 10^5^ M^–1^. The log*K*_a_ values correlate with the electrostatic potential (ESP) at the binding site, calculated by DFT methods. In addition, the incorporation of hydroxyl-terminated alkyl chains at the lower rim promotes the formation of higher-order complexes and further boosts anion binding, even in competitive aqueous–organic media. These hydroxyalkyl-footed receptors display exceptional selectivity for HSO_4_^–^, with a selectivity factor of 17 over similar tetrahedral oxyanions. Transmembrane anion transport studies in large unilamellar vesicles reveal that the nitro-substituted resorcin[4]­arene is by far the most effective chloride transporter in this series, followed by the CN-substituted analogue, emphasizing that the most strongly binding receptors are not necessarily the most efficient transporters. Detailed analysis of molecular lipophilicity potential (MLP) maps shows that subtle differences in upper- and lower-rim polarity, as well as excessive hydrophilicity at the lower rim, can diminish transport efficiency by hindering membrane reorientation or promoting interfacial anchoring. These mechanistic and structure–activity insights provide clear design principles for developing next-generation CH-bonding transporters with improved performance. Collectively, these results highlight the potential of resorcin[4]­arenes as tunable platforms for tailoring anion binding strength, selectivity, and anionophoric properties through simple peripheral modifications.

## Graphical abstract

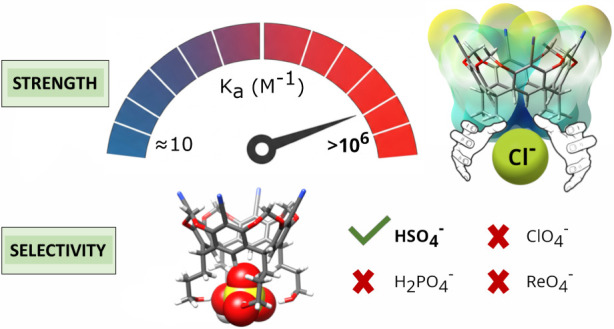

## Introduction

Anions are ubiquitous in chemical and biological systems, where they play essential roles in enzymatic catalysis, signal transduction, environmental processes, and materials science. Consequently, the selective binding, sensing, and transport of anions are of paramount importance.^1–4^ While conventional anion receptors rely on hydrogen bonding or electrostatic interactions with positively charged moieties, charge-neutral receptors based on CH hydrogen bond donors have recently emerged as a promising alternative.^5–17^ These CH-bonding receptors offer notable advantages, stemming primarily from their chemical robustness, pH independence, and lipophilicity. This characteristic enables them to function in a wide range of environments, regardless of anion basicity, making them attractive candidates for sensors, anion-responsive materials, and membrane transport systems, particularly in contexts where avoiding cytotoxic proton cotransport is crucial.^8,18–20^

Despite their potential, designing effective CH-bonding receptors remains challenging. The relatively weak nature of CH···anion interactions compared to classical hydrogen bonds or electrostatic forces necessitates careful optimization of molecular designs. The key strategies include: (i) increasing the partial positive charge on CH donors through the introduction of electron-withdrawing groups (EWGs); (ii) maximizing the number of CH···anion contacts; (iii) enforcing receptor preorganization to reduce entropic penalties; and (iv) leveraging cooperative binding effects. Macrocyclic scaffolds are particularly well-suited for integrating these design elements.^6,9,13,21^ Although macrocycles bearing electron-donating substituents, such as calixarenes, resorcinarenes, pillararenes, and various hybrid structures, are well represented and structurally diverse, macrocyclic scaffolds incorporating EWGs remain scarce. Notable exceptions, such as cyanostars,^12^ triazole-based macrocycles,^5^ bambusurils,^21,22^ and biotinurils^23^ have demonstrated the high potential of CH-bonding receptors; however, their development is often hampered by synthetic difficulties and limited tunability of their binding sites.

We have recently demonstrated that resorcin[4]­arenes - macrocycles traditionally regarded as electron-rich and widely used for cation binding,^24,25^ can be re-engineered into effective CH-bonding anion receptors by constraining their conformation and introducing EWGs at the upper rim.^26,27^ For instance, receptor **1**, featuring a preorganized cone conformation and a convergent array of CH groups at the lower rim (Figure 1a), exhibits strong affinity for chloride in THF (*K*_a_ = 1.36 × 10^5^ M^–1^) and promotes efficient and selective Cl^–^ transport across lipid bilayers, demonstrating both high binding strength and functional utility.

**Figure 1:**
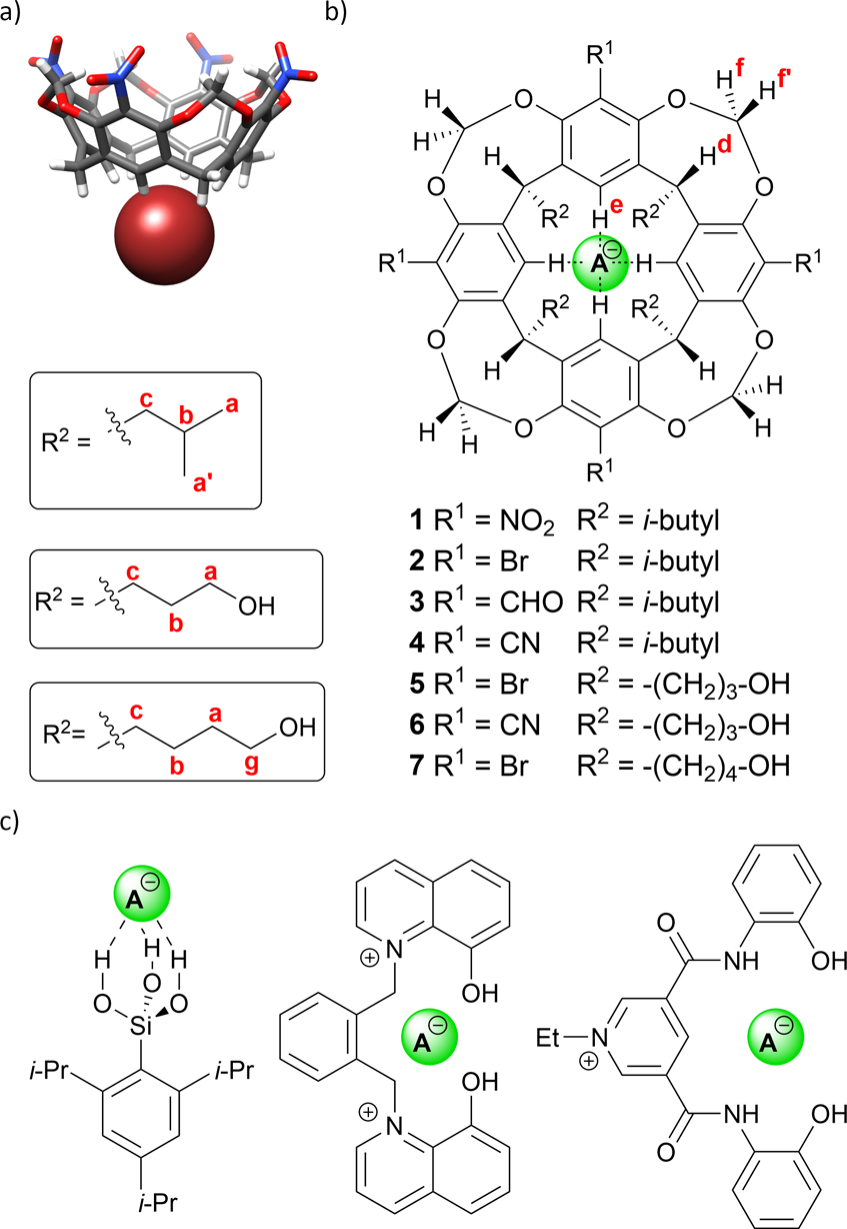
(a) Anion binding mode by nitroresorcin[4]­arene **1**;^26^ (b) structures of resorcin[4]­arenes studied in this work, along with the notation used for NMR signals; (c) selected examples of anion receptors featuring hydroxyl (OH) groups in their binding motifs.^29,31,33^

In this study, we address the scarcity of EWG-substituted macrocycles by developing a family of tunable CH-bonding receptors based on the easily synthesized resorcin[4]­arene scaffold (Figure 1b). We systematically examine how upper-rim substitution with various EWGs (−NO_2_, −Br, −CHO, and −CN in receptors **1**–**4**) influences their anion binding strength. In parallel, we investigate the modification of the lower rim (“feet”) with hydroxyalkyl groups (receptors **5**–**7**) to assess potential cooperativity between CH and OH hydrogen bond donors. Although hydroxyl groups are known to interact effectively with anions, either alone^28,29^ or cooperatively with other classical hydrogen bond donors (Figure 1c)^30–33^ - their interplay with hydrophobic CH donors has not been systematically investigated, especially in mixed aqueous–organic environments. Both upper- and lower-rim modifications were expected to modulate anion binding in a highly environment-dependent manner. While the impact of substituents on binding affinity is well established,^34^ these effects are also known to vary significantly with solvent composition.^35^ Accordingly, the binding properties of receptors **1**–**7** were evaluated in both aprotic (THF) and partially aqueous (THF:H_2_O) systems, revealing unique selectivity trends. Finally, the anion transport properties of the new receptors were investigated and compared with the previously published results for receptors **1** and **2**.

## Results

### Design, Synthesis, and Structure

The key structural features that enable effective anion binding by tetranitroresorcin[4]­arene **1** include the presence of EWGs at the central upper-rim positions R^1^ and conformational locking of the macrocycle in a cone geometry via methylene bridges between the resorcinol hydroxyl groups. This rigidified architecture fixes the out-of-plane orientation of −OCH_2_O– groups, restricting electron donation through resonance while preserving the electron-withdrawing inductive effect.^26^ These design principles were retained in all studied receptors **1**–**7**.

The syntheses of compounds **2**,^36^ **3**,^37^ **5**,^38^ and **7** ^38^ were accomplished via established literature methods, with slight modifications. For receptors **2**, **5**, and **7**, the general approach involved a one-pot acid-catalyzed condensation of resorcinol with the appropriate aldehyde to afford the macrocyclic resorcin[4]­arene scaffold, followed by bromination of the aromatic rings with NBS and finally, the bridging of the resorcinol hydroxyl groups using bromochloromethane. Receptor **3** was obtained via a different synthetic route, in which hydroxyl bridging was performed first, followed by formylation of aromatic rings with *n*-BuLi and DMF. Compounds **4** and **6** were obtained by postfunctionalization of **2** and **5**, respectively, through a copper-catalyzed cyanation using CuCN and FeCl_3_, under conditions adapted from literature protocols for analogous systems.^39^ Full experimental details are provided in the SI.

Attempts to synthesize resorcin[4]­arenes bearing hydroxyalkyl substituents at the lower rim and nitro groups at the upper rim were unsuccessful. The condensation of 2-nitroresorcinol with hydroxy aldehydes led exclusively to linear oligomeric products, while postmacrocyclization nitration resulted in degradation of the macrocyclic framework.

The ^1^H NMR spectra of compounds **2**–**7** in THF-*d*_8_ are consistent with cone conformations exhibiting *C*_4v_ symmetry for all receptors. To investigate the presence of intra- or intermolecular hydrogen bonding in the OH-appended receptors **5**–**7**, we carried out variable temperature (VT) NMR and Diffusion Ordered Spectroscopy (DOSY) experiments. At room temperature in THF-*d*_8_, the OH signals for **5** and **6** (receptor **7** was insoluble) were not visible; however, upon cooling to 255 K, well-resolved OH signals emerged at δ­(**5**, OH) = 4.65 ppm and δ­(**6**, OH) = 4.80 ppm. For comparison, under identical concentration and temperature conditions, the OH signal of *n*-butanol appeared at δ­(*n-*BuOH, OH) = 3.61 ppm. The significantly downfield-shifted OH signals of **5** and **6** suggest the involvement of their OH groups in hydrogen bonding interactions. DOSY measurements in THF-*d*_8_ yielded diffusion coefficients (*D*) of 6.72 × 10^–10^ m^2^ s^–1^ for **2**, corresponding to a hydrodynamic radius *r*_H_ of 6.7 Å, and 6.23 × 10^–10^ m^2^ s^–1^ for **6**, corresponding to *r*_H_ = 7.3 Å. These values, determined under identical experimental conditions, indicate that **6** does not significantly dimerize in THF-*d*_8_. Together, these data suggest that any hydrogen bonding in **5** and **6** is either intramolecular or involves interactions with THF or water molecules.

Determination of the crystal structure of **6** by X-ray crystallography (crystals grown from THF:water, Figure 2) revealed that the molecule adopts a distorted *C*_2v_-symmetric conformation. The structure features a rectangular upper-rim cavity occupied by a disordered THF molecule and a lower-rim cavity that hosts water molecules. Direct intramolecular hydrogen bonds between the lower-rim OH groups (“feet”) are absent; instead, these interactions are mediated by water molecules. In contrast to their behavior in THF, molecules of **6** in the crystal lattice form “feet-to-feet” dimers via intermolecular hydrogen bonds between their OH groups, likely driven by crystal packing effects.

**Figure 2:**
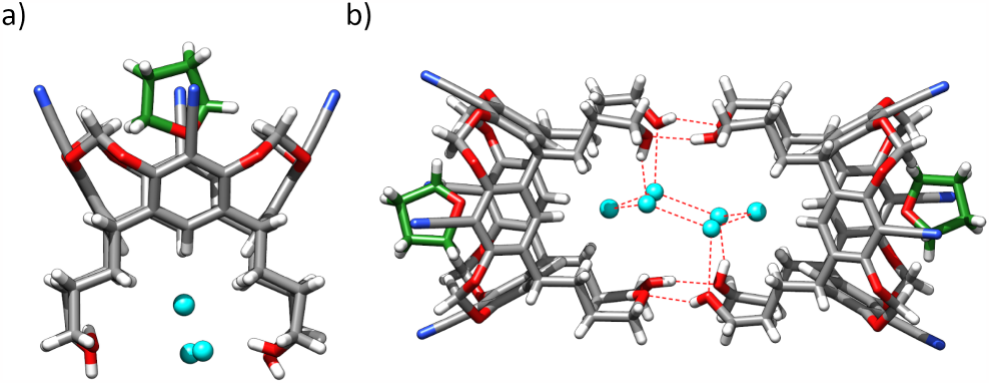
X-ray structure of **6**: (a) molecular structure with the closest surroundings (THF in green, water in cyan); (b) feet-to-feet dimers in the crystal lattice.

Although crystal packing can influence the conformation of flexible molecules, the interaction patterns observed in the solid state might still provide valuable structural insights. Notably, water molecules were found within the hydrophobic lower-rim cavity - a region typically unfavorable for polar guests. The oxygen atom of one such water molecule is engaged in eight CH···O interactions, with CH···O distances ranging from 2.79 to 2.87 Å. This suggests that the cavity is particularly well-suited for accommodating hydrogen bond acceptors, such as anions. Furthermore, the absence of intramolecular OH···OH hydrogen bonds in the crystal structure suggests that steric hindrances may prevent such interactions.

### Theoretical Calculations

For the initial assessment of the binding potential of the new receptors, we employed DFT calculations. Following the approach used by various authors,^6,18^ including our group,^26,27^ electrostatic surface potential (ESP) mapped onto electron density isosurfaces was used as a measure of the relative binding abilities of neutral receptors toward charged species. ESP values implicitly incorporate various substituent effects, such as Hammett parameters,^40^ dipole moments, and conformational factors. To speed up calculations, simplified models of compounds **1**–**4** were constructed by replacing the *i*-butyl chains at the lower rim (R^2^) with methyl groups (denoted **1a**–**4a**). As previously demonstrated, this modification has a negligible impact on both the conformation of the macrocyclic framework and its ESP values.^27^

DFT calculations (B3LYP/6–31+G­(d,p), with the solvation model based on density (SMD) for THF) revealed the following trend in ESP maxima: **2a** < **3a** < **1a** < **4a**, suggesting that the newly designed CN-substituted receptor **4** may exhibit superior anion-binding ability compared to the previously reported NO_2_-substituted receptor **1** (Figure 3a–d). Notably, the ESP values do not strictly correlate with either the Hammett σ_para_ constants^40^ or the inductive sigma σ_I_ value^41^ (Figure 3e,f). This discrepancy may be due to several factors: (1) Hammett parameters pertain to reactivity at carbon atoms and are not directly transferrable to ESP values at hydrogen atoms; (2) steric factors - for example, the NO_2_ group is bulkier than Br or CN and, due to repulsions with two *ortho*-positioned oxygen atoms, it is nearly orthogonal to the phenyl ring (angle 70°); and (3) the contributions of neighboring atomic partial charges can also affect the ESP values. Consequently, receptor **1**, substituted with NO_2_ groups (highest σ_para_) has a lower ESP value than the newly designed receptor **4**, which is substituted with CN groups (lower σ_para_).

**Figure 3:**
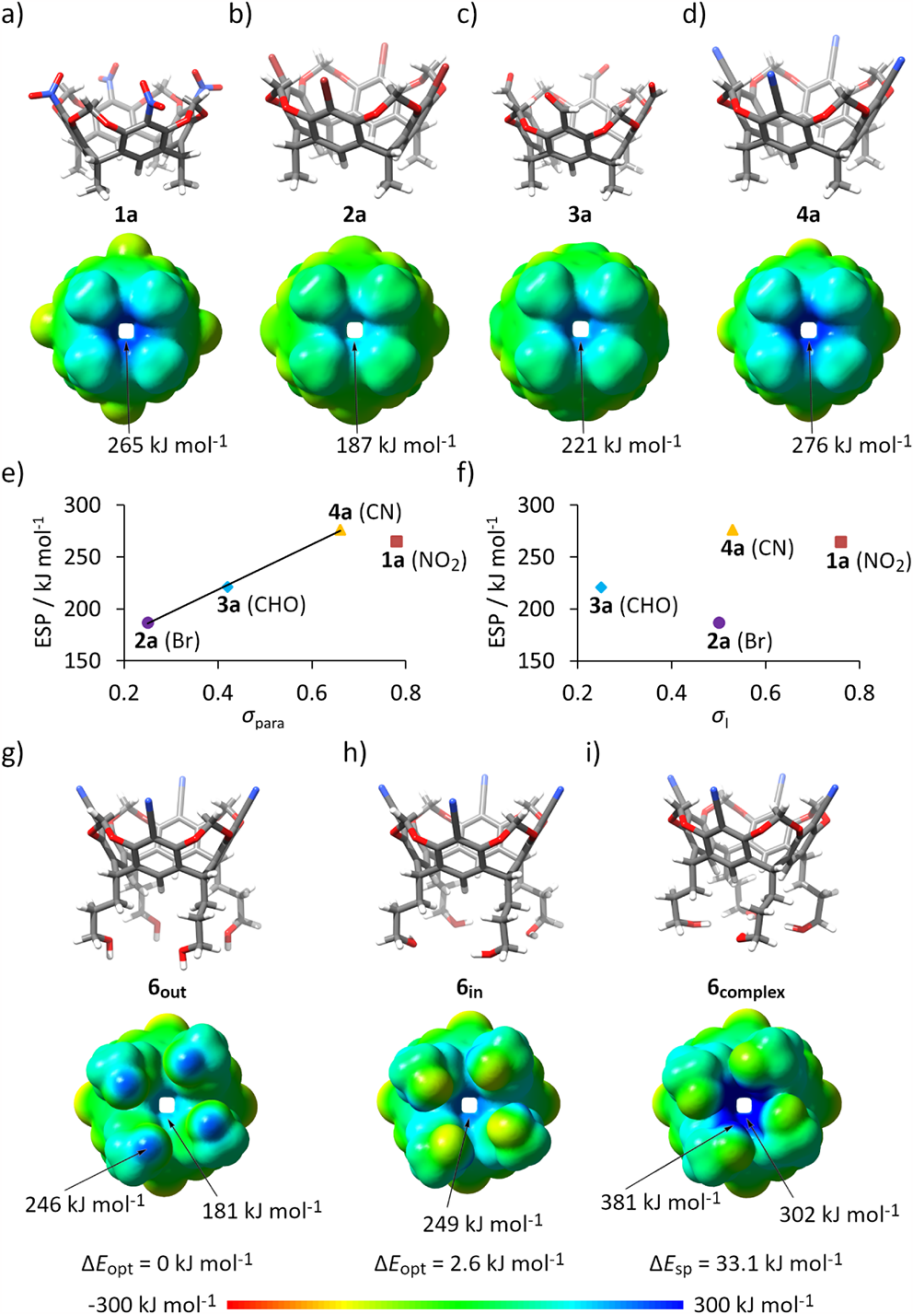
Theoretical calculations of molecular properties of receptors. Geometry optimized structures with ESP values mapped onto electron density isosurfaces for: (a) **1a**; (b) **2a**; (c) **3a**; (d) **4a**; (g–i) various conformations of **6**. Correlations between ESP values of **1a**-**4a** and Hammett substituent parameters: (e) σ_para_; and (f) inductive sigma σ_I_. All calculations were performed using DFT B3LYP/6–31+G­(d,p) with the SMD solvent model for THF, isosurface at 0.001 e au^–3^.

Calculations of ESP values for receptor **6**, which bears −(CH_2_)_3_–OH groups at the lower rim, were complicated by the conformational flexibility of the pendant alkyl chains and the possible formation of intramolecular hydrogen bonds between neighboring OH groups. To account for this, six initial geometries (including structures with various preset intramolecular hydrogen bond systems and the conformation found in the X-ray structure) were subjected to geometry optimization, ultimately converging to two distinct low-energy conformers: **6**_**out**_ and **6**_**in**_ (Figure 3g,h). Conformer **6**_**out**_, with the lowest energy, has all OH groups directed outward, away from the anion binding cavity. In contrast, **6**_**in**_ is higher in energy by 2.60 kJ mol^–1^ and has all OH groups pointing toward each other, although too far apart to form intramolecular hydrogen bonds (O–H···O distances >4.0 Å). These results suggest that intramolecular hydrogen bonding between OH groups in receptor **6** is energetically unfavorable, in agreement with the X-ray structure.

ESP values, reflecting the cumulative influence of nearby atoms, are highly sensitive to molecular conformation. We therefore analyzed ESP values for the two geometry-optimized conformers, **6**_**in**_ and **6**_**out**_, as well as for a hypothetical model **6**_**complex**_ (derived from the energy-minimized complex **6**⊃Cl^–^ by removing Cl^–^ and performing single-point calculations, Figure 3i). These conformers differ primarily in the spatial arrangement of their OH groups. Comparing ESP values at equivalent positions within the binding cavity reveals interesting trends. For both **6**_**in**_ and **6**_**out**_, the ESP values within the aromatic CH regions of the lower rim are lower than those calculated for compound **4a** (which features CH_3_ groups instead of −(CH_2_)_3_–OH at the same location). This suggests that in these conformations, the neighboring oxygen atoms of the OH groups electrostatically diminish the positive potential. However, a significant enhancement in ESP values within the cavity was observed when the OH groups were directed inward (as in **6**_**complex**_), indicating that properly preorganized OH groups can indeed strengthen anion binding. However, the **6**_**complex**_ conformation appears to be unstable in the absence of an anionic guest, relaxing to the conformer superimposable with **6**_**in**_ upon energy minimization.

In summary, theoretical calculations indicate that CN-substituted receptors should exhibit superior anion-binding properties compared to all other derivatives, including the previously reported NO_2_-substituted **1**. However, from the ESP calculations alone it is not immediately obvious if the OH groups in the lower rim will support anion binding, because their contributions are highly conformation dependent and can be either positive or negative.

### Anion Binding Studies

The anion binding properties of resorcin[4]­arene receptors **1**–**7** were investigated by a combination of UV–Vis and ^1^H NMR titrations, conducted at either 298 K (quantitative studies) or 255 K (qualitative studies), and in various solvents (Table 1; see SI for experimental details). The anions were added as either tetrabutylammonium or tetrapentylammonium salts. For receptors **1** and **2**, some binding data were reported in our previous paper,^26^ but additional results were obtained in this study. Receptor **4** showed limited solubility in most organic solvents, allowing quantitative binding studies only by UV–Vis titrations (*C* = 4.36 × 10^–5^ M), while its ^1^H NMR titrations provided qualitative insights (Figure 4a).

**Figure 4:**
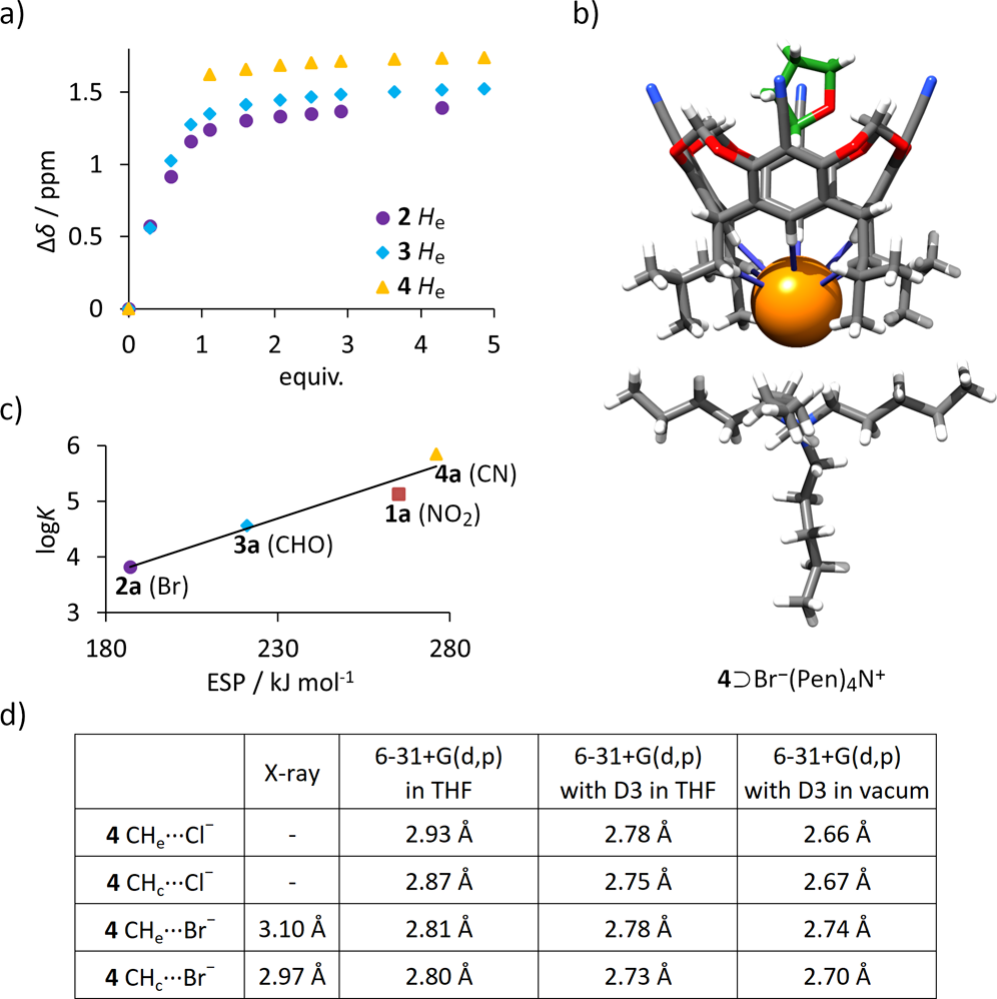
(a) ^1^H NMR shifts of H_e_ signals during titration of receptors **2**-**4** with Pen_4_NCl in THF-*d*_8_ (qualitative, due to broadening and disappearance of signals below 1 equiv); (b) X-ray structure of **4**⊃Br^–^ (THF in green, Br^–^ in orange); (c) correlation between experimental log*K*_a_ values and theoretically predicted ESPs; (d) CH···anion contacts found in the X-ray structure and energy-optimized complexes (DFT B3LYP with various basis sets).

**Table 1:** Apparent Association Constants (*K*_a_) for Receptors **1**–**4** Towards Cl^–^ and Br^–^ in THF and DCM-*d*_2_

Receptor	Anion	Solvent	*K*_a_/M^–1^ ^a^	Error^b^	Log*K*_a_
**1**	Cl^–^	THF	136 000 (UV–Vis)^26^	10%	5.14
**1**	Cl^–^	DCM-*d*_2_	81 (NMR)^26^	3%	1.91
**2**	Cl^–^	THF	6 700 (UV–Vis)^26^	3%	3.83
**3**	Cl^–^	THF	37 000 (UV–Vis)	4%	4.57
**4**	Cl^–^	THF	700 000 (UV–Vis)	25%	5.85
**4**	Br^–^	THF	600 000 (UV–Vis)	23%	5.78
**4**	Cl^–^	DCM-*d*_2_	320 (NMR)	2%	2.50

a All the titrations were performed at 298 K and were repeated at least twice.

b The error represented is the fitting error.

### Anion Binding in THF

^1^H NMR titrations of receptors **1**–**4** (bearing alkyl substituents on the lower rim) in THF-*d*_8_ demonstrated downfield shifts for protons H_e_ and H_c_ (Figure 4a), suggesting that anion binding occurs at the lower rim. This binding mode was confirmed by X-ray structure of **4**⊃Br^–^ complex (Pen)_4_N^+^ counterion, Figure 4b). In the complex, Br^–^ anion resides in the lower rim of receptor **4** forming short contacts with aromatic CH groups (C­(H_e_)···Br^–^ 4.03 Å, H_e_···Br^–^ 3.10 Å) and aliphatic CH groups (C­(H_c_)···Br^–^ 3.94 Å, H_c_···Br^–^ 2.97 Å).

The *K*_a_ values in THF were determined by UV–Vis titrations (fitted with 1:1 H:G binding model using BindFit^42,43^ ) as they were too high to be measured by ^1^H NMR titration method. The results indicate a pronounced influence of upper-rim substituents on binding affinity. Notably, the newly designed receptor **4** (bearing −CN substituents in the upper rim) exhibits the highest affinity of all alkyl-bearing receptors **1**–**4**, with *K*_a_(**4**, Cl^–^, THF) = 7.00 × 10^5^ M^–1^ being more than 5 times higher than *K*_a_(**1**, Cl^–^, THF). It is worth noting that in chlorinated solvents (DCM) substantial diminishing of binding affinities was observed, in agreement with previously reported trends,^26^ but still receptor **4** is more effective than **1**. Furthermore, it was found that the experimental log*K*_a_ values for receptors **1**–**4** correlate well with the theoretically predicted ESP values (Figure 4c). In contrast, the correlation with Hammett σ_para_ parameters are worse, likely due to the steric effects discussed above.

For receptors **5** and **6**, which feature hydroxyalkyl “feet” (receptor **7** being insoluble in THF), the ^1^H NMR binding isotherms show significant deviations from simple 1:1 stoichiometry (Figure 5a,b). The isotherms are steeper than expected for 1:1 binding and have inflection points at both 0.5 and 1.0 equiv (e.g., for H_e_ and OH signals), suggesting the initial formation of 2:1 host–guest (H_2_G) complexes, with varying relative contributions from H_e_ and OH binding groups. In receptor **5**, the lower ESP potential at H_e_ appears to shift the binding emphasis toward the OH groups. In contrast, for receptor **6**, signals of OH groups start to shift only after more than 1 equiv., indicating that binding is initially dominated by the H_e_ sites, while the OH signals remain less affected. Further addition of chloride, from 0.5 to 1.0 equiv, likely shifts the equilibrium from 2:1 host–guest complexes to 1:1 complex, as evidenced by the downfield shifts of H_e_ protons in both **5** and **6**. Notably, the OH signals remain largely unshifted in this range, indicating that their involvement in hydrogen bonding interactions is similar in both H_2_G and HG complexes. After more than one equivalent of chloride is added, further downfield shifts of the OH signals are observed, while H_e_ signals remain unchanged. This suggests the formation of HG_n_ (n > 1) complexes, most likely involving the OH groups. Because of these complex equilibria, the association constants for **5** and **6** could not be reliably determined, precluding direct comparison with receptors **1**–**4**. Instead, competitive pairwise titrations were performed to assess relative anion-binding strengths under identical conditions (Figure 5c,d). In the, experiment involving receptors **2** and **5**, the chemical shift changes for **5** were similar to those observed in individual-receptor titration, while signals of **2** remained largely unperturbed until binding of **5** approached saturation (Figure 5c). A similar behavior was observed in competitive experiments with **5** and **6** (Figure 5d), indicating that the chloride-binding affinities follow the order **2** < **5** < **6**, with differences of at least 2 orders of magnitude between receptors in each pair. These findings highlight the significant role of the lower rim OH groups in enhancing anion binding affinity.

**Figure 5:**
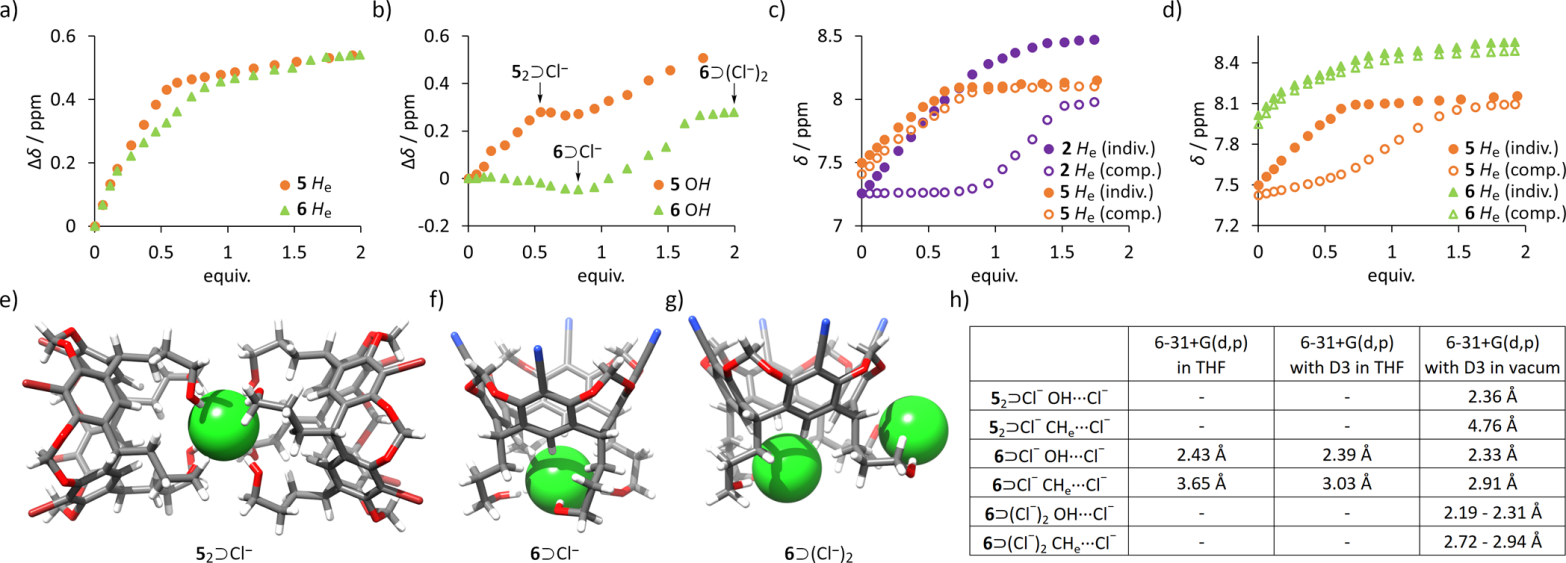
Changes in ^1^H NMR chemical shifts during individual titrations of receptors **5** and **6** with Pen_4_NCl for signals of: (a) H_e_; and (b) OH (*C* = 6.7 mM, THF-*d*_8_ at 255 K). Comparison of the changes in ^1^H NMR chemical shifts of H_e_ signals during competition and individual titrations for receptors: (c) **2**+**5**; and (d) **5**+**6** (*C*_2/5/6_ = 6.7 mM, THF-*d*_8_, all at 298 K). Energy-minimized structures of complexes with different stoichiometries (e–g). Relevant OH···Cl^–^ and CH···Cl^–^ contacts found in the energy-optimized complexes using DFT B3LYP with various basis sets (h).

### Anion Binding in THF:Water Mixtures

Given the strong binding observed in THF and the possible involvement of the OH groups, we extended our study to a more challenging (but also practically relevant) aqueous–organic medium (THF:water, 9:1, *v*:*v*). In this solvent mixture, we examined the affinities of receptors **1**–**7** toward chloride and tetrahedral oxyanions of biological and industrial importance: HSO_4_^–^, H_2_PO_4_^–^, ReO_4_^–^ (a structural analogue of radioactive TcO_4_^–^),^44^ and ClO_4_^–^ (a hydrophobic, weakly binding anion).

In THF-*d*_8_:D_2_O (9:1, *v*:*v*), the ^1^H NMR titration data for all receptors fitted well to 1:1 host–guest binding isotherms (Table 2). Receptors **5** and **7** showed similar binding affinities to all anions, suggesting that variations in the positioning of OH groups at the lower rim have little influence on binding. Receptor **6** demonstrated consistently higher binding affinities relative to receptors **5** and **7**, highlighting the importance of the −CN substituent at the upper rim in modulating binding strength. Notably, receptor **6** exhibited exceptional selectivity for HSO_4_^–^, with affinity exceeding that for other tetrahedral oxyanions by at least 1 order of magnitude. Qualitative inspection of the ^1^H NMR spectra provides further insight into the binding modes (Figure 6). Receptor **6** binds HSO_4_^–^ and H_2_PO_4_^–^ in the lower rim (with H_e_ signals experiencing downfield shifts of Δδ = +0.39 and +0.20 ppm, respectively), while ReO_4_^–^ and ClO_4_^–^ interact primarily with the upper rim (H_e_ signals experience only minimal upfield shifts of Δδ = −0.04 ppm in both cases, while internal methylene bridge signals shift downfield by Δδ = +0.21 and +0.31 ppm, respectively).

**Table 2:** Apparent Association Constants (*K*_a_) for Receptors 1–7 Towards Anions in THF-*d*_8_:D_2_O 9:1 (*v*:*v*)

Receptor	Anion	Hydration Energy/kJ mol^–1^	*K*_a_/M^–1^ ^a^	Error	Log*K*_a_
**1**	Cl^–^	–363	500	3%^26^	2.70
**1**	ClO_4_^–^	–245	3 800	7%^26^	3.58
**1**	HSO_4_^–^	–368	130	3%	2.11
**2**	Cl^–^	–363	<5	-	<0.70
**2**	HSO_4_^–^	–368	<5	-	<0.70
**3**			*Insoluble		
**4**			*Insoluble		
**5**	Cl^–^	–363	112	2%	2.05
**5**	HSO_4_^–^	–368	53	1%	1.72
**5**	H_2_PO_4_^–^	–522	<5		<0.70
**5**	ClO_4_^–^	–245	24	2%	1.38
**6**	Cl^–^	–363	385	3%	2.59
**6**	NO_3_^–^	–330	215	2%	2.33
**6**	HSO_4_^–^	–368	523	2%	2.72
**6**	H_2_PO_4_^–^	–522	30	1%	1.48
**6**	ClO_4_^–^	–245	50	1%	1.70
**6**	ReO_4_^–^	–330	15	1%	1.18
**7**	Cl^–^	–363	160	2%	2.20
**7**	HSO_4_^–^	–368	17	2%	1.23
**7**	ClO_4_^–^	–245	43	4%	1.63
**7**	H_2_PO_4_^–^	–522	<5	-	<0.70
**7**	ReO_4_^–^	–330	46	4%	1.66

a Association constants were determined by ^1^H NMR titrations at 298 K using Pen_4_NX or Bu_4_NX salts and titration isotherms were fitted with 1:1 binding model using BindFit.^42,43^

**Figure 6:**
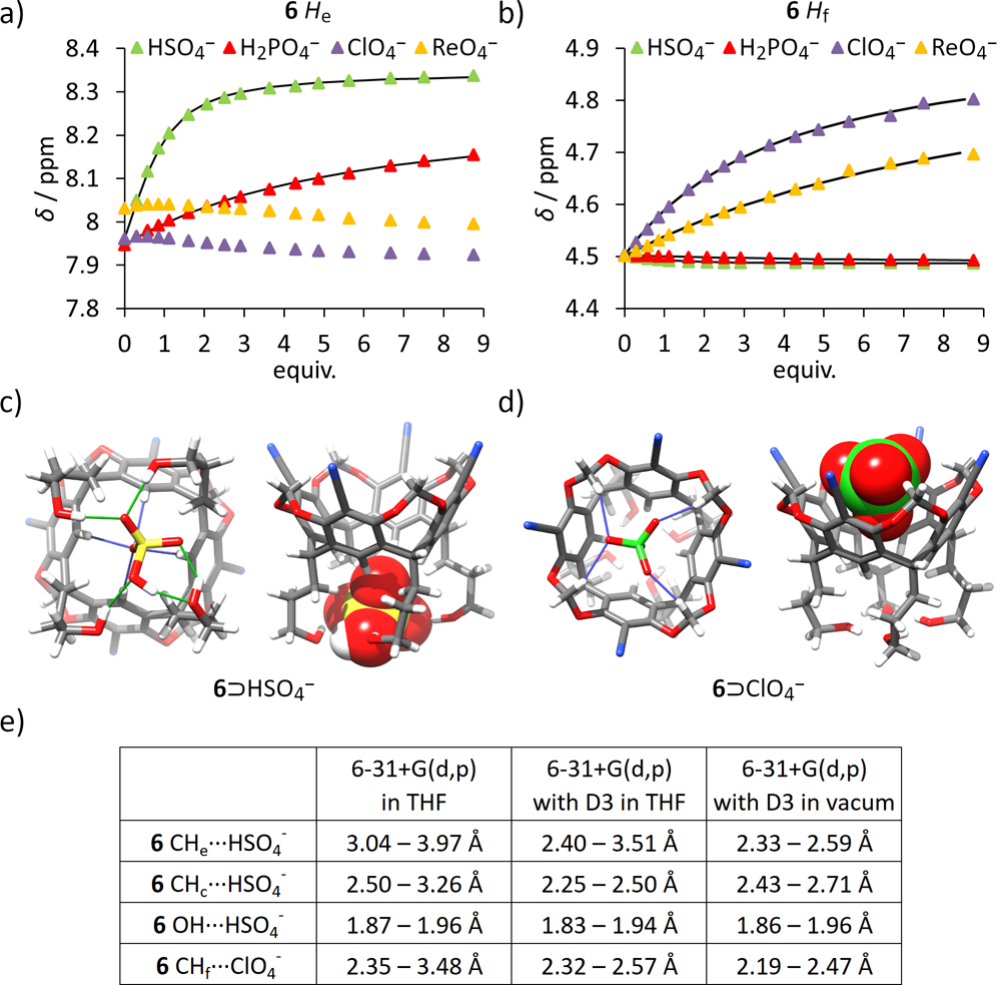
Binding of tetrahedral oxyanions by **6** in THF-*d*_8_:D_2_O (9:1, *v*:*v*). Changes in chemical shifts of receptor **6** during titrations with various anions: (a) H_e_; (b) H_f_. Geometry-optimized structures of complexes: (c) **6**⊃HSO_4_^–^; (d) **6**⊃ClO_4_^–^ (DFT B3LYP/6–31+G­(d,p) in vacuum); (e) OH···O and CH···O contacts found in the energy-optimized complexes using DFT B3LYP with various basis sets.

In mixed aqueous–organic environments such as THF-*d*_8_:D_2_O (9:1, *v*:*v*), anion binding is strongly affected by hydration energies, because both the anions and the binding sites of receptors are significantly hydrated. Accordingly, for a given receptor and anion, the same feature may have both positive and negative effects on binding; for instance, anions and receptors that form stronger hydrogen bonds are also more strongly hydrated.^45^ Therefore, it is challenging to predict the binding affinities in aqueous–organic mixtures, and there are no “typical trends” known from purely organic solvents. In THF-*d*_8_:D_2_O (9:1, *v*:*v*), receptors **5**–**7** are likely more strongly hydrated (especially in the OH region) than their analogs **1**–**4** with *i*-butyl tails. Accordingly, in this aqueous–organic environment, receptor **6** shows lower affinity toward Cl^–^ than receptor **1**, likely due to stronger receptor hydration. By the same virtue, the hydrophobic ClO_4_^–^ is completely excluded from the lower-rim binding site of receptor **6** and binds weakly only at the upper rim. It should be noted that binding of large hydrophobic anions in the upper rim of resorcin[4]­arenes was observed previously in organic media.^13,39,46^

Thus, in an aqueous–organic environment, the selectivities of receptors **6** and **1** are opposite: *K*_a_(Cl^–^)/*K*_a_(ClO_4_^–^) is 0.13 for receptor **1**, whereas for receptor **6** it is 1.4. This contrast is even more pronounced in the case of HSO_4_^–^, which is more hydrophilic than Cl^–^: receptor **1** has *K*_a_(HSO_4_^–^)/*K*_a_(ClO_4_^–^) = 0.03, whereas for receptor **6** this ratio exceeds 10. It is also worth noting that receptor **6** effectively discriminates between similarly shaped anions: HSO_4_^–^ and H_2_PO_4_^–^ - with a selectivity factor of 17, likely due to substantial differences in hydration energies and hydrogen bonding with the OH groups, as suggested by geometry optimization (Figure 6c-e).

### Anion Transport across Lipid Bilayers

The inherent lipophilicity, chemical robustness, and resistance to deprotonation make CH-bonding receptors promising candidates for the development of transmembrane anion transporters. Such systems are of considerable interest due to the essential role of anion transport in numerous physiological and pathological processes.^47–49^ We have previously demonstrated that resorcinarene **1** functions as a highly potent and selective Cl^–^/NO_3_^–^ antiporter.^26^ Here, we extend our investigations to the complete series of receptors **1**–**7**.

The chloride transport activity of receptors **1**–**7** was evaluated using large unilamellar vesicles (LUVs, 200 nm in diameter) composed of 1-palmitoyl-2-oleoyl-*sn*-glycero-3-phosphocholine (POPC) and encapsulating the halide-sensitive dye lucigenin (Figure 7a). LUVs were prepared in 225 mM NaNO_3_, with receptors preincorporated into the lipid bilayer at a receptor:lipid molar ratio of 1:500, except for receptor **1**, which was tested at 1:4000 due to its substantially higher activity. Chloride influx was initiated by the addition of 1 M NaCl to afford the extravesicular chloride concentration of 25 mM, and transport kinetics was monitored via chloride-induced quenching of lucigenin fluorescence.^50^

**Figure 7:**
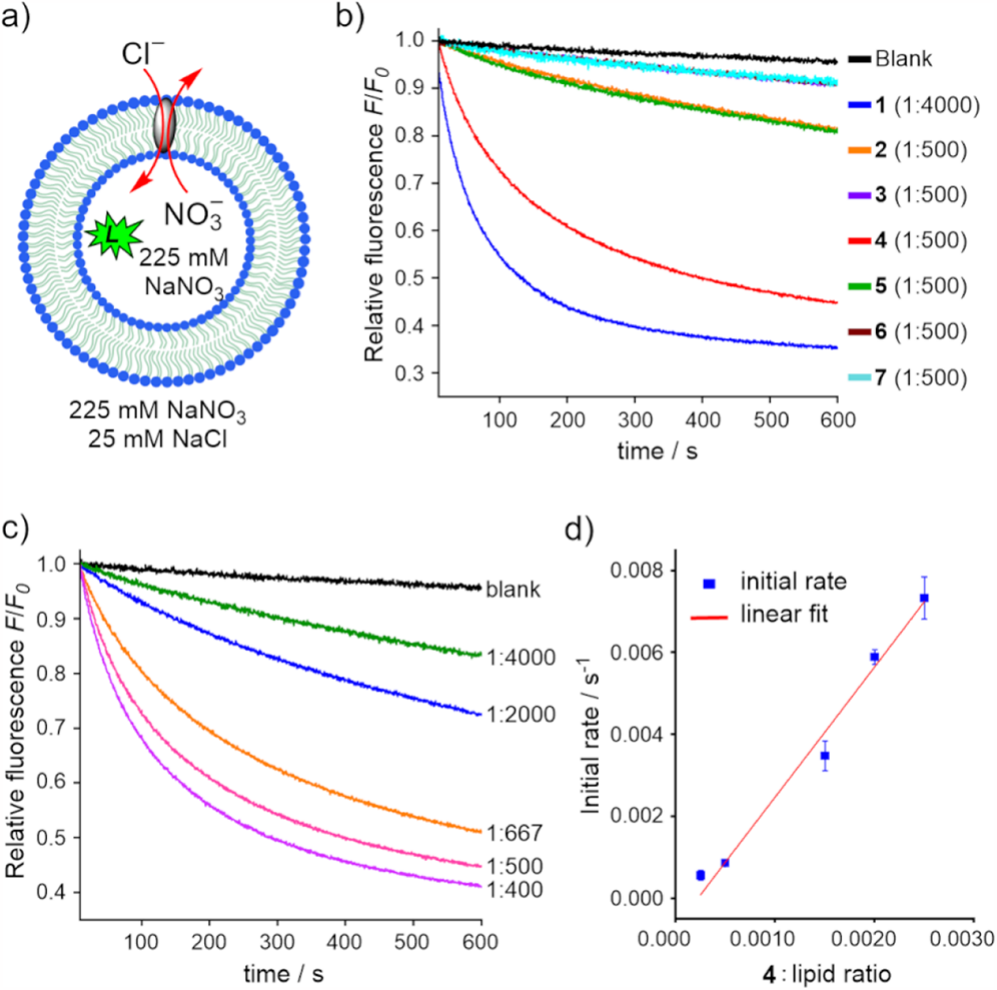
Cl^–^ transport by receptors **1**-**7**: (a) schematic representation of POPC LUVs containing chloride-sensitive dye lucigenin, used for studying Cl^–^/NO_3_^–^ exchange studies; (b) changes in relative fluorescence (*F*/*F*_0_) due to Cl^–^ influx into POPC LUVs mediated by receptors **1**-**7**, preincorporated into the lipid membrane at 1:500 or 1:4000 transporter-to-lipid molar ratios; (c) changes in relative fluorescence (*F*/*F*_0_) due to Cl^–^ influx into POPC LUVs mediated by receptor **4**, preincorporated in the lipid membrane at transporter-to-lipid molar ratios ranging from 1:4000 to 1:400; (d) initial Cl^–^ transport rates for receptor **4** as a function of transporter-to-lipid molar ratio with corresponding linear fit.

Of the seven receptors studied, only **1** and **4** exhibited significant chloride transport activity under these conditions (Figure 7b). The remaining receptors were either weakly active or inactive. The observed trend in transport efficiency: **1** > **4** > (**2**, **5**) > (**3**, **6**, **7**) ≈ 0, clearly demonstrates that transport activity does not correlate with chloride binding affinities, measured in THF or THF:water mixtures. Notably, receptor **1** (nitro-substituted) outperforms **4** (cyano-substituted), despite being a significantly weaker chloride binder. Similarly, the hydroxyl-footed receptors **5**–**7**, which displayed strong chloride binding, were essentially inactive. These findings reinforce the well-established notion that binding strength alone is a poor predictor of transmembrane transport activity.^51^

To probe the molecular features governing transport activity, we assessed the lipophilicity of receptors **1**–**7** both computationally (using ChemDraw and ChemAxon’s Playground) and experimentally, via reversed-phase HPLC (Figure 8a, see SI for details). All four *i*-butyl-footed receptors **1**–**4** were found to be highly lipophilic (estimated log*P* > 10), whereas hydroxyl-footed receptors **5** and **6** are significantly less so. The lipophilicity of receptor **7** could not be determined experimentally due to limited solubility, but both ChemDraw and Playground predict it to be very high, despite the presence of four hydroxyl groups. Thus, with the possible exception of **6**, all receptors appear sufficiently lipophilic to remain in the membrane, rather than leach into the aqueous phase.

**Figure 8:**
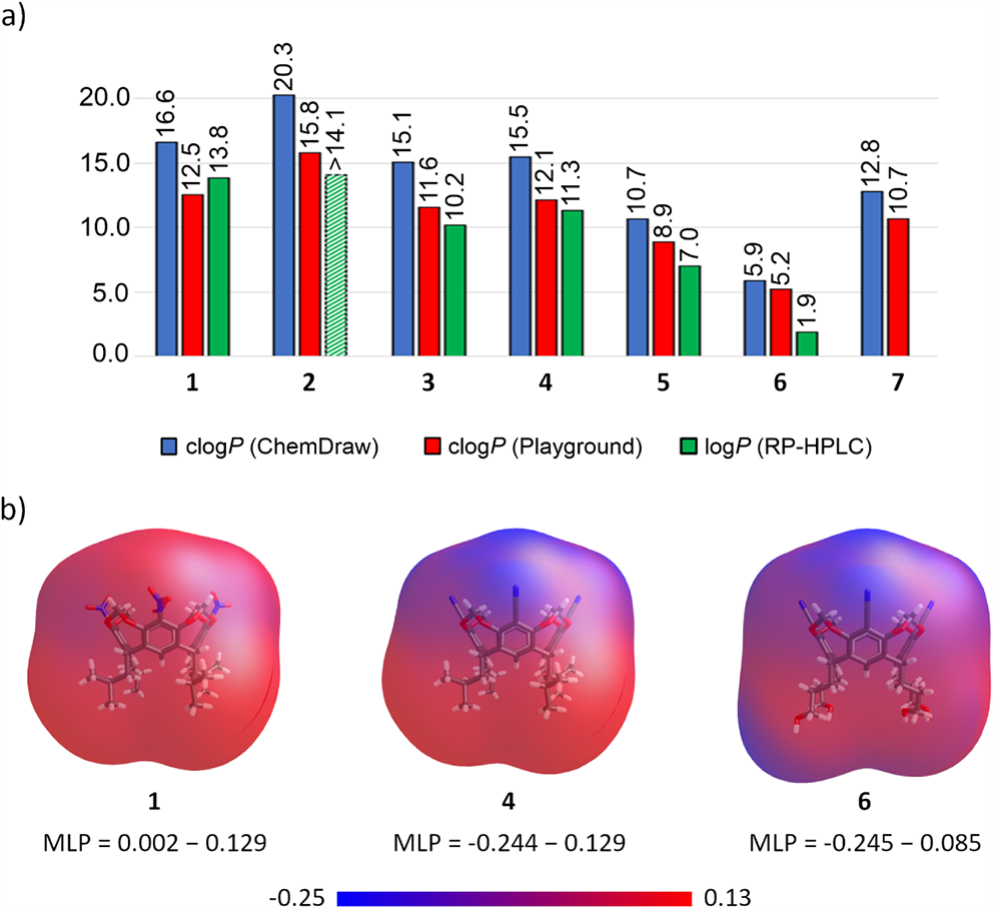
(a) Comparison of log*P* values estimated computationally by ChemDraw and Playground and experimentally (from RP-HPLC measurements); (b) Molecular Lipophilicity Potential (MLP, generated in Vega ZZ software)^52^ maps of selected receptors, showing spatial distribution of hydrophobic (red) and hydrophilic (blue) regions across the 3D molecular surface. MLP analysis highlights the distinctly more hydrophilic upper rim of receptor **4** compared to **1**.

However, global lipophilicity parameters such as log*P* do not capture the full picture. Local lipophilicity descriptors, such as Molecular Lipophilicity Potential (MLP) mapped on the molecule’s 3D surface (Figure 8b), offer more insight. MLP maps show that the lower rims of receptors **5**–**7**, bearing four hydroxyl groups each, are distinctly hydrophilic and thus likely orient toward the aqueous phase. While this orientation may facilitate anion capture, the strong affinity of the hydroxyl groups to water may anchor these receptors at the lipid–water interface, hindering their diffusion across the bilayer and limiting their transport activity.

A similar, but more subtle, effect may account for the lower activity of receptor **4** relative to **1**. Both compounds are highly lipophilic, but **4** is markedly less so, with log*P* = 11.3 vs 13.8 for **1**. Since the two receptors differ only in their upper-rim substituents (CN vs NO_2_), the upper rim of **4** must be much more hydrophilic than that of **1**. This notion is supported by MLP maps (Figure 8b), and is also consistent with the higher hydrogen-bond basicity of the cyano group (Abraham’s β parameter for benzonitrile is 0.33), compared with nitro (β = 0.28 for nitrobenzene).^53^ As a consequence, the exposure of the lower rim to the aqueous phase - which is necessary for anion sequestration - might be energetically more costly for **4**, reducing its transport efficiency.

To assess whether the activity of receptor **4** might be limited by aggregation or poor membrane solubility, we quantified its transport kinetics at varying membrane loadings (Figure 7c). Initial transport rates, obtained by fitting fluorescence traces to a biexponential function, scaled linearly with concentration up to 0.25 mol% (Figure 7d), consistent with monomeric carrier mechanism and good solubility. At higher loadings, a plateau in activity was observed, likely due to precipitation or aggregation (see SI).

Overall, our results support a mobile carrier mechanism for receptors **1** ^26^ and **4**, and highlight the importance of well-balanced lipophilicity^54^ for effective anion transport. Amphiphilic resorcinarenes with strongly hydrophilic lower rims (**5**-**7**) show little or no transport ability, likely because they remain anchored at the membrane interface, which hinders their transmembrane diffusion and limits their overall transport performance. The comparison of **1** and **4** shows that even modest differences in upper- and lower-rim polarity can significantly impact transport efficiency, possibly by affecting the receptor’s reorientation dynamics. If the upper rim is substantially more hydrophilic than the anion-binding lower rim, the receptor may fail to adopt the orientation required for efficient anion sequestration from the aqueous phase. These mechanistic and structure–activity insights offer a set of clear design principles for rationally developing resorcinarene-based transporters with enhanced performance.

## Conclusions

In conclusion, we have investigated a series of resorcin[4]­arenes functionalized at both the upper and lower rims as anion receptors. Systematic variation of the electron-withdrawing character of the upper rim substituents resulted in marked enhancement of binding affinity, reaching *K*_a_(Cl^–^, THF) = 7.0 × 10^5^ M^–1^ for the CN-substituted receptor. Notably, the binding affinities show a strong correlation with the surface electrostatic potential (ESP) at the binding site, calculated using DFT methods, while correlations with Hammett parameters are disrupted due to steric effects.

Modification of the lower rim with hydroxyl-bearing alkyl groups led to further enhancement of binding affinity and the formation of higher-order complexes with anions (H_2_G and HG_n_, n > 1). These OH-footed resorcin[4]­arenes effectively bind anions even in challenging aqueous–organic environments and exhibit remarkable selectivity for HSO_4_^–^ over other tetrahedral, singly charged oxyanions (e.g., *K*_a_(HSO_4_^–^)/*K*_a_(H_2_PO_4_^–^) = 17). Additionally, site-selective binding, dependent on the hydrogen bonding capabilities of the guest, was observed.

Anion transport studies using large unilamellar vesicles showed that the tetranitro-substituted resorcin[4]­arene is the most effective chloride transporter in this series, outperforming even the most strongly binding tetracyano analog. In contrast, receptors bearing hydroxyalkyl groups showed very low transport activity, despite their high chloride affinity. These findings demonstrate that strong anion binding alone is not sufficient for efficient transport, and suggest that the optimal lipophilicity balance between the upper and lower rims is key to the effective anion translocation across lipid bilayers by this class of transporters.

Overall, our findings clearly demonstrate that strategic peripheral modifications of resorcin[4]­arene scaffold provide a powerful means to fine-tune both the strength and selectivity of anion binding. Given the extensive diversity of resorcin[4]­arene scaffolds available in the literature, this approach holds considerable promise for designing receptors capable of targeting anions of various shapes and charges, including chiral anions.

## Supplementary Material



## Data Availability

The data that support the findings of this study (NMR spectra, titration data, anion transport data, theoretical calculations) are openly available in OPEN at 10.18150/0BWTLG.

## Notes

Details of synthetic procedures, NMR spectra, titration protocols, DFT calculation details, coordinates for the optimized structures, detailed protocols, and results of anion transport studies (PDF)
