# Supplementary material for: Tunable Anion Recognition at the Lower Rim of Resorcin[4]arenes: Strength, Selectivity, and Transport
Source: JACS Au. 2025 Sep 29;5(10):5137–46. doi: 10.1021/jacsau.5c01041 (PMC12569696; doi:10.1021/jacsau.5c01041)
Supplement: Supplementary file 1 [file au5c01041_si_001.pdf]

Supporting Information

Tuneable anion recognition at the lower rim of resorcin[4]arenes: strength, selectivity, and transport

Deepshikha Priyadarshini,[a] Ronedy Naorem,[b] Marek P. Szymański,[a] , Oksana Danylyuk,[c] Michał J. Chmielewski,[b,]* Agnieszka Szumna [a ,]*

[a] Institute of Organic Chemistry, Polish Academy of Sciences, Kasprzaka 44/52, 01-224 Warsaw, Poland.

[b] University of Warsaw, Faculty of Chemistry, Biological and Chemical Research Centre, Żwirki i Wigury 101, Warsaw, Poland.

[c] Institute of Physical Chemistry, Polish Academy of Sciences, Kasprzaka 44/52, 01-224 Warsaw, Poland.

TABLE OF CONTENTS

1. **GENERAL INFORMATION**.....5

2. **SYNTHESIS**.....7

2.1. Synthesis of receptors **2** and **4**.....7

2.2 Synthesis of receptor **3**.....11

2.3 Synthesis of receptors **5** and **6**.....13

2.4 Synthesis of receptor **7**.....17

3. **NMR SPECTRA OF RECEPTORS**.....20

4. **TITRATIONS**.....22

General procedures for titrations.....22

NMR titration of **1** with But_4NHSO_4 in THF-d_8/10% D_2O.....24

NMR titration of **2** with Pen_4NCl in THF-d_8/ 10% D_2O.....26

NMR titration of **2** with But_4NHSO_4 in THF-d_8/ 10%D_2O.....28

UV-vis titration of **3** with Pen_4NCl in THF.....29

NMR titration of **3** with Pen_4NCl in THF-d_8.....31

NMR titration of **3** with But_4NPhCO_2 in THF-d_8.....33

UV-vis titration of **4** with Pen_4NCl in THF.....35

UV-vis titration of **4** with Pen_4NBr in THF.....37

UV-vis titration of **4** with Pen_4NCl in DCM.....39

UV-vis titration of **4** with Pen_4NCl in $CHCl_3$.....41

NMR titration of **4** with Pen_4NCl in $CDCl_3$.....42

NMR titration of **4** with Pen_4NCl in DCM-d_2.....44

NMR titration of **4** (lower concentration) with Pen_4NCl in DCM-d_2.....46

NMR titration of **4** with Pen_4NCl in THF-d_8 48

NMR titration of **5** with Pen_4NCl in THF-d_8 at 255 K 50

NMR titration of **5** with Pen_4NBr in THF-d_8 at 255 K 52

NMR titration of **5** with But_4NReO_4 in THF-d_8 at 255 K 54

NMR titration of **5** with Pen_4NCl in THF-d_8/10% D_2O 56

NMR titration of **5** with But_4NHSO_4 in THF-d_8/10% D_2O 58

NMR titration of **5** with $But_4NH_2PO_4$ in THF-d_8/10% D_2O 60

NMR titration of **5** with But_4NClO_4 in THF-d_8/10% D_2O 62

NMR titration of **6** with Pen_4NCl in THF-d_8 at 255 K 64

NMR titration of **6** with Pen_4NBr in THF-d_8 at 255 K 66

NMR titration of **6** with But_4NReO_4 in THF-d_8 at 255 K 68

NMR titration of **6** with But_4NNO_3 in THF-d_8 at 255 K 70

NMR titration of **6** with Pen_4NCl in THF-d_8/10% D_2O 72

NMR titration of **6** with But_4NNO_3 in THF-d_8/10% D_2O 74

NMR titration of **6** with But_4NHSO_4 in THF-d_8/10% D_2O 76

NMR titration of **6** with $But_4NH_2PO_4$ in THF-d_8/10% D_2O 78

NMR titration of **6** with But_4NClO_4 in THF-d_8/10% D_2O 80

NMR titration of **6** with But_4NReO_4 in THF-d_8/10% D_2O 82

NMR titration of **7** with Pen_4NCl in THF-d_8/10% D_2O 84

NMR titration of **7** with But_4NHSO_4 in THF-d_8/10% D_2O 86

NMR titration of **7** with But_4NClO_4 in THF-d_8/10% D_2O 88

NMR titration of **7** with $But_4NH_2PO_4$ in THF-d_8/10% D_2O 90

NMR titration of **7** with But_4NReO_4 in THF-d_8/10% D_2O 91

5. **COMPETITION EXPERIMENTS** 93

Receptor **2** and receptor **5** with Pen_4NCl in THF-d_8 at 298 K........................93

Receptor **5** and receptor **6** with Pen_4NCl in THF-d_8 at 298 K........................95

6. **SINGLE CRYSTAL X-RAY DIFFRACTION**..96

7. **ANION TRANSPORT STUDIES**..100

Materials..100

Instruments and Methods...100

General Procedure for the Preparation of LUVs with Pre-Incorporated Transporters: 101

Data Acquisition 102

Processing the Data from the Lucigenin Assay:...103

Preparation of Stock Solution of Transporters: 105

Results of Cl^- Transport Experiments in $NaNO_3$ Medium Using Lucigenin-Loaded LUVs with Pre-Incorporated Resorcin[4]arene Transporters 106

Results from Concentration-Dependent Cl^- Transport Experiments Using Lucigenin-Loaded LUVs with Pre-Incorporated 4.......................................108

Fitting Anion Transport Data for Resorcin[4]arene 1: Calculation of Half-Life Time Values:...118

Fitting Anion Transport Data for Resorcin[4]arene 4: Calculation of Initial Rate Values 119

Fitting Anion Transport Data for Resorcin[4]arene 1: Calculation of Initial Rate Values 122

Estimation of log*P* 123

8. **THEORETICAL CALCULATIONS** 128

1. GENERAL INFORMATION

All solvents and chemicals used were purchased from Sigma Aldrich, TCI Europe N.V., Roth and Euriso-top, were of reagent grade and were used without further purification.

^{1}H NMR spectra were recorded at 298 K or 255 K on Bruker 400 MHz, Varian 500 or Varian 600 MHz instruments. The NMR spectra were referenced to the solvent residual signal (^{1}H: δ_{THF} = 3.58 ppm, δ_{DMSO} = 2.50 ppm, δ_{CHCl3} = 7.26 ppm, δ_{DCM} = 5.32 ppm, $\delta_{acetone}$ = 2.05 ppm; ^{13}C: δ_{THF} = 67.21 ppm, δ_{DMSO} = 39.52 ppm, δ_{CHCl3} = 77.16 ppm). UV-Vis spectra were measured on U-1900 Spectrophotometer. High resolution ESI mass spectra were recorded on a SYNAPT spectrometer.

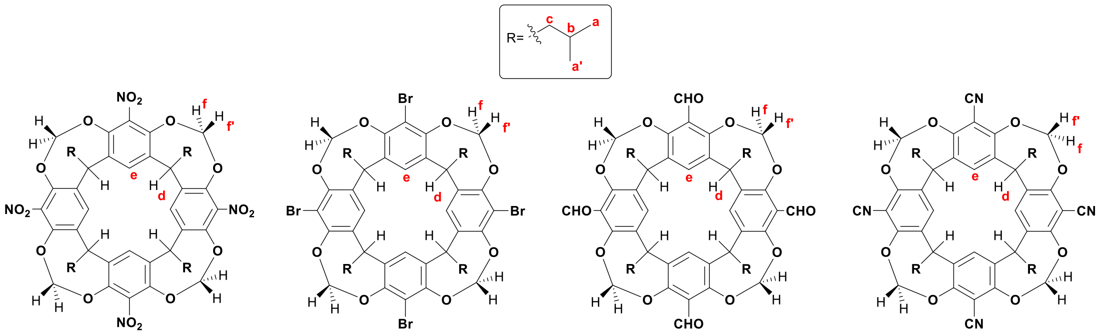

1 R= i-butyl | **2** R= i-butyl | **3** R= i-butyl | **4** R= i-butyl

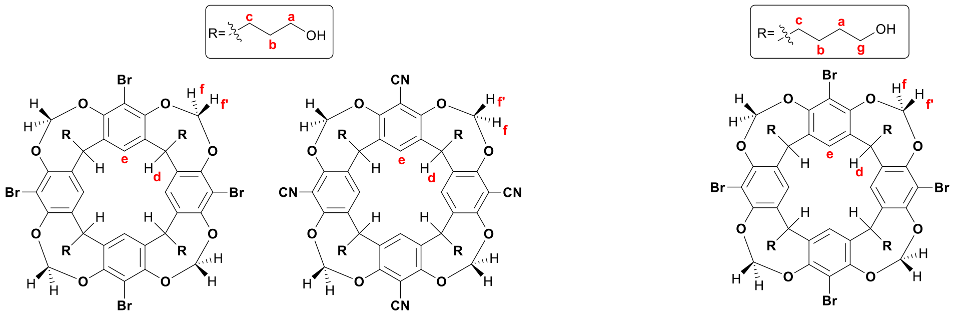

5 R =$(CH_2)_3$-OH | **6** R = $(CH_2)_3$-OH | **7** R =$(CH_2)_4$-OH

Figure S1. Chemical structures of the compounds used in this work and notation of their NMR signals.

2. SYNTHESIS

2.1. Synthesis of receptors 2 and 4

Synthesis of compound **S1**

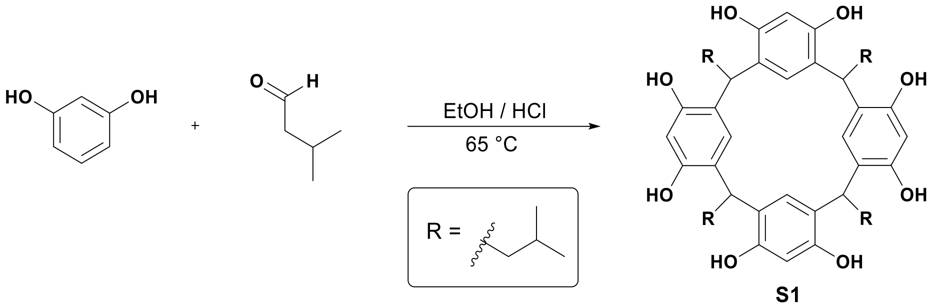

S1

The synthesis of compound **S1** was adapted from literature.[1] To a stirred solution of resorcinol (5.03 g, 45.4 mmol) in a 1:1 mixture of water (15 ml) and ethanol (15 ml) at 0 °C, isovaleraldehyde (3.90 g, 45.4 mmol) was added dropwise. The reaction mixture was stirred at 0 °C for 1 h, then heated to 65 °C and stirred for 4 days, during which a significant amount of precipitate formed. After cooling to room temperature, the precipitate was collected by filtration, washed multiple times with water, and dried under high vacuum to give **S1** as an orange solid. Yield: 7.32 g (86%).

The analytical data agree with the literature.[1]

^{1}H NMR (400 MHz, 298 K, $(CD_3)_2CO$) δ [ppm] = 8.57 (s, 8H), 7.52 (s, 4H), 6.25 (s, 4H), 4.46 (t, J = 8.0 Hz, 4H), 2.16 (dd, J = 8.0, 6.8 Hz, 8H), 1.47 (m, J = 6.6 Hz, 4H), 0.94 (d, J = 6.6 Hz, 24H).

[1] Tunstad, L. M.; Tucker, J. A.; Dalcanale, E.; Weiser, J.; Bryant, J. A.; Sherman, J. C. & Cram, D. J. Host-guest complexation. 48. Octol building blocks for cavitands and carcerands. *J. Org. Chem.* **1989**, *54*, 1305-1312.

Synthesis of compound **S2**

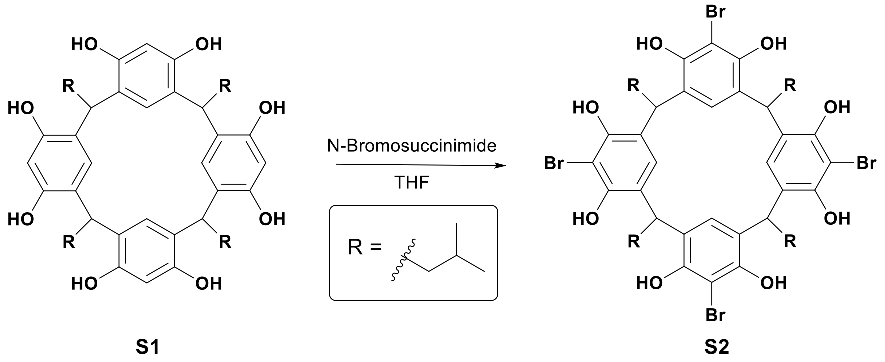

The synthesis of compound **S2** was adapted from literature procedures reported for similar compounds.[2] Compound **S1** (2.030 g, 2.81 mmol) was dissolved in THF (20 ml) and cooled to 0 °C. *N*-Bromosuccinimide (1.910 g, 10.7 mmol) was added portion wise over 15 minutes. The reaction mixture was allowed to warm to room temperature and stirred for 24 h. The solvent was then removed under reduced pressure to yield a dark yellow solid. Methanol (10 ml) was added to induce precipitation; the resulting solid was collected by filtration and dried under vacuum to give compound **S2** as a colourless powder. Yield: 1.230 g, (37%).

The analytical data are consistent with those reported in the literature.[2]
^{1}H NMR (400 MHz, 298 K, DMSO-d_6) δ [ppm] = 9.13 (s, 8H), 7.36 (s, 4H), 4.50 (t, J = 7.8 Hz, 4H), 2.07 (t, J = 7.3 Hz, 8H), 1.36 (m, J = 13.3, 6.6 Hz, 4H), 0.92 (d, J = 6.6 Hz, 24H).

[2] Abdurakhmanova, E. R.; Cmoch, P.; & Szumna, A. Three modes of interactions between anions and phenolic macrocycles: a comparative study. *Org. Biomol. Chem.* **2022**, *20*, 5095-5103.

Synthesis of receptor **2**

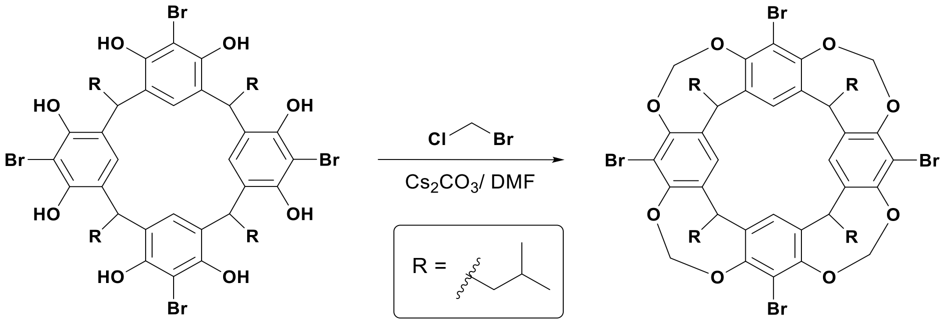

The synthesis of compound **2** was adapted from literature procedures reported for similar compounds.[3] Compound **S2** (1.210 g, 0.971 mmol) was weighed in a Schlenk tube and dissolved in anhydrous DMF (30 ml). Under an argon atmosphere, caesium carbonate (3.400 g, 9.7 mmol) was added. After stirring for approximately 30 min., bromochloromethane (5.030 g, 38.8 mmol) was added. The reaction mixture was stirred at 80 °C for 4 days. After cooling down to room temperature, the mixture was filtered, and the precipitate was washed multiple times with DCM. The combined filtrate was, then successively extracted with brine (×2 times) and water (×2 times). The organic layers were dried over anhydrous $MgSO_4$, filtered, and concentrated under reduced pressure to yield an oily residue, which was further triturated with methanol to give a colourless solid. Yield: 0.980 g (96%).

The analytical data are consistent with those reported in the literature.[3]
^{1}H NMR (400 MHz, 298 K, $CDCl_3$) δ [ppm] = 7.03 (s, 4H), 5.97 (d, J = 7.3 Hz, 4H), 5.00 (t, J = 8.1 Hz, 4H), 4.40 (d, J = 7.3 Hz, 4H), 2.09 (t, J = 7.5 Hz, 8H), 1.62 – 1.55 (m, 4H), 1.02 (d, J = 6.6 Hz, 24H).

[3] Mendez-Arroyo, J.; d'Aquino, A. I.; Chinen, A. B.; Manraj, Y. D.; & Mirkin, C. A. Reversible and selective encapsulation of dextromethorphan and β-estradiol using an asymmetric molecular capsule assembled via the weak-link approach. *J. Am. Chem. Soc.* **2017**, *139*, 1368-1371.

Synthesis of receptor **4**

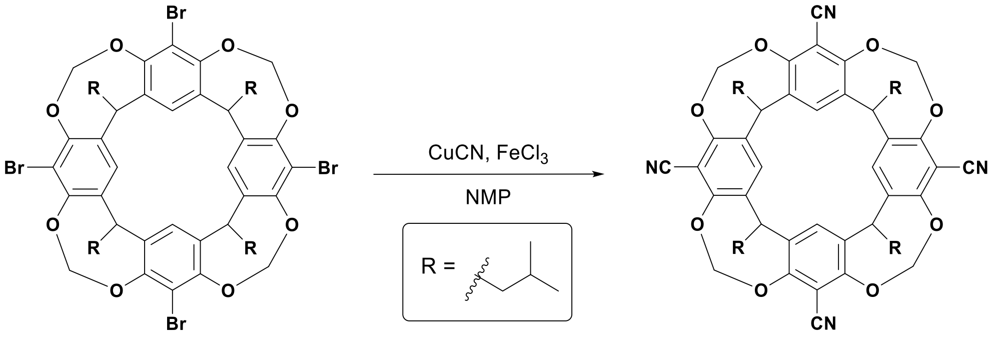

2 **4**

The synthesis of compound **4** was carried out following literature procedures for similar compounds.[4] Compound **2** (325 mg, 0.358 mmol) was dissolved in *N*-methylpyrrolidinone (NMP, 10 ml), and CuCN (320 mg, 3.576 mmol) was added. The reaction mixture was stirred under reflux for 24h. After cooling down to 80 °C, anhydrous $FeCl_3$ (928 mg, 5.722 mmol) was added, and the mixture was stirred for an additional 12h. Upon cooling to room temperature, the resulting precipitate was filtered off, washed multiple times with water, and dried under high vacuum to give a dark brown solid. The crude product was purified by flash chromatography [$CHCl_3$: methanol, 99:1 (*v*/*v*)] to give **4** as a colourless solid. Yield: 100 mg (42%).

^{1}H NMR (400 MHz, 298 K, $CDCl_3$) δ [ppm] = 7.26 (s, 4H), 6.10 (d, J = 7.4 Hz, 4H), 4.94 (t, J = 8.1 Hz, 4H), 4.58 (d, J = 7.4 Hz, 4H), 2.09 (t, J = 7.5 Hz, 8H), 1.62 – 1.55 (m, 4H), 1.03 (d, J = 6.7 Hz, 24H).

^{13}C NMR (151 MHz, $CDCl_3$) δ [ppm] = 156.3, 139.3, 125.0, 112.1, 104.6, 98.9, 38.1, 34.3, 29.8, 26.3, 22.8.

HR-MS: *m*/*z* $[M+H]^+$ calcd for $C_{52}H_{53}N_4O_8$: 861.3863, found: 861.3870

[4] Fochi, F.; Jacopozzi, P.; Wegelius, E.; Rissanen, K.; Cozzini, P.; Marastoni, E.; Fisicaro, E.; Manini, P.; Fokkens, R.; Dalcanale, E. Self-assembly and anion encapsulation properties of cavitand-based coordination cages. *J. Am. Chem. Soc.* **2001**, *123*, 7539–7552.

2.2 Synthesis of receptor 3

Synthesis of compound **S3**

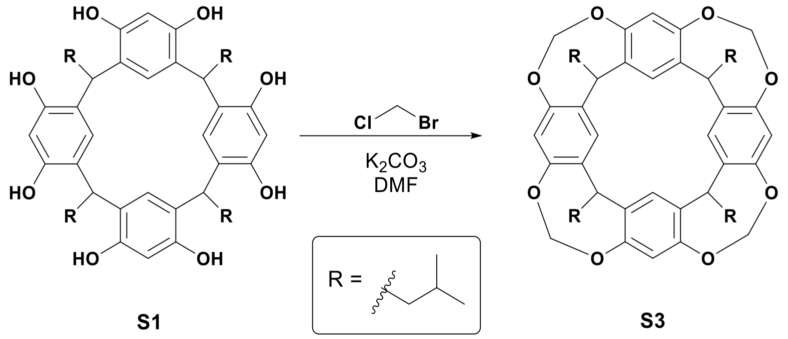

The synthesis of compound **S3** was carried out following literature procedures.[5] To a solution of **S1** (2.010 g, 2.81 mmol) in DMF (40 ml), potassium carbonate (5.205 g, 37.65 mmol) and bromochloromethane (1.32 ml, 19.7 mmol) were added. The reaction mixture was stirred at 55 °C for 24 h, then heated to 80 °C and stirred for an additional 48h. After cooling, the mixture was filtered and the solvent was evaporated under reduced pressure. The residue was dissolved in chloroform, filtered and concentrated. Ethyl acetate was added to the residue, resulting in the formation of a white precipitate, which was collected and dried to afford the compound **S3**. Yield: 1.160 g (54%).

The analytical data are consistent with those reported in the literature.[5]

^{1}H NMR (400 MHz, 298 K, DMSO-d_6) δ [ppm]) = 7.55 (s, 4H), 6.51 (s, 4H), 5.72 (d, J = 7.6 Hz, 4H), 4.72 (t, J = 8.1 Hz, 4H), 4.39 (d, J = 7.6 Hz, 4H), 2.24 (t, J = 7.5 Hz, 8H), 1.49 (m, 4H), 0.97 (d, J = 6.6 Hz, 24H).

[5] Jurek, P.; Jędrzejewska, H.; Rode, M. F.; Szumna, A. Recognition-Induced Enhanced Emission of Core-Fluorescent ESIPT-type Macrocycles. *Chem. Eur. J.* **2023**, *29*, e202203116.

Synthesis of receptor **3**

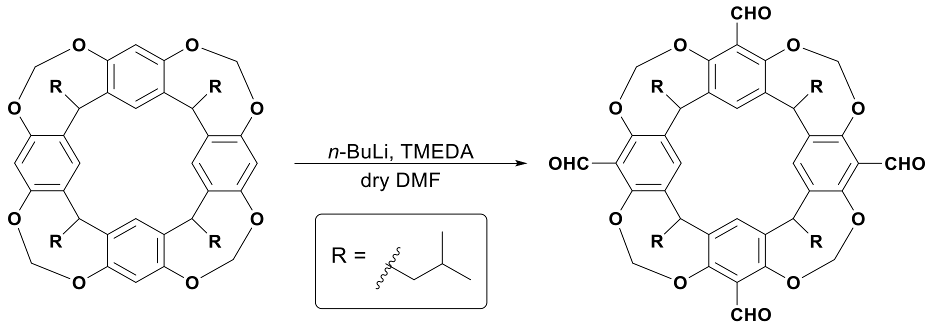

The synthesis of compound **3** was adapted from literature.[5] The reaction was carried out using standard Schlenk technique under argon. Compound **S3** (0.502 g, 0.657 mmol) and TMEDA (1.14 ml, 7.62 mmol) were dissolved in anhydrous THF (20 ml). *n*-BuLi (3.15 ml, 7.88 mmol) was added dropwise at 0 °C. After 1h, anhydrous DMF (0.76 ml, 9.85 mmol) was added. The reaction was allowed to warm to room temperature and left for 3 days with stirring. Then 1 M HCl (2.05 ml) was added and the mixture was extracted with ethyl acetate and washed with NaCl. The combined organic phases were dried over Na_2SO_4 and the solvents were evaporated in vacuo. The residue was washed with diethyl ether. The product **3** was obtained as white solid with 61% yield (352 mg).

The analytical data are in agreement with the literature.[5]
^{1}H NMR (400 MHz, 298 K, $CDCl_3$) δ [ppm]) 10.25 (s, 4H), 7.29 (s, 4H), 5.91 (d, J = 7.5 Hz, 4H), 5.03 (t, J = 8.2 Hz, 4H), 4.47 (d, J = 7.5 Hz, 4H), 2.10 (t, J = 7.9 Hz, 8H), 1.56 (p, J = 6.7 Hz, 4H), 1.04 (d, J = 6.6 Hz, 24H).

2.3 Synthesis of receptors 5 and 6

Synthesis of compound **S4**

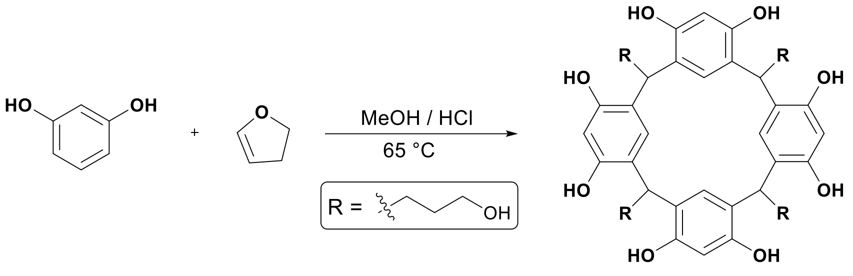

Resorcinol (10.00 g, 91 mmol) was dissolved in 60 ml of 4:1 (*v*:*v*) methanol:37% HCl under argon. 2,3-Dihydrofuran (7.1 ml, 91 mmol) was added via syringe pump over 4 h. The reaction mixture was stirred for 4 h at rt, and then was heated to 50 °C. After 7 days a considerable amount of precipitate was observed and the reaction mixture was allowed to cool to rt. The solid was filtered off and taken up in 300 ml of cold distilled water and sonicated[6]. The solid was again filtered off and dried overnight under vacuum to give 12.03 g of **S4** at 74% yield.

The analytical data are in agreement with the literature.[6]

^{1}H NMR (400 MHz, 298 K, DMSO-d_6) δ [ppm] 8.88 (s, 8H), 7.20 (s, 4H), 6.12 (s, 4H), 4.17 (t, J = 7.9 Hz, 4H), 3.39 (under water peak, m, 8H) 2.07 (m, J = 7.7 Hz, 8H), 1.31 (m, J = 6.9 Hz, 8H).

[6] Gibb, B. C.; Chapman, R. G.; Sherman, J. C. Synthesis of hydroxyl-footed cavitands. *J. Org. Chem.* **1996**, *61*, 1505–1509.

Synthesis of compound **S5**

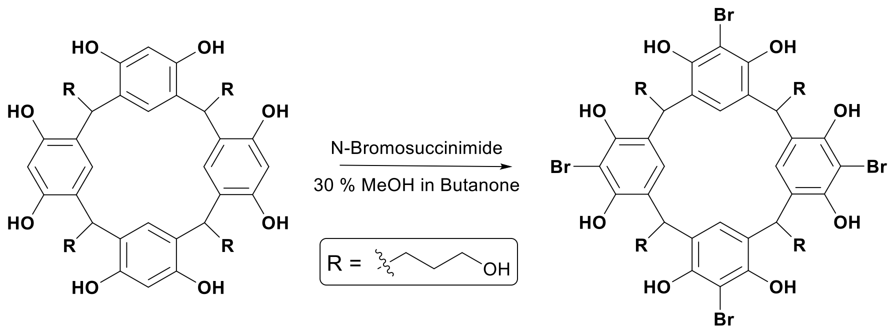

S4 **S5**

Compound **S4** (4.100 g, 5.5 mmol) was dissolved in a mixture of methanol:butanone (30:70, *v*:*v*, 80 ml). *N*-Bromosuccinimide (NBS, 5.200 g, 28 mmol) was added to this suspension, and the reaction mixture was stirred at rt in the dark for 5 h. Then, additional portion of NBS (2.100 g, 11 mmol) was added, and the reaction mixture was stirred overnight. The reaction mixture was filtered, and the solid was washed with 50 ml of cold butanone[6]. The solid was dried to give 5.002 g (87%) of **S5**.

The analytical data are in agreement with the literature.[6]

^{1}H NMR (400 MHz, 298 K, DMSO-d_6) δ [ppm] 9.06 (s, 8H), 7.38 (s, 4H), 4.32 (t, J = 7.9 Hz, 4H), 3.41 (t, J = 6.6 Hz, 8H), 2.20 (m, J = 7.8 Hz, 8H), 1.32 (m, J = 6.9 Hz, 8H).

Synthesis of receptor **5**

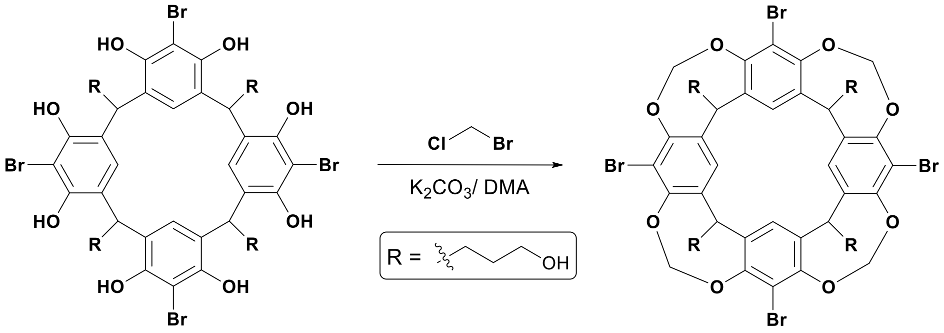

S5 5

Compound **S5** (2.000 g, 2 mmol) was dissolved in anhydrous DMA (10 ml) and added via syringe pump over 2 days to a stirred mixture of bromochloromethane (0.62 ml, 9.6 mmol) and potassium carbonate (4.200 g, 29 mmol) in anhydrous DMA (20 ml). The reaction mixture was stirred for another day at rt, and further bromochloromethane (0.62 ml, 9.6 mmol) was added. After this, the reaction mixture was stirred at 45 °C for 1 day. Bromochloromethane (0.62 ml, 9.6 mmol) was added again, and the reaction mixture was stirred at 65 °C for additional 3 days. After the reaction mixture was cooled to rt, the solvent was removed *in vacuo*. The solid residue was subjected to sonication in 50 ml of water followed by slow addition of 2 M HCl to neutralize the carbonate salts. The resulting mixture was filtered, and the solid was again washed with water (3 × 30 ml) and dried overnight under vacuum[6]. The crude was purified by flash chromatography [$CHCl_3$:methanol, 9:1 (*v*:*v*)] to give 0.820 g of the desired product **5** in 50% yield.

The analytical data are in agreement with the literature.[6]

^{1}H NMR (400 MHz, 298 K, DMSO-d_6) δ [ppm] 7.64 (s, 4H), 5.97 (d, J = 7.6 Hz, 4H), 4.69 (t, J = 8.1 Hz, 4H), 4.48 (broad, 4H), 4.29 (d, J = 7.6 Hz, 4H), 3.49 (m, 8H), 2.45 – 2.35 (m, 8H), 1.44 (m, J = 6.7 Hz, 8H).

Synthesis of receptor **6**

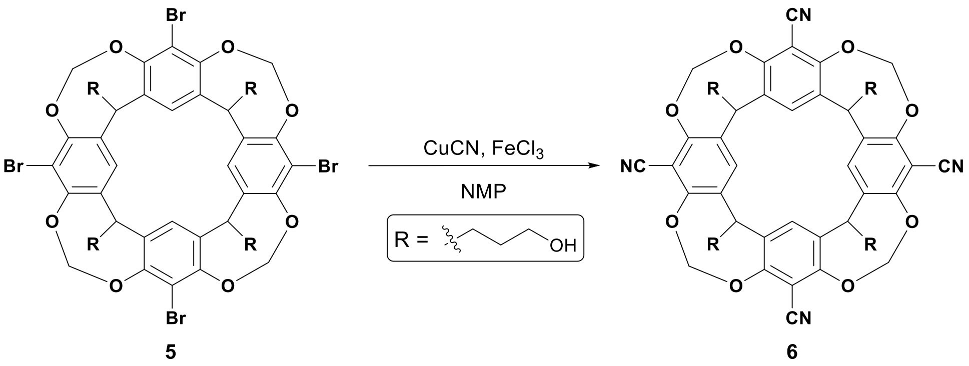

The synthesis of compound **6** is similar to the synthesis of compound **2**. To a solution of **5** (255 mg, 0.235 mmol) in *N*-methyl pyrrolidinone (NMP) (10 ml), CuCN was added (210 mg, 2.35 mmol). The mixture was stirred at reflux for 24h. After the reaction is allowed to cool down to 80°C, anhydrous $FeCl_3$ (610 mg, 3.76 mmol) was added and the solution was stirred for additional 6h. After the mixture was allowed to cool down to room temperature, the precipitate was filtered off, washed multiple times with water, and dried under high vacuum to give a dark brown coloured solid. The solid was subjected to flash chromatography [$CHCl_3$: methanol 9:1 (v/v)] to give **6** as a beige solid in 59% yield (120 mg).

^{1}H NMR (500 MHz, 298 K, THF-d_8) δ [ppm] 7.95 (s, 4H), 6.06 (d, J = 7.5 Hz, 4H), 4.79 (t, J = 8.3 Hz, 4H), 4.53 (d, J = 7.4 Hz, 4H), 4.38 (broad, 4H), 3.66 (m, J = 5.6 Hz, 8H), 2.56 (under water peak, m, 8H), 1.53 (m, J = 6.8 Hz, 8H).

^{13}C NMR (126 MHz, 298 K, THF-d_8) δ [ppm] 155.9, 139.2, 127.0, 111.6, 103.9, 98.8, 61.2, 36.7, 30.9, 25.7.

HR-MS: *m*/*z* $[M+H]^+$ calcd. for $C_{48}H_{45}N_4O_{12}$: 869.3034, found: 869.3021.

2.4 Synthesis of receptor 7

Synthesis of compound **S6**

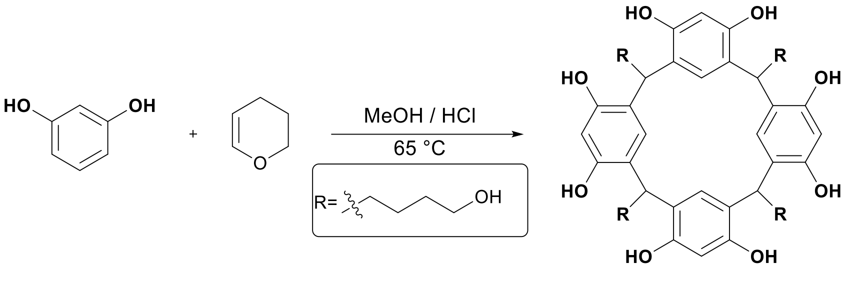

S6

Resorcinol (10.000 g, 55 mmol) was dissolved in 60 ml of 4:1 (*v*:*v*) methanol/37% HCl under argon. 3,4-Dihydro-2*H*-pyran (5.00 ml, 55 mmol) was then added via syringe pump over 4 h. The mixture was stirred at rt 4 h and then further, heated to 50 °C. After 4-5 days, when the formation of a significant amount of precipitate was visible, the reaction was stopped and the reaction mixture was allowed to cool to rt. The solid was filtered off and then taken up in 200 ml of cold distilled water and sonicated[6]. The solid was again filtered off and dried overnight to give **S6** in 23% yield (4.020 g) as yellowish orange solid.

The analytical data are in agreement with the literature.[6]

^{1}H NMR (400 MHz, 298 K, DMSO-d_6) δ [ppm] 8.89 (s, 8H), 7.22 (s, 4H), 6.12 (s, 4H), 4.17 (t, J = 7.7 Hz, 4H), 3.76 (broad, 4H), 3.33 (t, J = 6.7 Hz, 8H), 2.08 (m, 8H), 1.45 (m, 8H), 1.25 (m, 8H).

Synthesis of compound **S7**

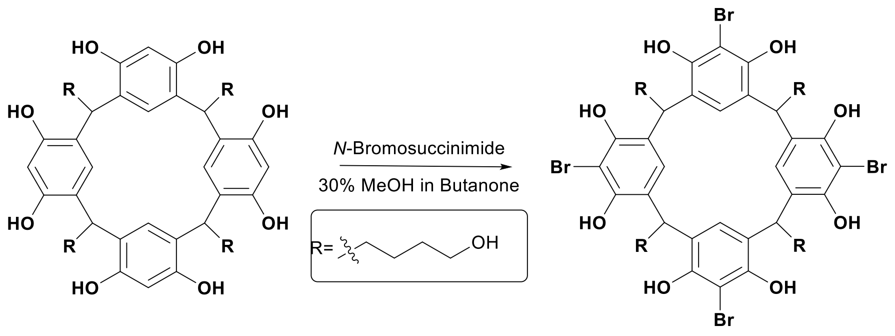

Compound **S6** (4.100 g, 5.1 mmol) was added to 60 ml of 30% methanol in butanone. *N*-Bromosuccinimide (NBS, 4.500 g, 25 mmol) was added and the reaction mixture was stirred at rt in the dark for 5 h. Additional NBS (1.830 g, 10.2 mmol) was added, and the reaction mixture was stirred overnight. The reaction mixture was filtered, and the solid was washed with 50 ml of cold butanone[6]. The solid was dried to give 2.020 g of **S7** in 36% yield.

The analytical data are in agreement with the literature.[6]

^{1}H NMR (400 MHz, 298 K, DMSO-d_6) δ [ppm] 9.12 (s, 8H), 7.40 (s, 4H), 4.31 (t, J = 7.8 Hz, 4H), 3.34 (t, J = 6.6 Hz, 9H), 2.20 (m, 8H), 1.46 (m, 8H), 1.23 (m, 8H).

Synthesis of receptor **7**

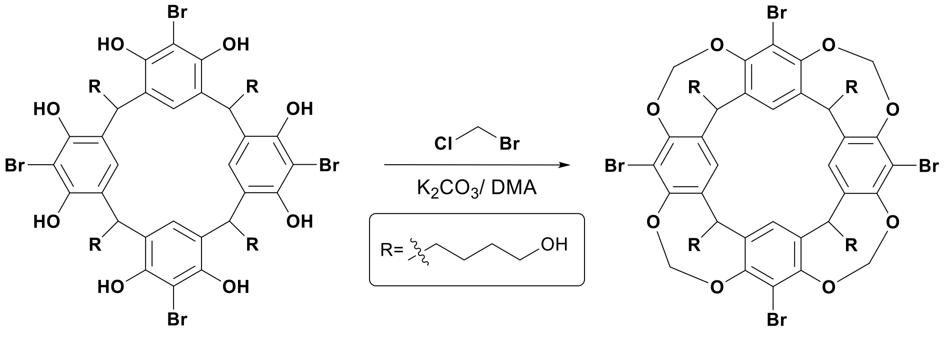

Compound **S7** (1.820 g, 1.64 mmol) was dissolved in 30 ml of anhydrous DMA and added via syringe pump over 2 days to a stirred mixture of bromochloromethane (0.53 ml, 8.3 mmol) and potassium carbonate (3.410 g, 24.7 mmol) in anhydrous DMA (20 ml). The reaction mixture was stirred for another day at rt, and further bromochloromethane (0.53 ml, 8.31 mmol) was added. After this, the reaction mixture was stirred at 45 °C for 1 day. Bromochloromethane (0.53 ml, 8.3 mmol) was added again, and the reaction mixture was stirred at 65 °C for additional 3 days. After the reaction mixture was cooled to rt, the solvent was removed *in vacuo*. The solid residue was subjected to sonication in 50 ml of water followed by slow addition of 2 M HCl to neutralize the carbonate salts. The resulting mixture was filtered, and the solid was again washed with water (3 × 30 ml) and dried overnight under vacuum[6]. The crude was purified by flash chromatography [$CHCl_3$: methanol 9:1 (*v*/*v*)], to give 0.920 g of compound **7** in 48% yield.

The analytical data are in agreement with the literature followed.[6]

^{1}H NMR (400 MHz, 298 K, DMSO-*d*$_6$) *δ* [ppm] δ 7.63 (s, 4H), 5.98 (d, *J* = 7.6 Hz, 6H), 4.67 (t, *J* = 8.1 Hz, 6H), 4.39 (broad, 4H), 4.28 (d, *J* = 7.6 Hz, 4H), 3.38 (t, *J* = 6.4 Hz, 8H), 2.45 – 2.34 (m, 8H), 1.51 (m, 8H), 1.30 (m, 8H).

3. NMR SPECTRA OF RECEPTORS

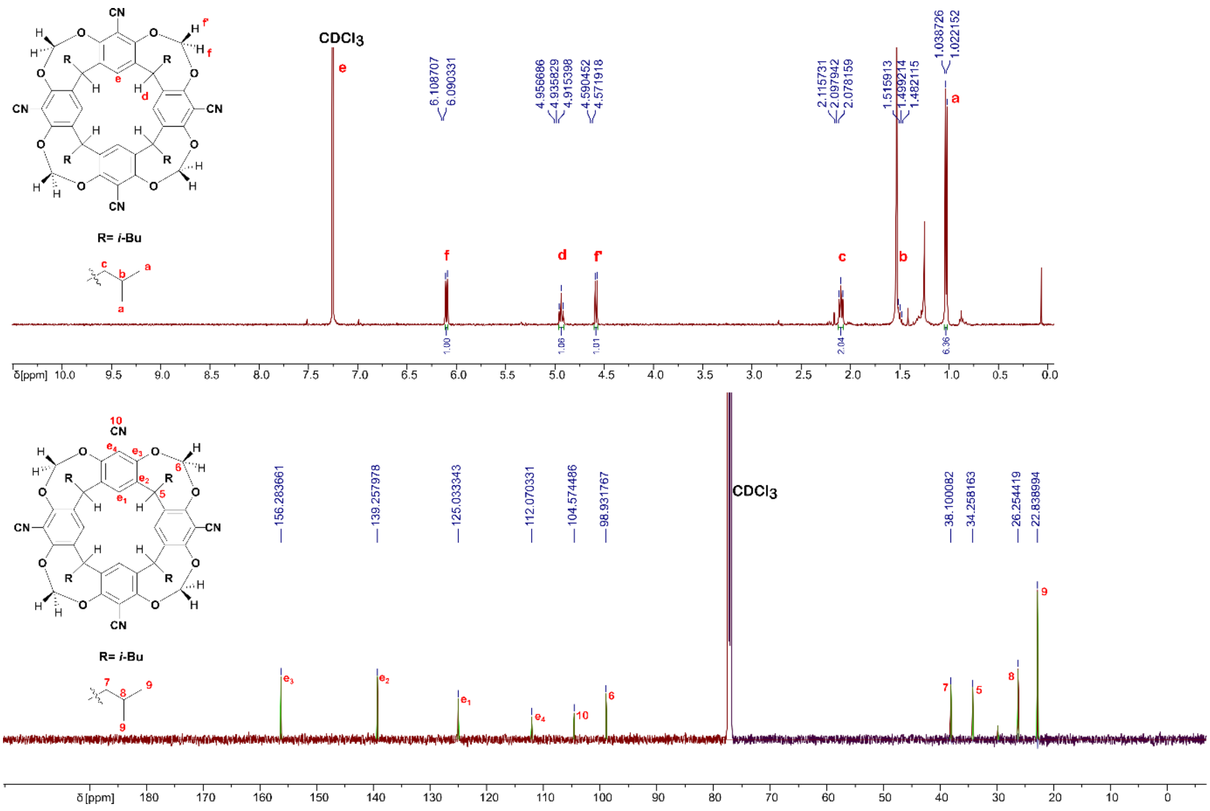

Figure S2. ^{1}H NMR (400 MHz, 298 K, $CDCl_3$) and ^{13}C NMR (151 MHz, 298 K, $CDCl_3$) spectra of receptor **4**.

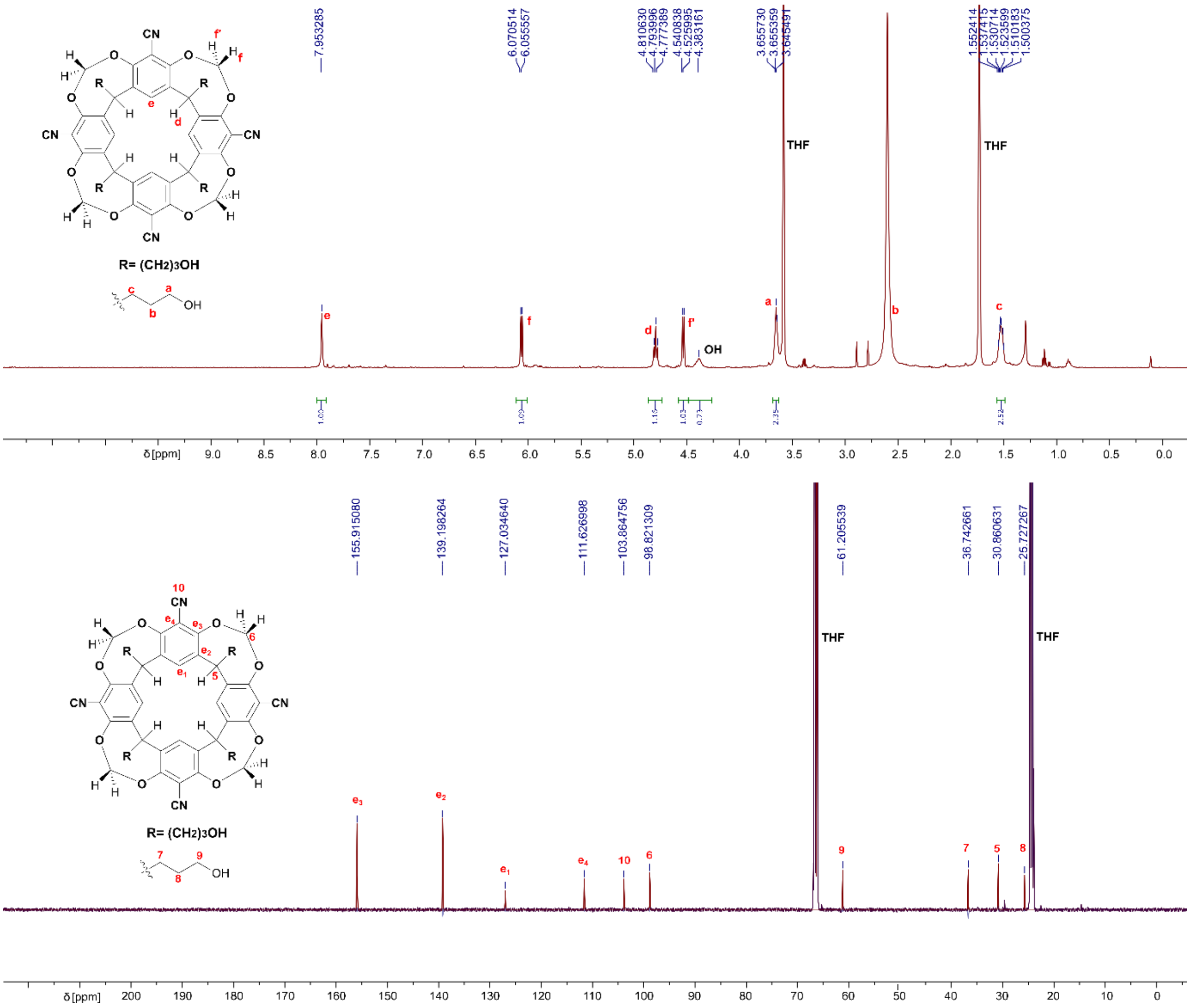

Figure S3. ^{1}H NMR (500 MHz, 298 K, THF-d_8) and ^{13}C NMR (126 MHz, 298 K, THF-d_8) spectra of receptor **6**.

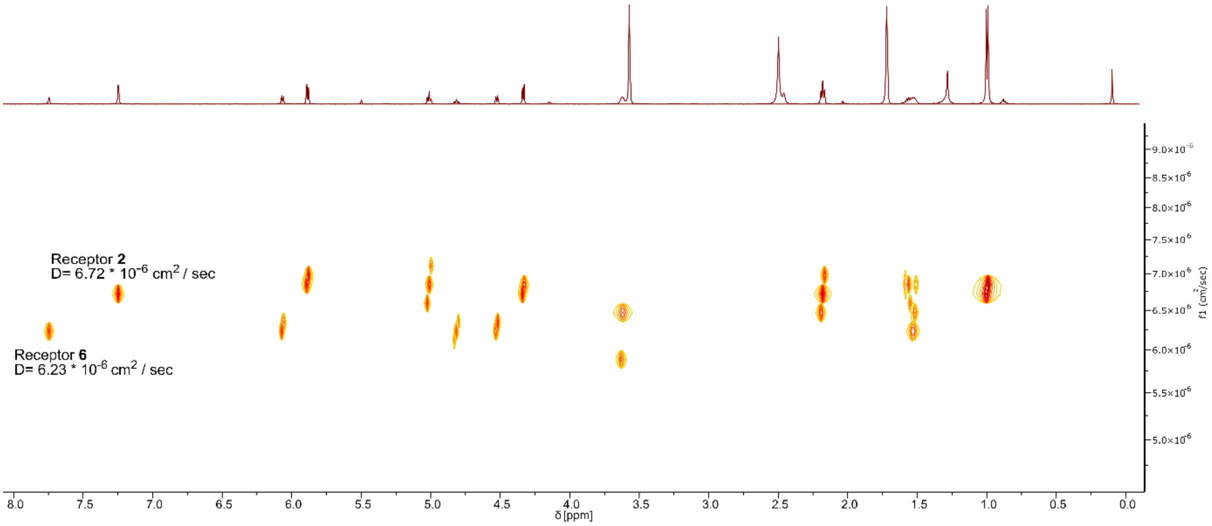

Figure S4. DOSY spectrum of a mixture of **2** and **6** (500 MHz, 298 K, THF-d_8).

4. TITRATIONS

General procedures for titrations

^{1}H NMR titrations:

All the reagents were weighted separately on an Ohaus Discovery analytical balance (readability 0.01 mg). A 0.0067 M solution of the receptor was prepared by dissolution of the receptor (0.01 mmol) in 1.5 ml of the desired solvent (THF-d_8 or $CDCl_3$). From this stock, 0.5 ml of the solution was transferred to the NMR tube as a titrant (analyte). The titrant solutions were prepared by dissolving 0.1 mmol (A) or 0.02 mmol (B) of the required ammonium salt in 1 ml of the stock receptors solution (in order to avoid dilution of the receptor during the experiment). Thus, the titrant solutions containing A (0.0067 M receptor and 0.1 M salt) or B (0.0067 M receptor and 0.02 M salt) were prepared.

The titration was performed by adding aliquots of the titrant solution (10 - 200 μl) to the reference sample and recording ^{1}H NMR spectra after each addition. ^{1}H NMR spectra were recorded at 298 K or 255 K using Bruker 400 MHz.

^{1}H NMR competitive titrations:

All the reagents were weighted separately on an Ohaus Discovery analytical balance (readability 0.01 mg). A solution containing receptor **2** and receptor **5** (0.0067 M each) was prepared by dissolution of the **1** (0.01 mmol) and **5** (0.01 mmol) in 1.5 ml of the desired solvent (THF-d_8). From this stock, 0.5 ml of the solution was transferred to the NMR tube as a titrant (analyte). The titrant solution was prepared by dissolving 0.02 mmol of the required ammonium salt in 1 ml of the stock solution (in order to avoid dilution of the receptors during the experiment). Thus, the titrant solution containing **2**, **5**, and salt (0.0067 M of **2**, 0.0067 M of **5**, and 0.02 M of salt) was prepared. The similar procedure was also followed for the competitive titration of receptor **5** and **6**.

The titration was performed by adding aliquots of the titrant solution (10-100 µl) to the reference sample and recording ^1H NMR spectra after each addition at 255 K using Bruker 400 MHz.

UV-Vis titrations:

Receptor **3**: To the solution of the receptor (C = 3.66 $\times 10^{-5}$ M, prepared by dissolution of 0.00073 mmol of the host in 20 ml of THF), a solution containing a guest (C = 0.00073 M, 0.00146 mmol) and the host (C = 3.66 $\times 10^{-5}$ M, 0.00073 mmol) in THF was added in portions (20-400 µl). Titrants were prepared by dissolving the required ammonium salt in the solution of the receptor, in order to avoid dilution of the receptor during titration.

Receptor **4**: To the solution of the receptor (C = 4.36$\times 10^{-5}$ M, prepared by dissolution of 0.00087 mmol of the host in 20 ml of THF), a solution containing a guest (C = 0.00087 M, 0.00174 mmol) and the host (C = 4.36$\times 10^{-5}$ M, 0.00087 mmol) in THF was added in portions (20-400 µl). Titrants were prepared by dissolving the required ammonium salt in the solution of the receptor, in order to avoid dilution of the receptor during titration.

To the solution of the receptor (C = 3.48 $\times 10^{-5}$ M, prepared by dissolution of 0.00069 mmol of the host in 20 ml of DCM), a solution containing a guest (C = 0.00069 M, 0.00139 mmol) and the host (C = 3.48 $\times 10^{-5}$ M, 0.00069 mmol) in DCM was added in portions (20-400 µl). Titrants were prepared by dissolving the required ammonium salt in the solution of the receptor, in order to avoid dilution of the receptor during titration.

UV spectra were recorded at room temperature on U-1900 Spectrophotometer (UV/Vis). All UV spectra and experiments were repeated thrice. The solvents for UV-Vis titrations, purified using a solvent purification system (SPS) was used.

NMR titration of 1 with But_4NHSO_4 in THF-d_8/10% D_2O

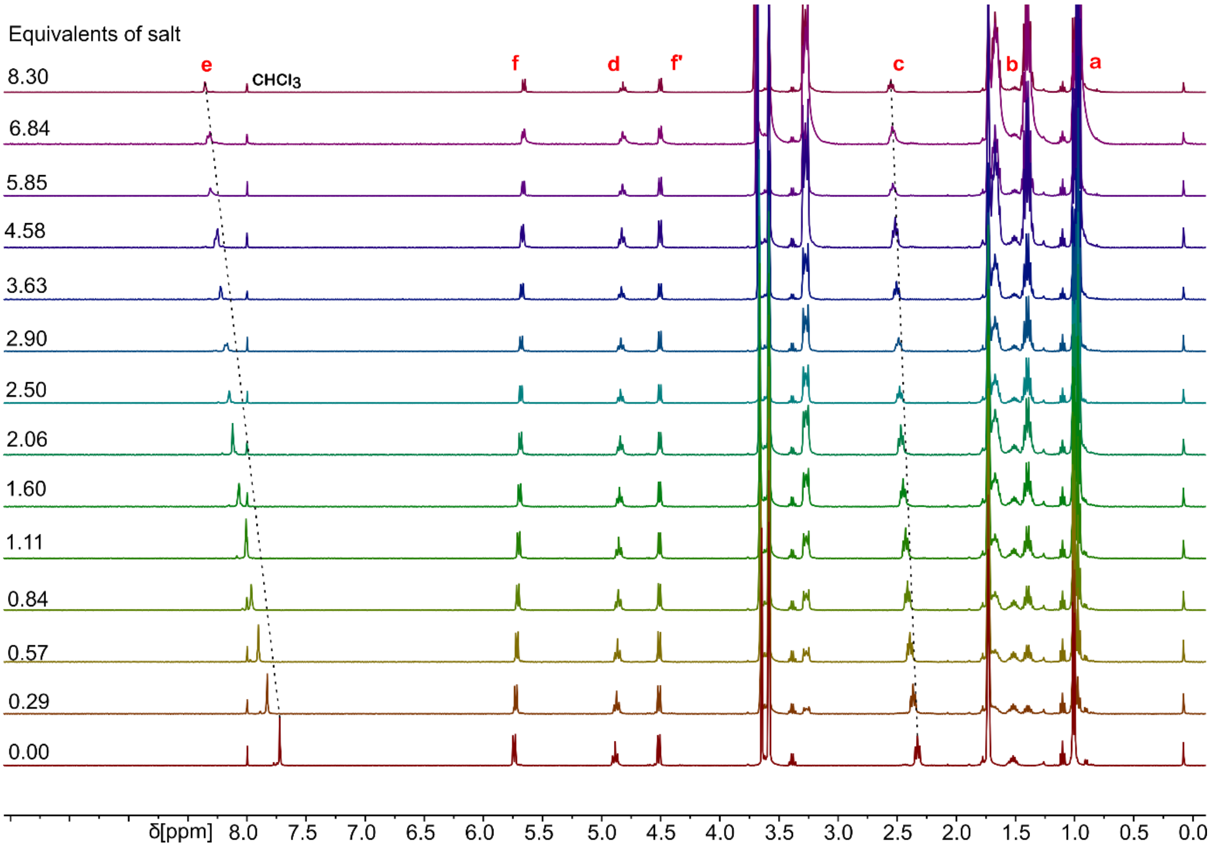

Figure S5. ^{1}H NMR spectra for the titration of **1** (0.0067 M) with **But_4NHSO_4** in THF-d_8/10% D_2O at 298 K.

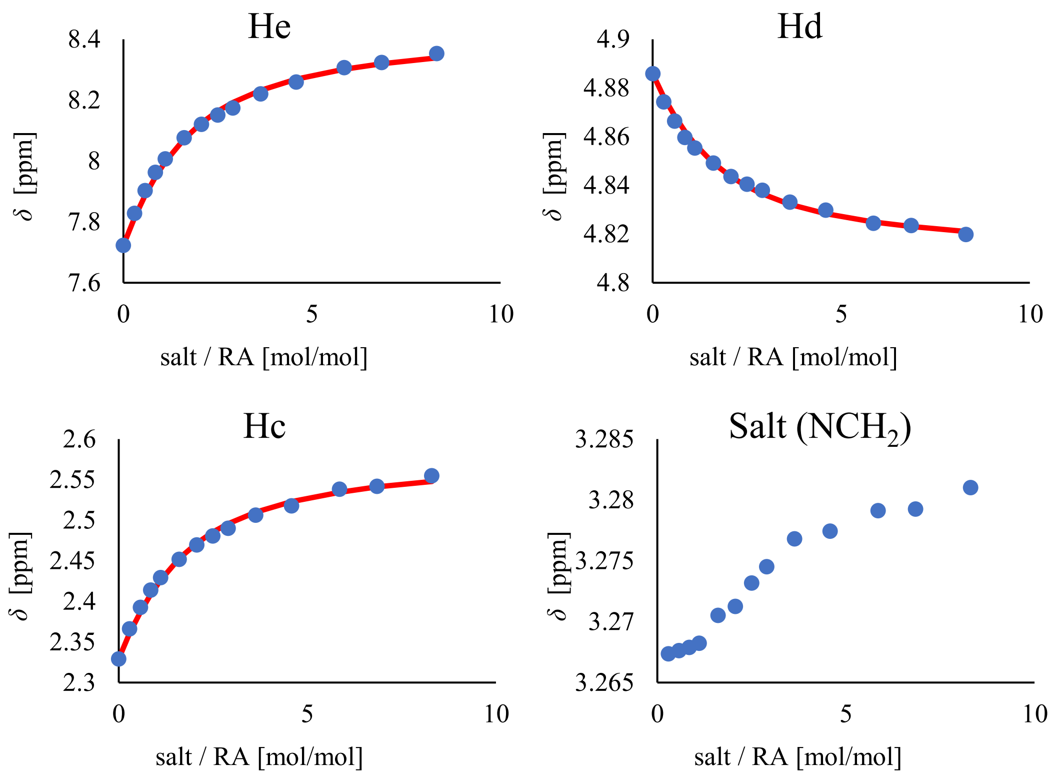

K (M^{-1})	K error (%)	SSR	Datapoints fitted	Params fitted
129.293199	3.35470521	0.00212926	42	4

http://app.supramolecular.org/bindfit/view/219accb9-46fe-4806-b0d0-2f2ecb2045fb

Figure S6. ^{1}H NMR experimental points and fitted curves for the titration of **1 (RA)** (0.0067 M) with **But_4NHSO_4 (salt)**. ^{1}H NMR chemical shift changes for: (a) H_e; (b) H_d (c) H_c and (d) NCH_2 of the salt (298 K, THF-d_8/10% D_2O). Red lines correspond to fitted curves.

NMR titration of 2 with Pen_4NCl in THF-d_8/ 10% D_2O

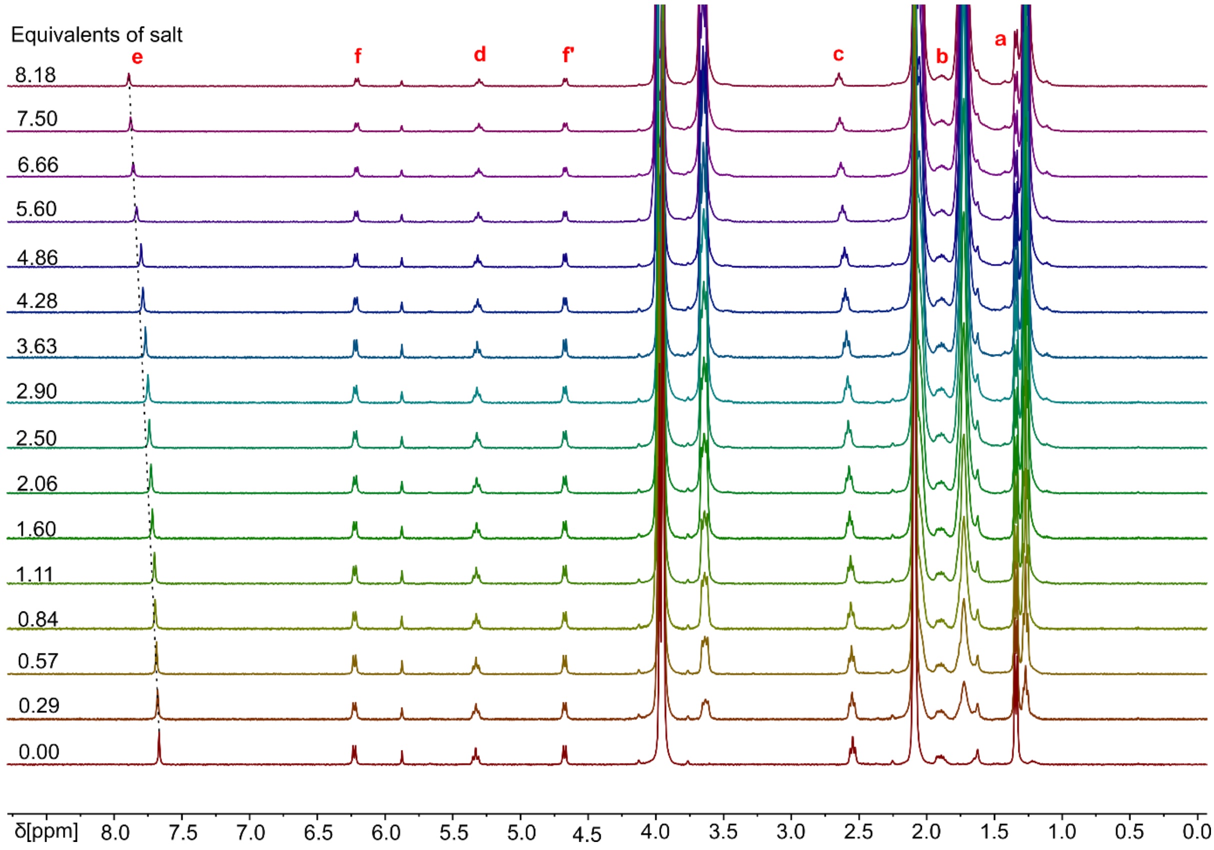

Figure S7. ^{1}H NMR spectra for the titration of **2** (0.0067 M) with **Pen_4NCl** in THF-d_8/ 10%D_2O at 298 K.

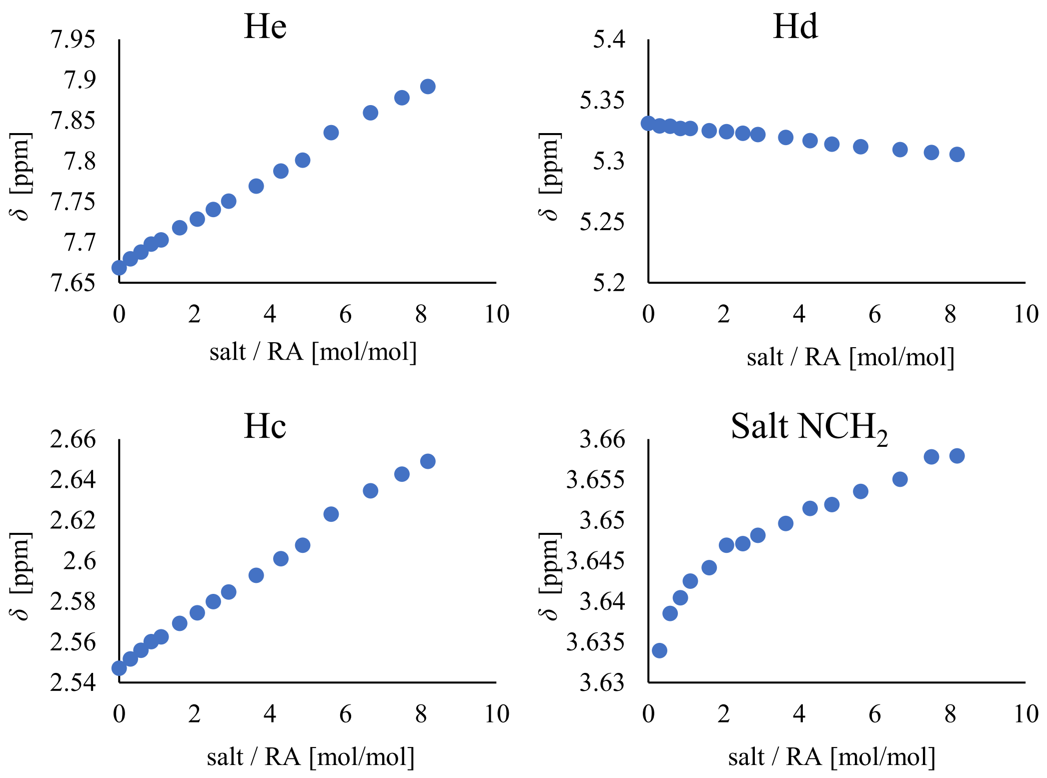

Figure S8. ^{1}H NMR experimental points for the titration of **2 (RA)** (0.0067 M) with **Pen$_4$NCl (salt)**. ^{1}H NMR chemical shifts' changes for: (a) H_e; (b) H_d (c) H_c and (d) NCH_2 of the salt (298 K, THF-d_8/10% D_2O). The data could not be fitted due to negligible binding.

NMR titration of 2 with But_4NHSO_4 in THF-d_8/ 10%D_2O

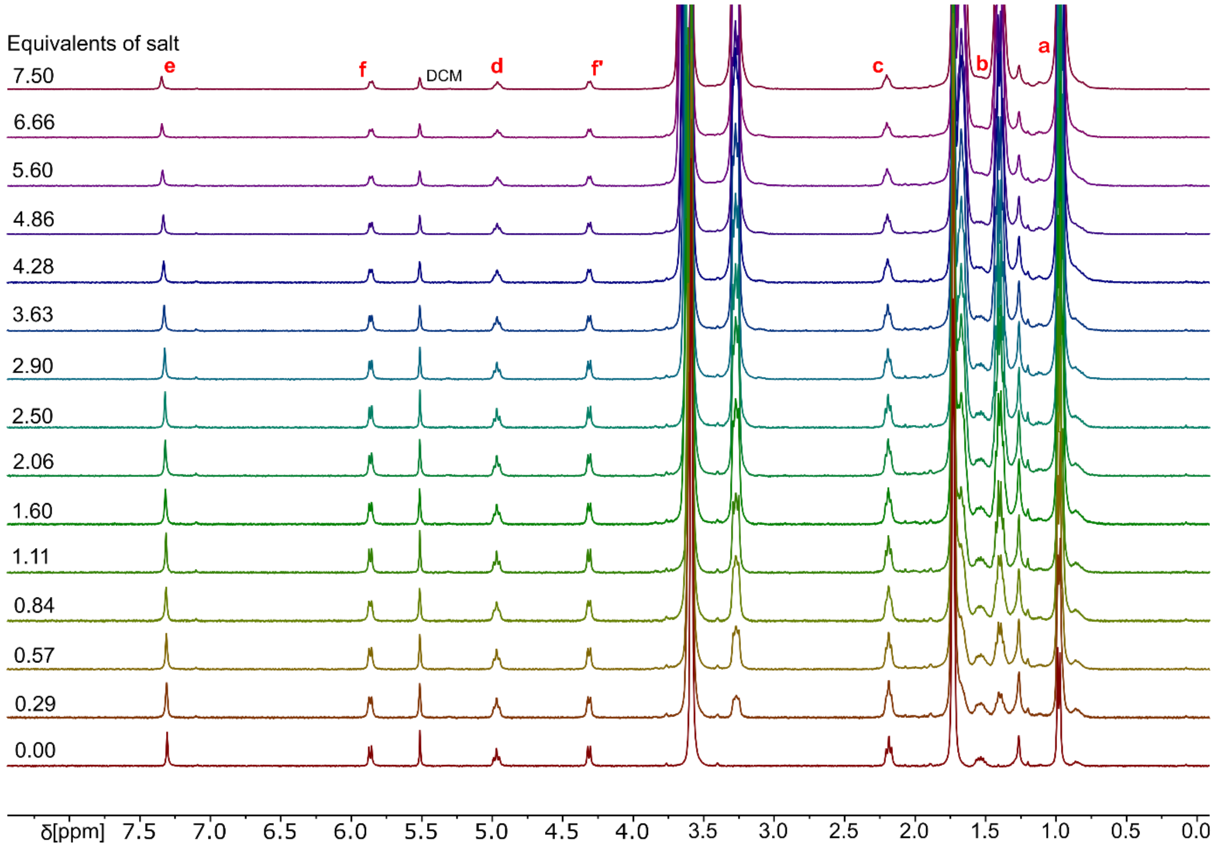

Figure S9. ^{1}H NMR spectra for the titration of **2** (0.0067 M) with **But_4NHSO_4** in THF-d_8/10% D_2O at 298 K.

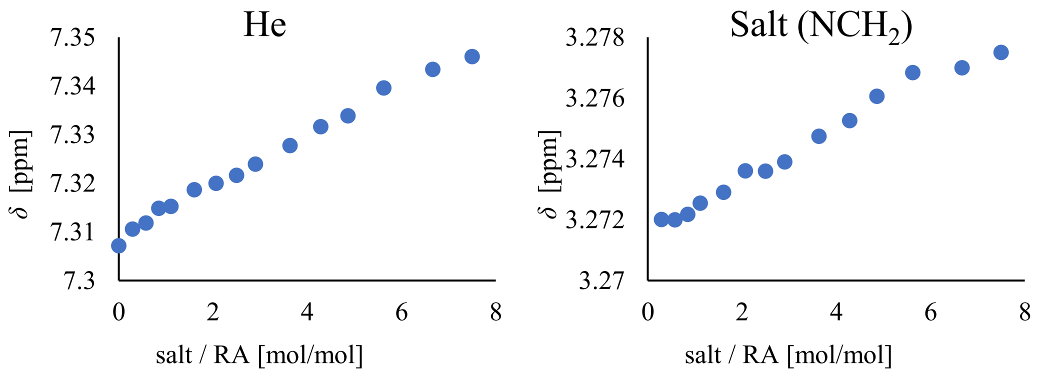

Figure S10. ^{1}H NMR experimental points for the titration of **2 (RA)** (0.0067 M) **with But_4NHSO_4 (salt)**.^{1}H NMR chemical shifts' changes for: (a) H_e; (b) NCH_2 of the salt (298 K, THF-d_8/10%D_2O). The data could not be fitted due to negligible binding.

UV-vis titration of 3 with Pen_4NCl in THF

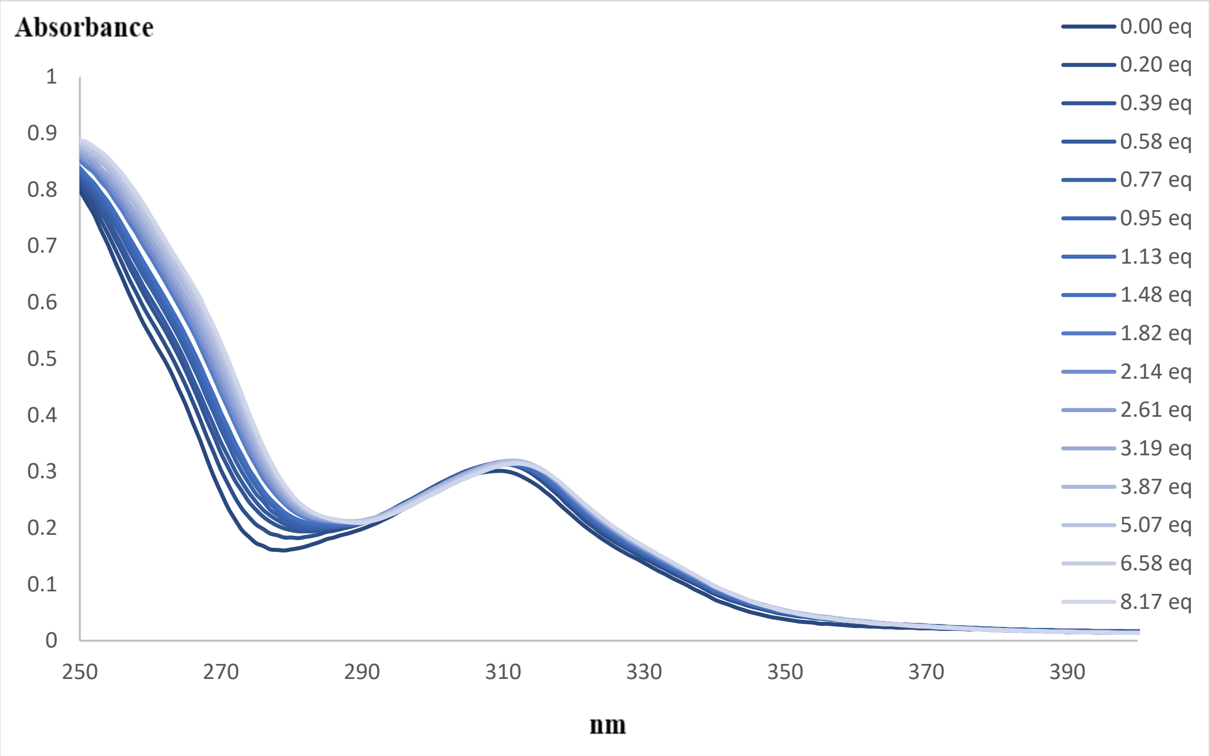

Figure S11. UV-vis spectra for titration of **3** (3.66 $\times 10^{-5}$ M) with **Pen_4NCl** in THF at 298 K.

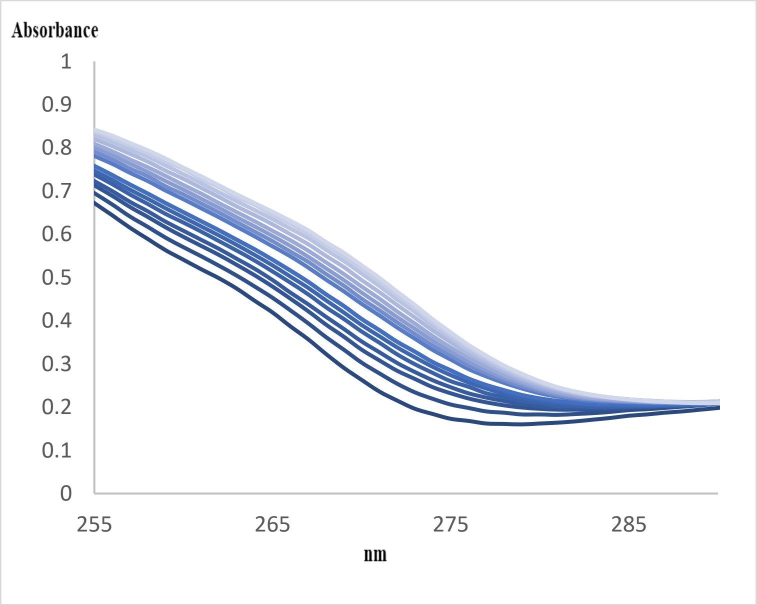

Figure S12. Zoomed UV-vis spectra for titration of **3** (3.66 $\times 10^{-5}$ M) with **Pen_4NCl** in THF at 298 K.

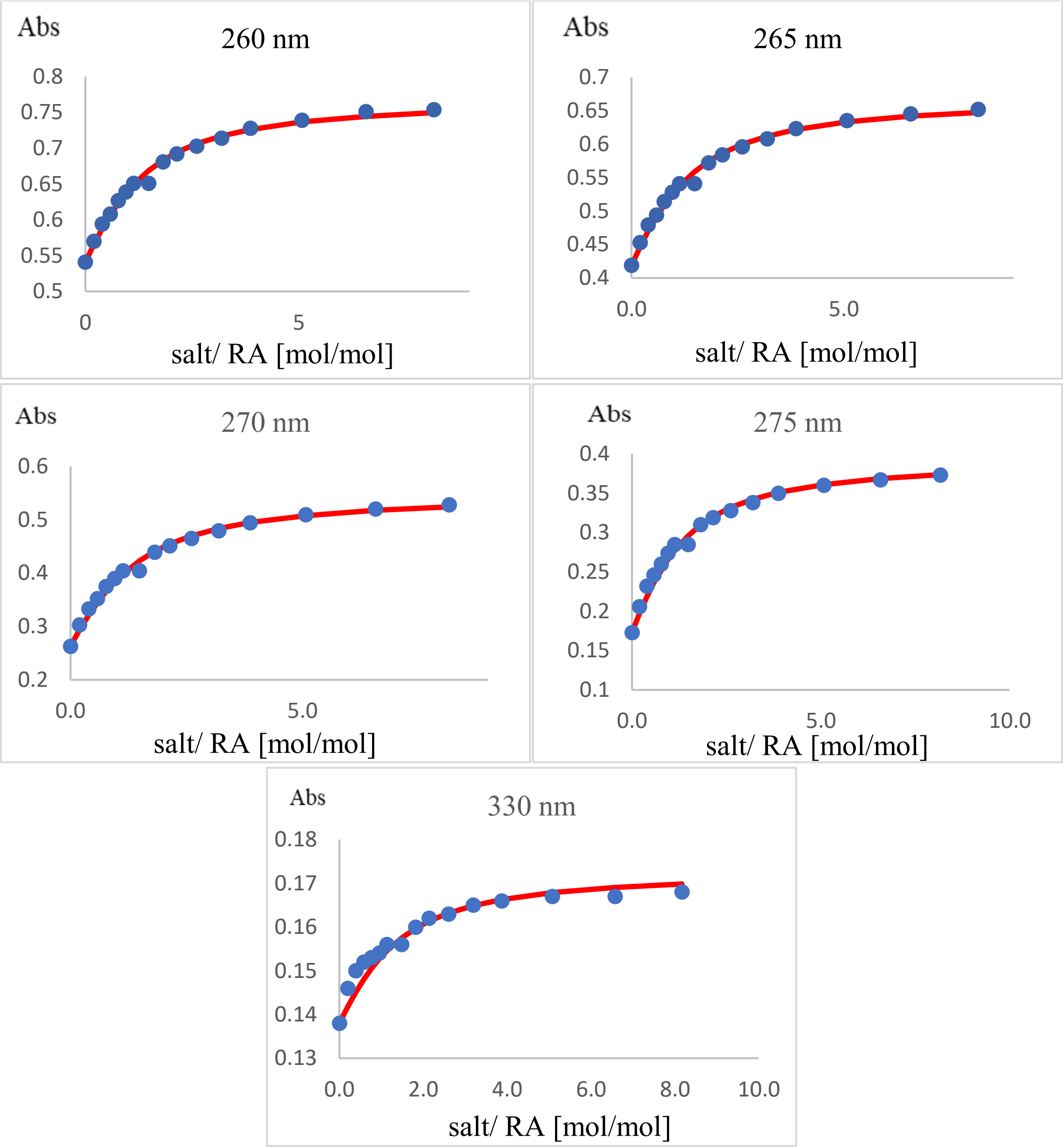

**K(M^{-1})**	**K error (%)**	**SSR**	**Datapoints fitted**	**Params fitted**
36810.59	± 3.9398	2.9125e-3	96	7

http://app.supramolecular.org/bindfit/view/89dc7b82-7739-4dbd-8d77-16123fbc3cb6

Figure S13. Experimental points and fitted curves for UV-vis titration of **3 (RA)** (3.66 ×10^{-5} M) with **Pen$_4$NCl (salt)** in THF at 298 K. Red lines correspond to fitted curves.

NMR titration of 3 with Pen_4NCl in THF-d_8

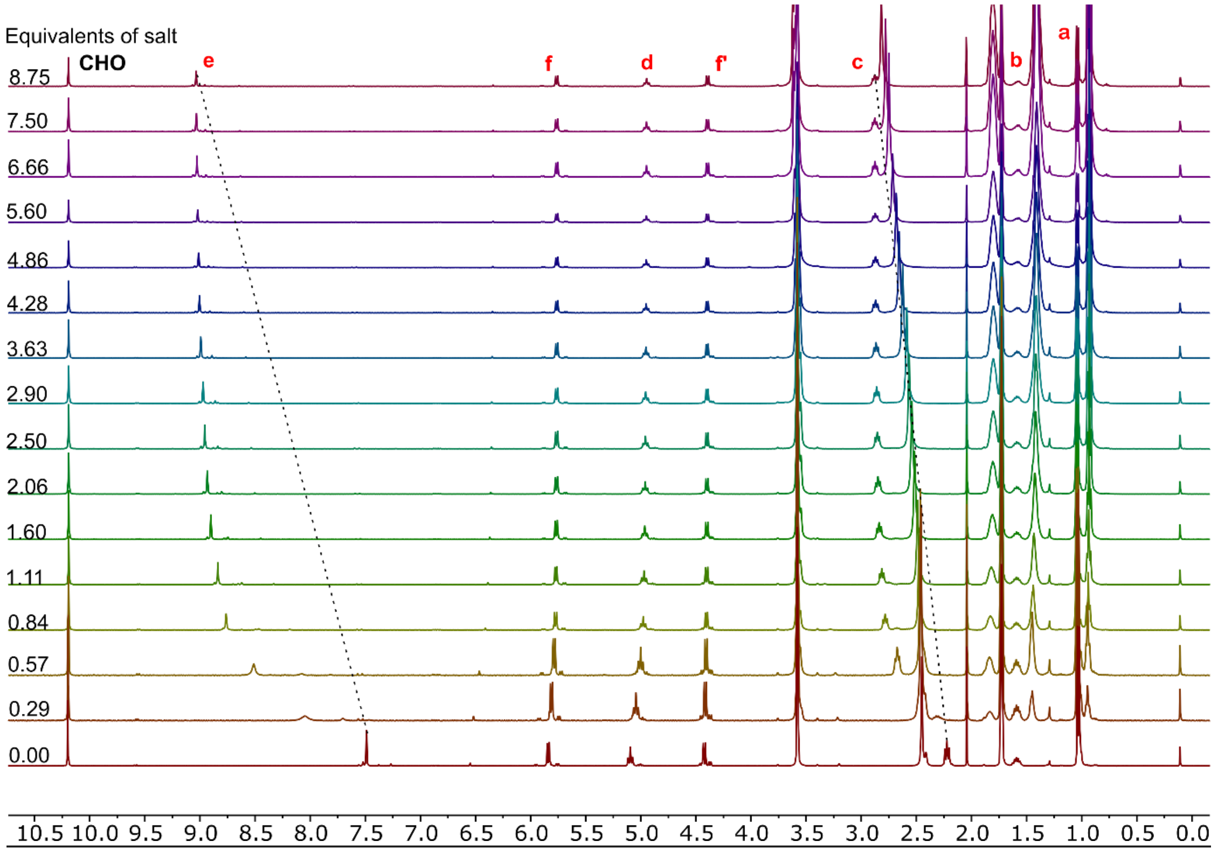

Figure S14. ^{1}H NMR spectra for the titration of **3** (0.0067 M) with **Pen_4NCl** in THF-d_8 at 298 K.

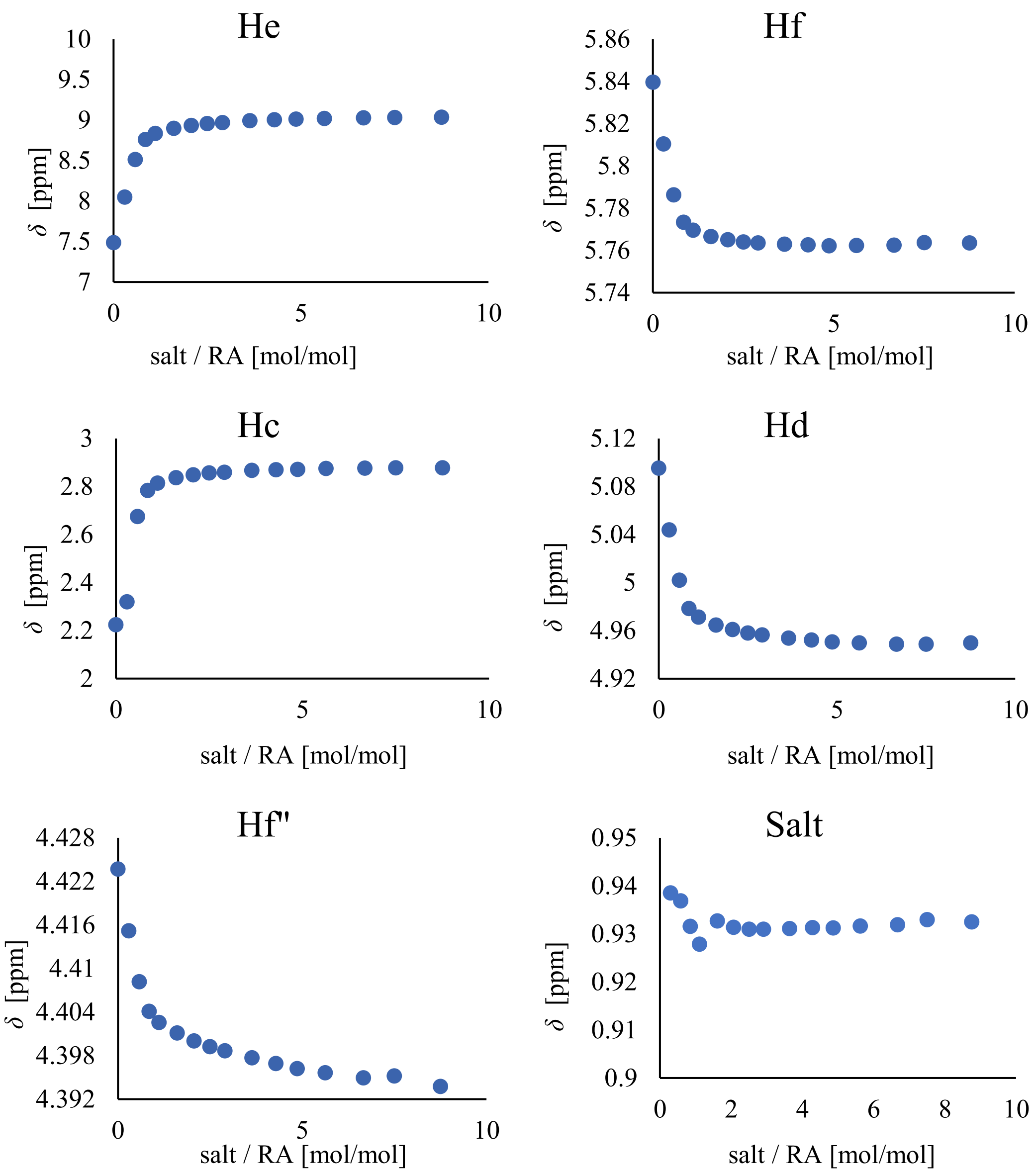

K (M^{-1})	K error (%)	SSR	Datapoints fitted	Params fitted
12192.33	± 62.7867	0.0868	80	6

http://app.supramolecular.org/bindfit/view/ac42de11-1295-4790-b97e-20e3abd000ba

Figure S15.^{1}H NMR experimental points for the titration of **3 (RA)** (0.0067 M) with **Pen_4NCl (salt)**. ^{1}H NMR chemical shifts' changes for: (a) H_e; (b) H_f; (c) H_c; (d) H_d; (e) $H_{f'}$; and (f) CH_3 of the salt (298 K, THF-d_8). The data could not be fitted reasonably due to high error.

NMR titration of 3 with But_4NPhCO_2 in THF-d_8

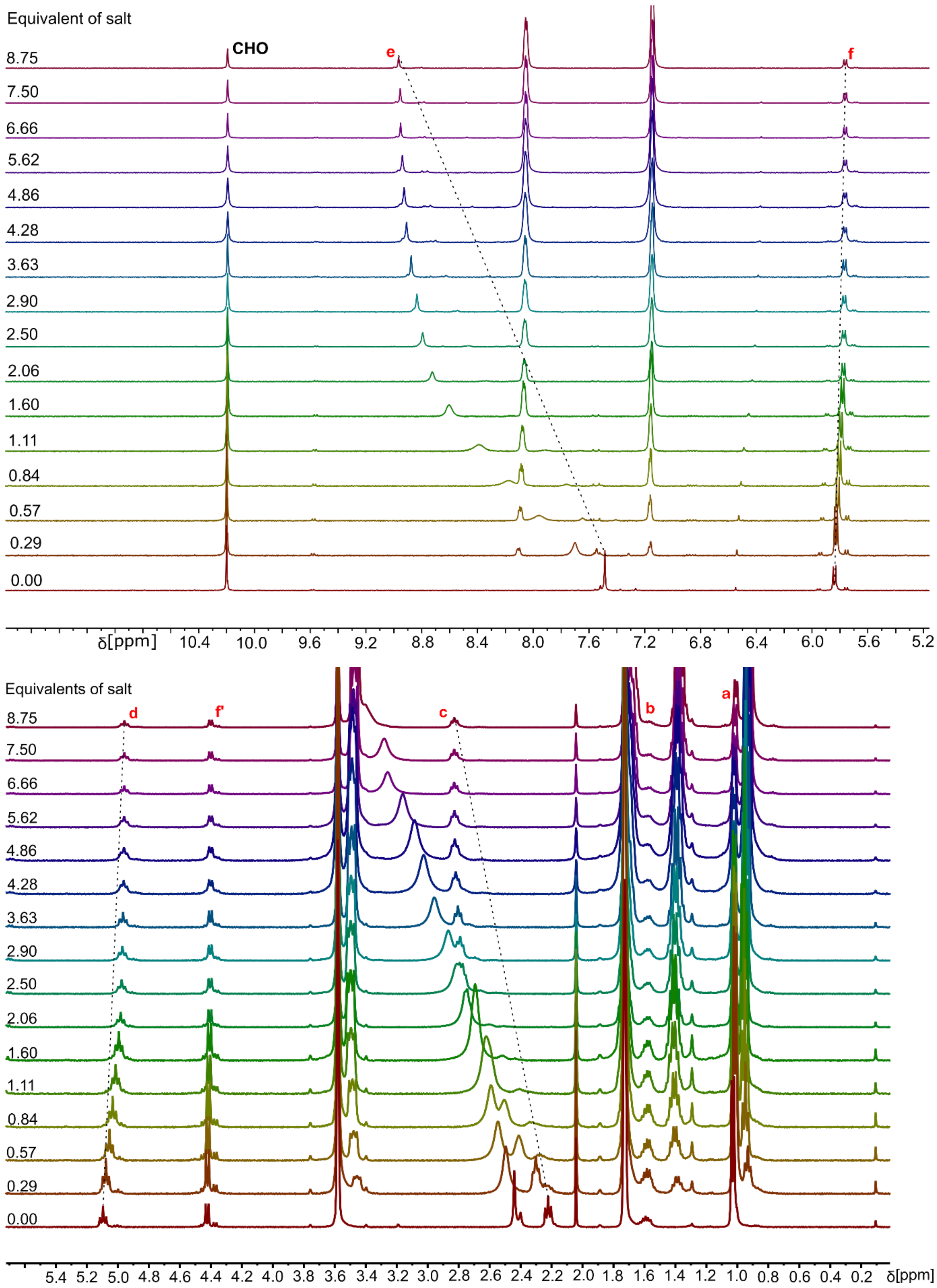

Figure S16. ^1H NMR spectra for the titration of **3** with **But_4NPhCO_2** in THF-d_8.

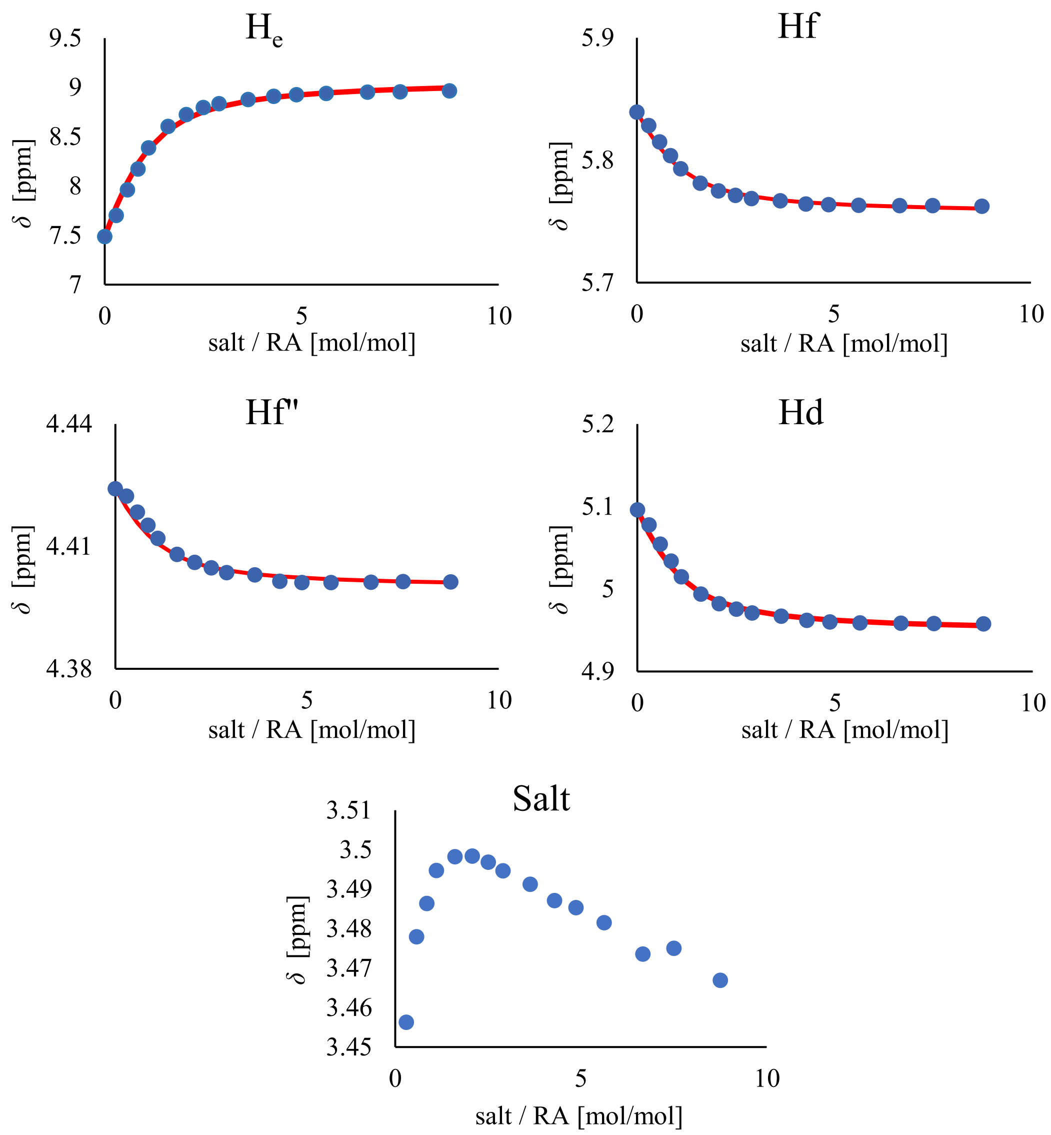

K (M^{-1})	K error (%)	SSR	Datapoints fitted	Params fitted
342.21	± 5.6716	2.5517e-2	64	5

http://app.supramolecular.org/bindfit/view/06dca980-d73e-4ddc-b982-7f80215dea64

Figure S17.^{1}H NMR experimental points and fitted curves for the titration of **3 (RA)** (0.0067 M) with **But$_4$NPhCO$_2$ (salt)**. ^{1}H NMR chemical shifts' changes for: (a) H_e; (b) H_f; (c) $H_{f'}$; (d) H_d; and (e) NCH_2 of the salt (298 K, THF-d_8). Red lines correspond to fitted curves.

UV-vis titration of 4 with Pen_4NCl in THF

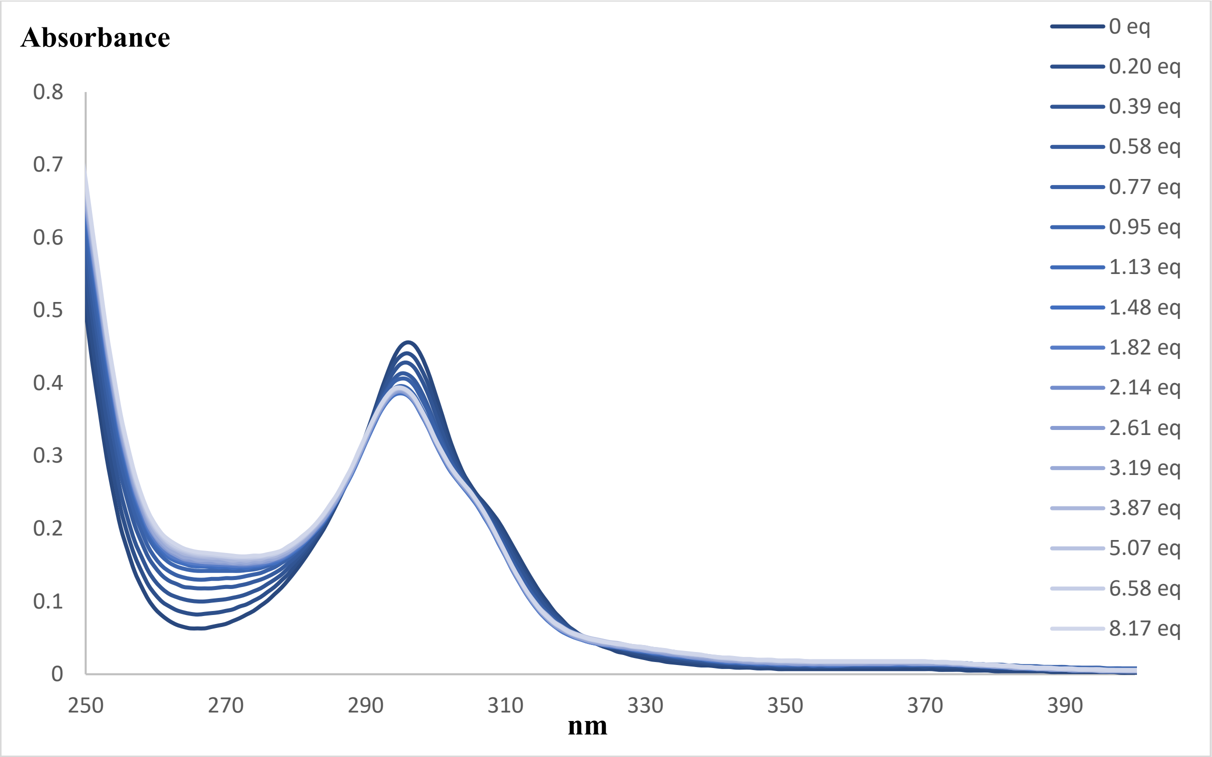

Figure S18. UV-vis spectra for titration of **4** (4.36×10^{-5} M) with **Pen_4NCl** in THF at 298 K.

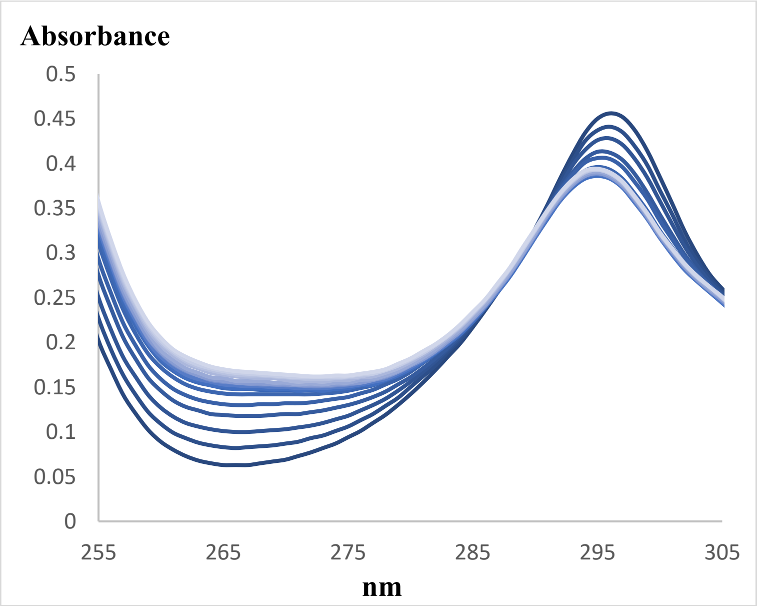

Figure S19. Zoomed UV-vis spectra for titration of **4** (4.36×10^{-5} M) with **Pen_4NCl** in THF at 298 K.

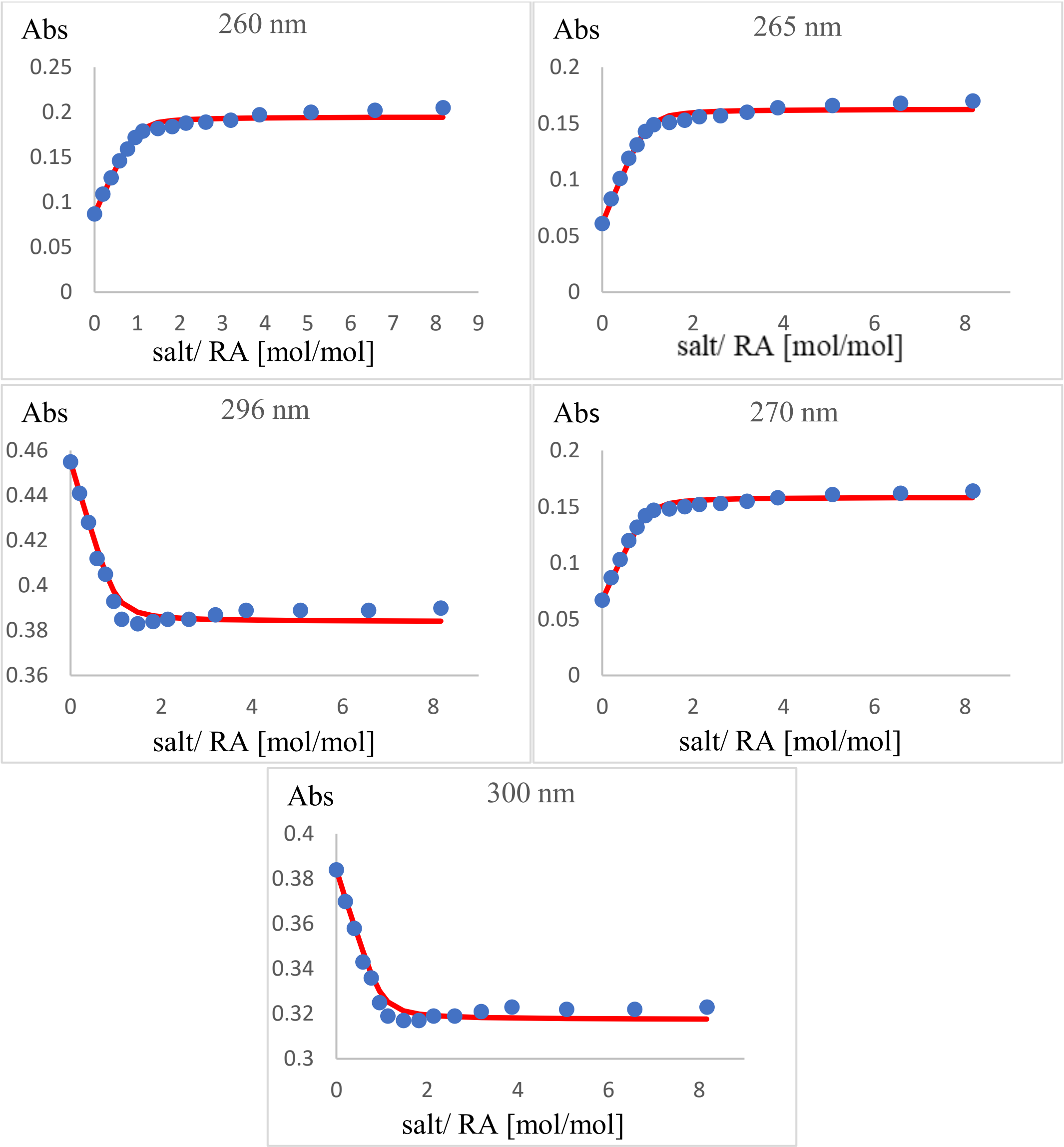

K (M^{-1})	K error (%)	SSR	Datapoints fitted	Params fitted
668954.452	25.85	1.3021e-3	96	7

http://app.supramolecular.org/bindfit/view/a716a359-69c8-455e-850b-9b58d18bbbf3

Figure S20. Experimental points and fitted curves for UV-vis titration of **4 (RA)** (4.36×10^{-5} M) with **Pen_4NCl (salt)** in THF at 298 K. Red lines correspond to fitted curves.

UV-vis titration of 4 with Pen_4NBr in THF

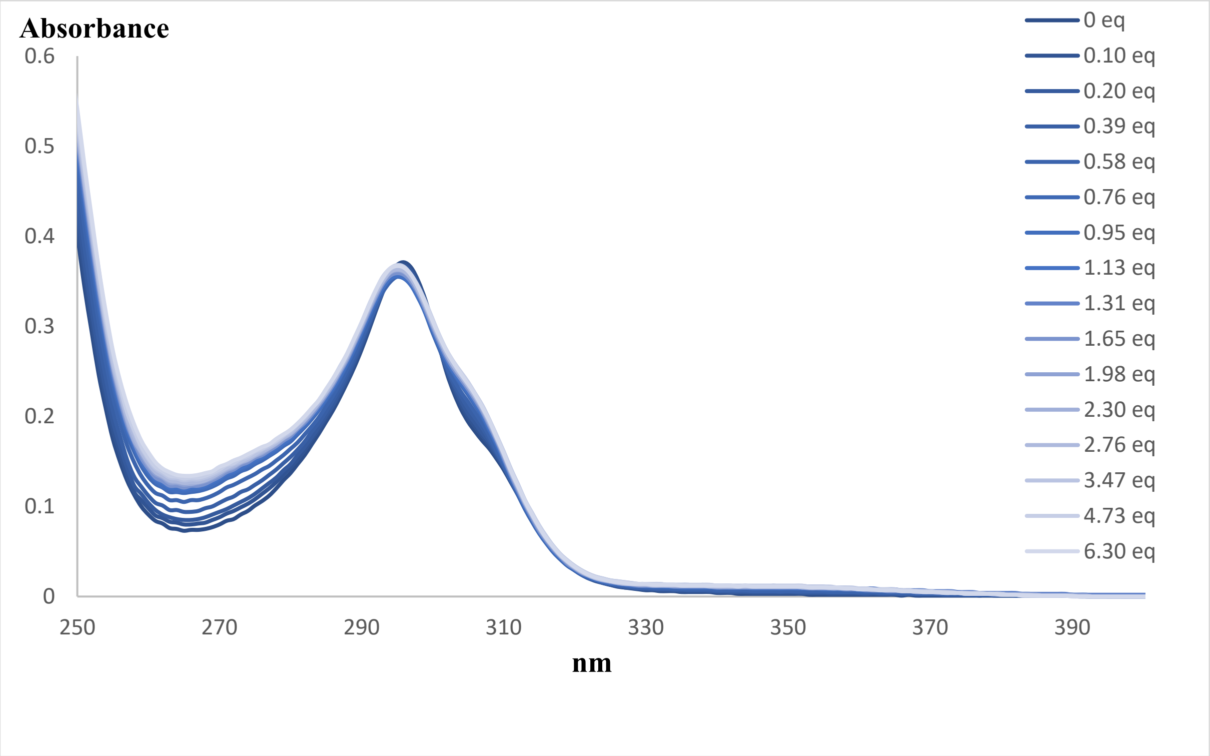

Figure S21. UV-vis spectra for titration of **4** (4.36×10^{-5} M) with **Pen_4NBr** in THF at 298 K.

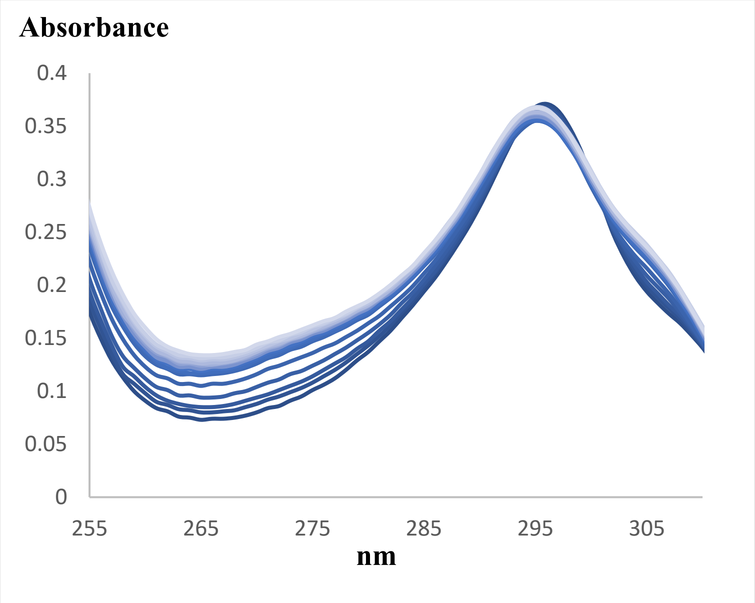

Figure S22. Expanded region of UV-vis spectra for titration of **4** (4.36×10^{-5} M) with **Pen_4NBr** in THF at 298 K.

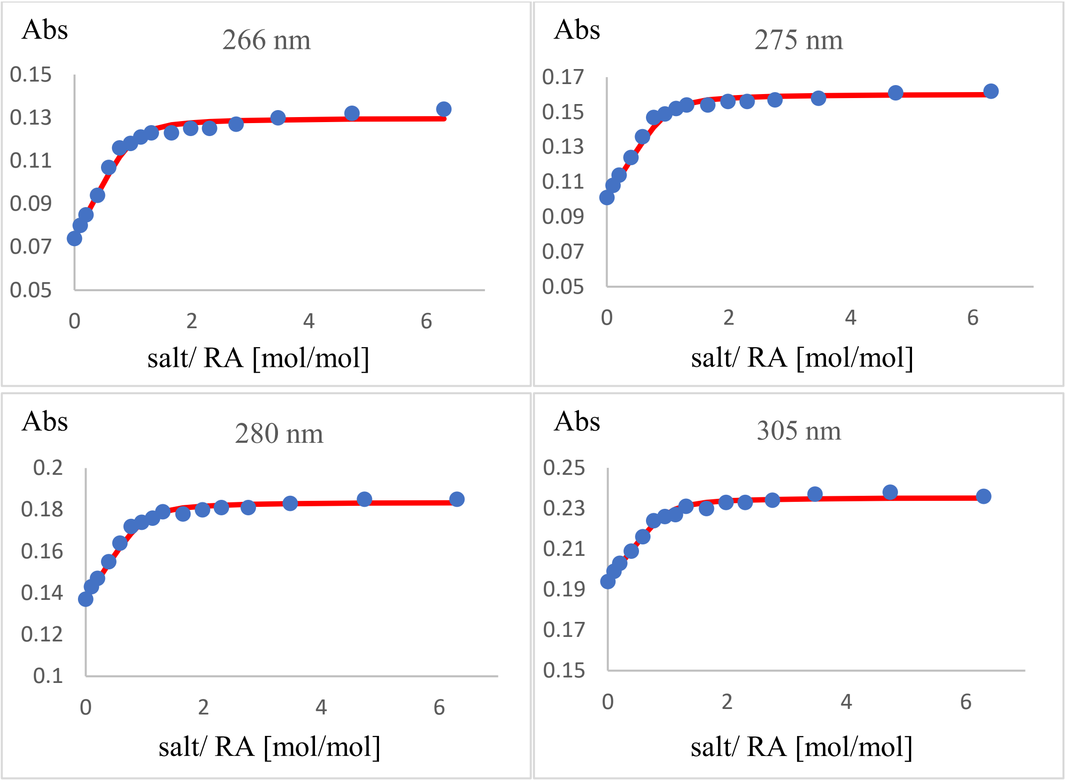

K (M^{-1})	K error (%)	SSR	Datapoints fitted	Params fitted
595993.63	± 23.0476 %	2.6213e-4	64	5

http://app.supramolecular.org/bindfit/view/2ccd3bc0-20a1-4619-8516-8fa917adec87

Figure S23. Experimental points and fitted curves for UV-vis titration of **4 (RA)** (4.36×10^{-5} M) with **Pen_4NBr (salt)** in THF at 298 K. Red lines correspond to fitted curves.

UV-vis titration of 4 with Pen_4NCl in DCM

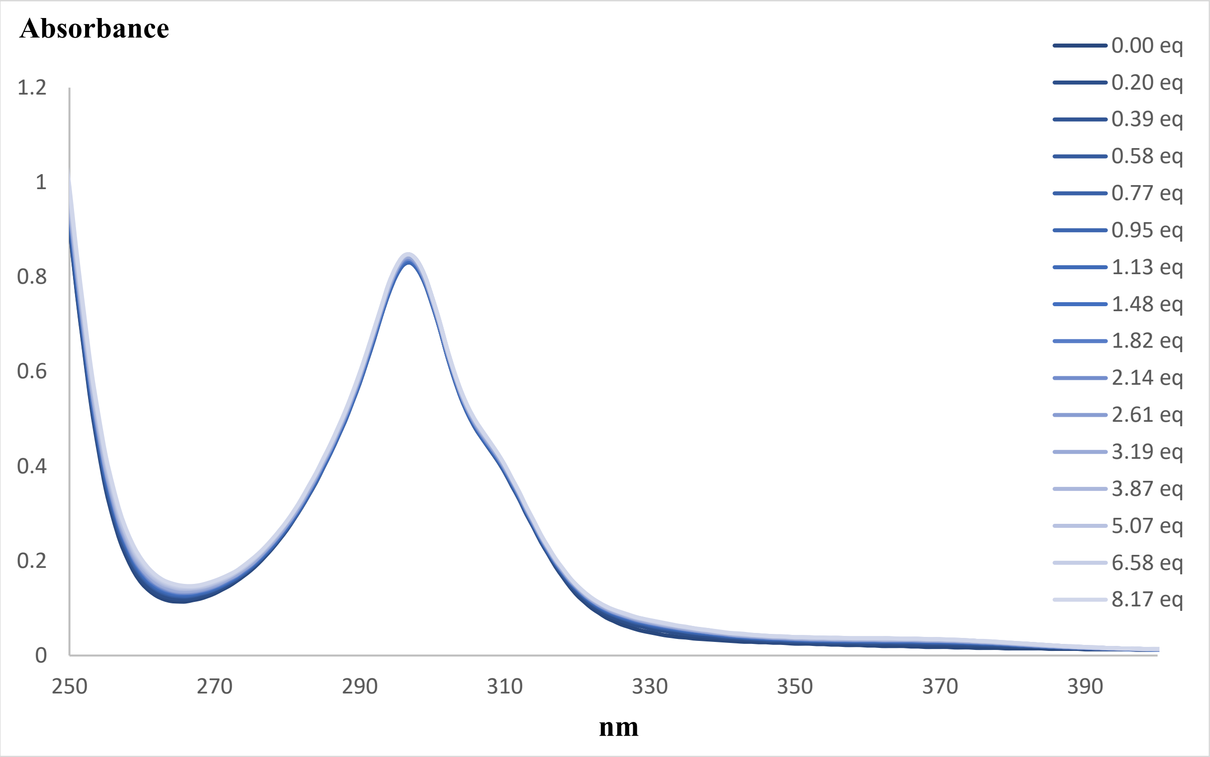

Figure S24. UV-vis titration of **4** (3.48×10^{-5} M) with **Pen_4NCl** in DCM at 298K

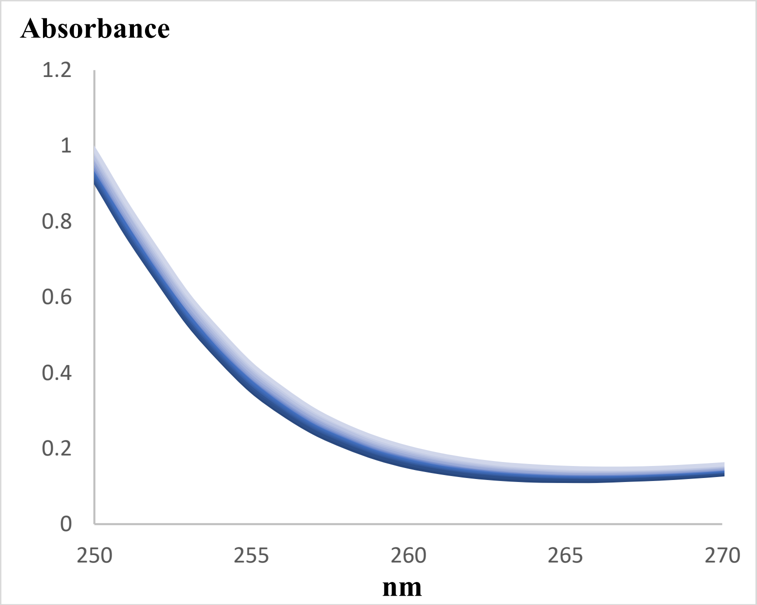

Figure S25. Expanded region of UV-vis titration of **4** (3.48×10^{-5} M) with **Pen_4NCl** in DCM at 298K

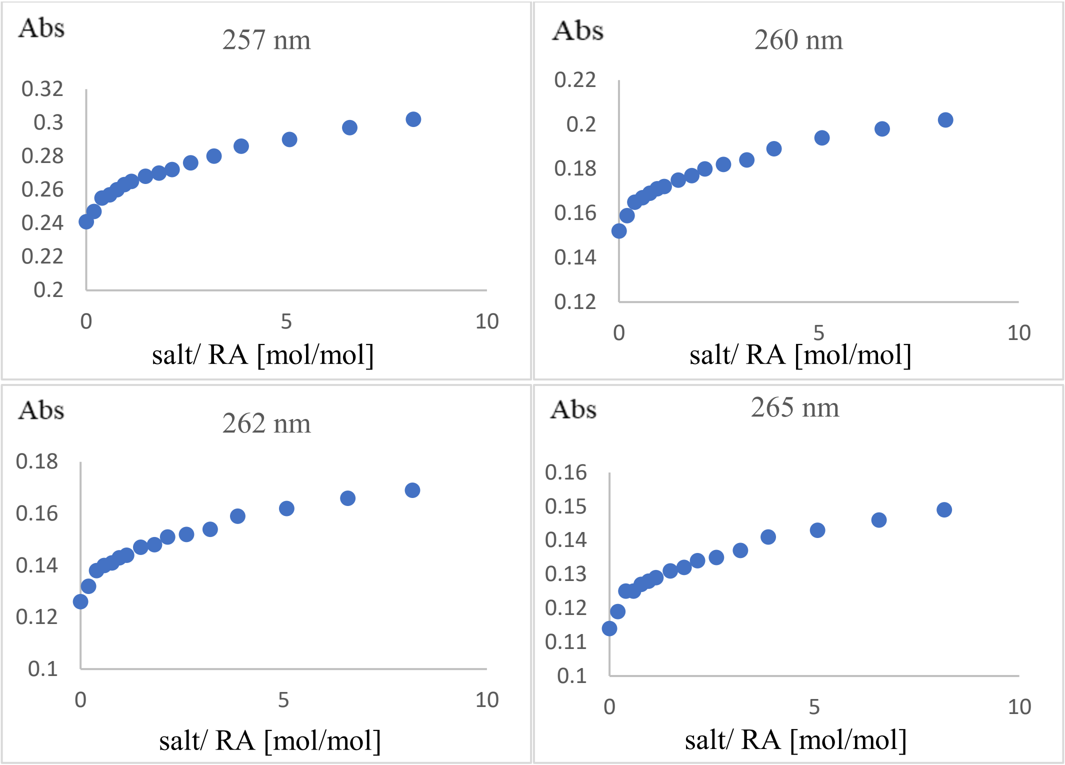

Figure S26. Experimental points for UV-vis titration of **4 (RA)** (3.48×10^{-5} M) with **Pen_4NCl (salt)** in DCM at 298 K. The data was not fitted due to insignificant changes in the absorbance.

UV-vis titration of 4 with Pen_4NCl in $CHCl_3$

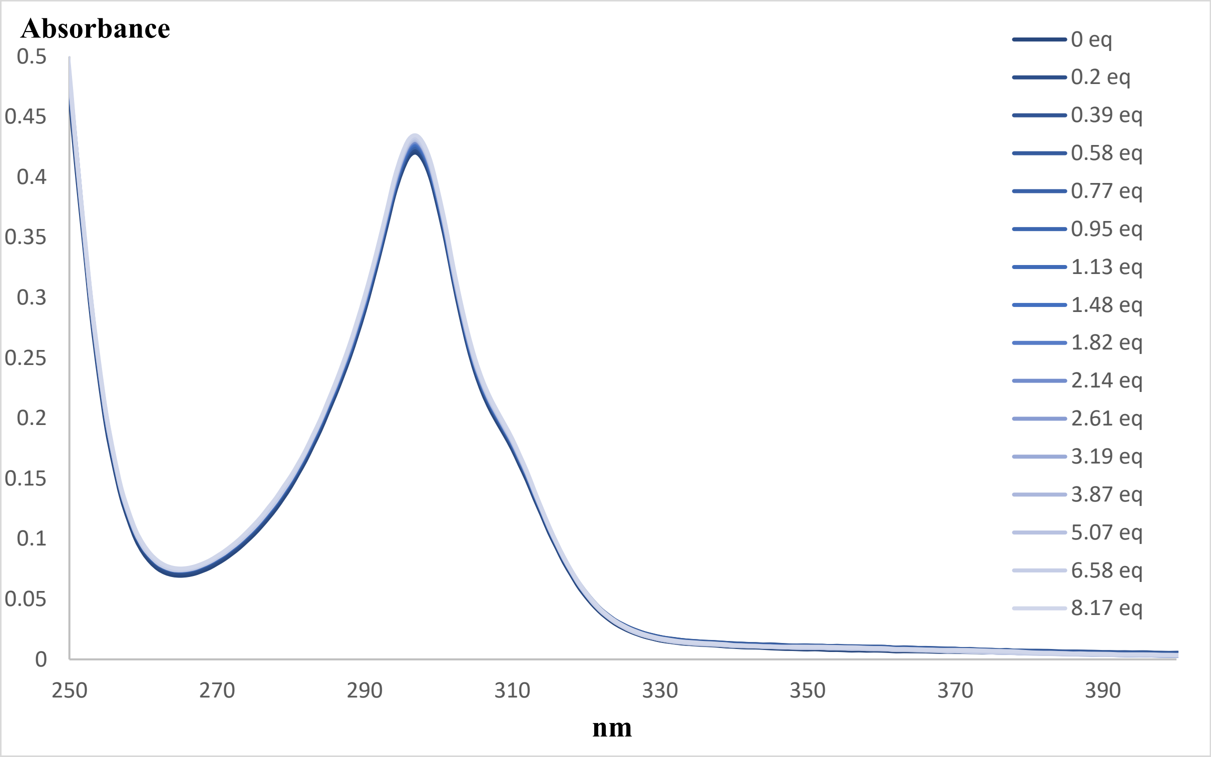

Figure S27. UV-vis titration of **4** (3.48 $\times 10^{-5}$ M) with **Pen_4NCl** in $CHCl_3$ at 298K

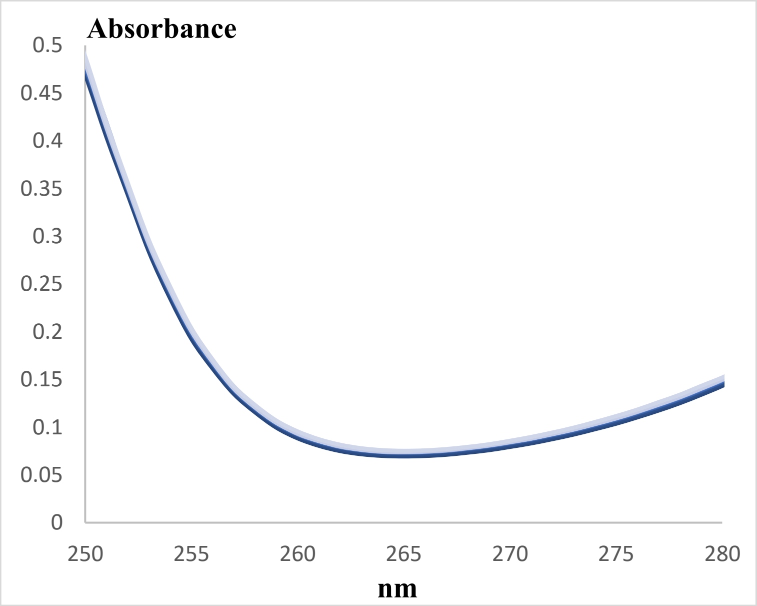

Figure S28. Zoomed UV-vis titration of **4 (RA)** (3.48 $\times 10^{-5}$ M) with **Pen_4NCl (salt)** in $CHCl_3$ at 298 K. The data could be fitted reasonably due to negligible changes.

NMR titration of 4 with Pen_4NCl in $CDCl_3$

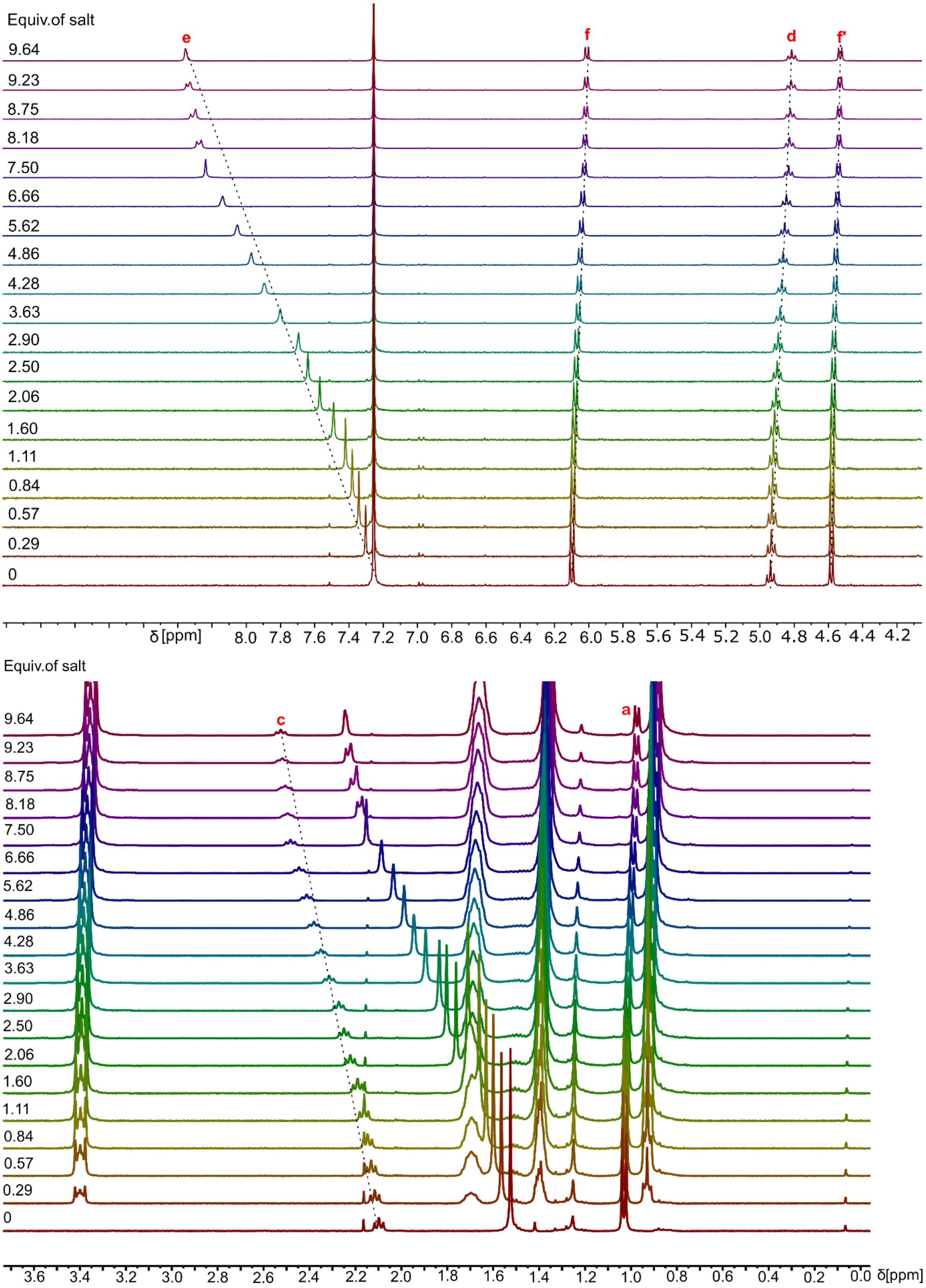

Figure S29. ^{1}H NMR spectra for the titration of **4** with **Pen_4NCl** in $CDCl_3$ at 298K

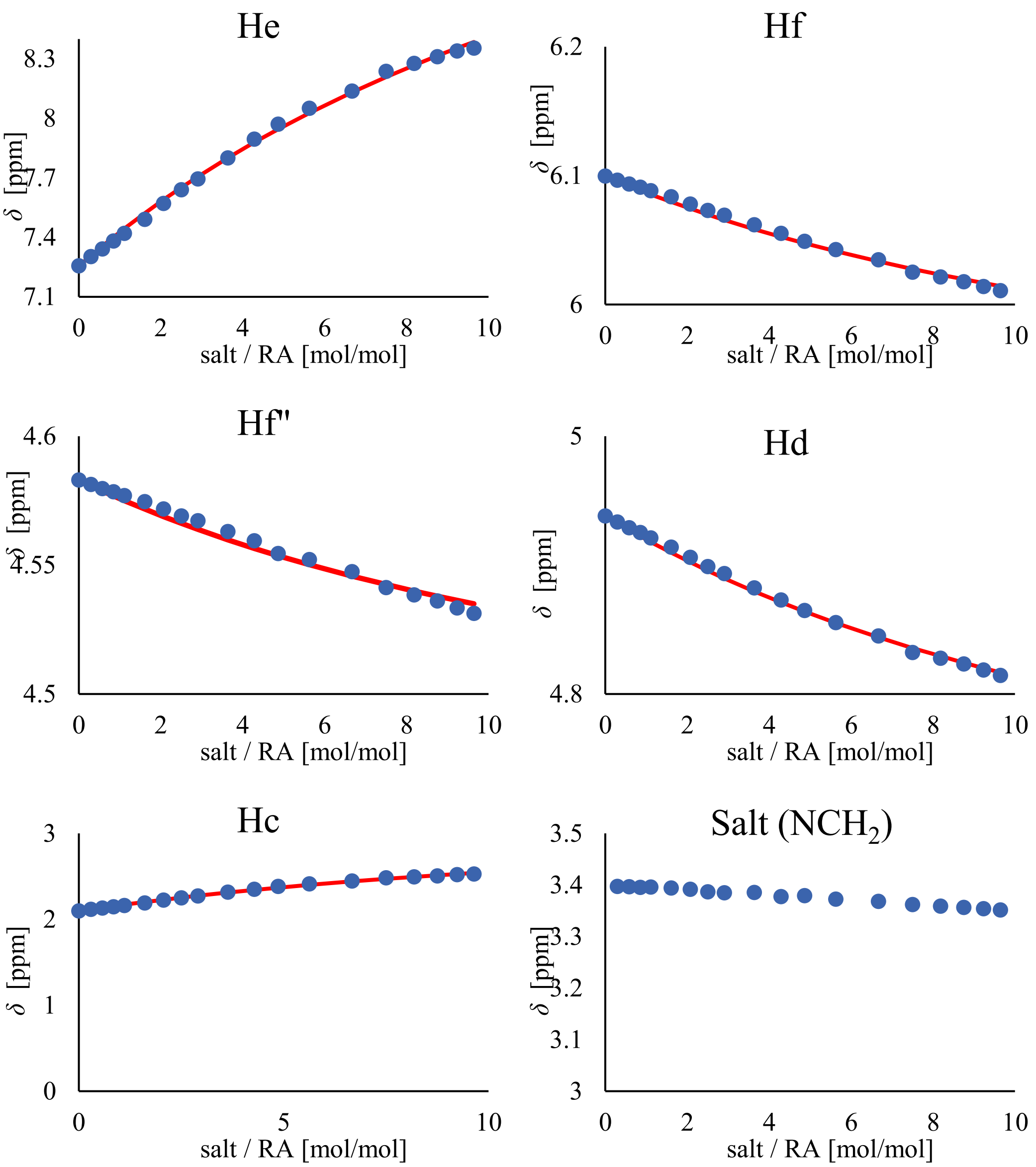

**K(M^{-1})**	**K error (%)**	**SSR**	**Datapoints fitted**	**Params fitted**
9.14	± 0.8635	8.0078e-3	95	6

http://app.supramolecular.org/bindfit/view/602d487a-ac1e-49fe-93fb-3724f52025c0

Figure S30.^{1}H NMR experimental points and fitted curves for the titration of **4** **(RA)** (0.0067 M) with **Pen$_4$NCl (salt).** ^{1}H NMR chemical shifts' changes for: (a) H_e; (b) H_f; (c) $H_{f''}$; (d) H_d; (e) H_c; and (f) NCH_2 of the salt (298 K, $CDCl_3$). Red lines correspond to fitted curves.

NMR titration of 4 with Pen_4NCl in DCM-d_2

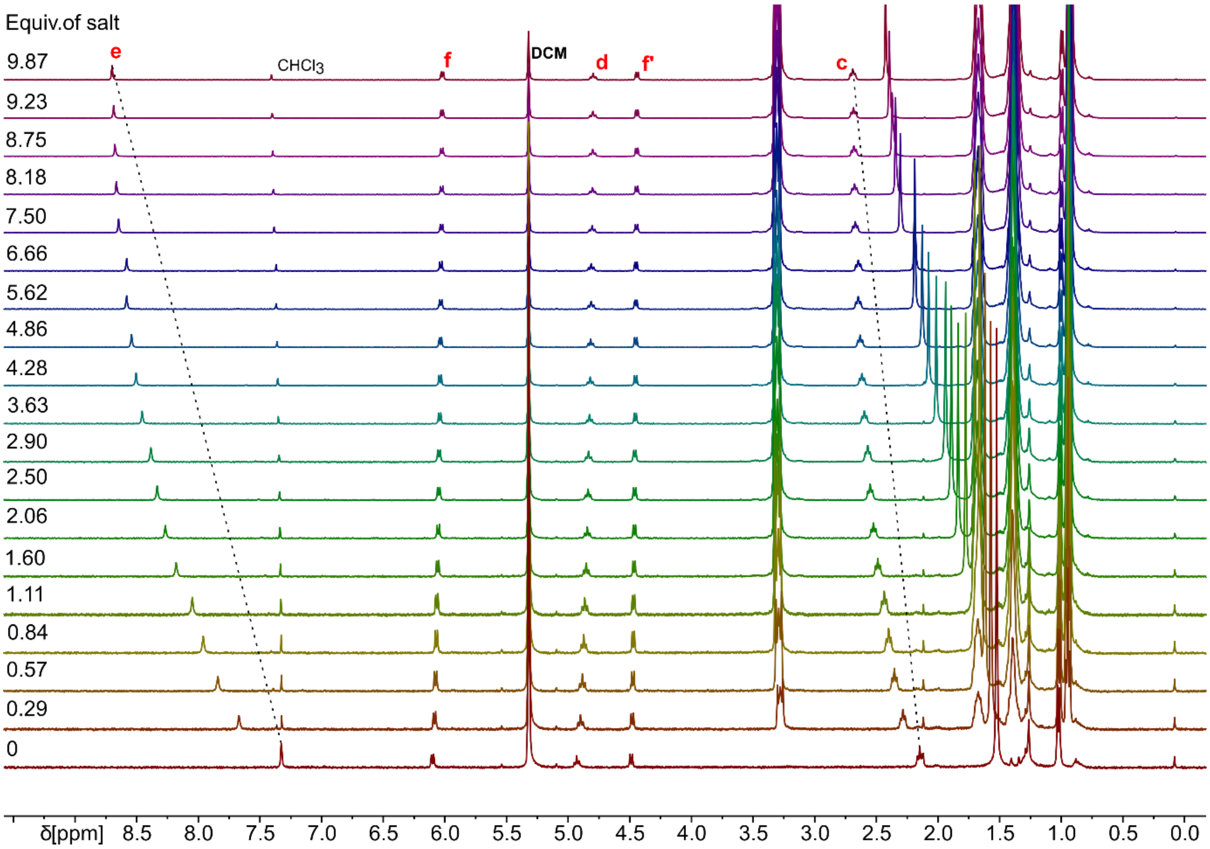

Figure S31. ^1H NMR spectra for the titration of **4** with **Pen_4NCl** in DCM-d_2 at 298K

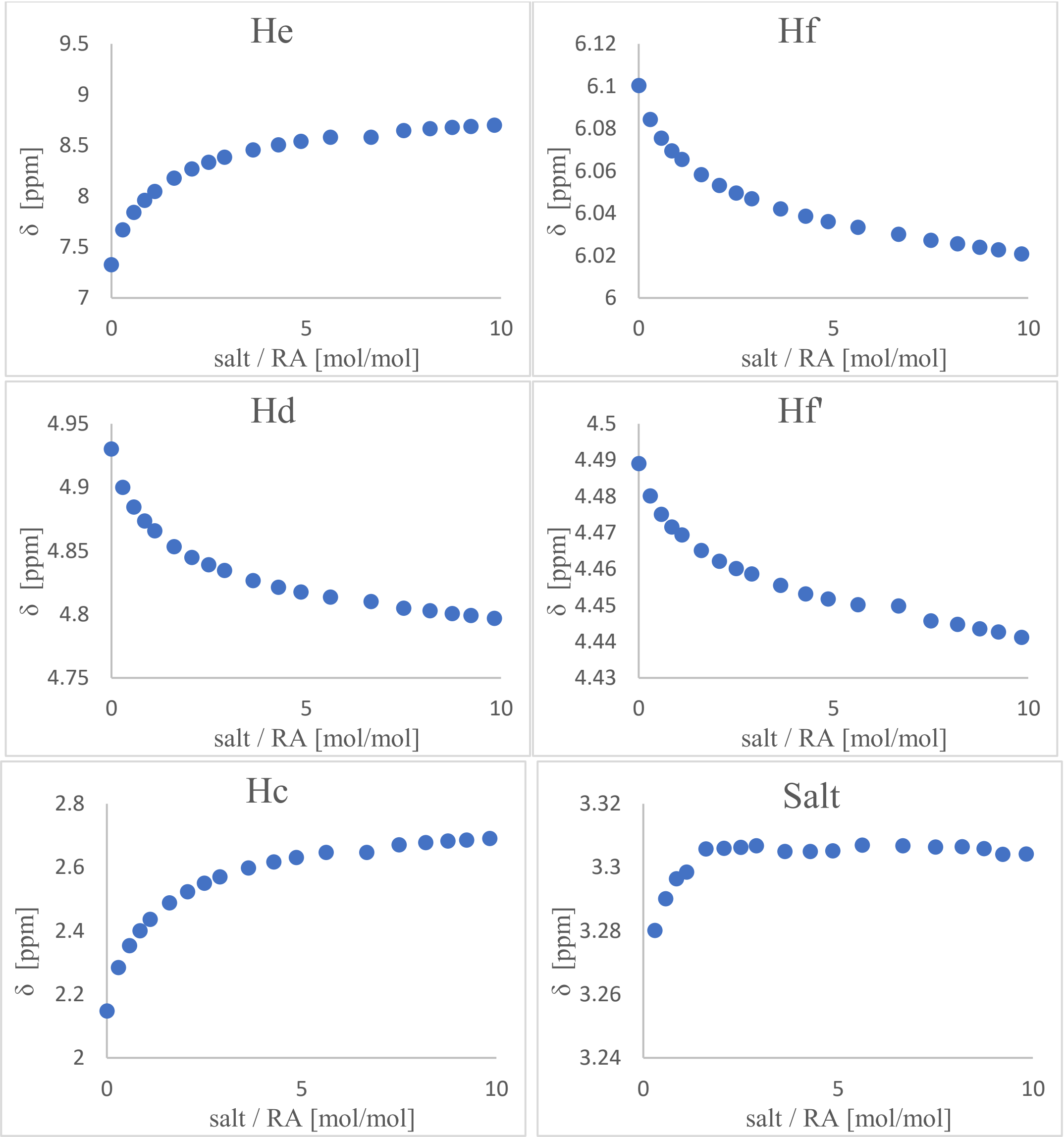

Figure S32.^{1}H NMR experimental points for the titration of **4 (RA)** (0.0067 M) with **Pen$_4$NCl (salt).** ^{1}H NMR chemical shifts' changes for: (a) H_e; (b) H_f; (c) H_d (d) $H_{f'}$; (e) H_c; and (f) NCH_2 of the salt (298 K, DCM-d_2). The data could not be fitted reasonably, due to the solubility issues of the receptor. The receptor was partially soluble in DCM, at this concentration.

NMR titration of 4 (lower concentration) with Pen_4NCl in DCM-d_2

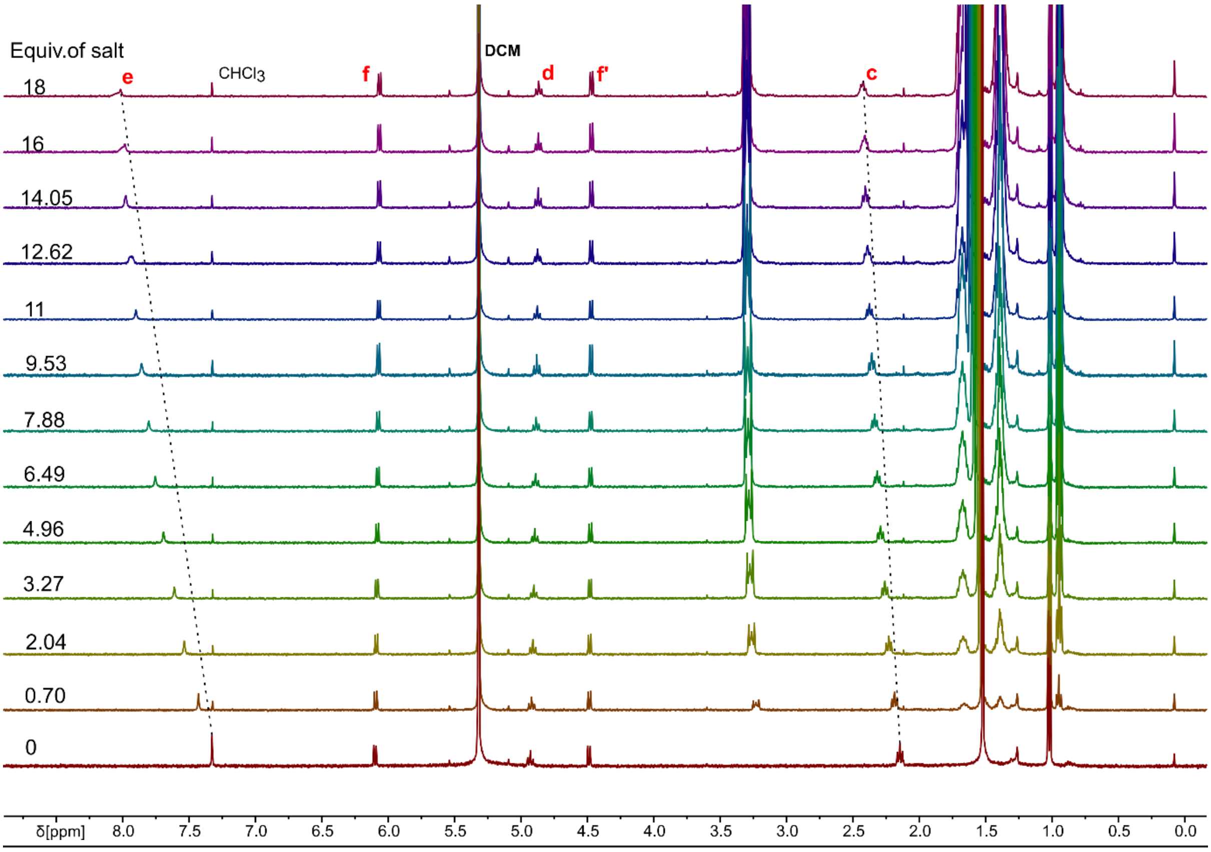

Figure S33. ^{1}H NMR spectra for the titration of **4** with **Pen_4NCl** in DCM-d_2 at 298K. <u>The concentration of receptor **4**, was significantly decreased so as to facilitate solubility in DCM (0.38 mM).</u> At, this concentration the receptor was completely soluble in DCM.

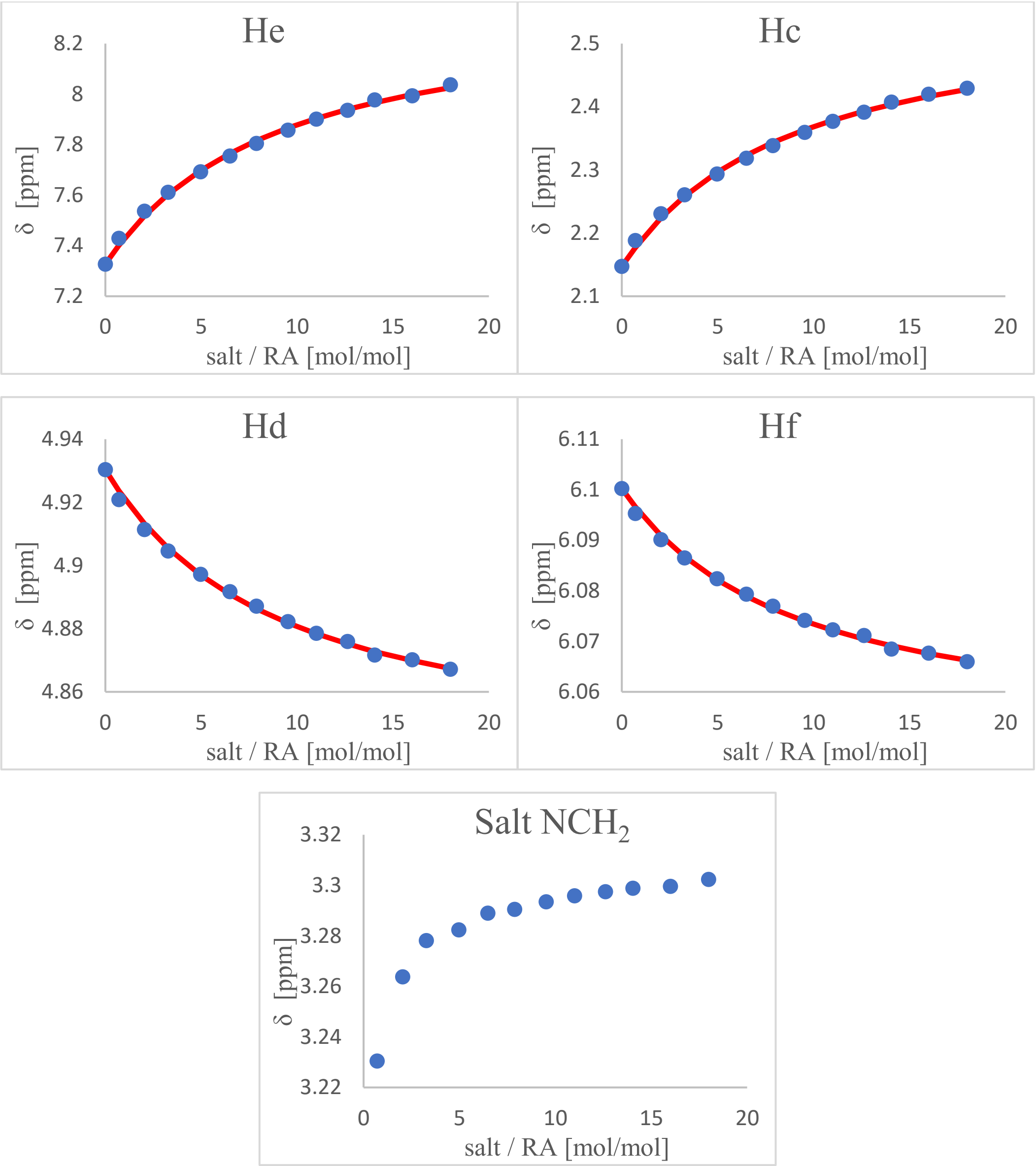

K(M^{-1})	K error (%)	SSR	Datapoints fitted	Params fitted
316.27	± 1.6886	2.1677e-3	52	5

http://app.supramolecular.org/bindfit/view/43bb8e33-1065-471b-8c05-9fde03059f9e

Figure S34.^{1}H NMR experimental points for the titration of **4 (RA)** (0.38 mM) with **Pen$_4$NCl (salt).** ^{1}H NMR chemical shifts' changes for: (a) H_e; (b) H_c; (c) H_d (d) $H_{f'}$; (e) NCH_2 of the salt (298 K, DCM-d_2). Red lines correspond to fitted curves.

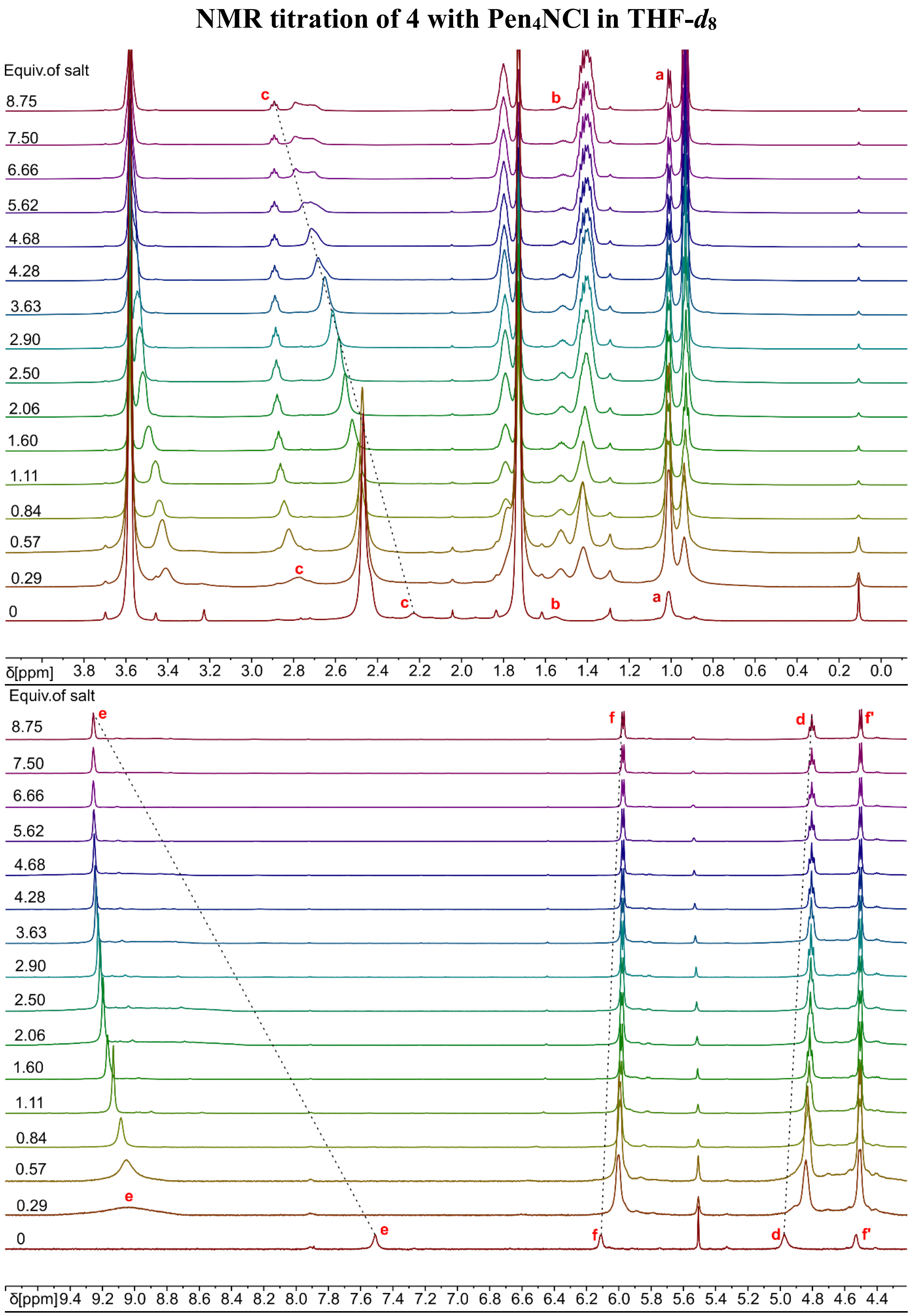

Figure S35. ^{1}H NMR spectra for the titration of **4** with **Pen_4NCl** in THF-d_8 at 298K

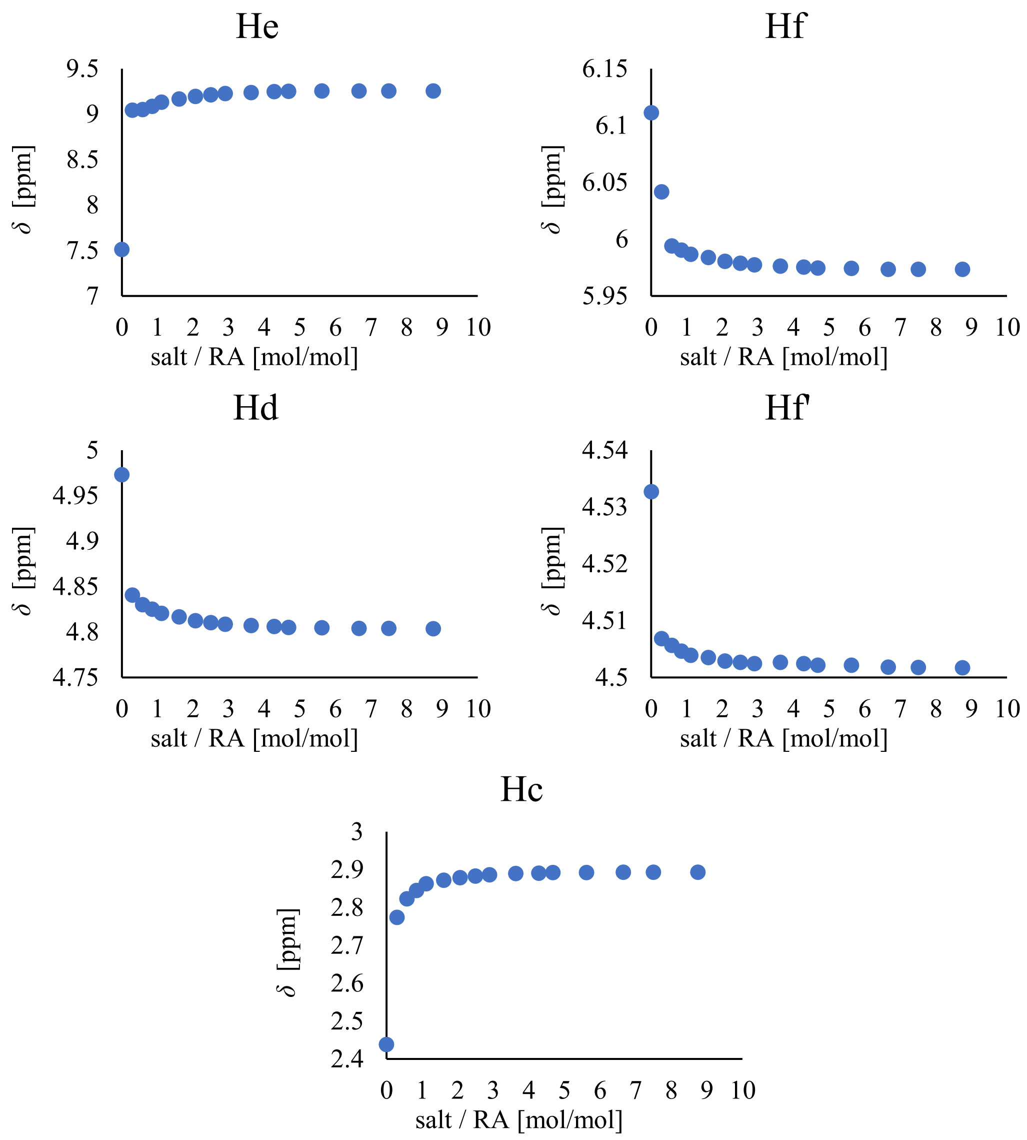

Figure S36.^{1}H NMR experimental points for the titration of **4 (RA)** (0.0067 M) with **Pen$_4$NCl (salt).** ^{1}H NMR chemical shifts' changes for: (a) H$_e$; (b) H$_f$; (c) H$_d$ (d) H$_{f'}$; (e) H$_c$; (298 K, THF-d_8). The data could not be fitted reasonably, due to the solubility issues of the receptor at this concentration in THF-d_8, which can be inferred from the poor quality of spectra at 0 equiv. of the salt. The [receptor-anion complex] started being soluble after the addition of approximately 1 equiv. of the salt.

NMR titration of 5 with Pen_4NCl in THF-d_8 at 255 K

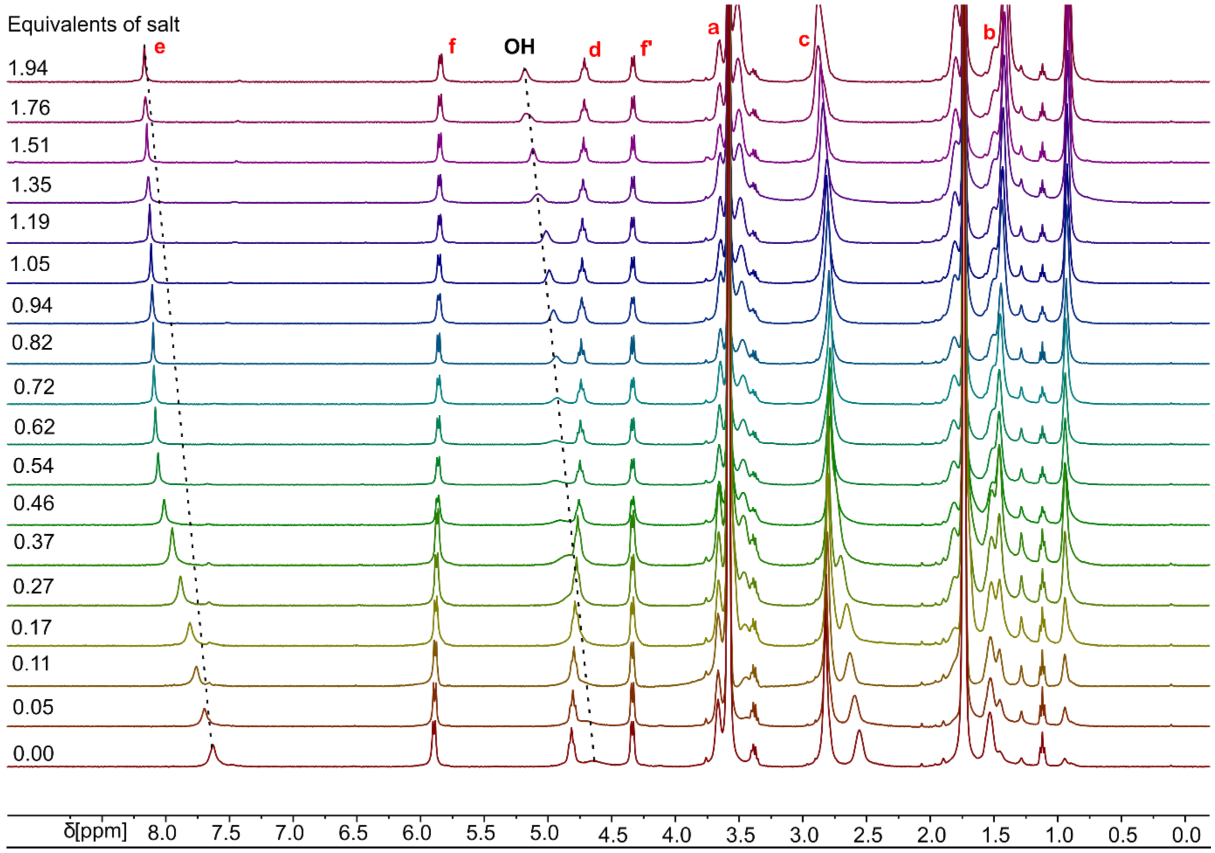

Figure S37.^{1}H NMR spectra for the titration of **5** (0.0067 M) with **Pen_4NCl** in THF-d_8 at 255 K.

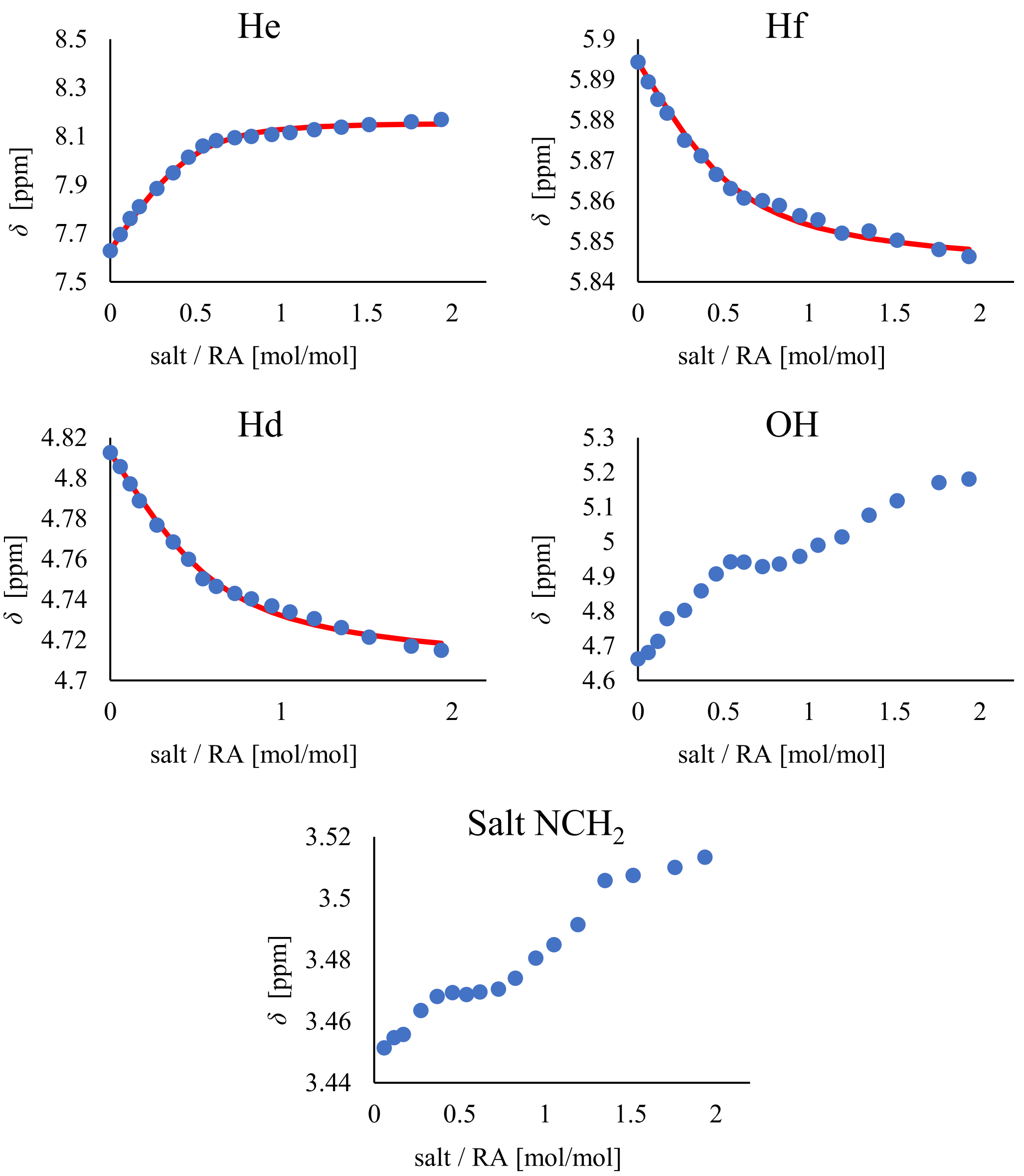

K11(M–1)	K error (%)	SSR	Datapoints fitted	Params fitted
1562.36	± 4.9048	2.2136e-3	54	7

http://app.supramolecular.org/bindfit/view/c524a8d0-2196-4b10-9b69-ea82194c8f98

Figure S38. ^{1}H NMR experimental points and fitted curves for the titration of **5 (RA)** (0.0067 M) with **Pen$_4$NCl (salt)**. ^{1}H NMR chemical shifts' changes for: (a) H_e; (b) H_f; (c) H_d; (d) H_{OH}; and (f) NCH_2 of the salt (255 K, THF-d_8). The above data has been fitted in a 2:1 binding model (Non-cooperative/ Nelder-mead). Red lines correspond to fitted curves.

NMR titration of 5 with Pen_4NBr in THF-d_8 at 255 K

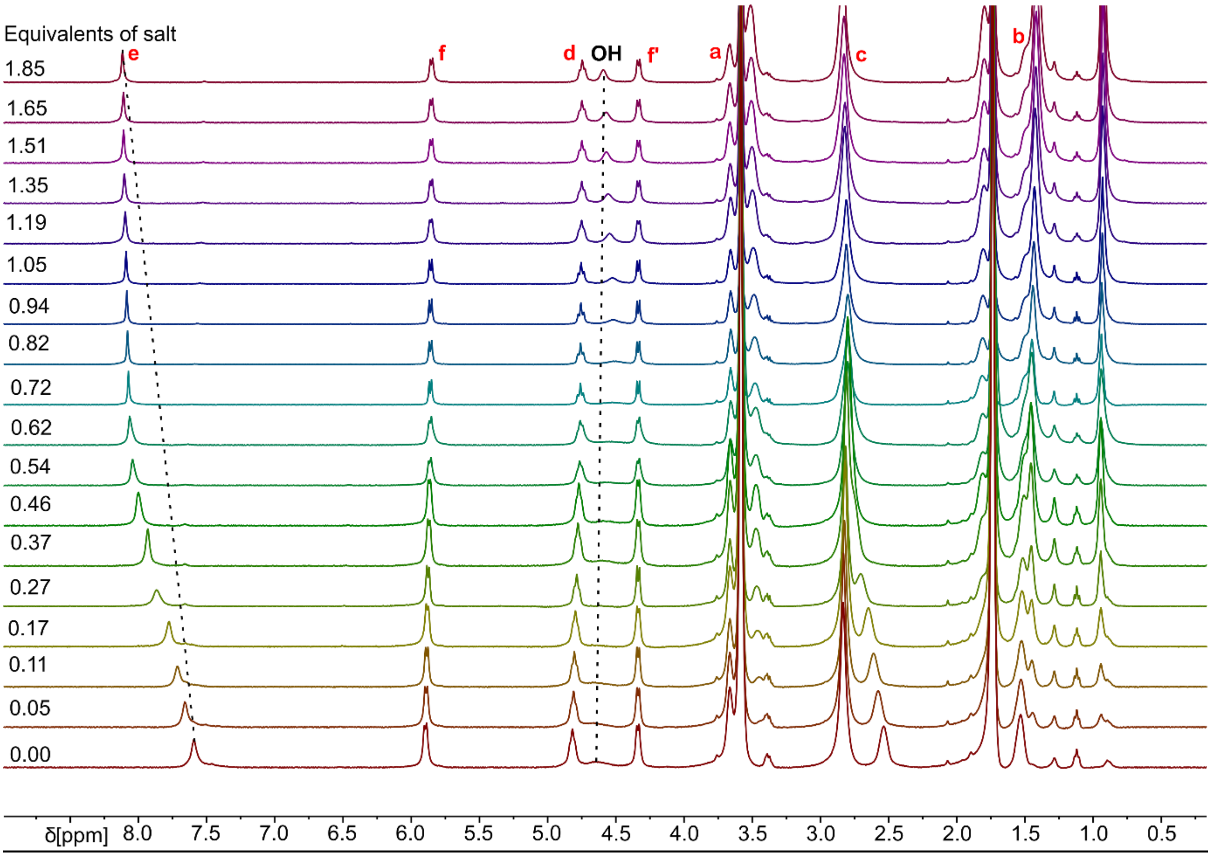

Figure S39. ^{1}H NMR spectra for the titration of **5** (0.0067 M) with **Pen_4NBr** in THF-d_8 at 255 K.

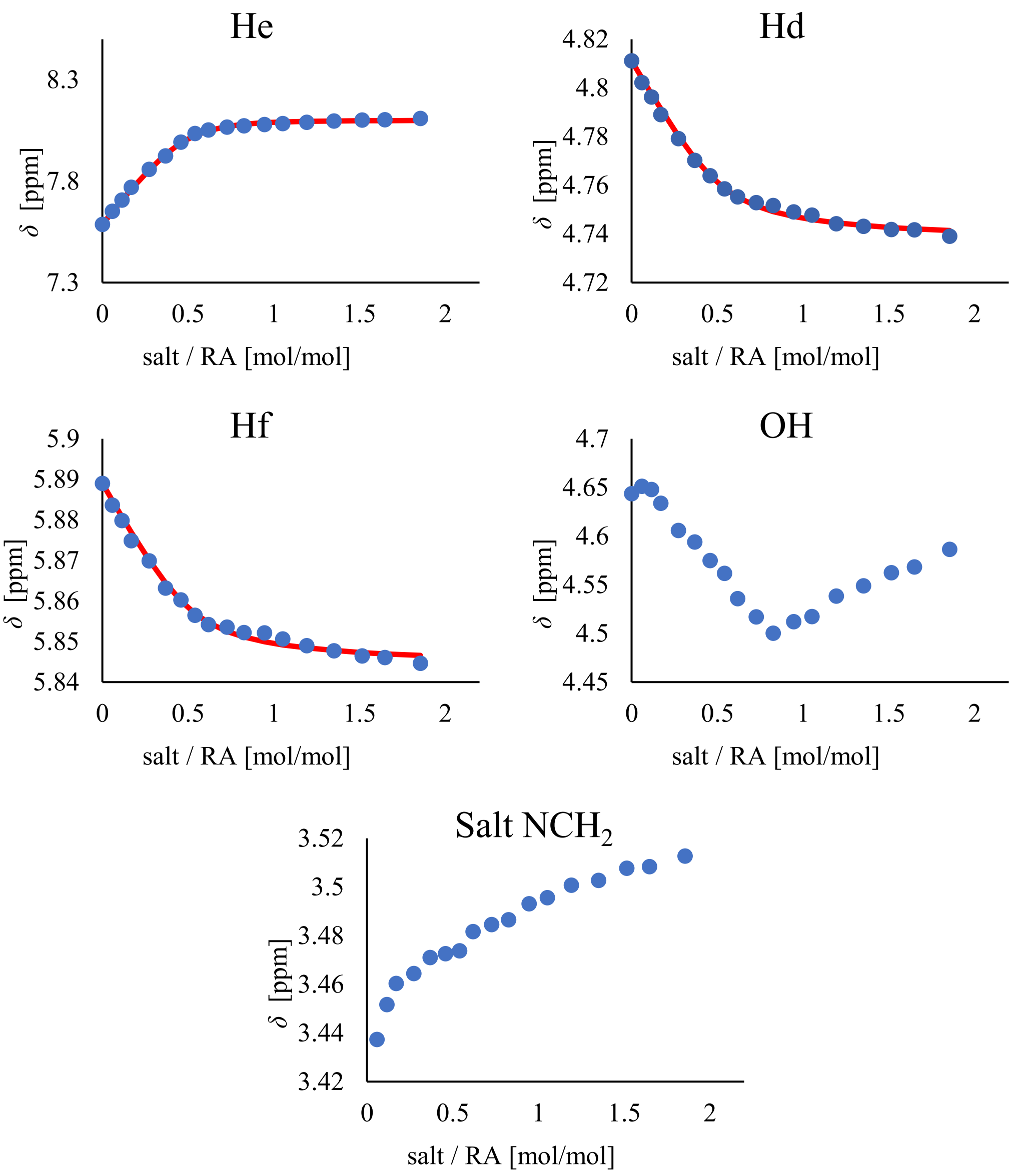

K$_{11}$(M^{-1})	K error (%)	SSR	Datapoints fitted	Params fitted
3788.69	± 4.9813	7.2844e-4	54	7

http://app.supramolecular.org/bindfit/view/8e3f4de1-fcbd-465d-8dc2-3b8c41ef5f62

Figure S40. ^{1}H NMR experimental points and fitted curves for the titration of **5 (RA)** (0.0067 M) with **Pen$_4$NCl (salt)**. ^{1}H NMR chemical shifts' changes for: (a) H$_e$; (b) H$_d$; (c) H$_f$; (d) H$_{OH}$; and (e) NCH$_2$ of the salt (255 K, THF-d_8). The above data has been fitted in a 2:1 binding model (Non-cooperative/ Nelder-mead). Red lines correspond to fitted curves.

NMR titration of 5 with But_4NReO_4 in THF-d_8 at 255 K

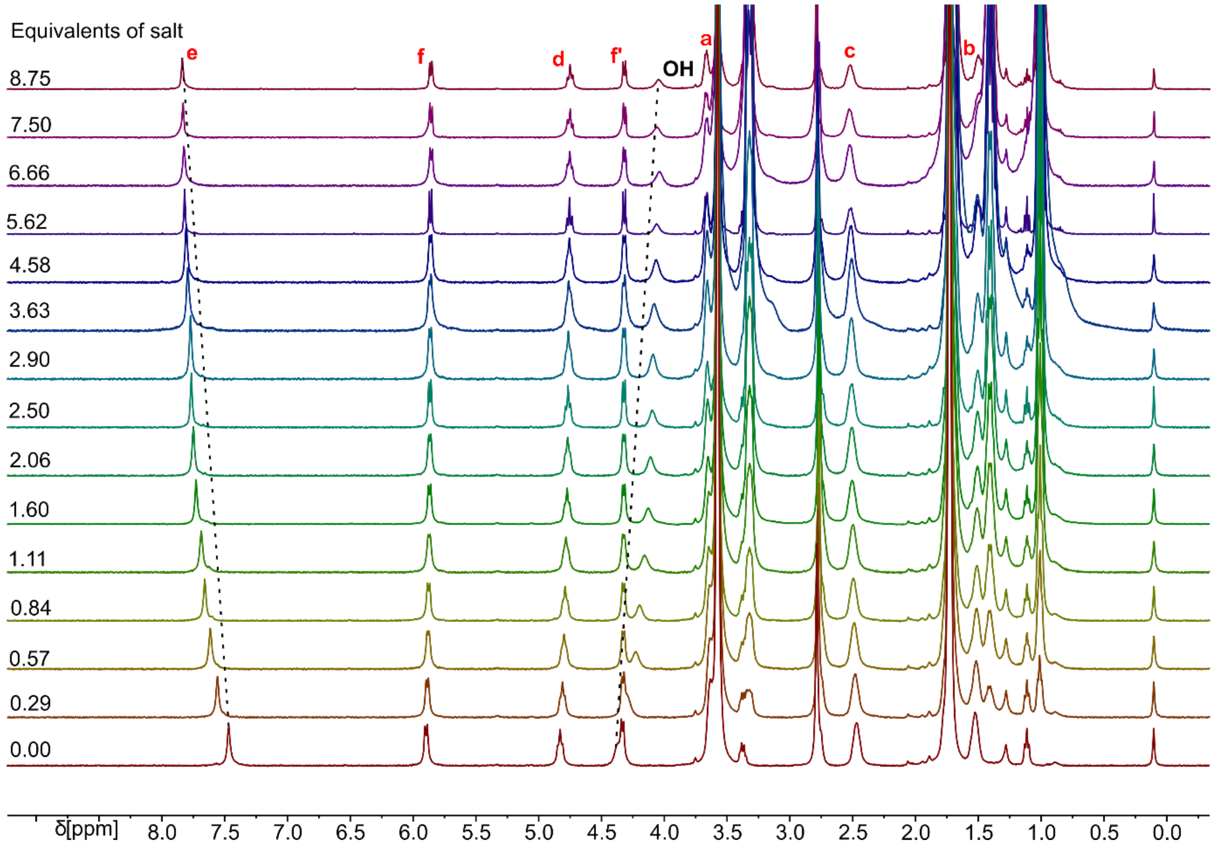

Figure S41. ^{1}H NMR spectra for the titration of **5** (0.0067 M) with **But_4NReO_4** in THF-d_8 at 255 K.

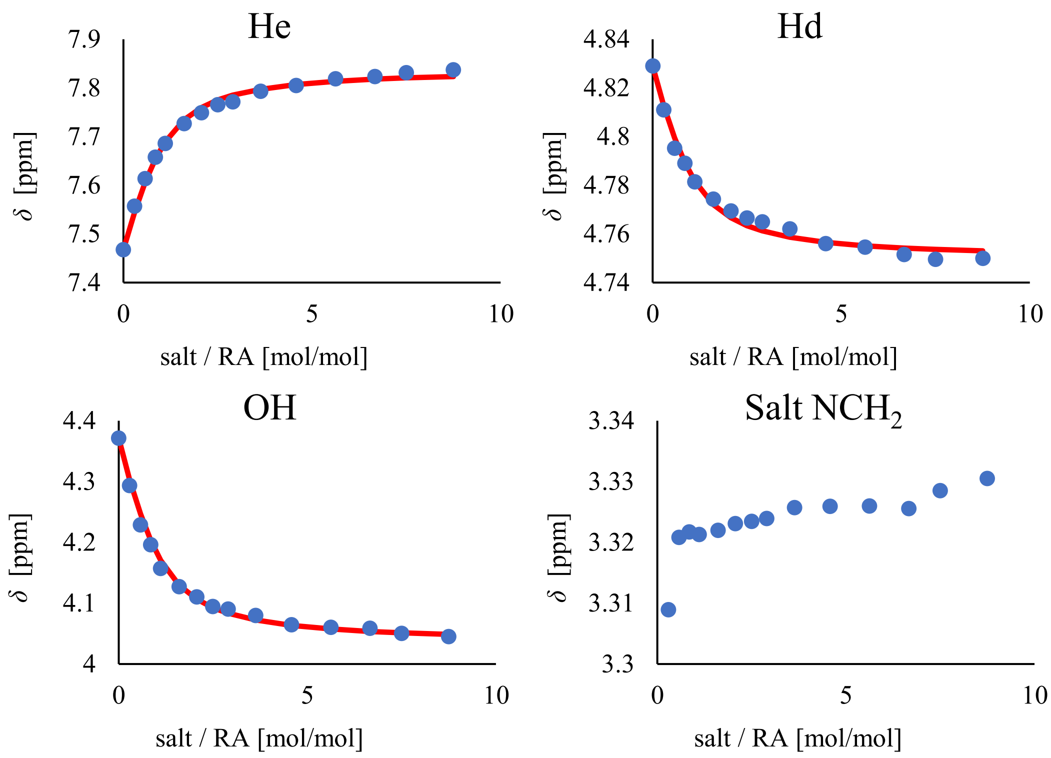

**K_{11}(M^{-1})**	**K error (%)**	**SSR**	**Datapoints fitted**	**Params fitted**
429.066256	6.83463947	0.00201208	45	4

http://app.supramolecular.org/bindfit/view/d647b19c-b492-4787-ac0b-7adc596b5924

Figure S42. ^{1}H NMR experimental points and fitted curves for the titration of **5 (RA)** (0.0067 M) with **But_4NReO_4 (salt)**. ^{1}H NMR chemical shifts' changes for: (a) H_e; (b) H_d; (c) H_{OH}; and (d) NCH_2 of the salt (255 K, THF-d_8). Red lines correspond to fitted curves.

NMR titration of 5 with Pen_4NCl in THF-d_8/10% D_2O

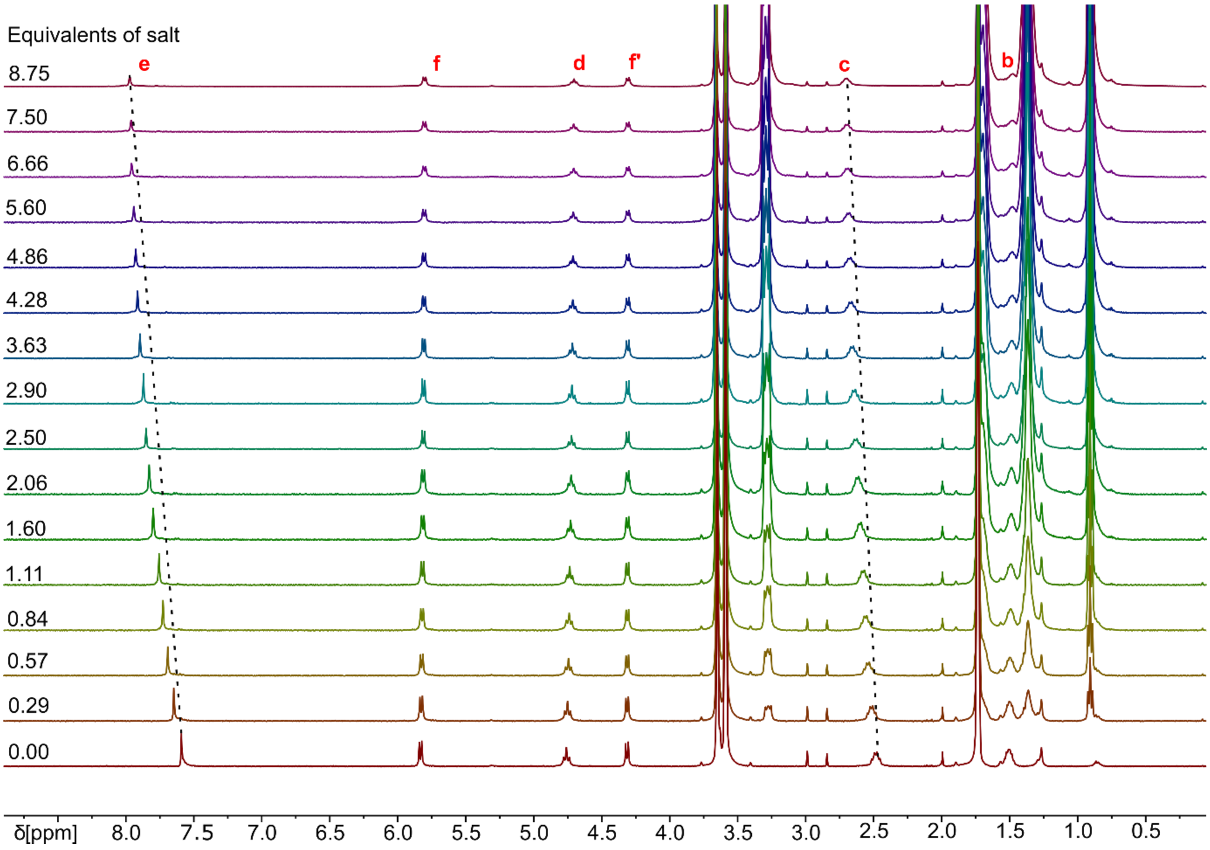

Figure S43. ^{1}H NMR spectra for the titration of **5** (0.0067 M) with **Pen_4NCl** in THF-d_8/ 10% D_2O at 298 K.

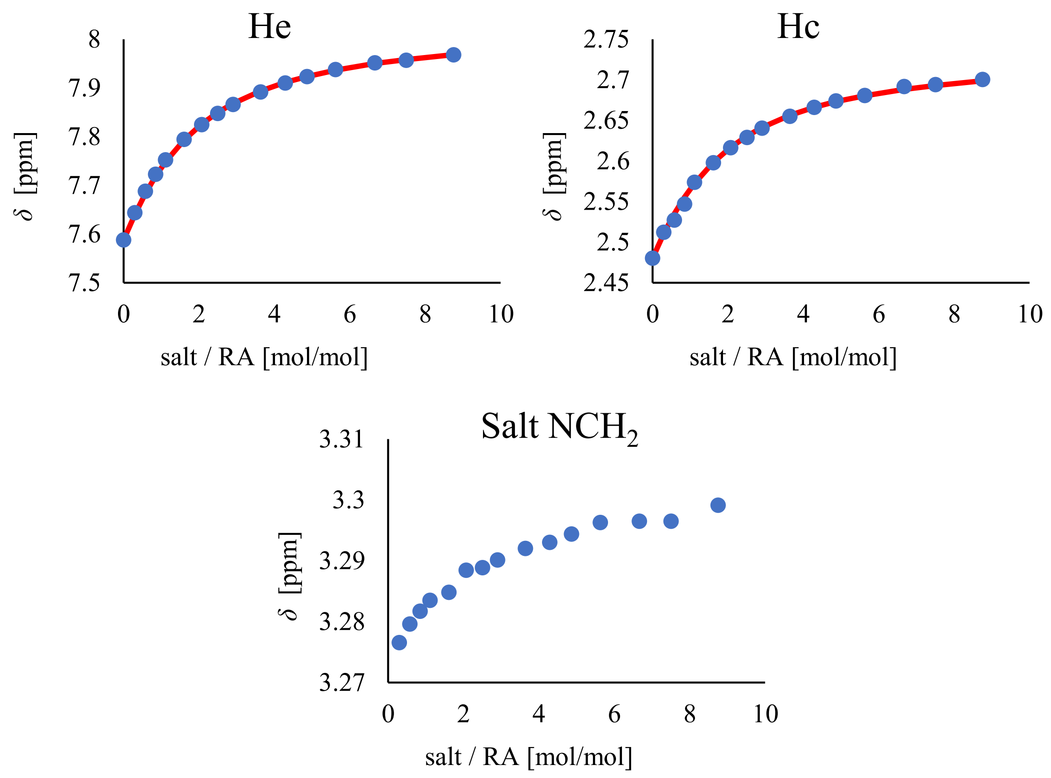

K_{11}(M^{-1})	*K* error(%)	SSR	Datapoints fitted	Params fitted
112.21	±1.8043	2.5156e-4	32	3

http://app.supramolecular.org/bindfit/view/57798932-770d-4cb8-97b0-c2a382447561

Figure S44. ^{1}H NMR experimental points and fitted curves for the titration of **5 (RA) (**0.0067 M) with **Pen$_4$NCl (salt)** ^{1}H NMR chemical shifts' changes for: (a) H_e; (b) H_c and (c) NCH_2 of the salt (298 K, THF-*d*$_8$/ 10% D_2O). Red lines correspond to fitted curves.

NMR titration of 5 with But_4NHSO_4 in THF-d_8/10% D_2O

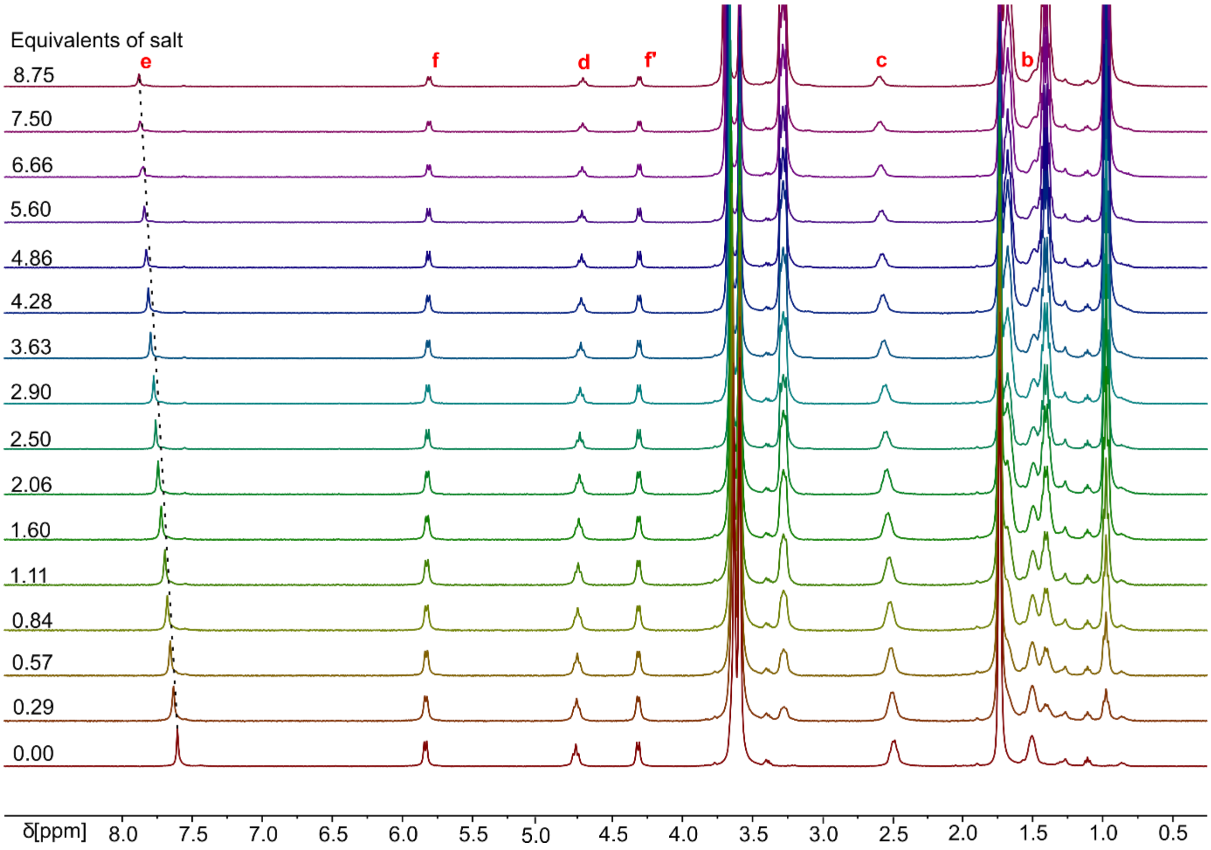

Figure S45. ^{1}H NMR spectra for the titration of **5** (0.0067 M) with **But_4NHSO_4** in THF-d_8/10% D_2O at 298 K.

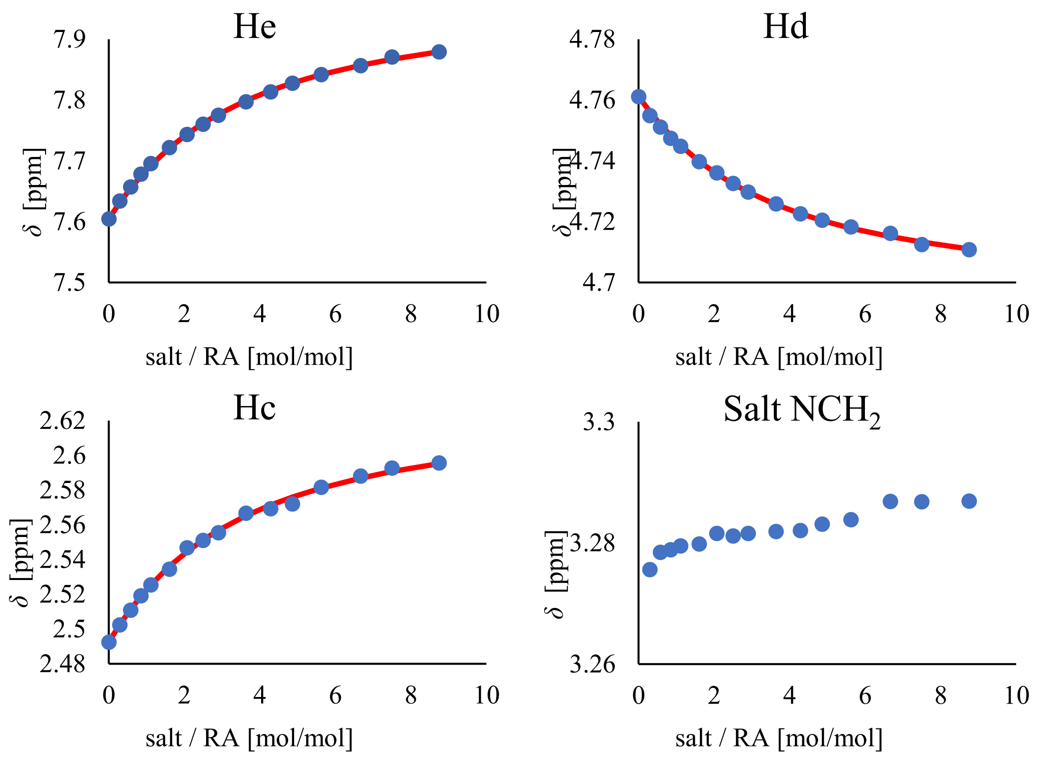

K_{11}(M^{-1})	K error (%)	SSR	Datapoints fitted	Params fitted
52.90	± 1.0425	1.0295e-4	48	4

http://app.supramolecular.org/bindfit/view/4dbefffd-55a5-4079-aef7-a058024634ac

Figure S46. ^{1}H NMR experimental points and fitted curves for the titration of **5 (RA)** (0.0067 M) with **But$_4$NHSO$_4$ (salt)** ^{1}H NMR chemical shifts' changes for: (a) H_e; (b) H_d (c) H_c and (d) NCH_2 of the salt (298 K, THF-d_8/10%D_2O). Red lines correspond to fitted curves.

NMR titration of 5 with $But_4NH_2PO_4$ in THF-d_8/10% D_2O

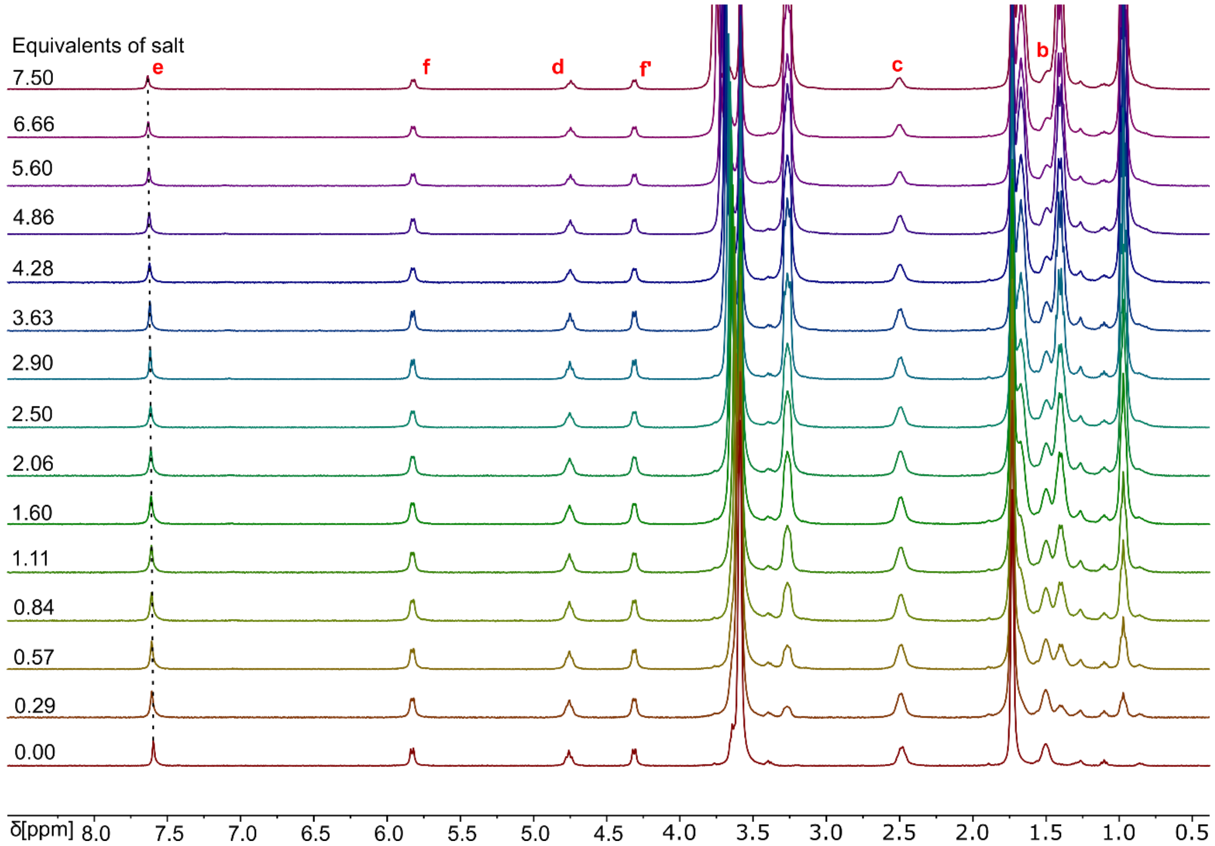

Figure S47. ^{1}H NMR spectra for the titration of **5** (0.0067 M) with **$But_4NH_2PO_4$** in THF-d_8/10% D_2O at 298 K.

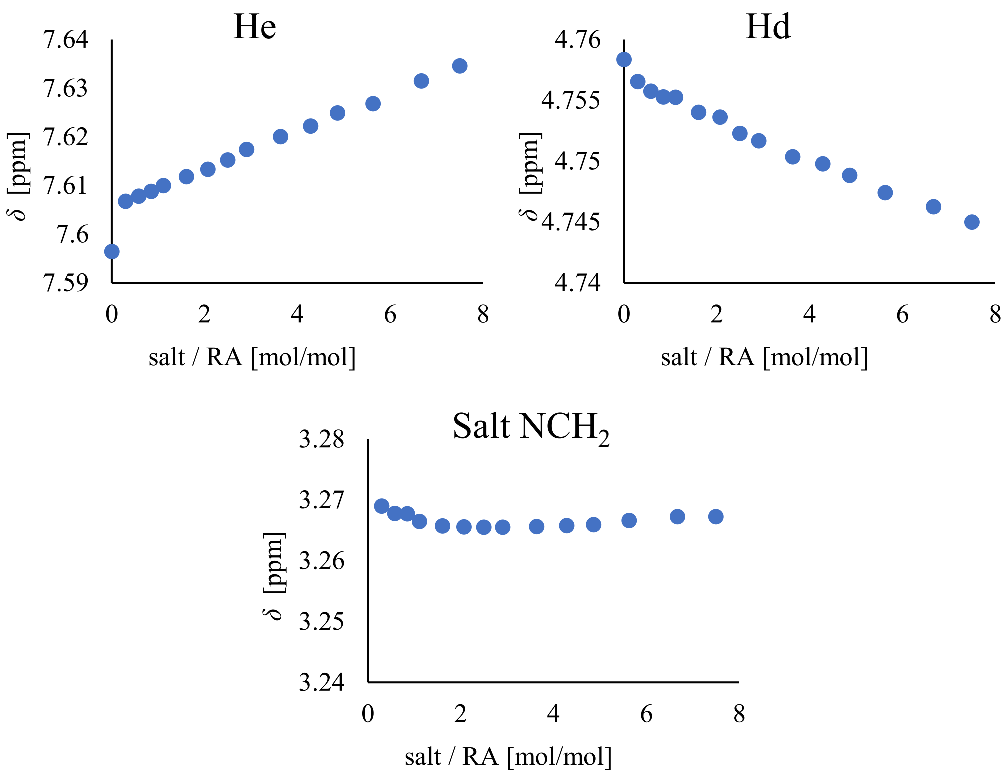

Figure S48. ^{1}H NMR experimental points for the titration of **5 (RA)** (0.0067 M) with **But$_4$NH$_2$PO$_4$ (salt)** ^{1}H NMR chemical shifts' changes for: (a) H$_e$; (b) H$_d$ and (c) NCH$_2$ of the salt (298 K, THF-d_8/10%D$_2$O). <u>The data could not be fitted due to negligible binding.</u>

NMR titration of 5 with But_4NClO_4 in THF-d_8/10% D_2O

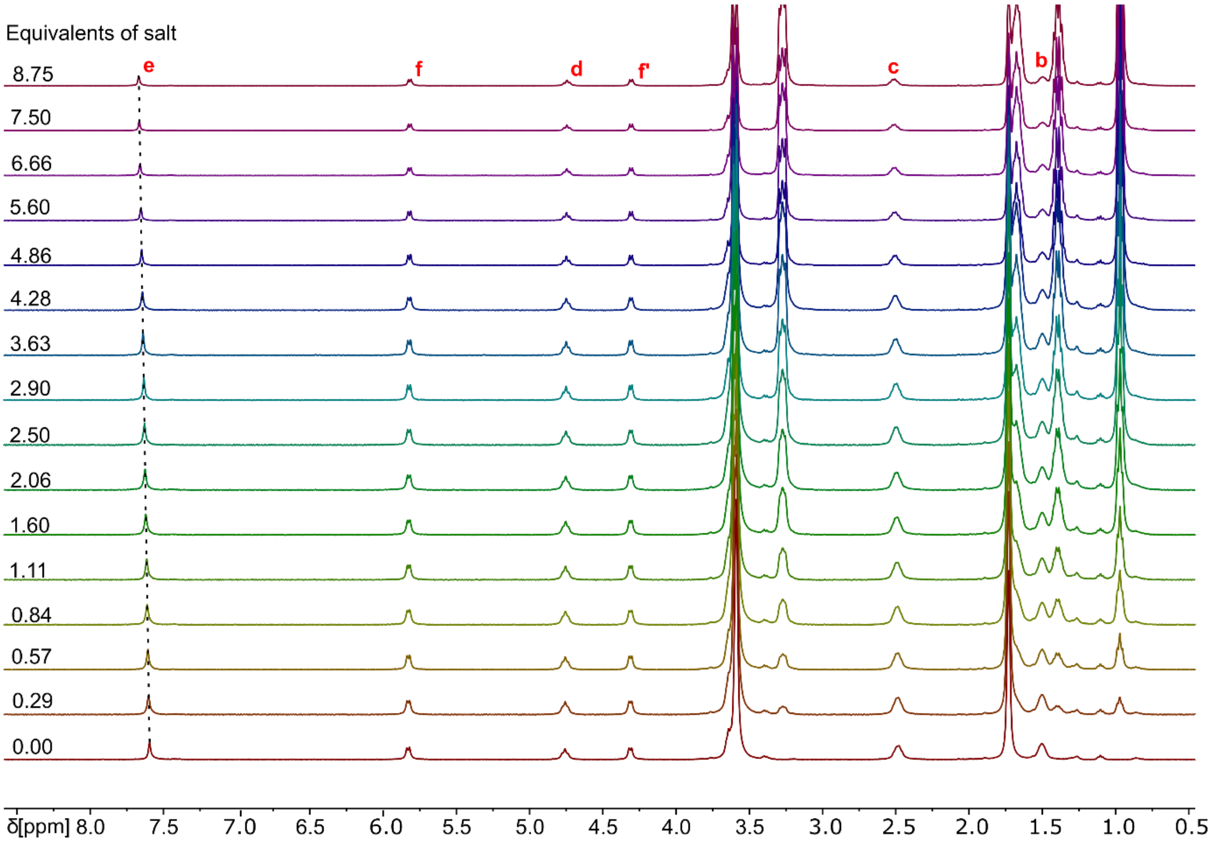

Figure S49. ^{1}H NMR spectra for the titration of **5** (0.0067 M) with **But_4NClO_4** in THF-d_8/10%D_2O at 298 K.

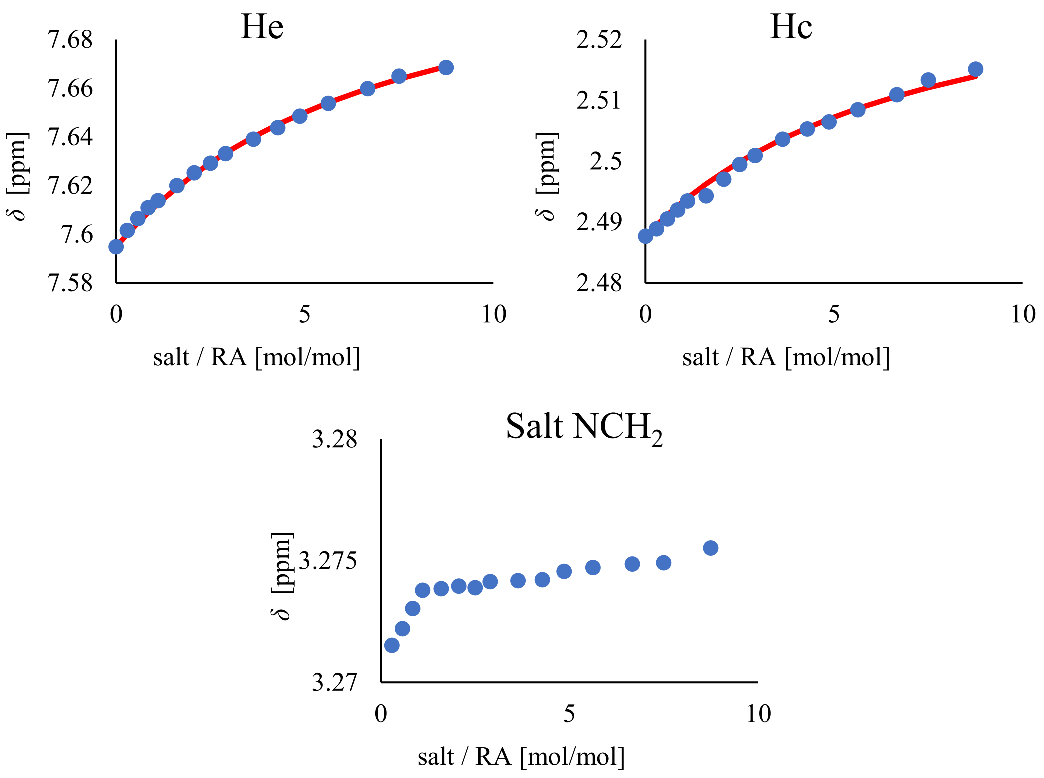

**K_{11}(M^{-1})**	**K error (%)**	**SSR**	**Datapoints fitted**	**Params fitted**
24.03	± 2.0854	2.8600e-5	32	3

http://app.supramolecular.org/bindfit/view/c0bc2fc7-54ff-4ebc-bf85-a240ce31b2e5

Figure S50. ^{1}H NMR experimental points and fitted curves for the titration of **5** (**RA**) **(**0.0067 M) with **But_4NClO_4**. (**salt**) ^{1}H NMR chemical shifts' changes for: (a) H_e; (b) H_c and (c) NCH_2 of the salt (298 K, THF-d_8/10% D_2O). Red lines correspond to fitted curves.

NMR titration of 6 with Pen_4NCl in THF-d_8 at 255 K

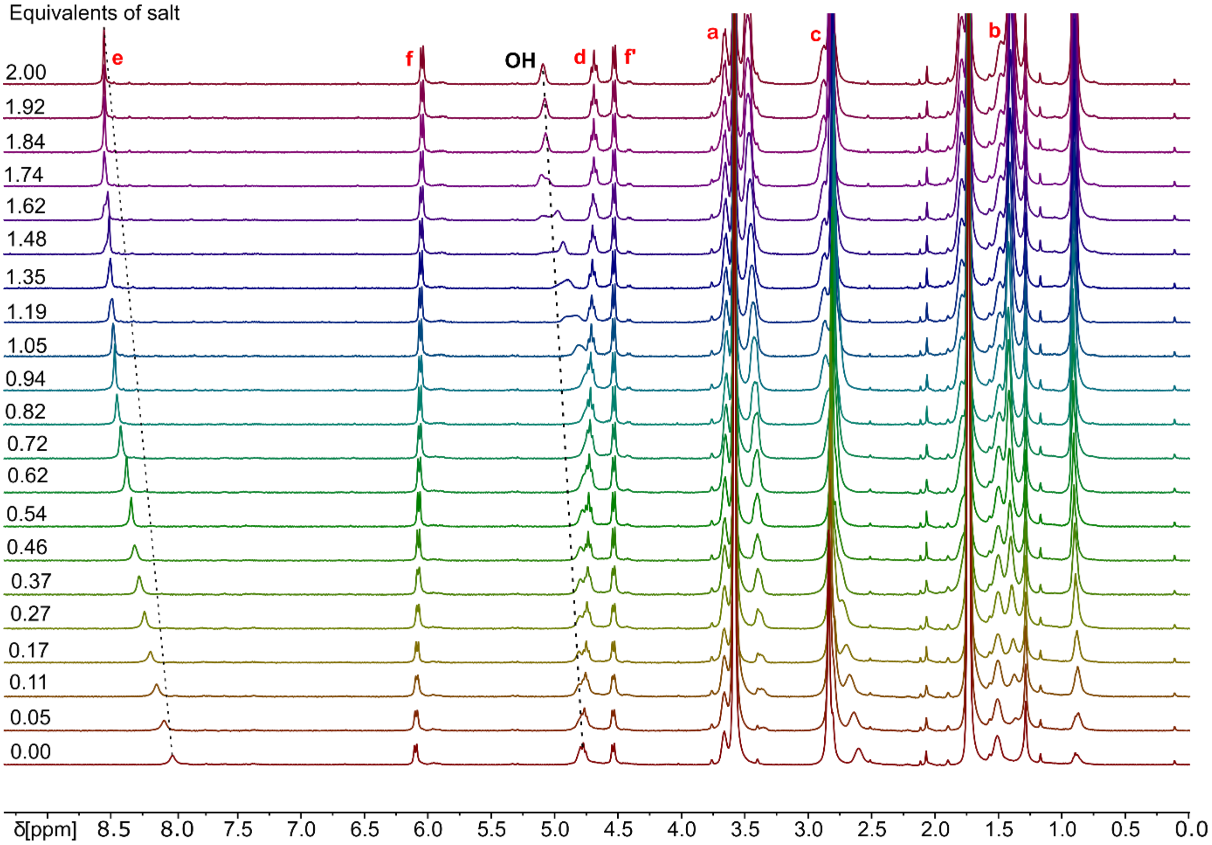

Figure S51. ^{1}H NMR spectra for the titration of **6** (0.0067 M) with **Pen_4NCl** in THF-d_8 at 255K

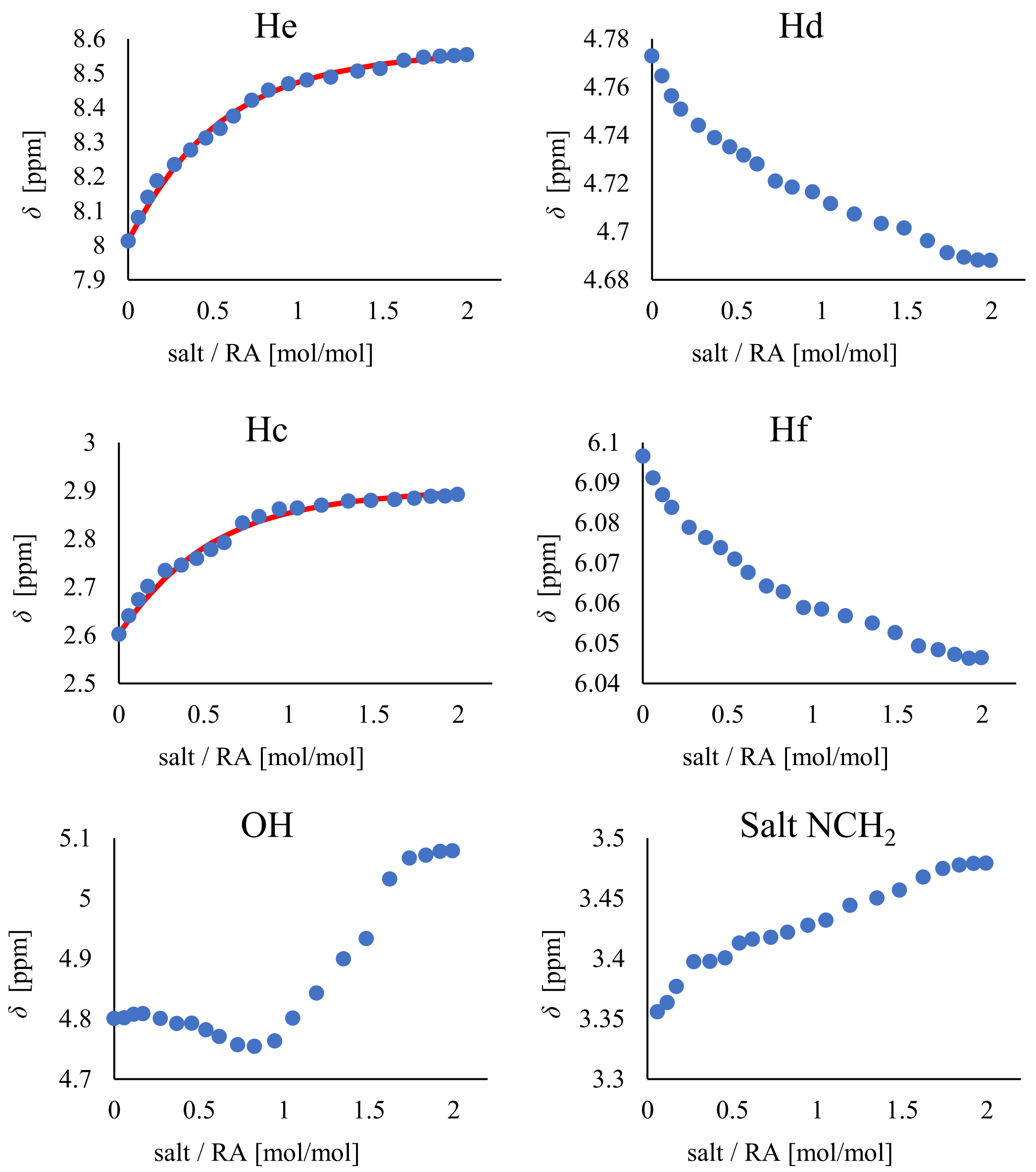

K₁₁(M⁻¹)	K error (%)	SSR	Datapoints fitted	Params fitted
357.52	± 2.2457	6.0346e-3	84	5

http://app.supramolecular.org/bindfit/view/79496e63-cb81-4337-bb62-3d14ca155b53

Figure S52. ^{1}H NMR experimental points and fitted curves for the titration of **6 (RA)** (0.0067 M) with **Pen$_4$NCl (salt)**. ^{1}H NMR chemical shifts' changes for: (a) H_e; (b) H_d; (c) H_c; (d) H_f; (e) H_{OH}; and (f) NCH_2 of the salt (255 K, THF-*d*$_8$). The above data has been fitted in a 2:1 binding model (Statistical/ L-BFGS-B). Red lines correspond to fitted curves.

NMR titration of 6 with Pen_4NBr in THF-d_8 at 255 K

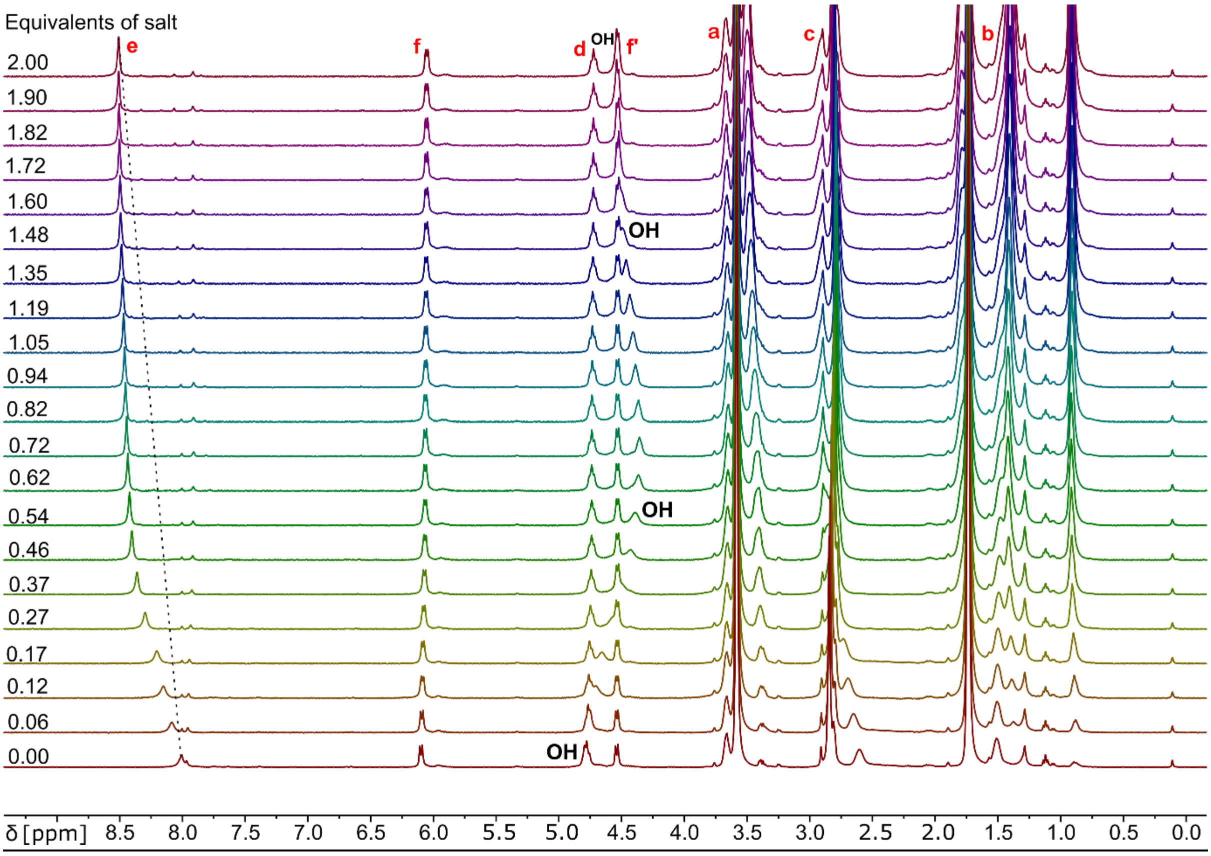

Figure S53. ^{1}H NMR spectra for the titration of **6** (0.0067 M) with **Pen_4NBr** in THF-d_8 at 255 K.

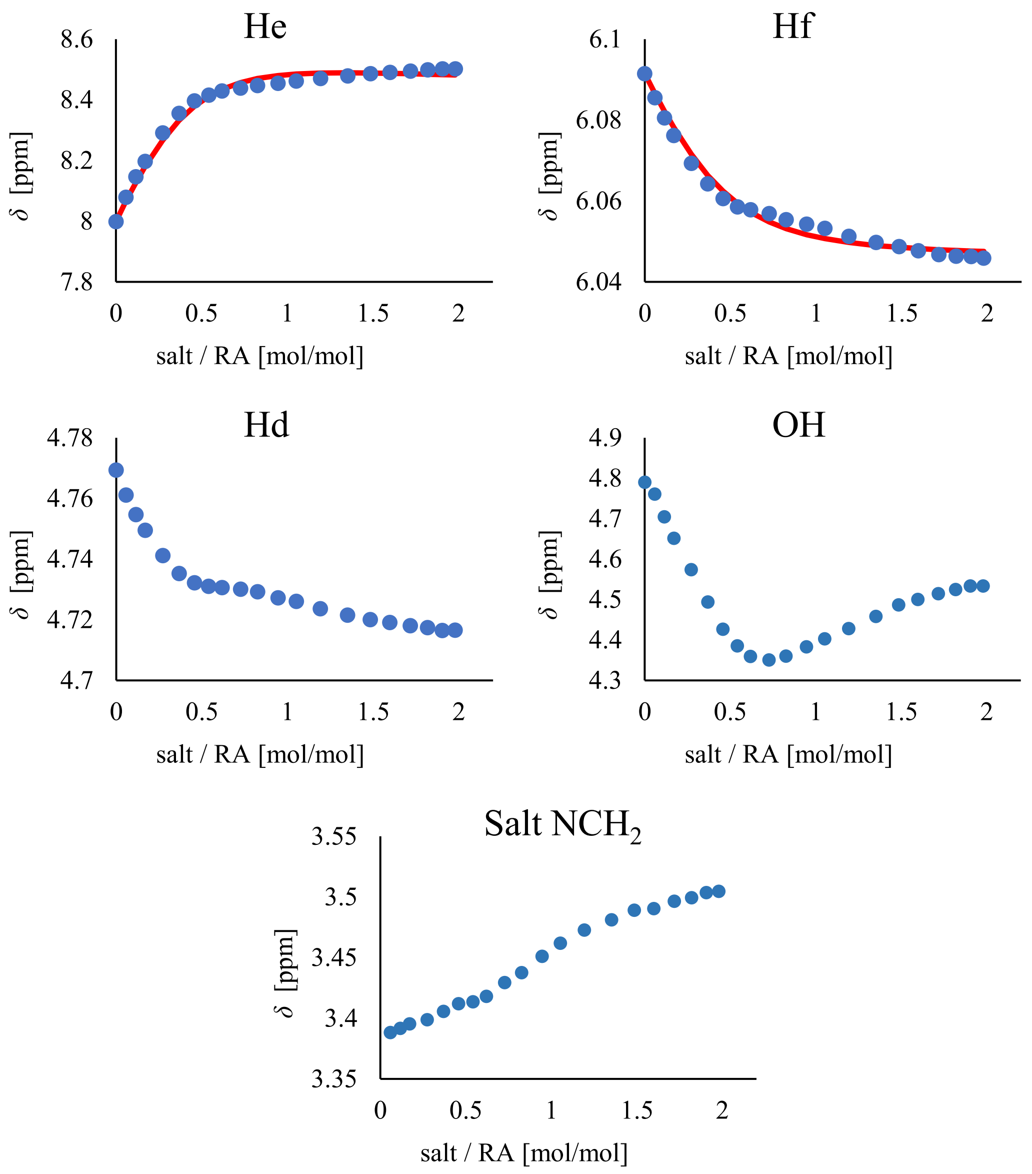

**K_{11}(M^{-1})**	**K error (%)**	**SSR**	**Datapoints fitted**	**Params fitted**
905.39	± 4.4671	6.1903e-3	63	7

http://app.supramolecular.org/bindfit/view/811ab7c3-60a4-41ce-8097-b628ee5ef447

Figure S54. ^{1}H NMR experimental points and fitted curves for the titration of **6 (RA)** (0.0067 M) with **Pen_4NBr (salt)**. ^{1}H NMR chemical shifts' changes for: (a) H_e; (b) H_f; (c) H_d; (d) H_{OH}; and (e) NCH_2 of the salt (255 K, THF-d_8). The above data has been fitted in a 2:1 binding model. (Non-cooperative/ Nelder-mead). Red lines correspond to fitted curves.

NMR titration of 6 with But_4NReO_4 in THF-d_8 at 255 K

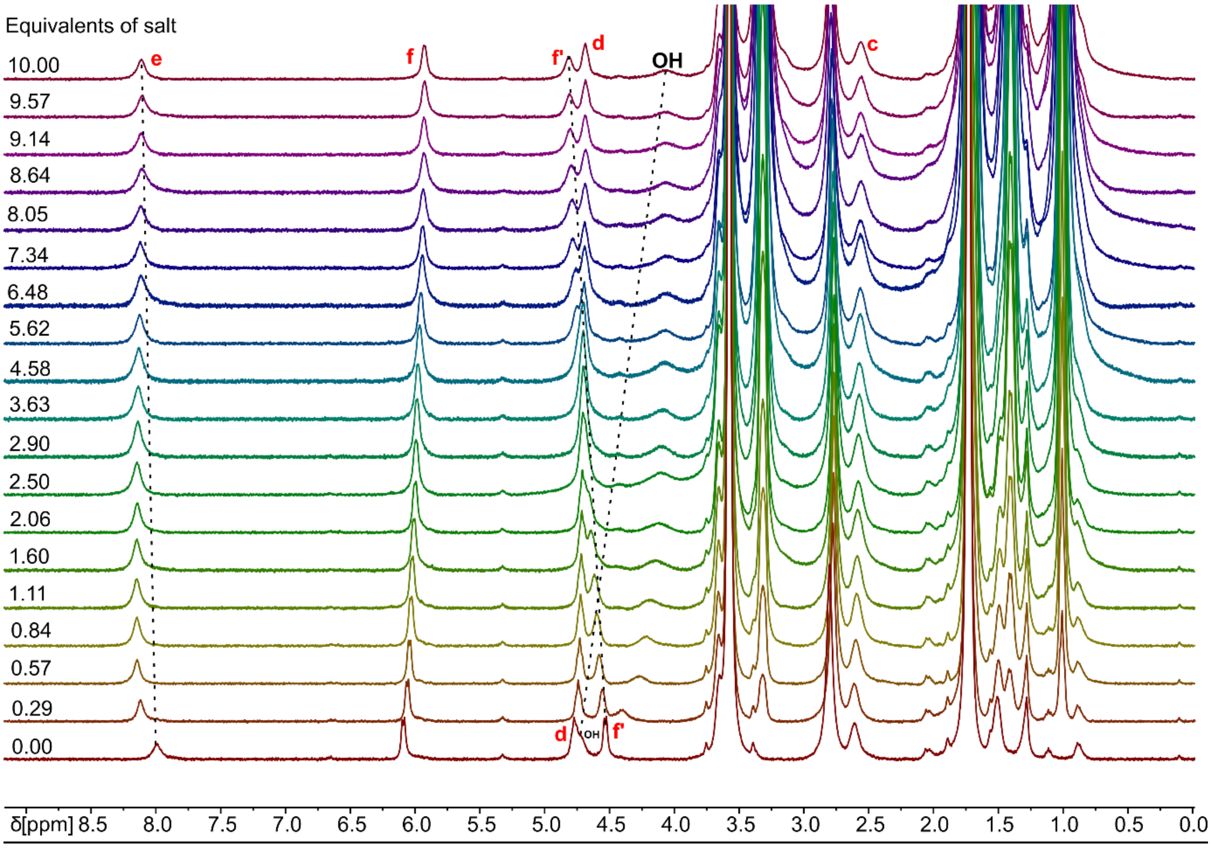

Figure S55.^{1}H NMR spectra for the titration of **6** (0.0067 M) with **But_4NReO_4** in THF-d_8 at 255 K.

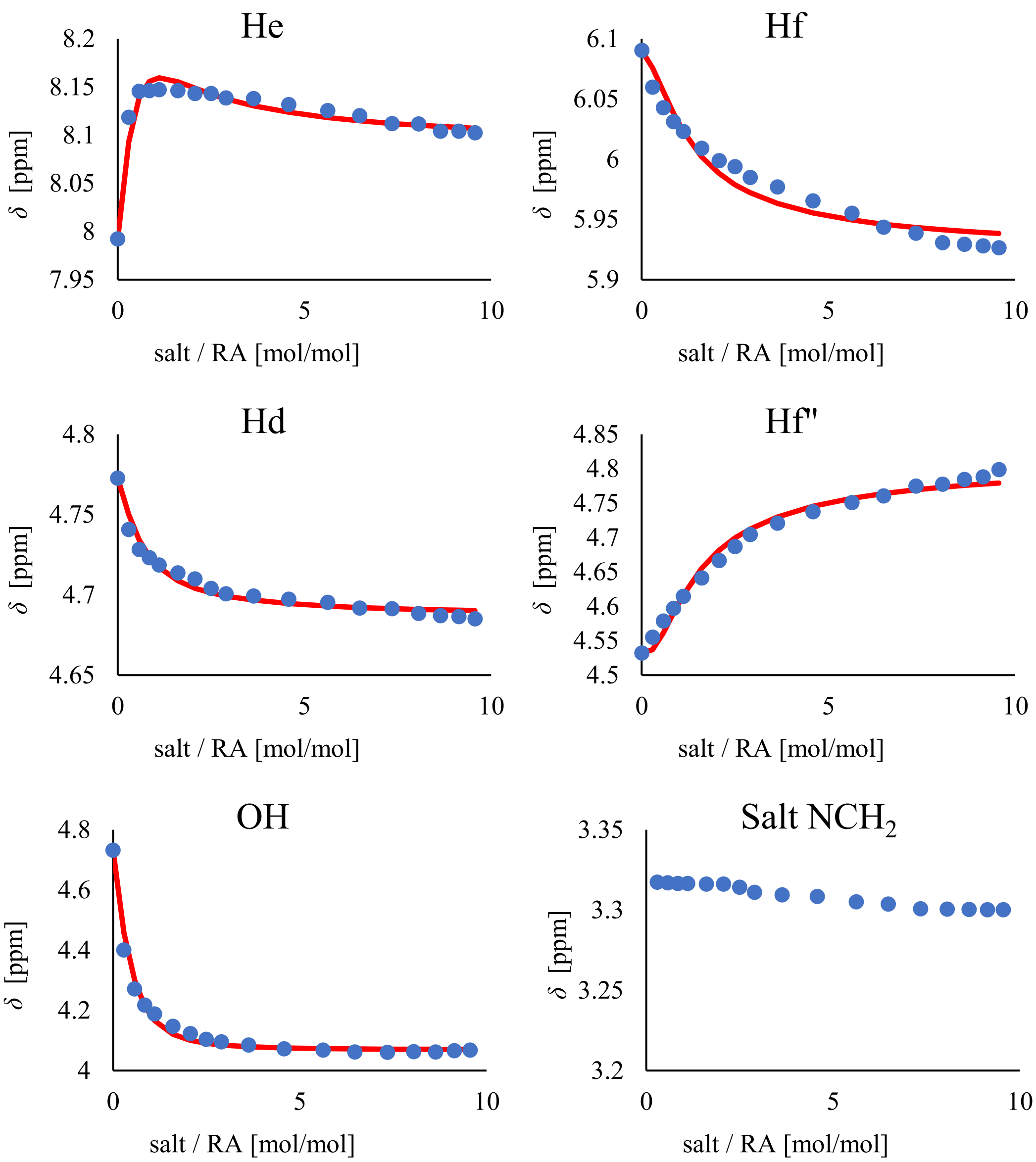

K_{11}(M^{-1})	K error (%)	SSR	Datapoints fitted	Params fitted
468.80	± 5.3485	1.2143e-2	90	11

http://app.supramolecular.org/bindfit/view/2c747b17-dbe7-44ae-9599-945a8b5d3983

Figure S56. ^{1}H NMR experimental points and fitted curves for the titration of **6 (RA)** (0.0067 M) with **But_4NReO_4 (salt)** ^{1}H NMR chemical shifts' changes for: (a) H_e; (b) $H_{f;}$ (c) H_d; (d) $H_{f'}$ (e) H_{OH}; and (f) NCH_2 of the salt (255 K, THF-d_8). The above data has been fitted in a 2:1 binding model. (Non-cooperative/ Nelder-mead). Red lines correspond to fitted curves.

NMR titration of 6 with But_4NNO_3 in THF-d_8 at 255 K

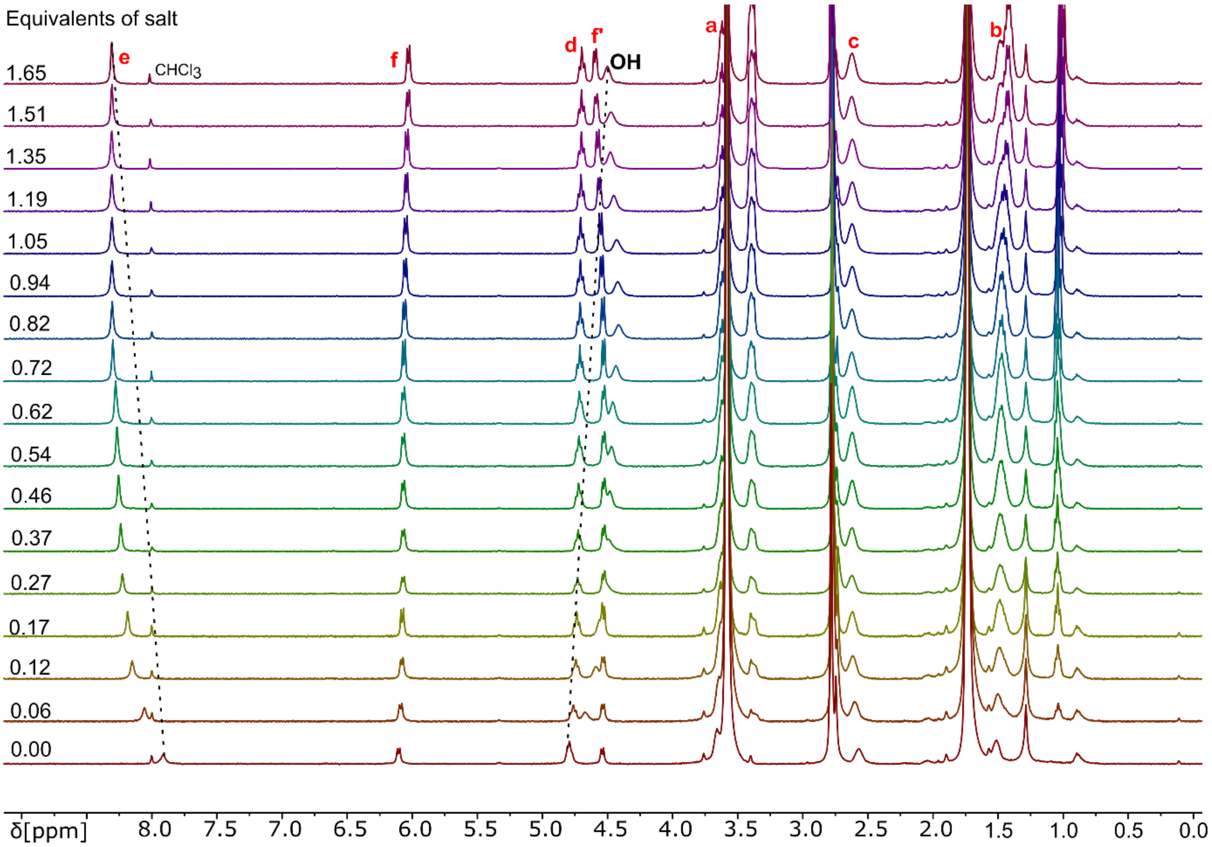

Figure S57. ^{1}H NMR spectra for the titration of **6** (0.0067 M) with **But_4NNO_3** in THF-d_8 at 255 K.

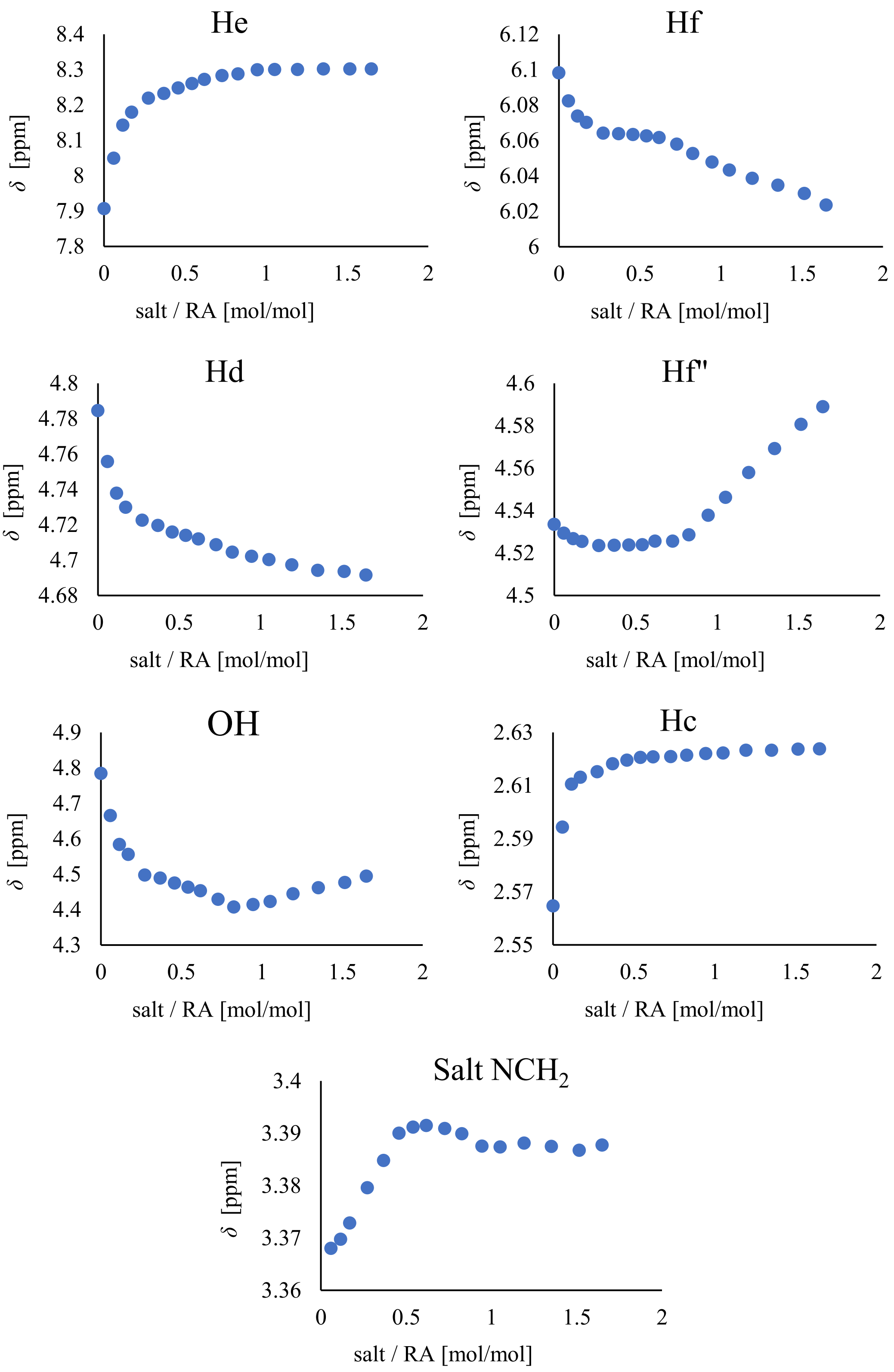

Figure S58. ^{1}H NMR experimental points for the titration of **6 (RA)** (0.0067 M) with **But_4NNO_3 (salt)** ^{1}H NMR chemical shifts' changes for: (a) H_e; (b) H_f; (c) H_d; (d) $H_{f'}$ (e)H_c (f) H_{OH}; and (g) NCH_2 of the salt (255 K, THF-d_8). The data could not be fitted reasonably due to high error.

NMR titration of 6 with Pen_4NCl in THF-d_8/10% D_2O

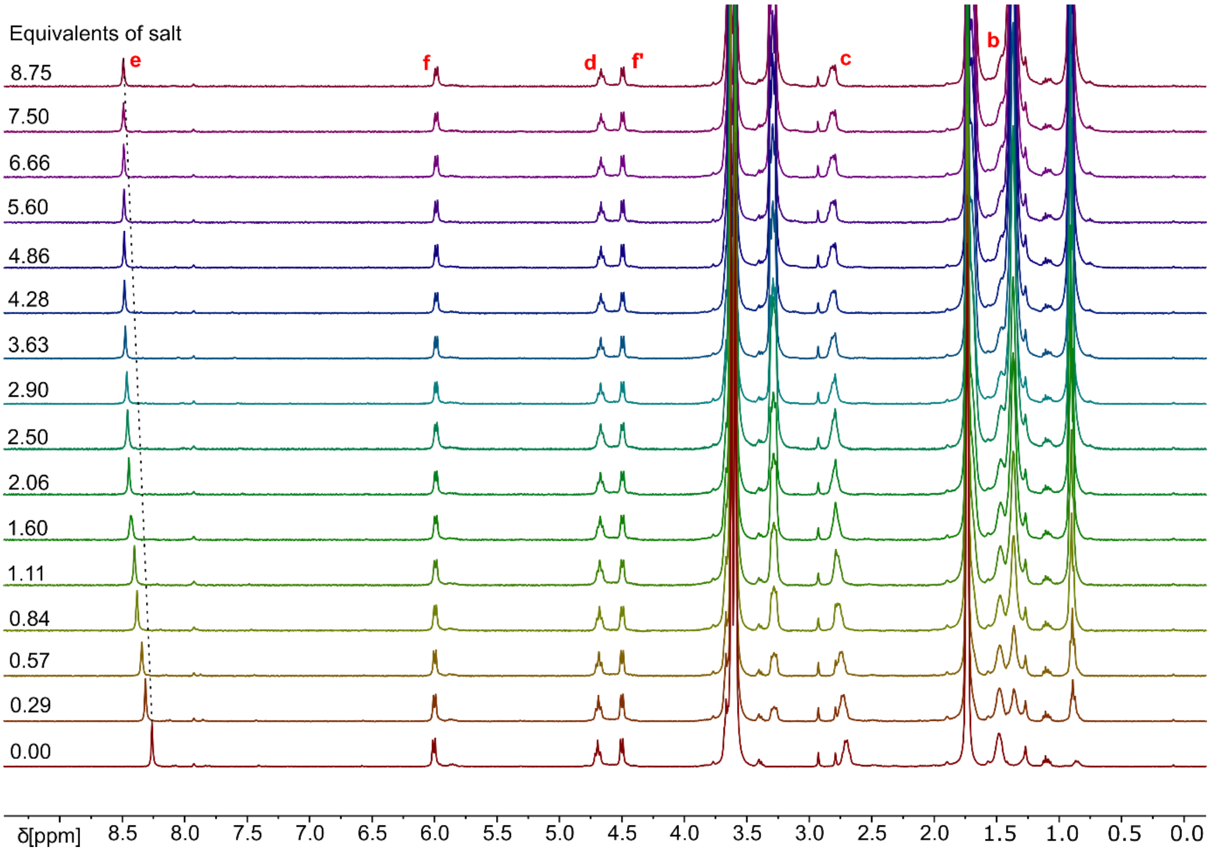

Figure S59. ^{1}H NMR spectra for the titration of **6** (0.0067 M) with **Pen_4NCl** in THF-d_8/ 10% D_2O at 298 K.

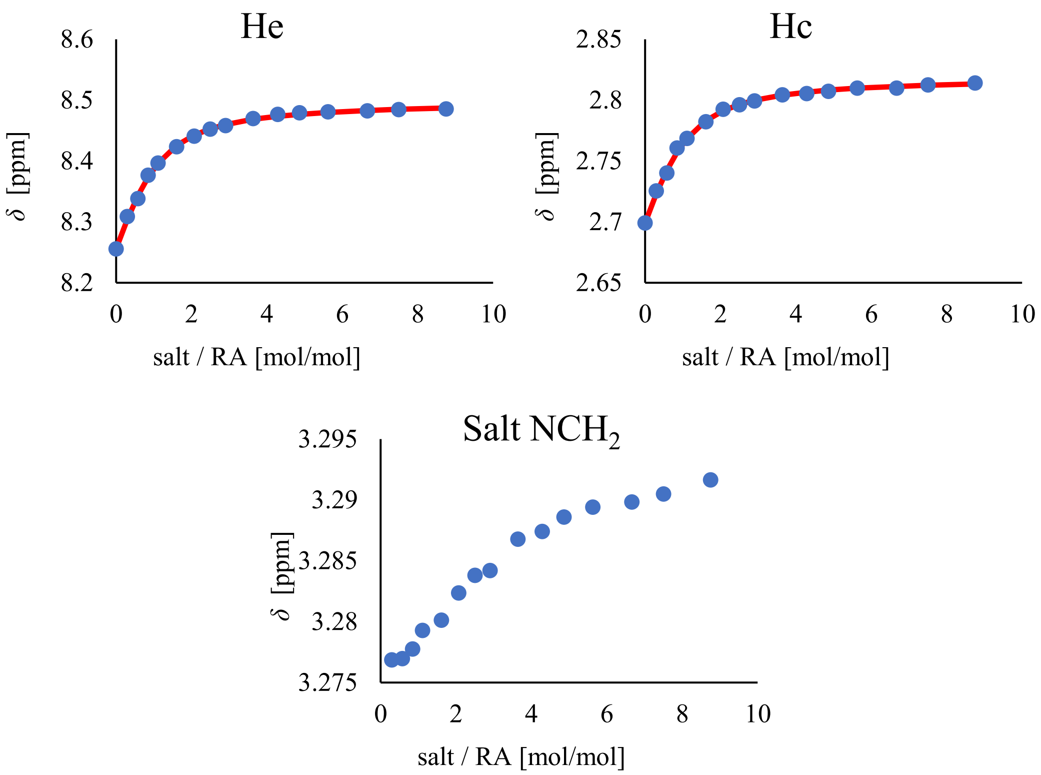

http://app.supramolecular.org/bindfit/view/c8806538-567f-43a5-a090-d6582772436b

**K_{11}(M^{-1})**	**K error (%)**	**SSR**	**Datapoints fitted**	**Params fitted**
384.98	± 3.5974	1.2283e-4	32	3

Figure S60. ^{1}H NMR experimental points and fitted curves for the titration of **6 (RA)** (0.0067 M) with **Pen_4NCl. (salt)** ^{1}H NMR chemical shifts' changes for: (a) H_e; (b) H_c; and (c) NCH_2 of the salt (298 K, THF-d_8/10% D_2O). Red lines correspond to fitted curves.

NMR titration of 6 with But_4NNO_3 in THF-d_8/10% D_2O

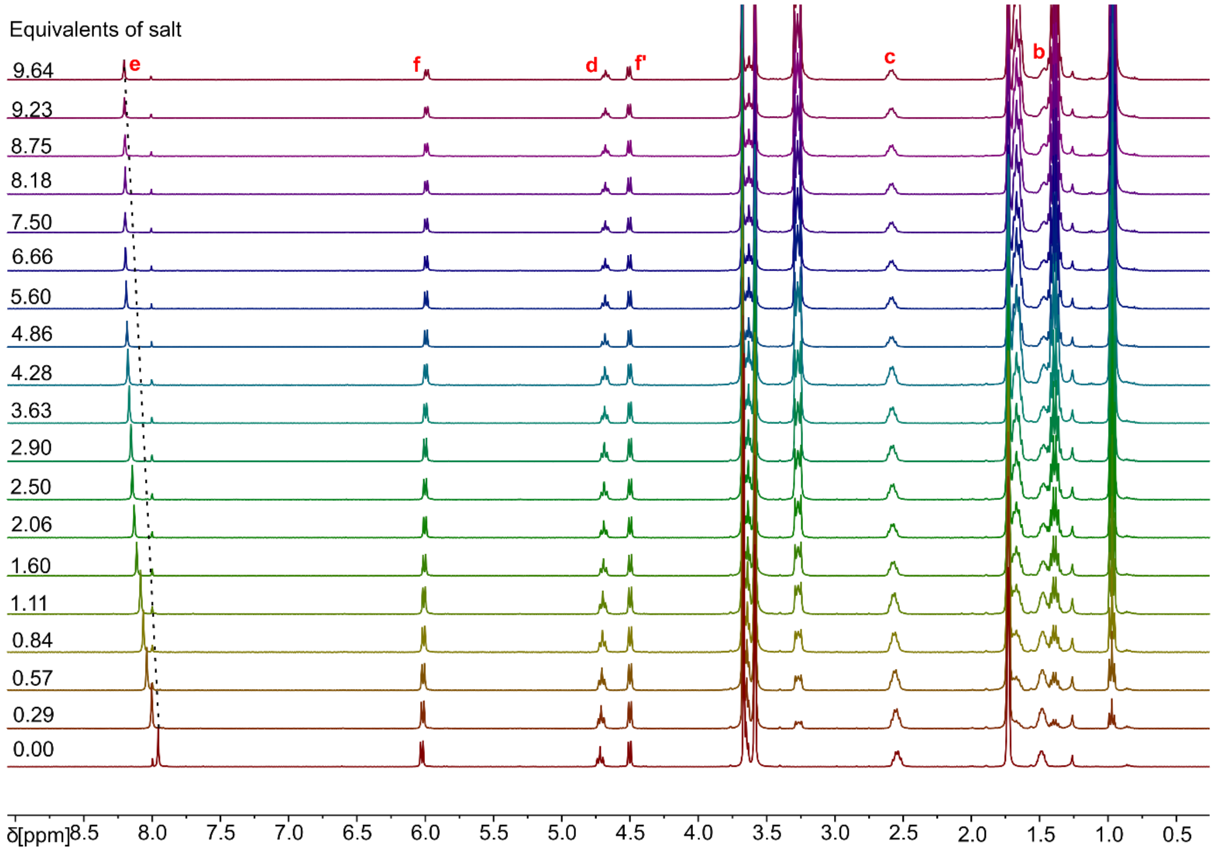

Figure S61. ^{1}H NMR spectra for the titration of **6** (0.0067 M) with **But_4NNO_3** in THF-d_8/10%D_2O at 298 K.

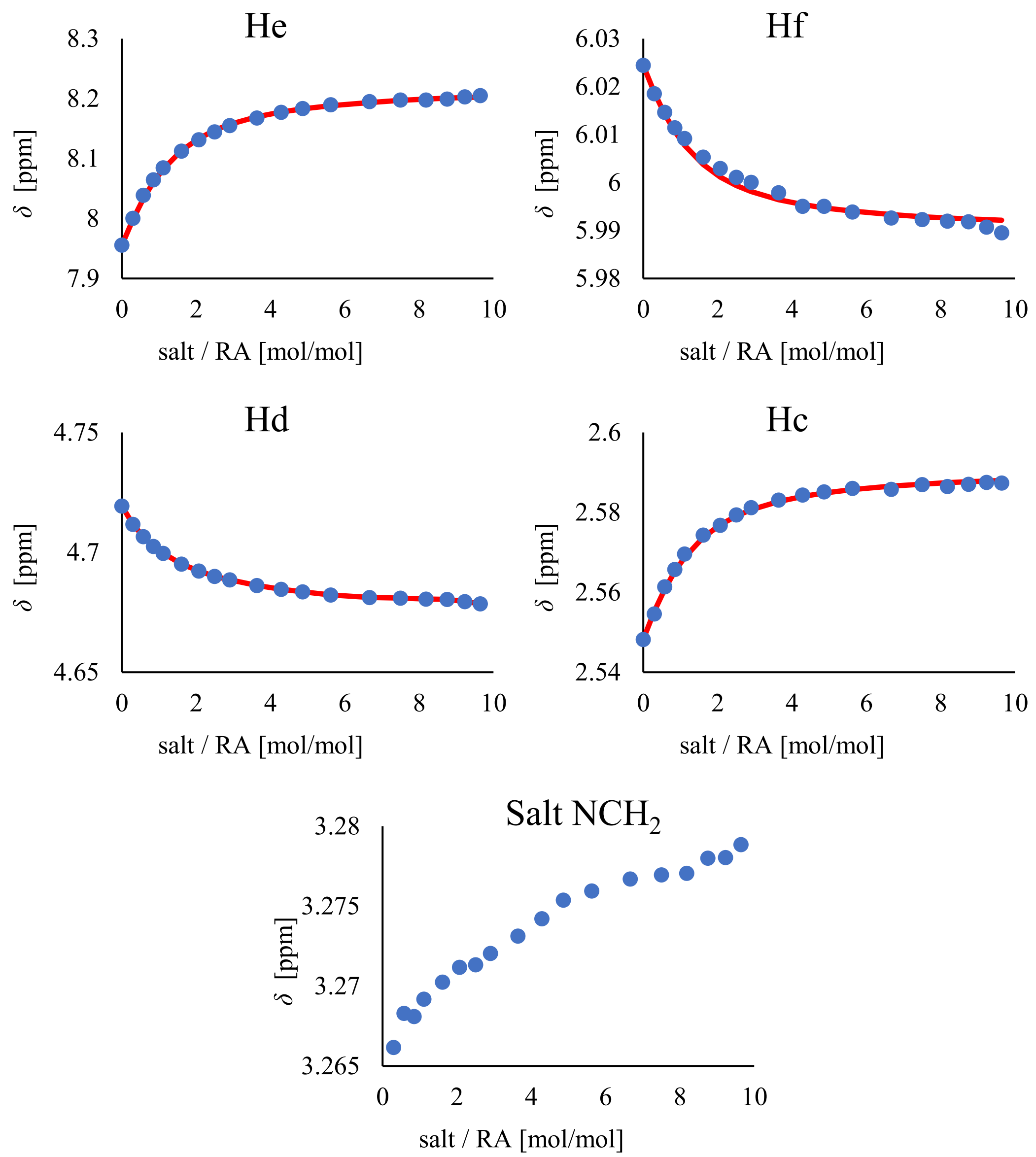

K_{11}(M^{-1})	K error (%)	SSR	Datapoints fitted	Params fitted
215.10	± 1.7813	1.3481e-4	76	5

http://app.supramolecular.org/bindfit/view/d4bc1068-471f-420b-a173-150c78e8118e

Figure S62. ^{1}H NMR experimental points and fitted curves for the titration of **6 (RA)** (0.0067 M) with **But_4NNO_3 (salt)** ^{1}H NMR chemical shifts' changes for: (a) H_e; (b) H_f; (c) H_d; (d) H_c and (e) NCH_2 of the salt (298 K, THF-d_8/10% D_2O). Red lines correspond to fitted curves.

NMR titration of 6 with But_4NHSO_4 in THF-d_8/10% D_2O

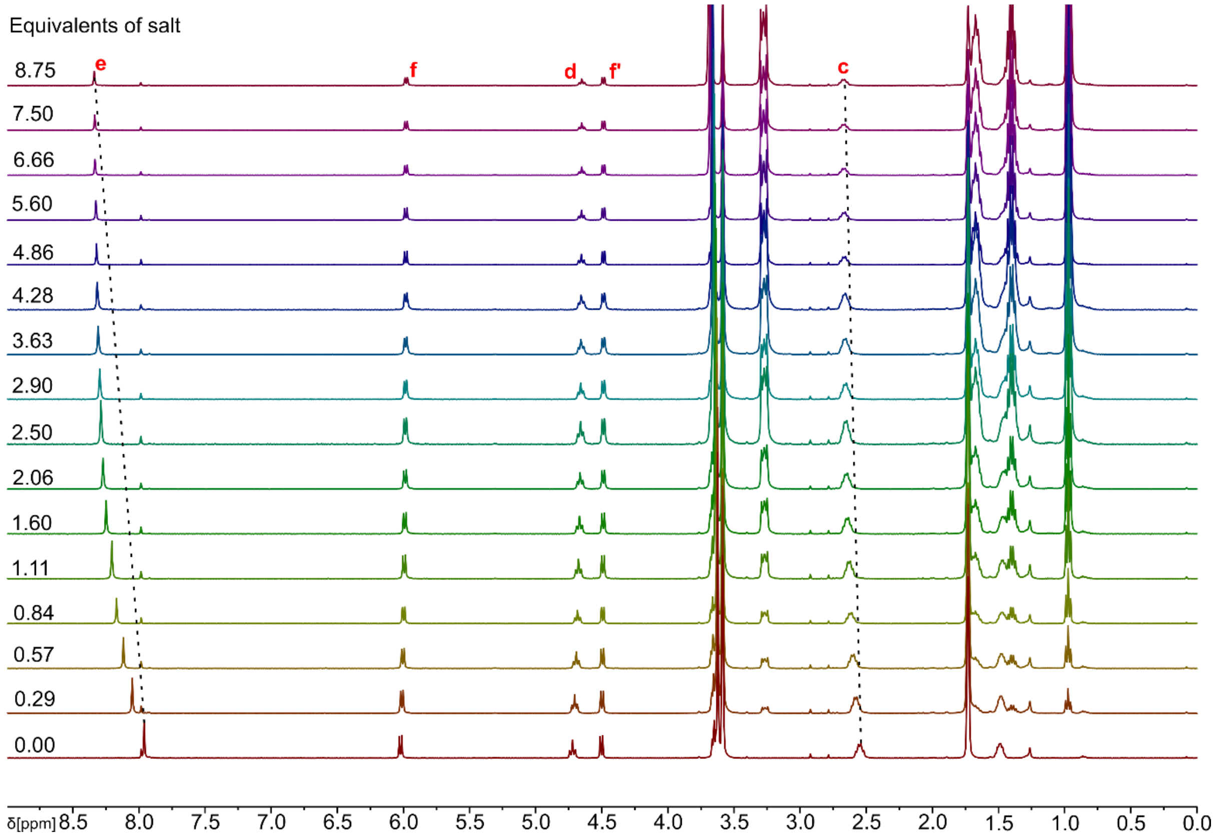

Figure S63. ^{1}H NMR spectra for the titration of **6** (0.0067 M) with **But_4NHSO_4** in THF-d_8/10% D_2O at 298 K.

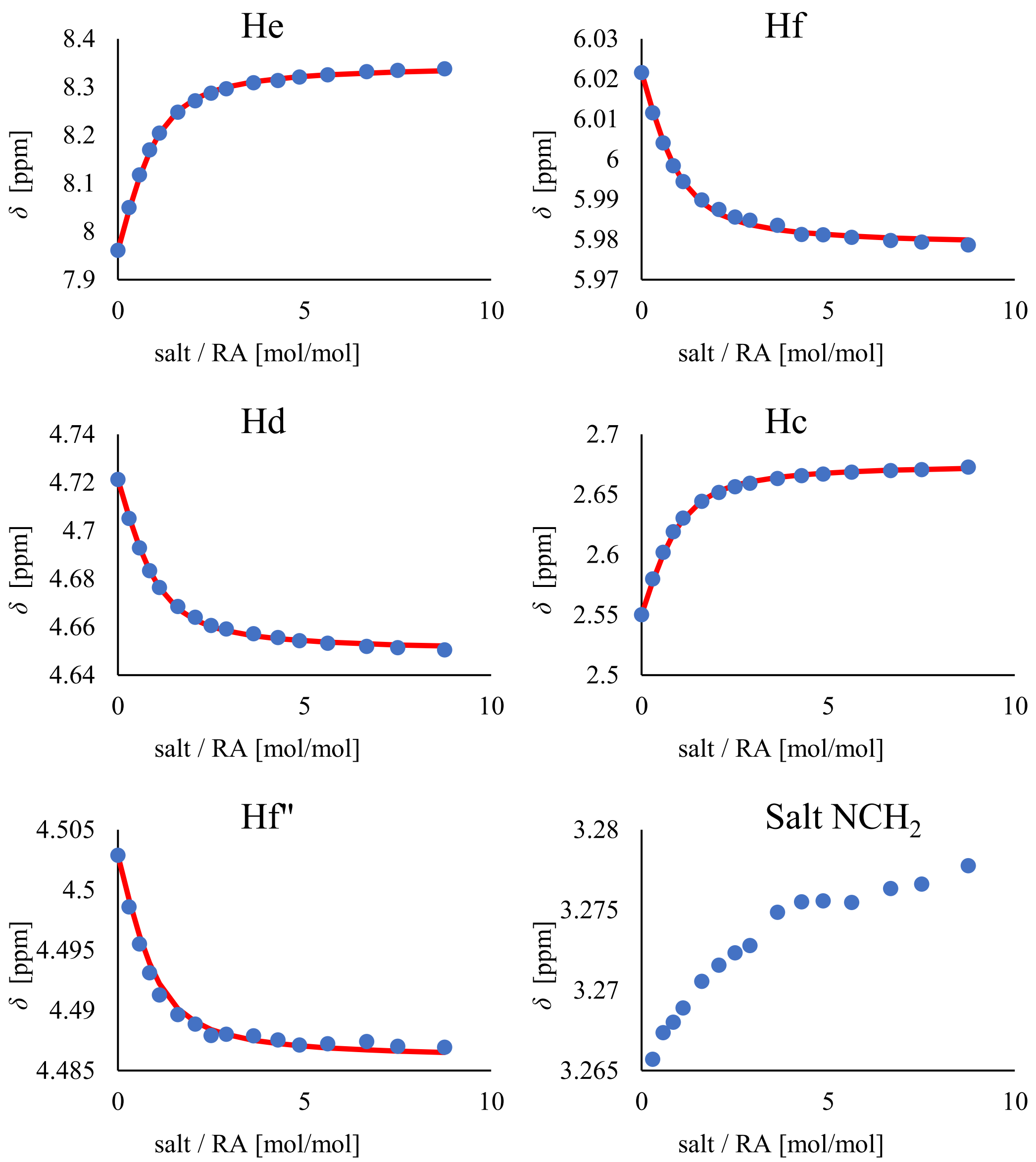

K_{11}(M^{-1})	K error (%)	SSR	Datapoints fitted	Params fitted
523.67	± 2.2181	2.3106e-4	80	6

http://app.supramolecular.org/bindfit/view/b33124ca-153b-49b2-a6e8-c97b8a30c2ae

Figure S64. ^{1}H NMR experimental points and fitted curves for the titration of **6 (RA)** (0.0067 M) with **But$_4$NHSO$_4$ (salt)** ^{1}H NMR chemical shifts' changes for: (a) H$_e$; (b) H$_f$; (c) H$_d$; (d) H$_c$; (e) H$_{f'}$ and (f) NCH$_2$ of the salt (298 K, THF-*d*$_8$/10% D$_2$O). Red lines correspond to fitted curves.

NMR titration of 6 with $But_4NH_2PO_4$ in THF-d_8/10% D_2O

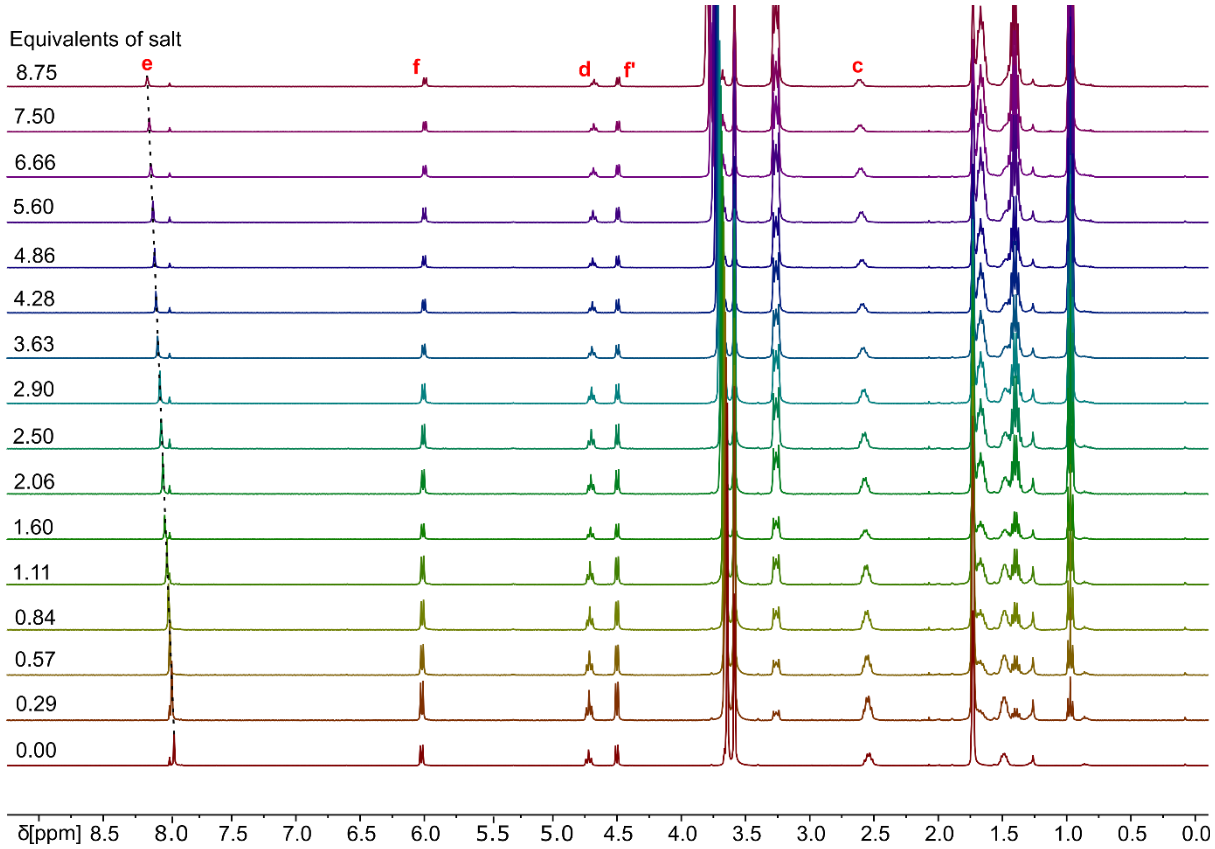

Figure S65. ^1H NMR spectra for the titration of **6** (0.0067 M) with **$But_4NH_2PO_4$** in THF-d_8/10% D_2O at 298 K.

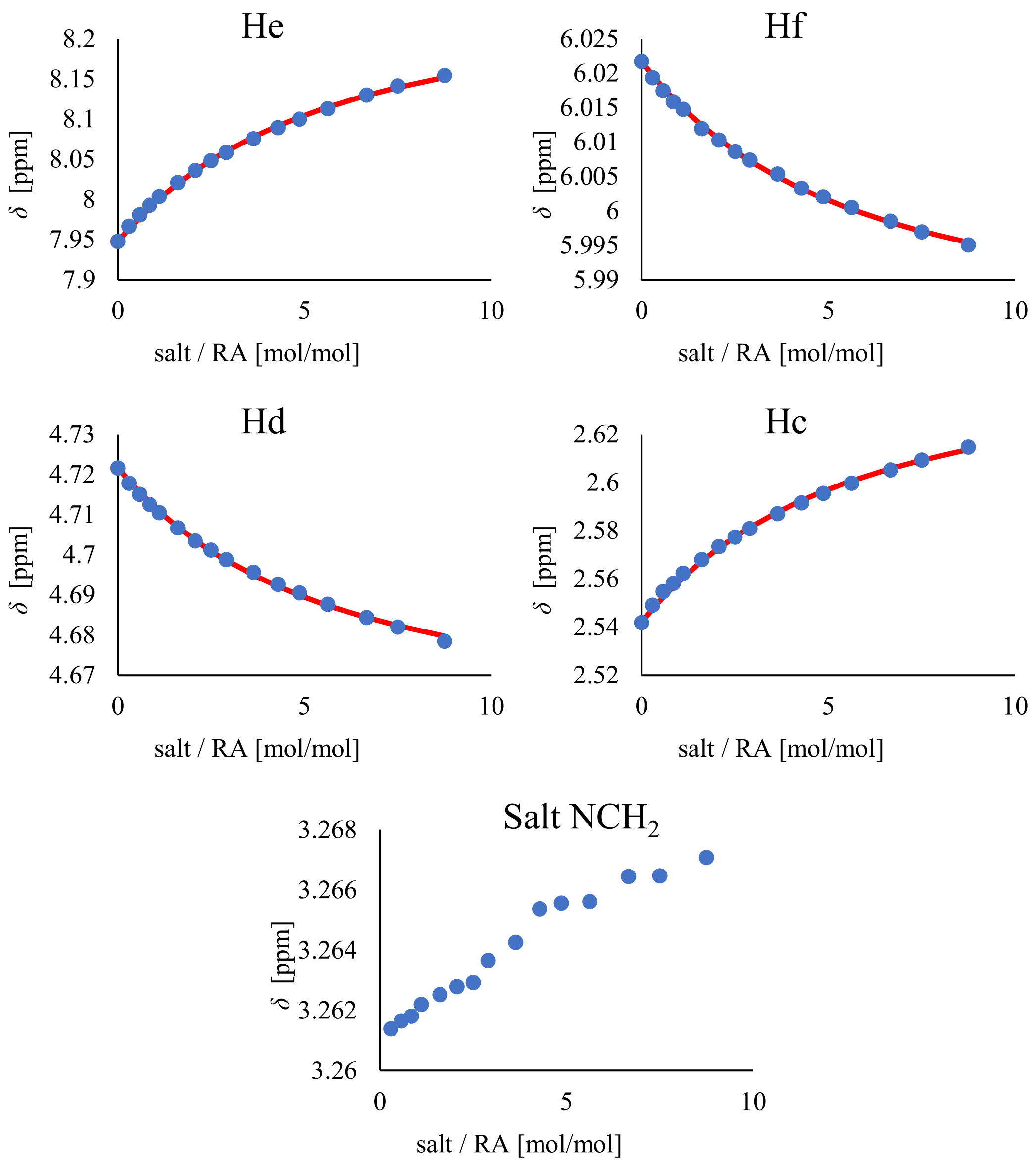

K_{11}(M^{-1})	K error (%)	SSR	Datapoints fitted	Params fitted
30.49	± 1.0129	1.0285e-4	64	5

http://app.supramolecular.org/bindfit/view/3c9b9a40-0af9-43c1-a353-d818643861b6

Figure S66. ^{1}H NMR experimental points and fitted curves for the titration of **6 (RA)** (0.0067 M) **with But$_4$NH$_2$PO$_4$ (salt)** ^{1}H NMR chemical shifts' changes for: (a) H_e; (b) H_f; (c) H_d; (d) H_c; and (e) NCH_2 of the salt (298 K, THF-d_8/10% D_2O). Red lines correspond to fitted curves.

NMR titration of 6 with But_4NClO_4 in THF-d_8/10% D_2O

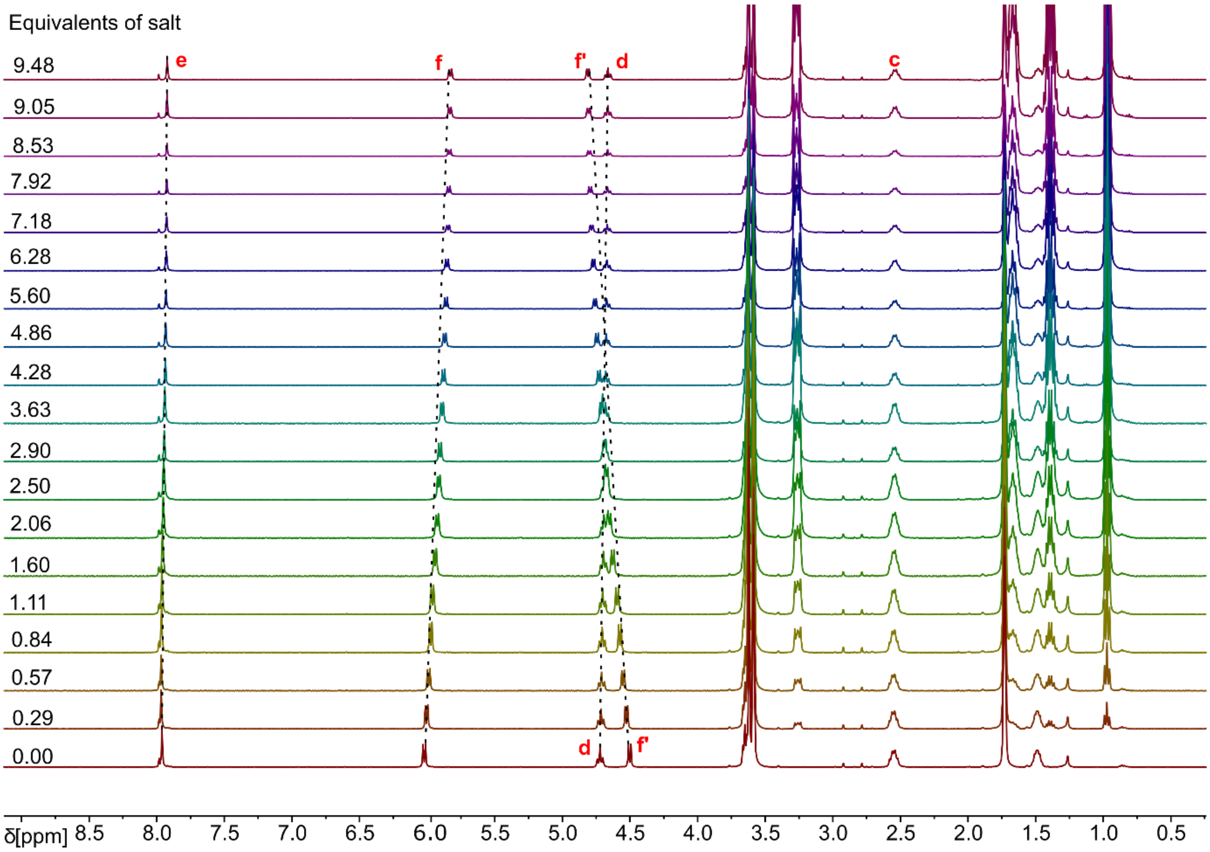

Figure S67. ^{1}H NMR spectra for the titration of **6** (0.0067 M) with **But_4NClO_4** in THF-d_8/10% D_2O at 298 K.

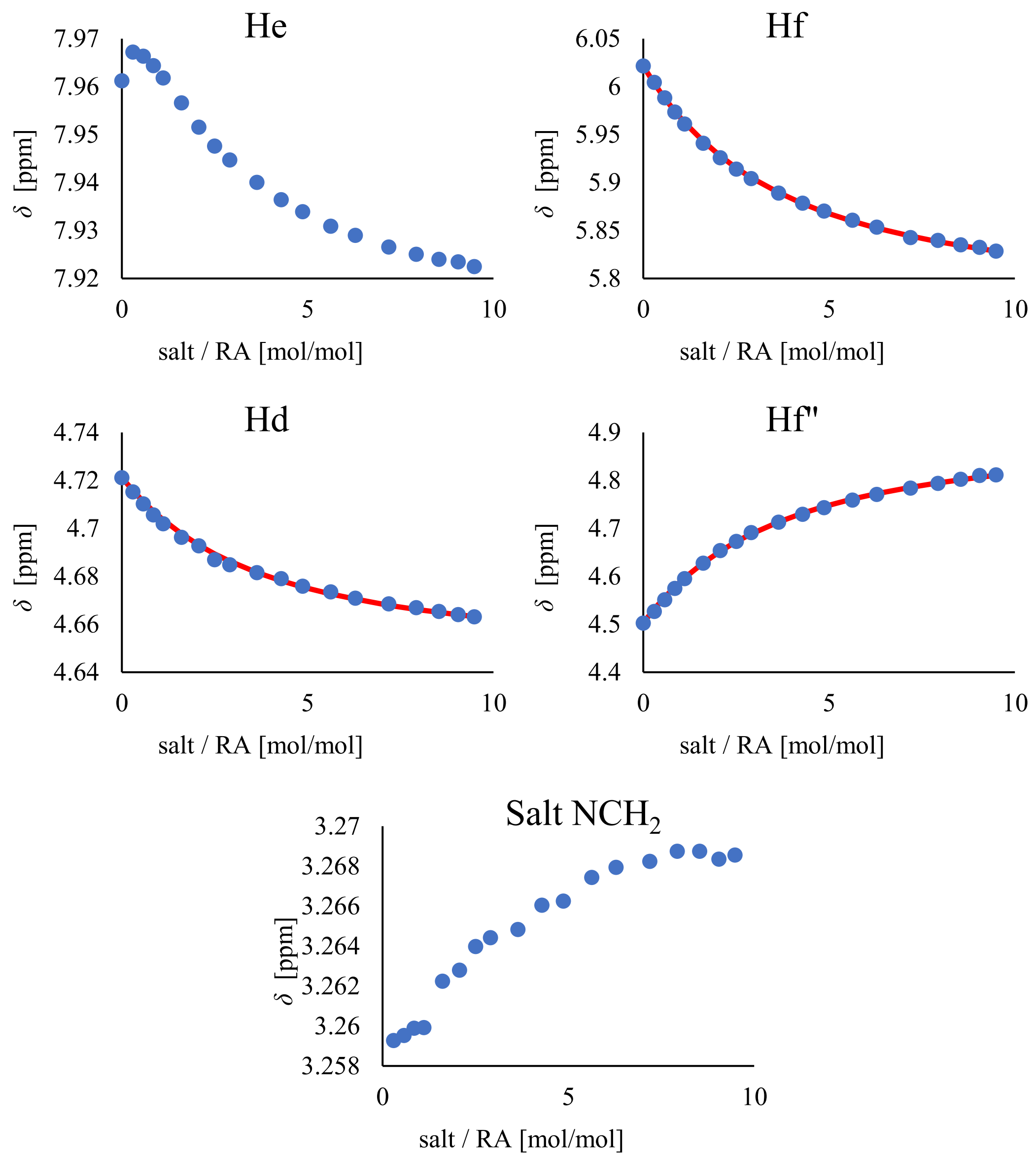

K_{11}(M^{-1})	K error (%)	SSR	Datapoints fitted	Params fitted
49.49	± 0.8173	1.4217e-4	57	4

http://app.supramolecular.org/bindfit/view/1b9af8d6-0167-494f-83cd-a2510b34ff7c

Figure S68. ^{1}H NMR experimental points and fitted curves for the titration of **6 (RA)** (0.0067 M) with **But$_4$NClO$_4$ (salt)**. ^{1}H NMR chemical shifts' changes for: (a) H_e; (b) H_f; (c) H_d; (d) $H_{f''}$; and (e) NCH_2 of the salt (298 K, THF-d_8/10% D_2O). Red lines correspond to fitted curves.

NMR titration of 6 with But_4NReO_4 in THF-d_8/10% D_2O

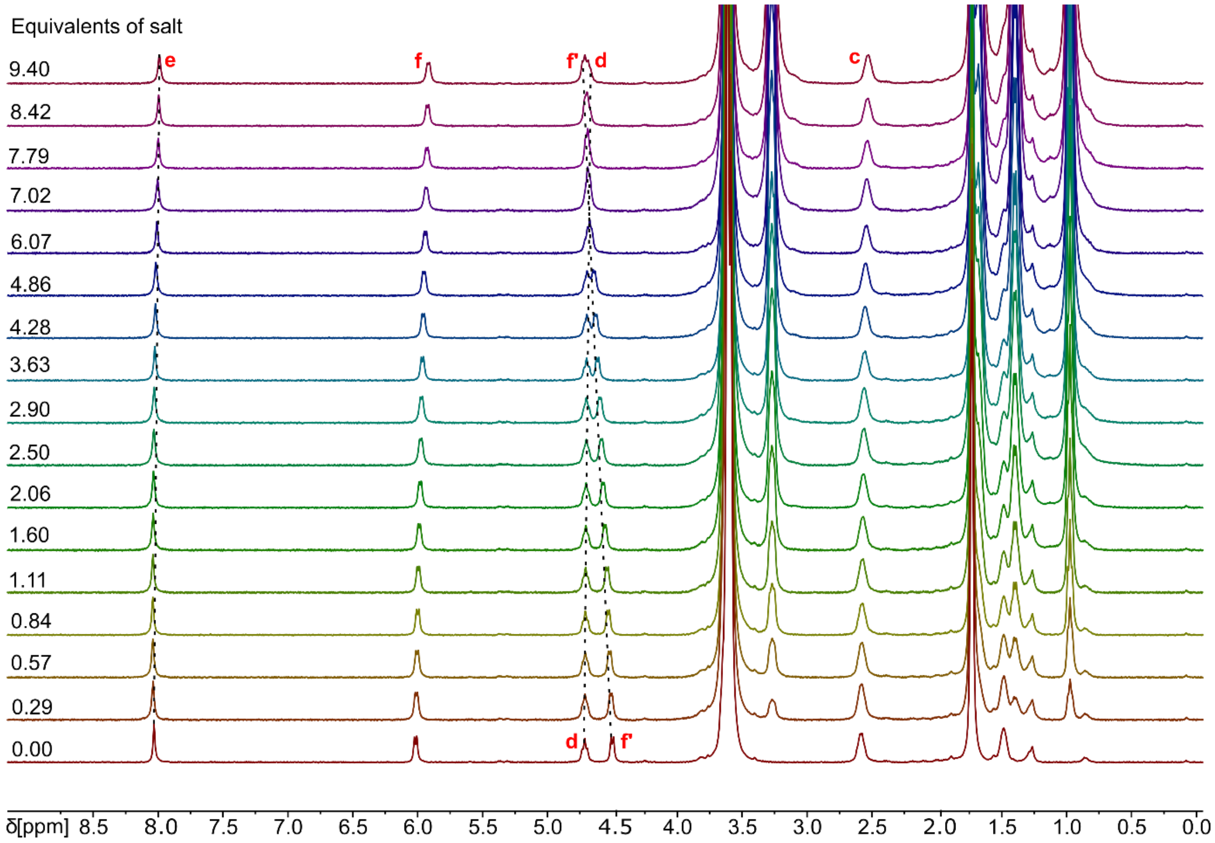

Figure S69. ^{1}H NMR spectra for the titration of **6** (0.0067 M) with $\mathbf{But_4NReO_4}$ in THF-d_8/10% D_2O at 298 K.

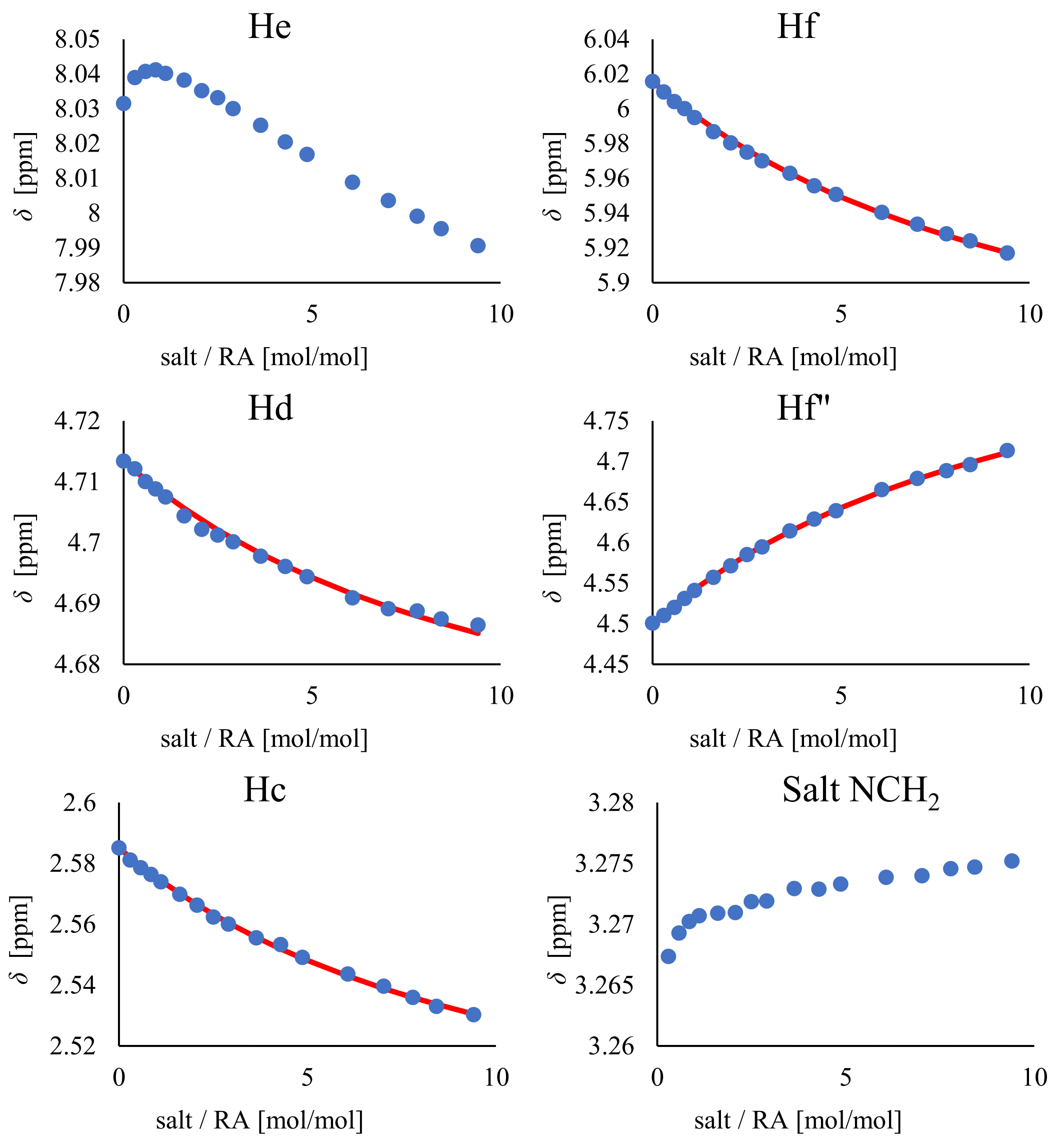

K_{11}(M^{-1})	K error (%)	SSR	Datapoints fitted	Params fitted
15.12	± 0.6421	7.7839e-5	68	5

http://app.supramolecular.org/bindfit/view/8b683154-d1a8-4e53-9162-09c840b38b6b

Figure S70. ^{1}H NMR experimental points and fitted curves for the titration of **6** **(RA)** (0.0067 M) with **But_4NReO_4 (salt).** ^{1}H NMR chemical shifts' changes for: (a) H_e; (b) H_f; (c) H_d; (d) $H_{f''}$; (e) H_c; and (f) NCH_2 of the salt (298 K, THF-d_8/10% D_2O). Red lines correspond to fitted curves.

NMR titration of 7 with Pen_4NCl in THF-d_8/10% D_2O

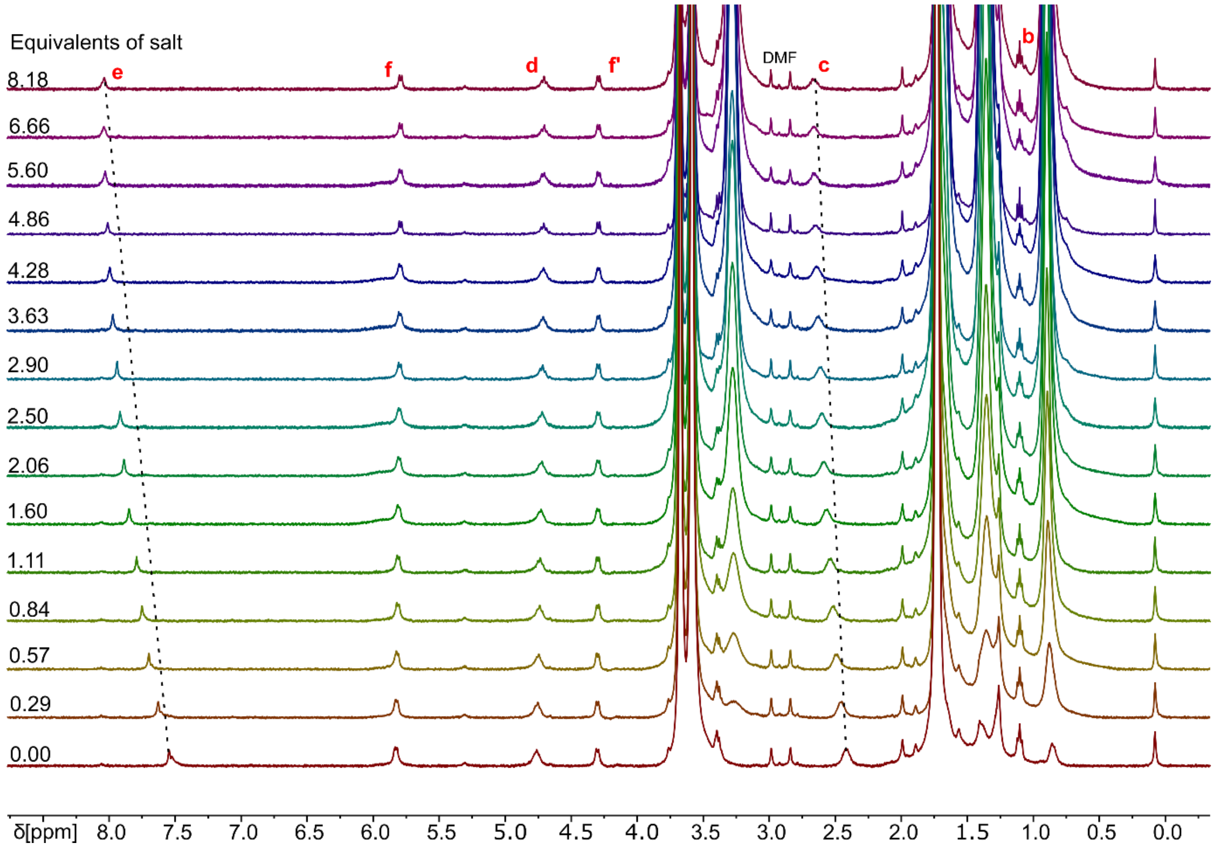

Figure S71. ^{1}H NMR spectra for the titration of **7** (0.0067 M) with **Pen_4NCl** in THF-d_8/10% D_2O at 298 K.

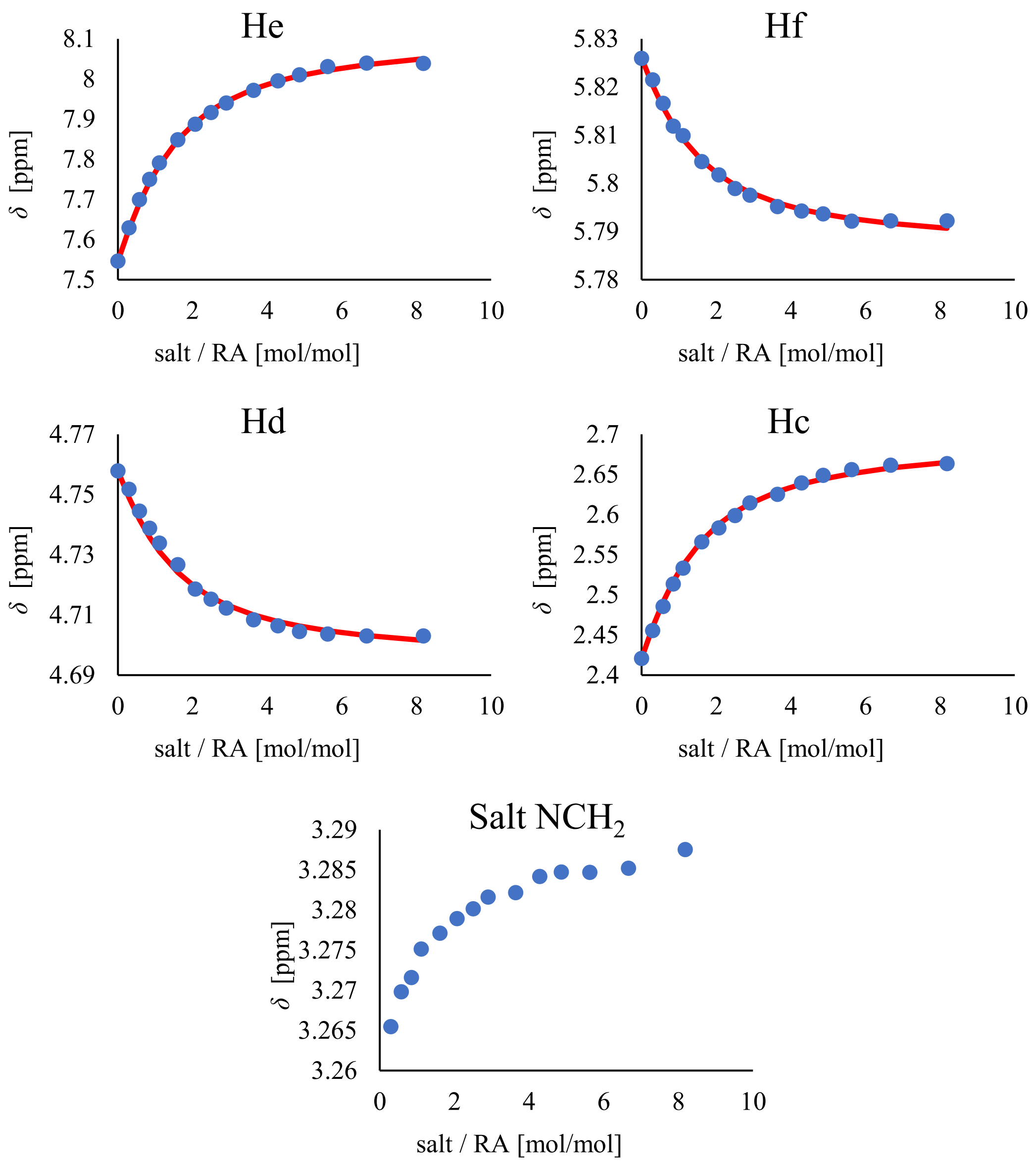

K_{11}(M^{-1})	K error (%)	SSR	Datapoints fitted	Params fitted
157.617704	1.87631062	0.00068592	60	5

http://app.supramolecular.org/bindfit/view/1fff6385-1029-4216-84d0-971380ae1518

Figure S72. ^{1}H NMR experimental points and fitted curves for the titration of **7 (RA)** (0.0067 M) with **Pen$_4$NCl (salt)** ^{1}H NMR chemical shifts' changes for: (a) H_e; (b) H_f; (c) H_d; (d) H_c; and (e) NCH_2 of the salt (298 K, THF-d_8/10% D_2O). Red lines correspond to fitted curves.

NMR titration of 7 with But_4NHSO_4 in THF-d_8/10% D_2O

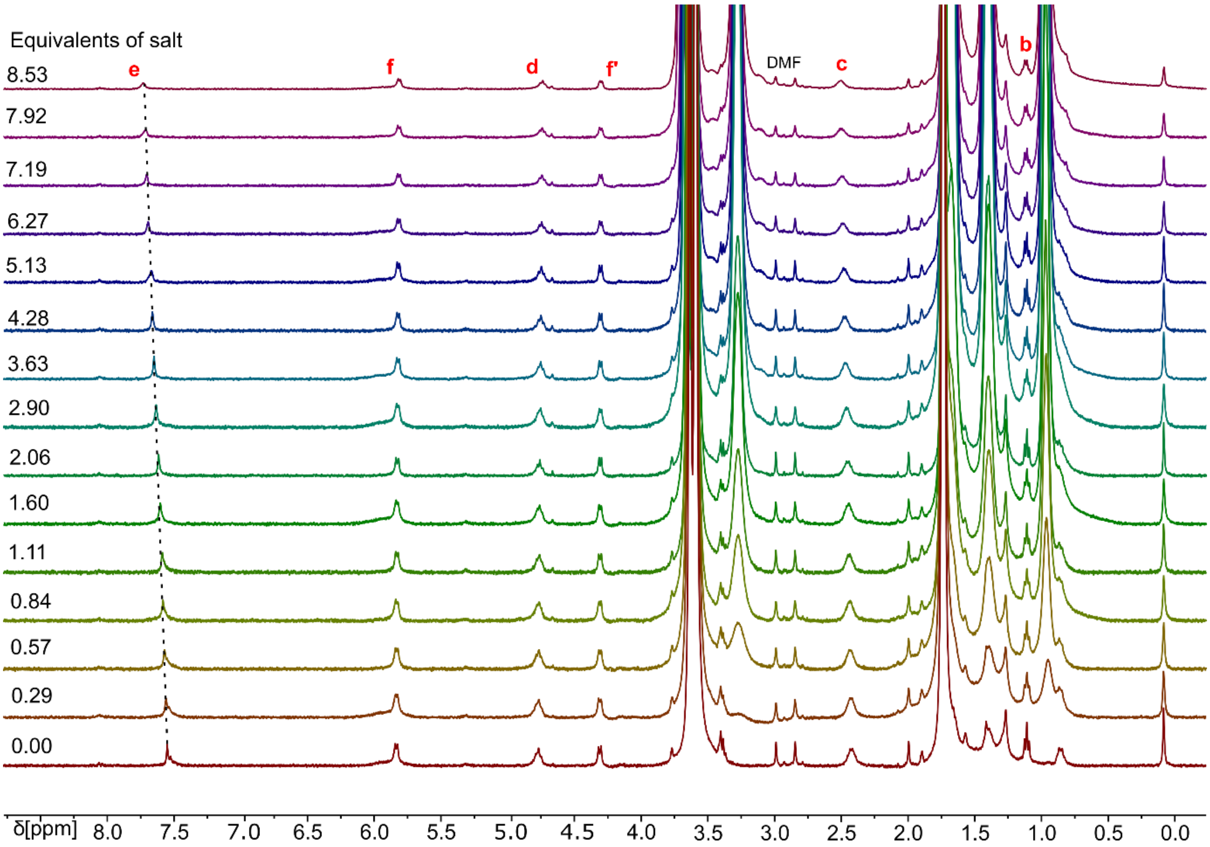

Figure S73. ^{1}H NMR spectra for the titration of **7** (0.0067 M) with **But_4NHSO_4** in THF-d_8/10%D_2O at 298 K.

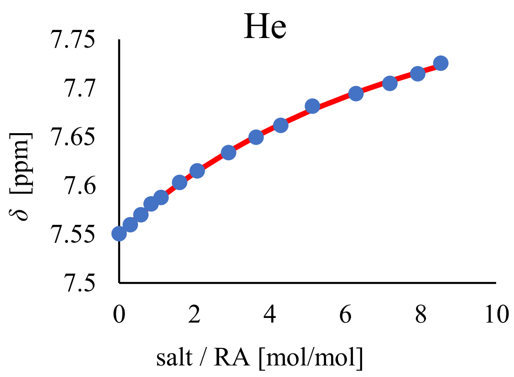

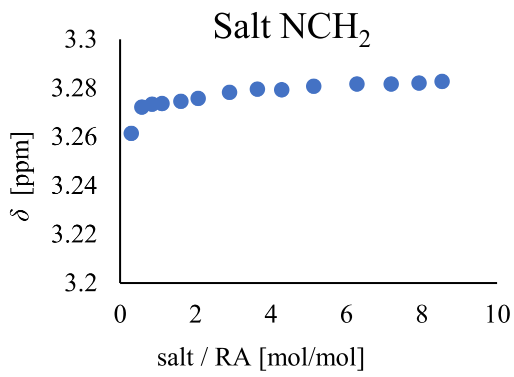

K_{11}(M^{-1})	K error (%)	SSR	Datapoints fitted	Params fitted
17.2968579	1.61283934	3.6617E-05	15	2

http://app.supramolecular.org/bindfit/view/60cf653a-307c-4b88-9a87-067f929b5992

Figure S74. ^{1}H NMR experimental points and fitted curves for the titration of **7 (RA)** (0.0067 M) with **But_4NHSO_4 (salt)** ^{1}H NMR chemical shifts' changes for: (a) H_e; and (b) NCH_2 of the salt (298 K, THF-d_8/10%D_2O). Red lines correspond to fitted curves.

NMR titration of 7 with But_4NClO_4 in THF-d_8/10% D_2O

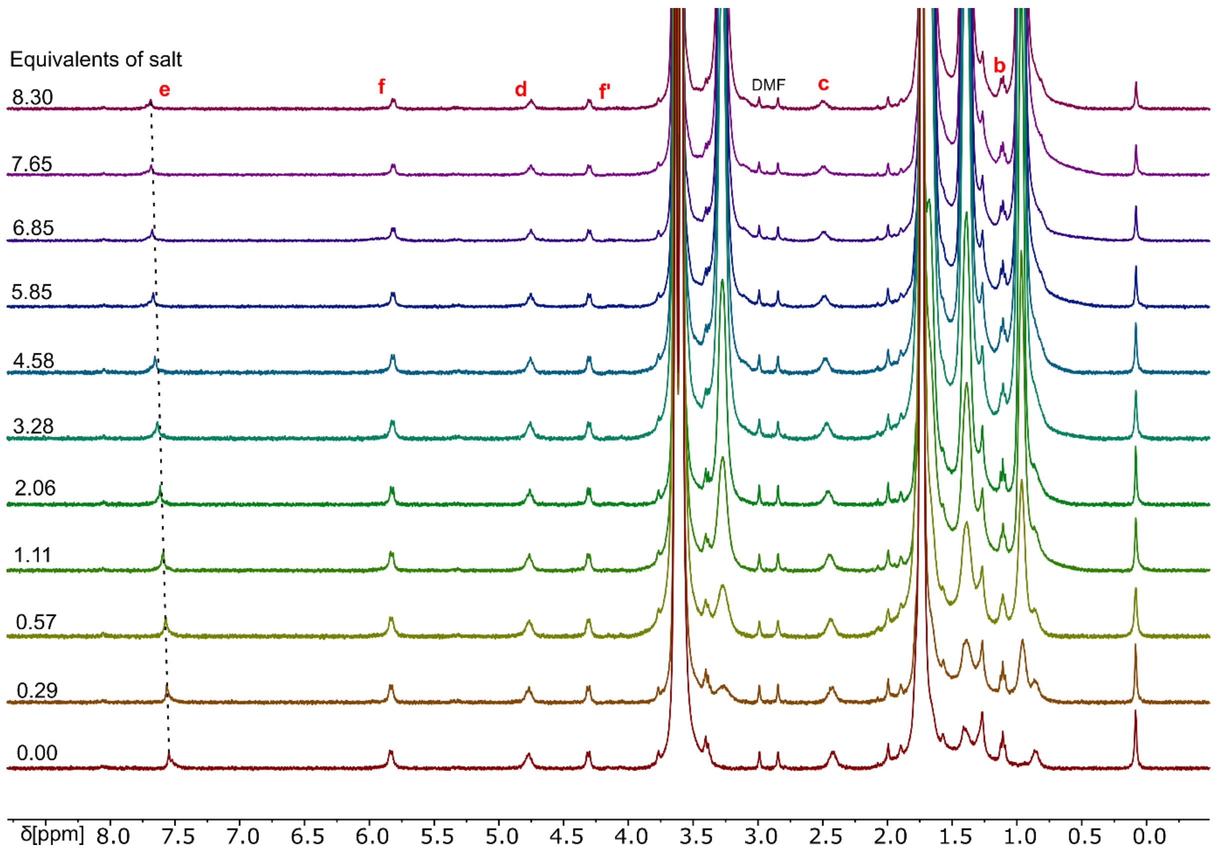

Figure S75. ^{1}H NMR spectra for the titration of **7** (0.0067 M) with **But_4NClO_4** in THF-d_8/10%D_2O at 298 K.

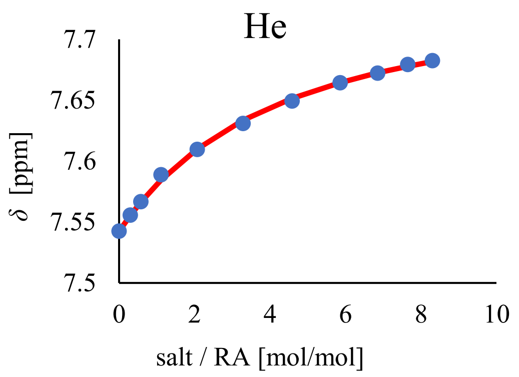

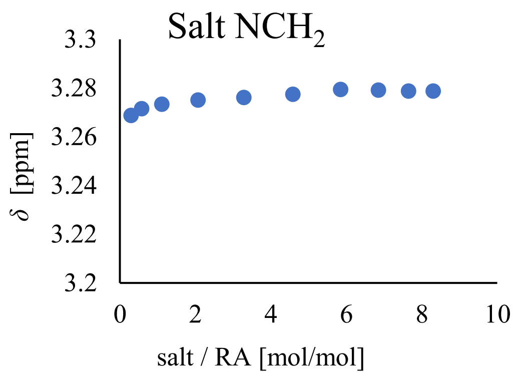

K_{11}(M^{-1})	K error (%)	SSR	Datapoints fitted	Params fitted
43.0134179	3.87079388	3.5348E-05	11	2

http://app.supramolecular.org/bindfit/view/26015d07-bad0-422f-bdb0-227c2181fa2e

Figure S76. ^{1}H NMR experimental points and fitted curves for the titration of **7 (RA)** (0.0067 M) with **But_4NClO_4 (salt)** ^{1}H NMR chemical shifts' changes for: (a) H_e; and (b) NCH_2 of the salt (298 K, THF-d_8/10%D_2O). Red lines correspond to fitted curves.

NMR titration of 7 with $But_4NH_2PO_4$ in THF-d_8/10% D_2O

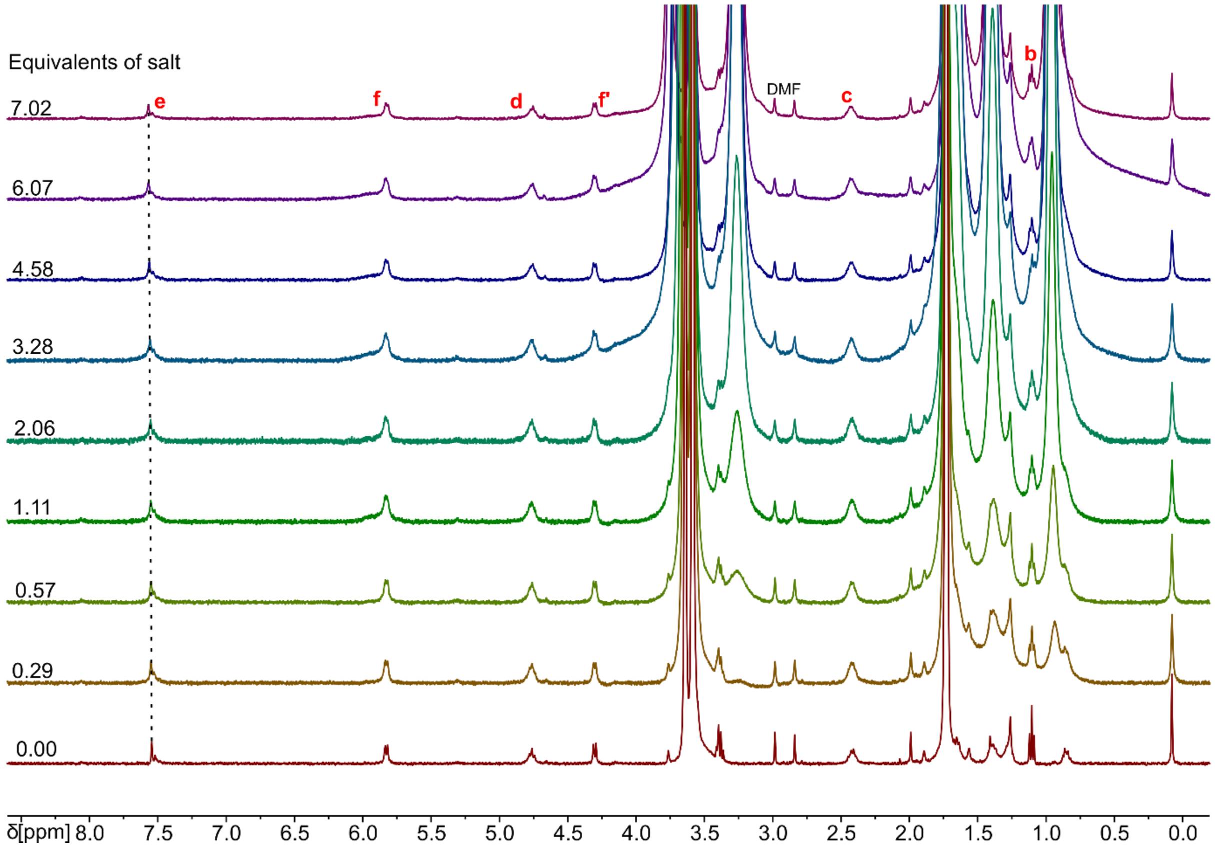

Figure S77. ^{1}H NMR spectra for the titration of **7** (0.0067 M) with **$But_4NH_2PO_4$** in THF-d_8/10%D_2O at 298 K.

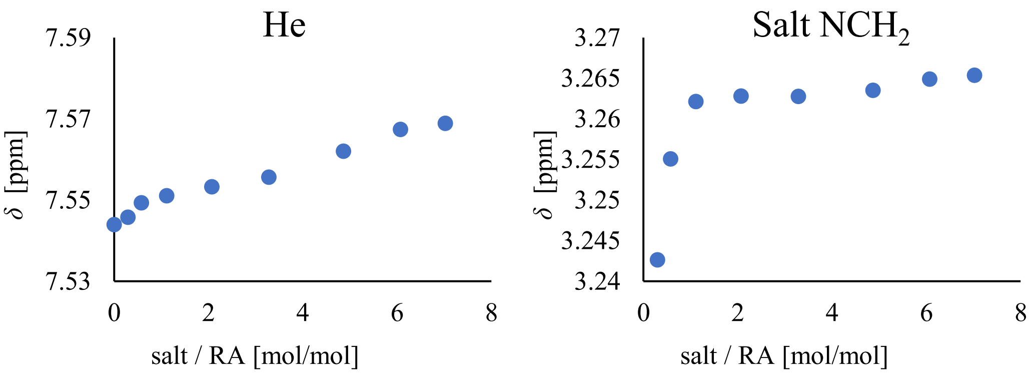

Figure S78. ^{1}H NMR experimental points for the titration of **7 (RA)** (0.0067 M) with **$But_4NH_2PO_4$ (salt).** ^{1}H NMR chemical shifts' changes for: (a) H_e; and (b) NCH_2 of the salt (298 K, THF-d_8/10%D_2O). The data could not be fitted due to negligible binding.

NMR titration of 7 with But_4NReO_4 in THF-d_8/10% D_2O

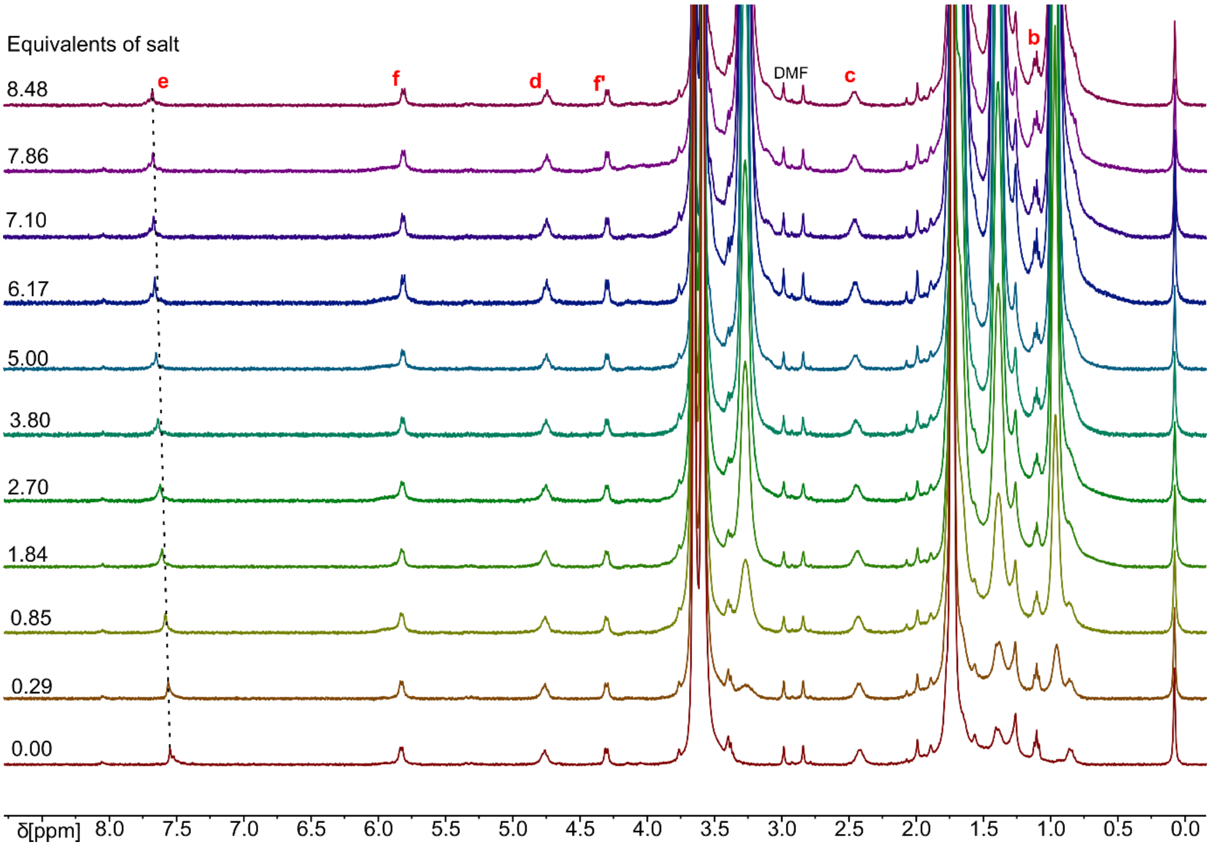

Figure S79. ^{1}H NMR spectra for the titration of **7** (0.0067 M) with **But_4NReO_4** in THF-d_8/10%D_2O at 298 K.

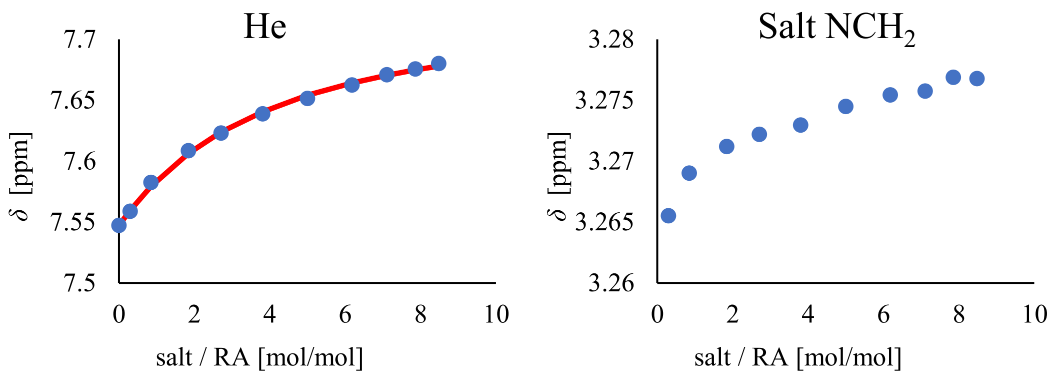

K_{11}(M^{-1})	K error (%)	SSR	Datapoints fitted	Params fitted
45.7525301	4.19648107	3.6988E-05	11	2

http://app.supramolecular.org/bindfit/view/d60355f7-5488-488c-b1e1-578a5ccc3645

Figure S80. ^{1}H NMR experimental points and fitted curves for the titration of **7 (RA)** (0.0067 M) with **But_4NReO_4 (salt)**^{1}H NMR chemical shifts' changes for: (a) H_e; and (b) NCH_2 of the salt (298 K, THF-d_8/10%D_2O). Red lines correspond to fitted curves.

5. COMPETITION EXPERIMENTS

Receptor 2 and receptor 5 with Pen_4NCl in THF-d_8 at 298 K

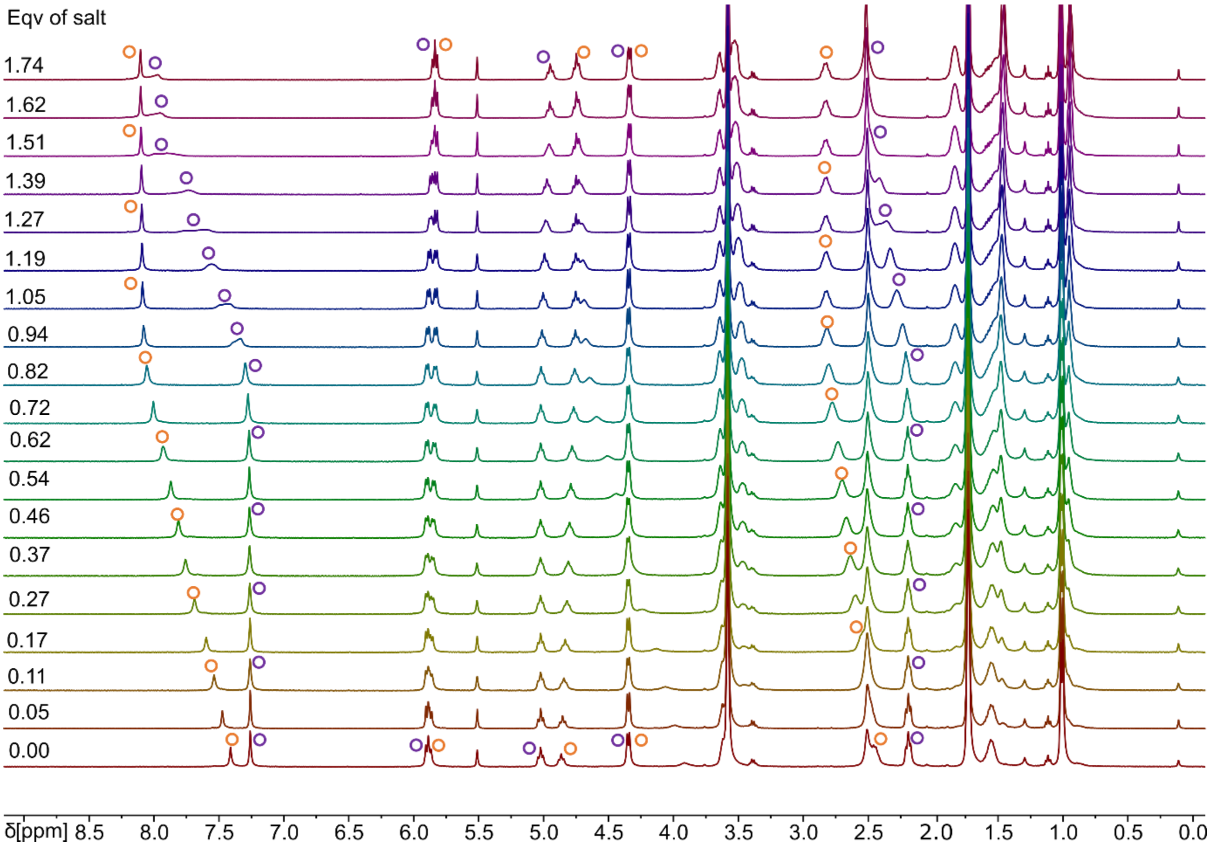

Figure S81. ^{1}H NMR competitive titration. ^{1}H NMR spectra for the titration of mixture of receptors **2** (○) (0.0067 M) + **5** (○) (0.0067 M) with Pen_4NCl in THF-d_8 at 298 K.

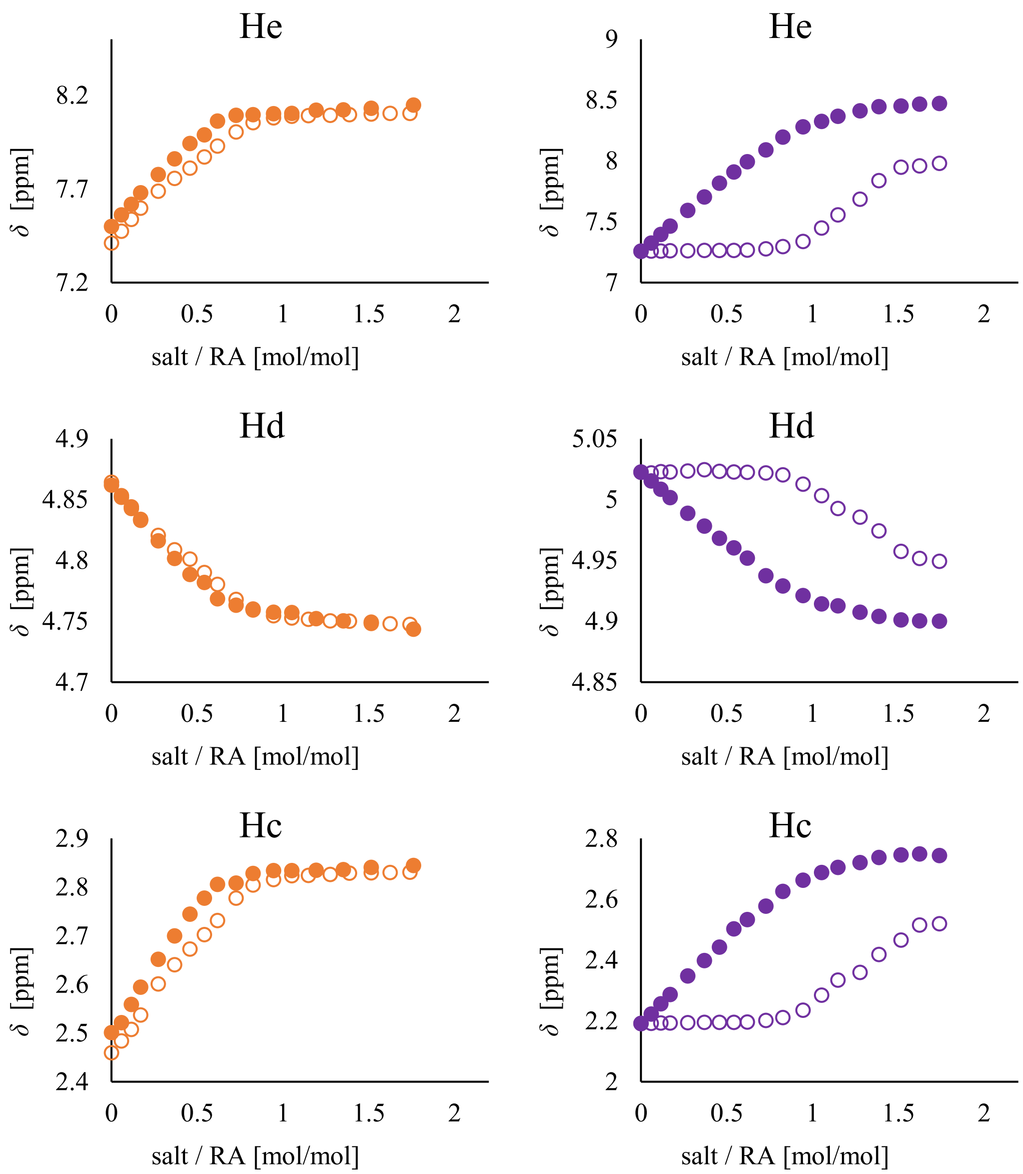

Figure S82. ^{1}H NMR competitive titration. Comparison of chemical shift changes during titrations of: mixture of receptors **2** (○) (0.0067 M) + **5** (○) (0.0067 M) **(RA)** with **Pen_4NCl (salt)** in THF-d_8 at 298 K with the analogous titrations of separate components **2** (●) and **5** (●) with Pen_4NCl in THF-d_8 at 298 K.

Receptor 5 and receptor 6 with Pen$_4$NCl in THF-d_8 at 298 K

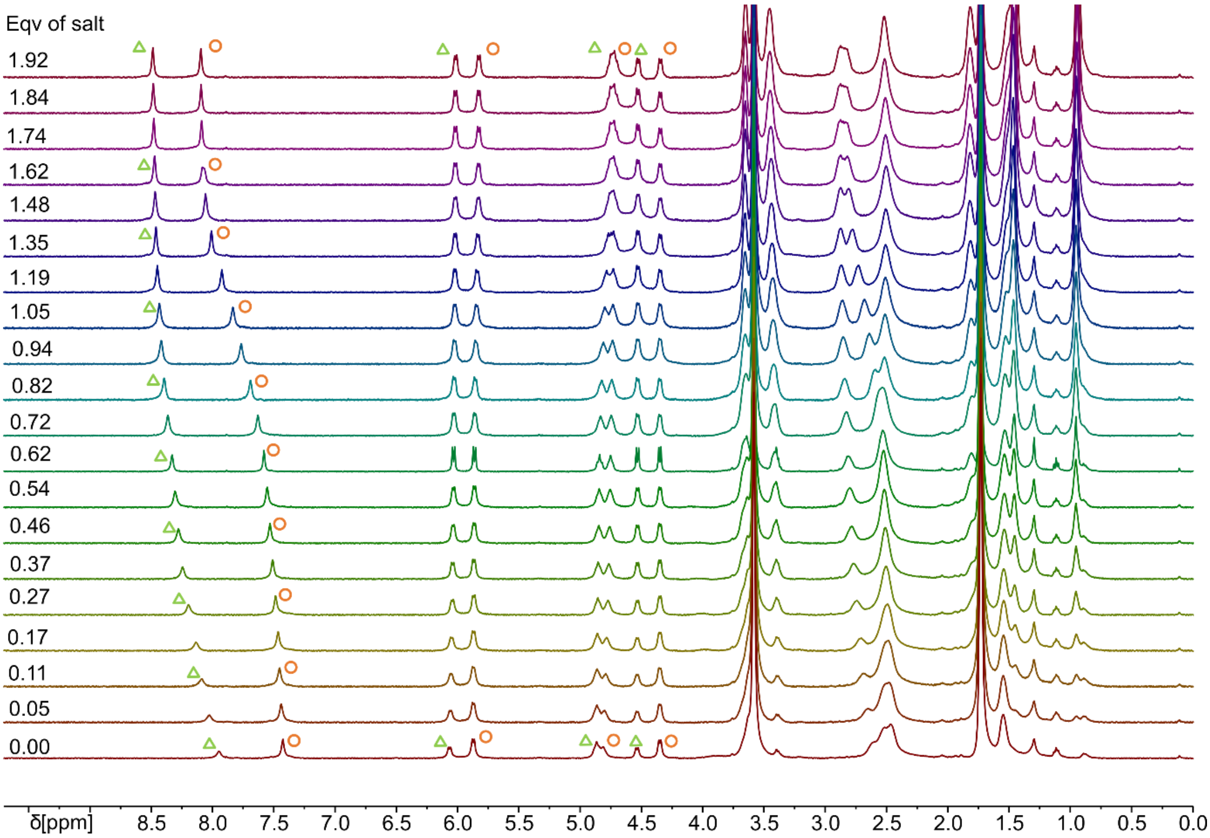

Figure S83. ^{1}H NMR competitive titration. ^{1}H NMR spectra for the titration of mixture of receptors **5** (○) (0.0067 M) + **6** (Δ) (0.0067 M) with Pen$_4$NCl in THF-d_8 at 298 K.

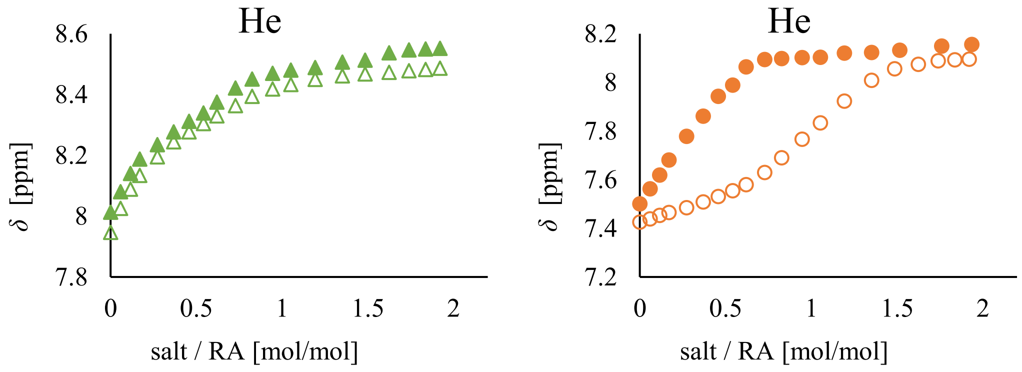

Figure S84. ^{1}H NMR competitive titration. Comparison of chemical shift changes during titrations of: mixture of receptors **5** (○) (0.0067 M) + **6** (Δ) (0.0067 M) **(RA)** with **Pen$_4$NCl (salt)** in THF-d_8 at 298 K with the analogous titrations of separate components **5** (●) + **6** (▲) with Pen$_4$NCl in THF-d_8 at 298 K.

6. SINGLE CRYSTAL X-RAY DIFFRACTION

The crystals were embedded in the inert perfluoropolyalkylether (viscosity 1800cSt; ABCR GmbH) and mounted using Hampton Research Cryoloops. The crystals were flash cooled to 100.0(1) K in a nitrogen gas stream and kept at this temperature during the experiments. The X-ray data were collected on a SuperNova Agilent diffractometer using Cu*Kα* radiation (λ = 1.54184 Å) and Mo*Kα* radiation (λ = 0.71073 Å). The data were processed with *CrysAlisPro*.[7] Structures were solved by direct methods and refined using *SHELXL*[8] under *WinGX*.[9]

[7] Agilent Technologies, *CrysAlisPro*, Version 1.171.40.84a.
[8] Sheldrick, G.M. Crystal Structure Refinement with SHELXL. *Acta Crystallogr., Sect. C: Struct. Chem*. **2015**, *C71*, 3-8.
[9] Farrugia, L.J. WinGX suite for small-molecule single-crystal crystallography. *J. Appl. Cryst*. **1999**, *32*, 837- 838.

Crystal data for [4 complex Pen$_4$NBr]:

The single crystals for X-ray analysis were obtained by slow evaporation of a solution containing **4** and Pen$_4$NBr (1:1) in THF/10%DMF/10%$CHCl_3$. ($C_{76}H_{104}BrN_5O_9$), *Mr* = 1311.6, colorless prism, tetragonal, space group *I 4*, *a* = 11.6555(2), *c* = 28.3961(5) Å, *V* = 3857.63(15) Å^3, *Z* = 2, ρ_{calc} = 1.13 g cm^{-3}, μ(CuKα) = 1.16 mm^{-1}, θ_{max} = 70.5°, 23563 reflections measured, 3610 unique, 344 parameters, *R* = 0.070, *wR* = 0.188 (*R* = 0.081, *wR* = 0.208 for all data), GooF = 1.05. CCDC 2443642.

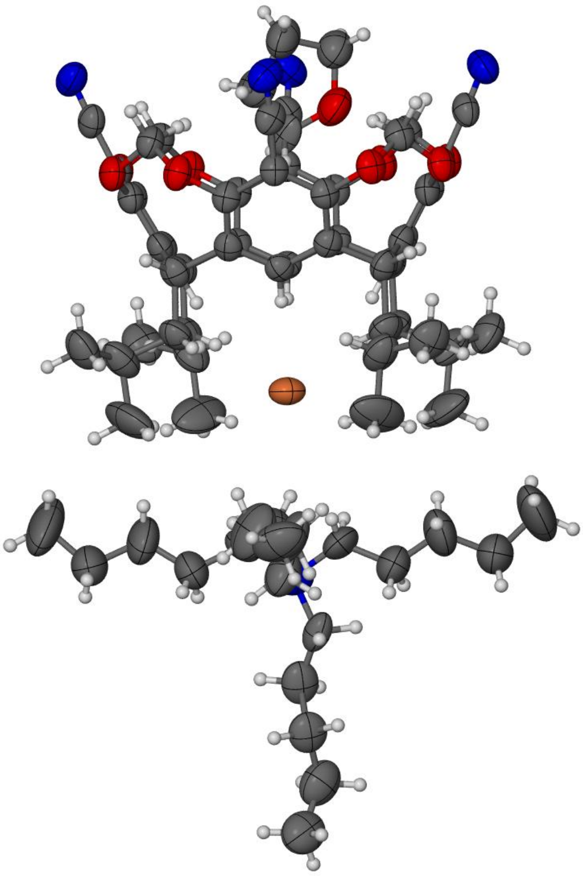

Figure S85. Expanded asymmetric unit of **[4 complex Pen$_4$NBr]**. Only one position of the disordered by symmetry THF molecule and tetrapentylammonium cation (both lie on a four-fold axis) is shown for clarity. Displacement ellipsoids are drawn at the 50% of probability level.

Crystal data for 6:

The single crystals for X-ray analysis were obtained by slow evaporation of a solution containing **6** in THF/10%H_2O.

($C_{60}H_{74.5}N_4O_{18.25}$), *Mr* = 1143.5, colorless prism, monoclinic, space group *I 2/m*, *a* = 11.6626(8), *b* =26.328(2), *c* = 19.6105(14) Å, β = 106.038(7)°, *V* = 5787.1(8)Å^3, *Z* = 4, ρ_{calc} = 1.31 g cm^{-3}, μ(MoKα) = 0.10 mm^{-1}, θ_{max} = 25.7°, 51078 reflections measured, 9671 unique, 467 parameters, *R* = 0.069, *wR* = 0.170 (*R* = 0.140, *wR* = 0.185 for all data), GooF = 0.88. CCDC 2443641.

The crystal was non-merohedrally twinned by 180° rotation around [0.22 0.00 0.98] in the reciprocal space. The structure solution was conducted with data of one component only and the refinement was conducted as a 2-component twin. The twin factor refined to 0.353.

The unit cell contains disordered solvent molecules which have been treated as a diffuse contribution to the overall scattering without specific atom positions by SQUEEZE/PLATON.[10] The crystals have been obtained from the solvent mixture comprising hexane, THF and water. The atomistic solvent disorder model for THF molecules residing between layers of the macrocycles was not satisfactory. The number of electrons recovered from the solvent accessible voids in the unit cell amounts to 302 electrons, corresponding to *app*. 76 electrons *per* formula unit, where 80 electrons are expected for the full occupancy of two THF molecules. The contribution of these two disordered THF molecules removed by the SQUEEZE have been included in the overall formula, formula weight, density, F (000), *etc* in CIF file.

[10] Spek AL. PLATON SQUEEZE: a tool for the calculation of the disordered solvent contribution to the calculated structure factors. *Acta Crystallogr., Sect. C: Struct. Chem.* **2015,** *C71*, 9-18.

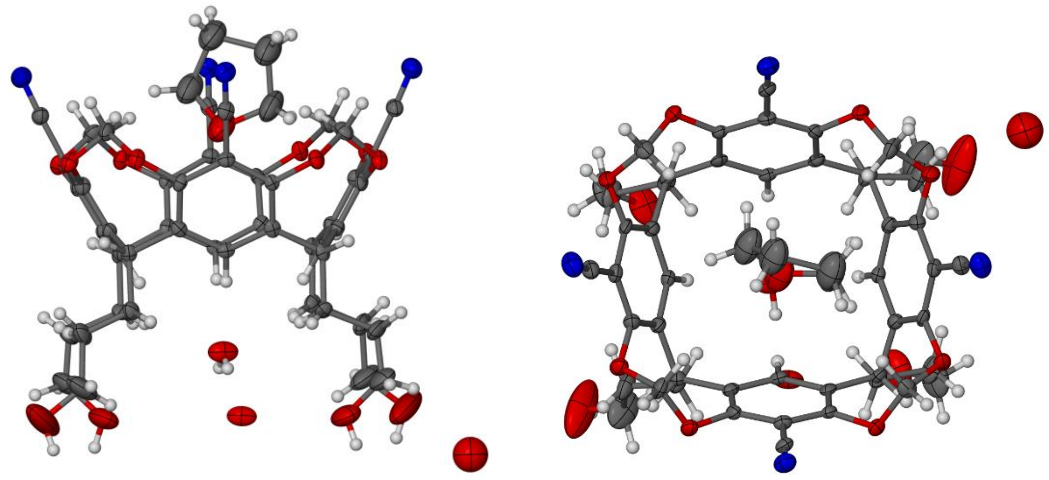

Figure S86. Expanded asymmetric unit of **6** – side and top views. Only one position of the disordered by symmetry THF molecule residing in the macrocyclic cavity is shown for clarity. Displacement ellipsoids are drawn at the 50% of probability level.

7. ANION TRANSPORT STUDIES

Materials

All solvents and reagents were obtained from commercial sources and used without any further purification or modification, unless otherwise stated. The sources of specific materials and reagents are listed below:

Sigma-Aldrich:

- Aluminum oxide (activated, basic; Cat. No. 199443)
- Sodium hydroxide solution (2 N, Titripur; Cat. No. 1.09136.1000)
- Chloroform (>99%, stabilized with amylenes; Cat. No. 372978)
- Sodium nitrate (≥99.0%; Cat. No. 221341)
- *N*,*N*′-Dimethyl-9,9′-biacridinium dinitrate (lucigenin; Cat. No. M8010)
- 1-Palmitoyl-2-oleoyl-*sn*-glycero-3-phosphocholine (POPC; ≥99%, Cat. No. 850457P, Avanti PC).

GE Healthcare:

- Sephadex™ G-50 Superfine (Cat. No. 51186500-EG).

Milli-Q water was used in all anion transport studies. Tetrahydrofuran (THF), purified using a solvent purification system (SPS), was used for the preparation of certain stock solutions, as specified below.

Instruments and Methods

- Fluorescence Spectroscopy:

Fluorescence spectra were acquired using a Hitachi F-7000 fluorescence spectrophotometer equipped with a magnetic stirrer (Hitachi High-Tech Science) and a temperature controller from Huber.

- Extrusion:

An AVESTIN LiposoFast-Basic extruder with polycarbonate membranes (200 nm pore size, Avanti Polar Lipids, Inc.) was used for the extrusion process during the preparation of LUVs.

General Procedure for the Preparation of LUVs with Pre-Incorporated Transporters:

Large unilamellar vesicles (LUVs) with pre-incorporated resorcin[4]arenes were prepared following a previously reported procedure,[11] except for the following modifications: no cholesterol was used and chloroform was dried under argon.

Analytical grade chloroform (Sigma-Aldrich, Cat. No. 372978, stabilized with amylenes) was first de-acidified by passing through a pad of activated basic alumina in a glass Pasteur pipette. This de-acidified chloroform was then used to prepare a 10 mM solution of POPC.

In a 5 ml round bottom flask, 600 µl of 10 mM POPC solution in chloroform and the required amount of resorcin[4]arene stock solution was combined and vortexed to ensure thorough mixing. The solvent was then evaporated under a stream of argon, and the resulting lipid film was dried under high vacuum for 2 h.

The dried lipid film was hydrated with 0.5 ml of lucigenin solution (0.8 mM in 225 mM aqueous solution of $NaNO_3$, pre-filtered through a <0.2 µm PTFE syringe filter) and vortexed at 1500 rpm for 1 h at room temperature. The suspension was then subjected to 10 freeze-thaw cycles using liquid nitrogen and a 30 °C water bath to eliminate multilamellar vesicles. Next, 0.5 ml of the same lucigenin solution was added, and the entire mixture was extruded 31 times

[11] Abdurakhmanova, E. R.; Mondal, D.; Jędrzejewska, H.; Cmoch, P.; Danylyuk, O.; Chmielewski, M. J.; Szumna, A. Supramolecular umpolung: Converting electron-rich resorcin[4]arenes into potent CH-bonding anion receptors and transporters. *Chem* **2024**, *10*, 1910–1924.

through a 200 nm polycarbonate membrane to form LUVs with an average diameter of 200 nm.

To remove extravesicular lucigenin, the vesicle suspension was passed through a Sephadex G50 column (approximately 2 g of Sephadex G-50 packed in a 20 × 1.8 cm column) using 225 mM aqueous solution of $NaNO_3$ as the eluent. The collected vesicle suspension was then diluted to a final volume of 15 ml with the same $NaNO_3$ solution, yielding a final lipid concentration of approximately 0.4 mM.

Data Acquisition

Into a clean and dry quartz cuvette (10 × 10 mm) equipped with a small magnetic stir bar, 2 ml of the vesicle suspension (0.4 mM) was added. The cuvette was placed in the spectrofluorometer equipped with an integrated stirrer, and fluorescence intensity was recorded as a function of time (λ_{ex} = 455 nm, λ_{em} = 505 nm).

Anion transport was initiated by injecting a pulse of NaCl (50 µl, 1 M) at t = 0 s. Fluorescence was monitored for 600 s following NaCl addition. Finally, Triton-X (20 µl of 10% *v*/*v* solution in water) was added to lyse the vesicles, and data collection continued for an additional 30 s.

Notes:

1. Each transport experiment was performed at least twice with independently prepared batches of liposomes, and the results were averaged as described below.
2. Control (blank) experiments were performed using vesicles prepared in the same manner, but without the addition of resorcin[4]arene.

Processing the Data from the Lucigenin Assay:

Quantification of transport rates from fluorescence data:

First, fluorescence data recorded prior to the addition of NaCl were discarded. The initial time point t_0 was set as the last point recorded immediately before the sudden fluorescence drop caused by the addition of NaCl. The fluorescence data from the first 9.5 seconds after the injection (0.0-9.5 s), which reflect quenching of residual lucigenin and mixing effects, were also excluded from analysis.

The remaining fluorescence intensity values (F) were reciprocated ($1/F$) and plotted as a function of time, with the first $1/F$ value corresponding to t = 10.0 s.

According to the Stern-Volmer equation, $1/F$ is directly proportional to the chloride concentration. Therefore, the fluorescence data collected from 10 to 600 s were fitted with a single-exponential decay function:

$$\frac{1}{F_{(t)}} = \frac{1}{F_\infty} - (\frac{1}{F_\infty} - \frac{1}{F_0})e^{-bt}$$

where F_∞, F_0, and b are fitting parameters.

Graphical representation of transport kinetics:

The F_0 values obtained from the fitting procedure were used to normalize the fluorescence intensity data as $F(t)/F_0$. Normalized data from replicate measurements were averaged to generate a single curve, $F_{AVG}(t)/F_0$, for each experiment. Subsequently, the $F_{AVG}(t)/F_0$ curves from independent experiments (using different liposome batches) were further averaged to give the final plot, $F_{FINAL}(t)/F_0$, showing the transport kinetics. The $F_{FINAL}(t)/F_0$ curves are presented together with their corresponding error margins, indicated by shaded areas in pale colour.

Calculation of transport rate constants and half-life times:

The transport rate constants (k) was determined from the fitting parameters b as follows. First, the b values obtained from all curves recorded using the same batch of liposomes were averaged to give b_{AVG}. The final k value was obtained by averaging b_{AVG} values from independent experiments (i.e. the experiments performed with different batches of liposomes).

Half-life times $t_{1/2}$ were calculated according to the following formula:

$$t_{1/2} = \frac{\ln(2)}{k}$$

Calculation of initial rates and specific initial rates:

To calculate the initial transport rate (I), the normalized fluorescence data from each replicate ($F(t)/F_0$, 10.0-600 s) were reciprocated and averaged to generate $(F_0/F(t))_{AVG}$, for each experiment. Next it was plotted as $(F_0/F(t))_{AVG}$ versus time and these curves were fitted with a double exponential function:

$$\frac{F_0}{F_{(t)}} = y_0 - Ae^{-Bt} - Ce^{-Dt}$$

The initial rates (I) were calculated using the fitting parameters A, B, C, and D as follows:

$$I = (A \times B) + (C \times D)$$

Initial rates from independent experiments were averaged to yield the final values.

To assess transporter efficiency, specific initial rates (I_{specific}) were obtained by dividing each initial rate I by the corresponding transporter-to-lipid molar ratio. The values of (I_{specific}) obtained at different transporter concentrations were averaged to give the final value.

Preparation of Stock Solution of Transporters:

Table S1. Solvents used for the preparation of stock solutions of transporters for pre-incorporation of transporters into LUVs.

**Compound**	**Solvent(s) used**
**1**	Chloroform
**2**	Chloroform
**3**	Chloroform
**4**	Chloroform
**5**	THF
**6**	THF:H_2O (9:1, *v*:*v*)
**7**	THF:H_2O (9:1, *v*:*v*)

Results of Cl^- Transport Experiments in $NaNO_3$ Medium Using Lucigenin-Loaded LUVs with Pre-Incorporated Resorcin[4]arene Transporters

The Cl^- transport assay with pre-incorporated resorcin[4]arenes revealed that receptor **4** exhibits superior transport activity compared to receptors **2, 3**, **5**, **6,** and **7**. As a result, further studies were focused on transporter **4**.

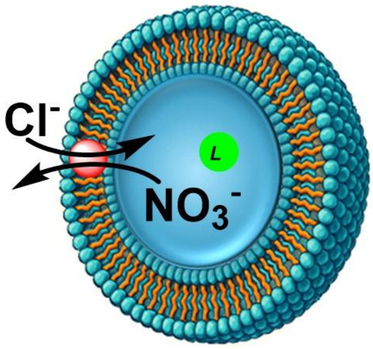

Details of Assay Condition	
**Inside**	**Outside**
0.8 mM lucigenin	——
225 mM $NaNO_3$	225 mM $NaNO_3$
——	25 mM NaCl

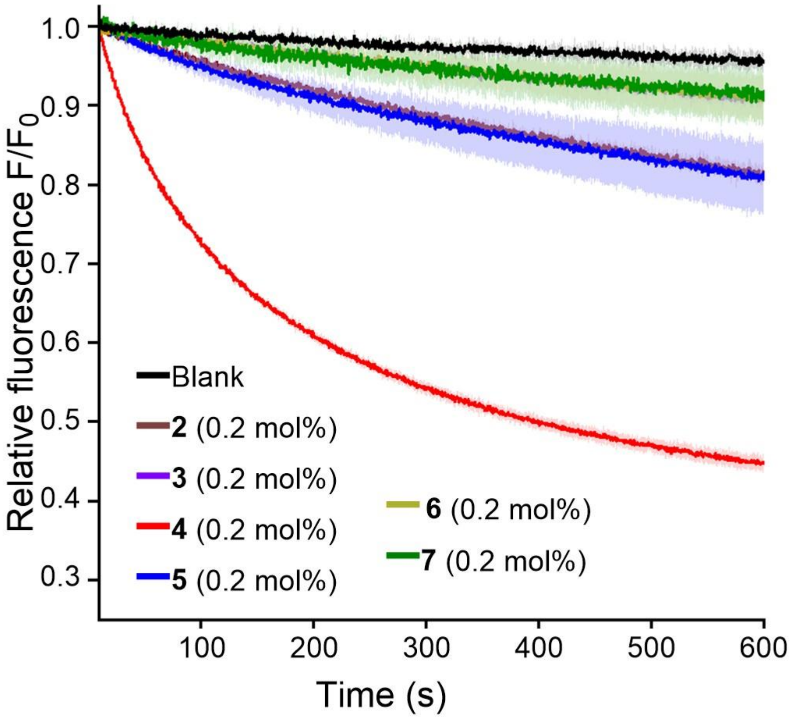

Figure S87. Changes in relative fluorescence intensity F/F_0 due to the transport of Cl^-ions into LUVs by resorcin[4]arenes **2**–**7** pre-incorporated into the lipid membrane at a concentration of 0.2 mol%. The graph compares the transport efficiencies of these resorcinarenes under identical conditions.

a)

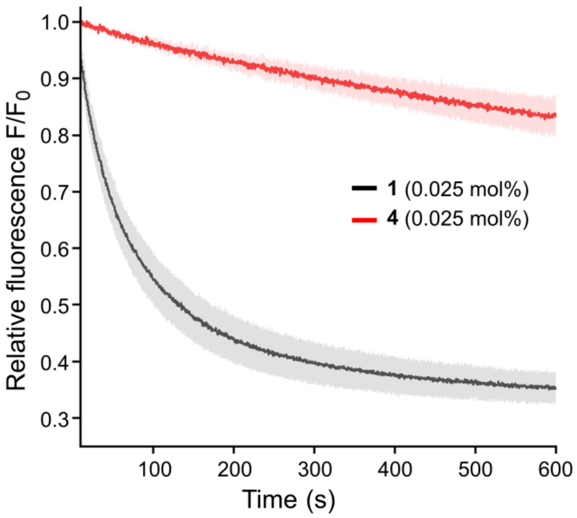

b)

Compoud [0.025 mol%]	**1**	**4**
Transport rate constant (*k*) [s^{-1}]	0.00575	0.00058
Half-life time ($t_{1/2}$) [s]	121	1202
Initial rate (*I*) [s^{-1}]	0.01255	0.00056
Specific initial rate ($I_{Specific}$) [s^{-1}]	50	2.2571

Figure S88. a) Changes in relative fluorescence intensity F/F_0 due to the transport of Cl^- ions into LUVs by resorcin[4]arenes **1** and **4**, pre-incorporated into the lipid membrane at a concentration of 0.025 mol%. b) Transport rate constants (*k*), half-life time ($t_{1/2}$) values, initial rates (*I*), and specific initial rate ($I_{specific}$) determined from fitting the results of chloride transport into LUVs mediated by resorcin[4]arene **1** and **4** at 0.025 mol%, pre-incorporated into the lipid membrane.

Results from Concentration-Dependent Cl^- Transport Experiments Using Lucigenin-Loaded LUVs with Pre-Incorporated 4.

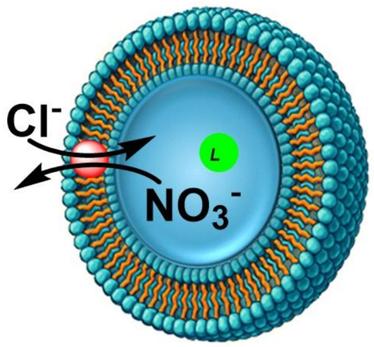

Details of Assay Condition	
**Inside**	**Outside**
0.8 mM lucigenin	——
225 mM $NaNO_3$	225 mM $NaNO_3$
——	25 mM NaCl

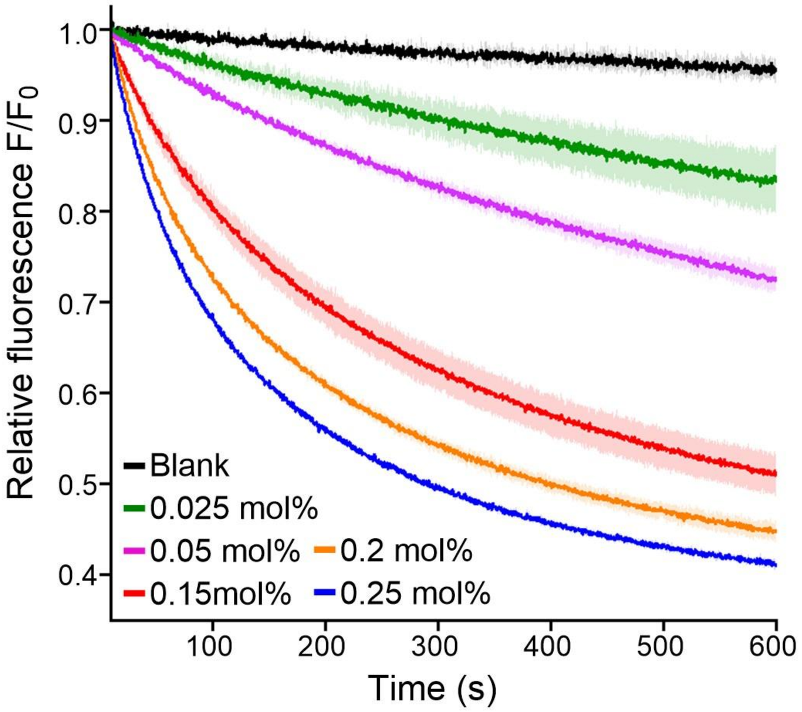

Figure S89. Changes in relative fluorescence intensity F/F_0 due to the transport of Cl^-ions into LUVs by resorcin[4]arene **4**, pre-incorporated into the lipid membrane at varying concentrations.

Following the procedure described above, fluorescence data from experiments with different concentrations of pre-incorporated **4** were processed to calculate: transport rate constant (k), half-life time ($t_{1/2}$), initial rate (I), and specific initial rate ($I_{specific}$).

Table S2. Transport rate constants (k), half-life time ($t_{1/2}$) values, initial rates (I), and specific initial rate ($I_{specific}$) determined from fitting the results of concentration-dependent chloride transport studies into LUVs mediated by resorcin[4]arene **4**, pre-incorporated into membrane.

**Concentration of 4 [mol%]:**	**0.25**	**0.2**	**0.15**	**0.05**	**0.025**
Transport rate constant (k) [s^{-1}]	0.00323	0.00273	0.00180	0.00077	0.00058
Half-life time ($t_{1/2}$) [s]	214	254	384	894	1202
Initial rate (I) [s^{-1}]	0.00733	0.00589	0.00348	0.00087	0.00056
Specific initial rate ($I_{specific}$) [s^{-1}]	2.9337	2.9471	2.3188	1.7315	2.2571
Averaged specific initial rate ($I_{specific}$) [s^{-1}]	2.4377				

Initial Rate:

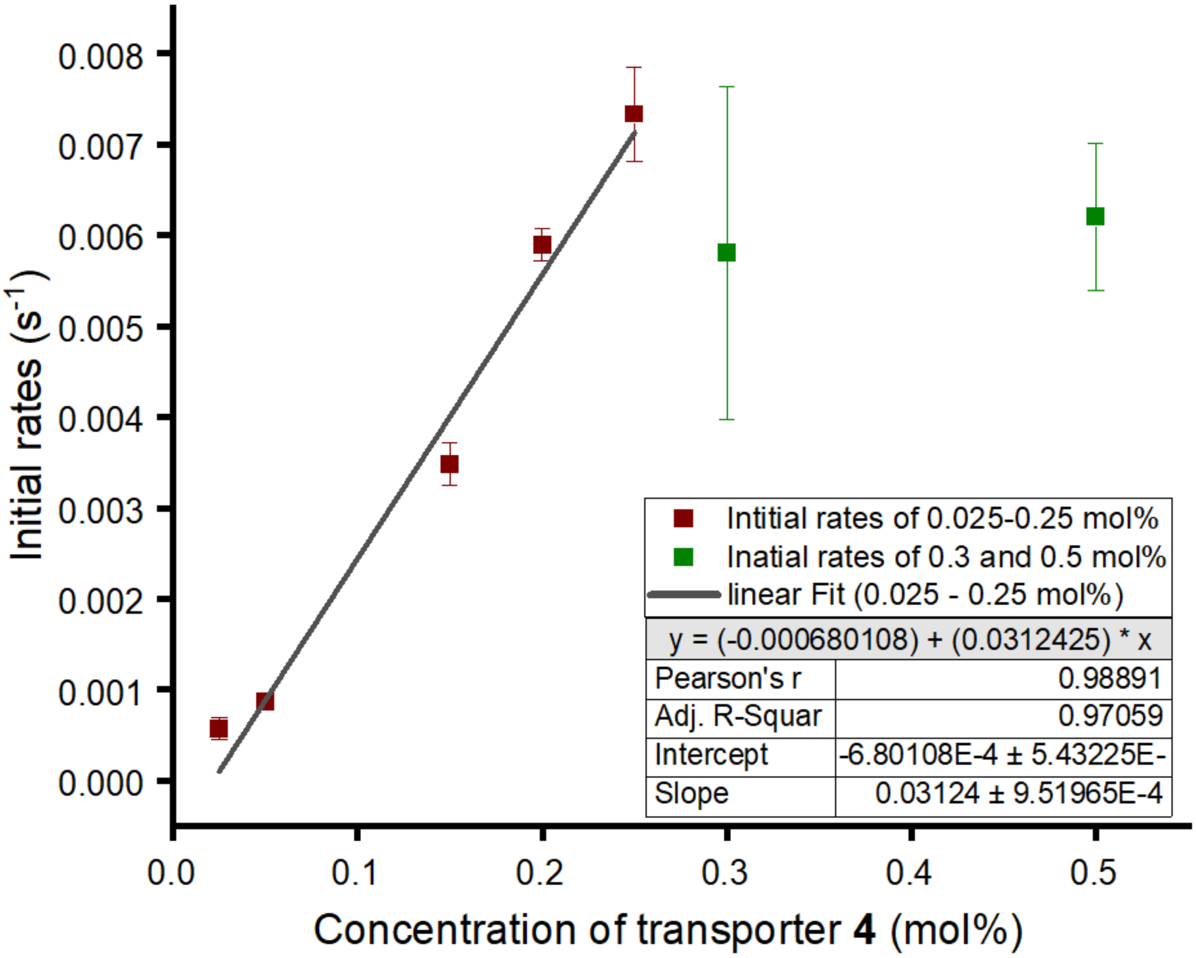

Figure S90. Initial rate (I) of Cl^{-} transport by receptor **4** plotted as a function of transporter concentration (mol%). A linear fit was applied (0.025 – 0.25 mol%) to illustrate the concentration dependence of transport activity. Beyond 0.25 mol% plateau was observed, suggesting that the solubility limit of **4** was exceeded.

Fitting Anion Transport Data for Resorcin[4]arene 4: Calculation of Half-Life Time Values:

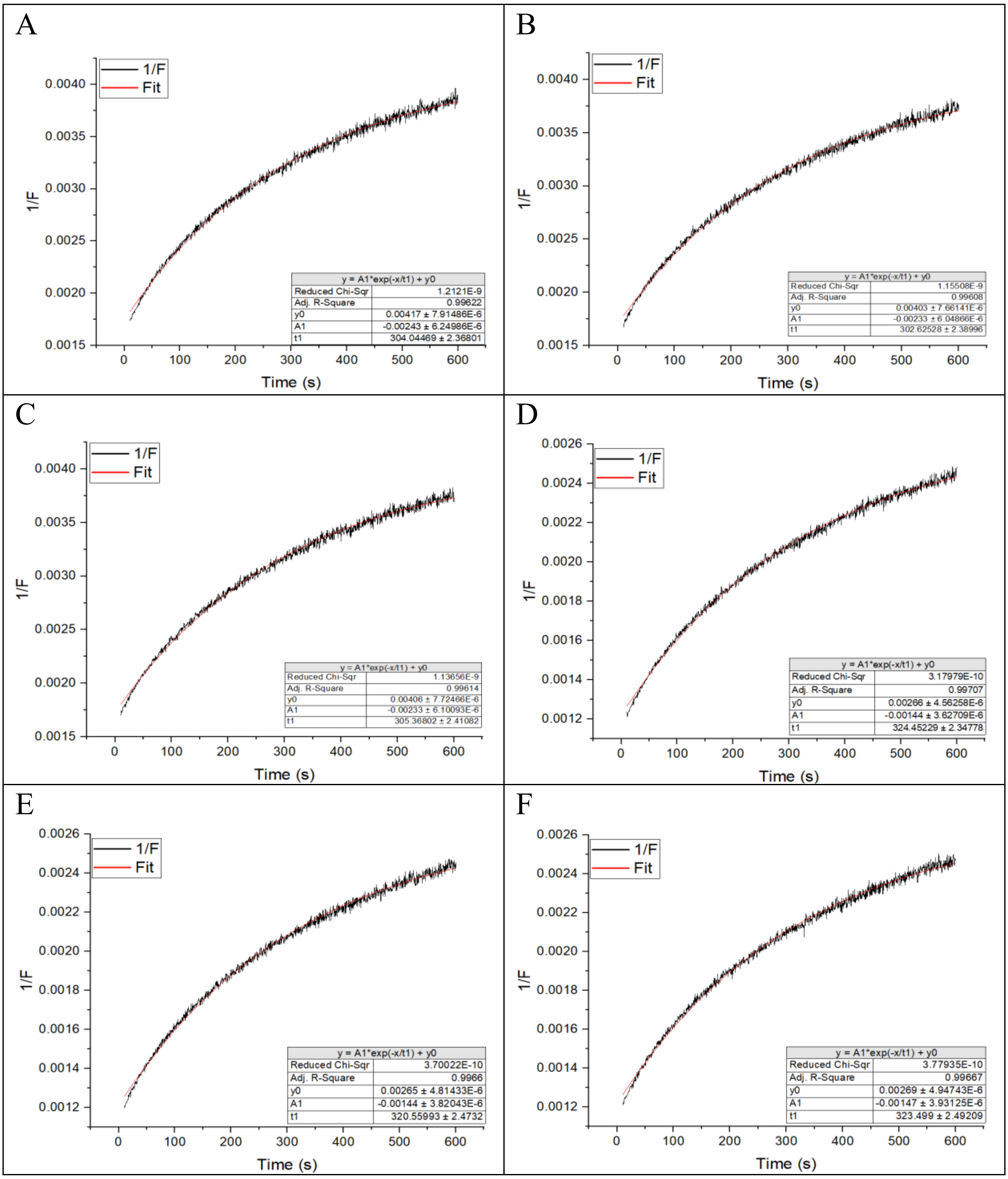

Figure S91. Changes in the reciprocated fluorescence (1/F) over time and corresponding single exponential decay fits for the transport of Cl^- ions into POPC LUVs by resorcin[4]arene **4**, pre-incorporated into the lipid bilayer at 0.5 mol% with respect to lipid.

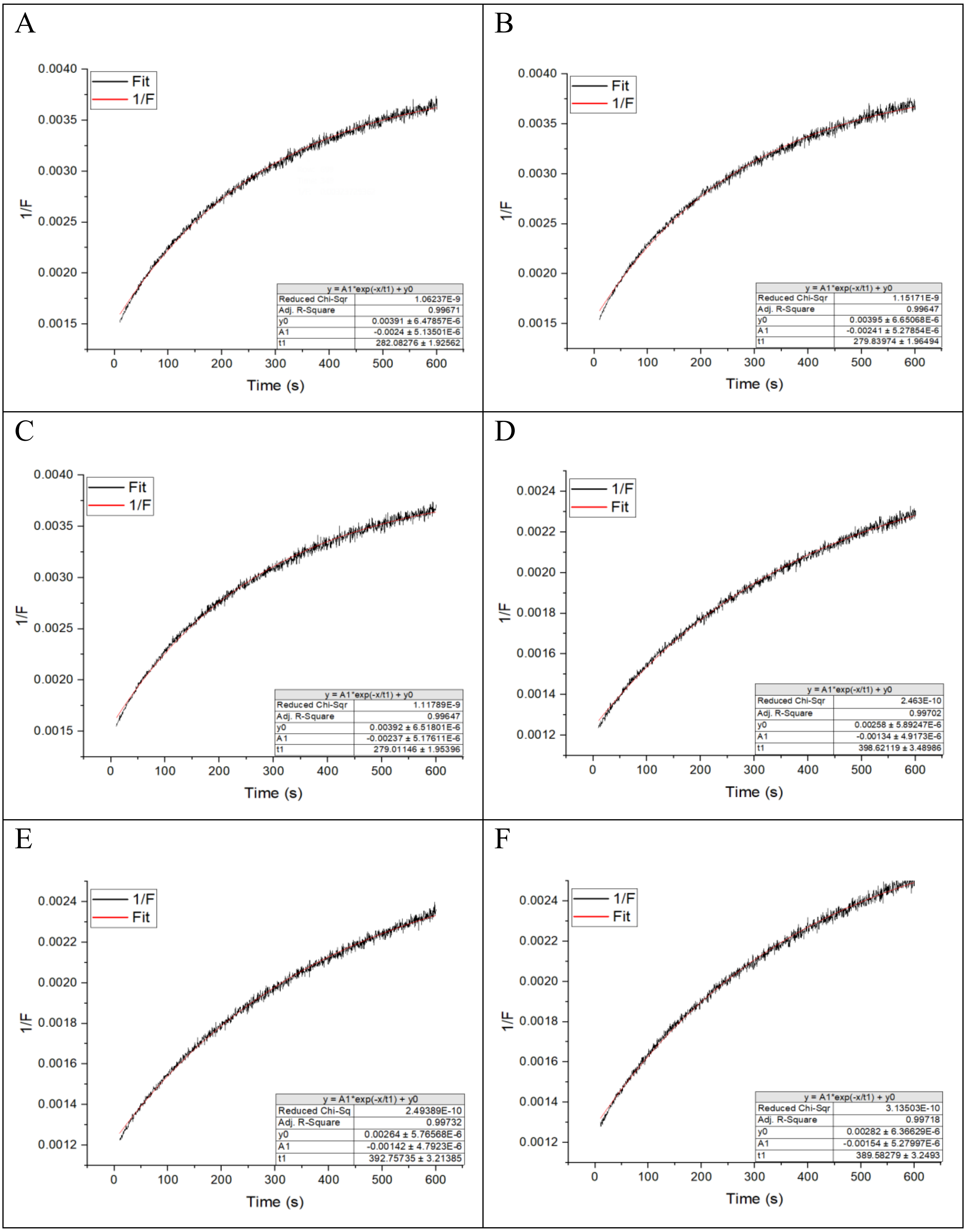

Figure S92. Changes in the reciprocated fluorescence (1/F) over time and corresponding single exponential decay fits for the transport of Cl^{-} ions into POPC LUVs by resorcin[4]arene **4**, pre-incorporated into the lipid bilayer at 0.3 mol% with respect to lipid.

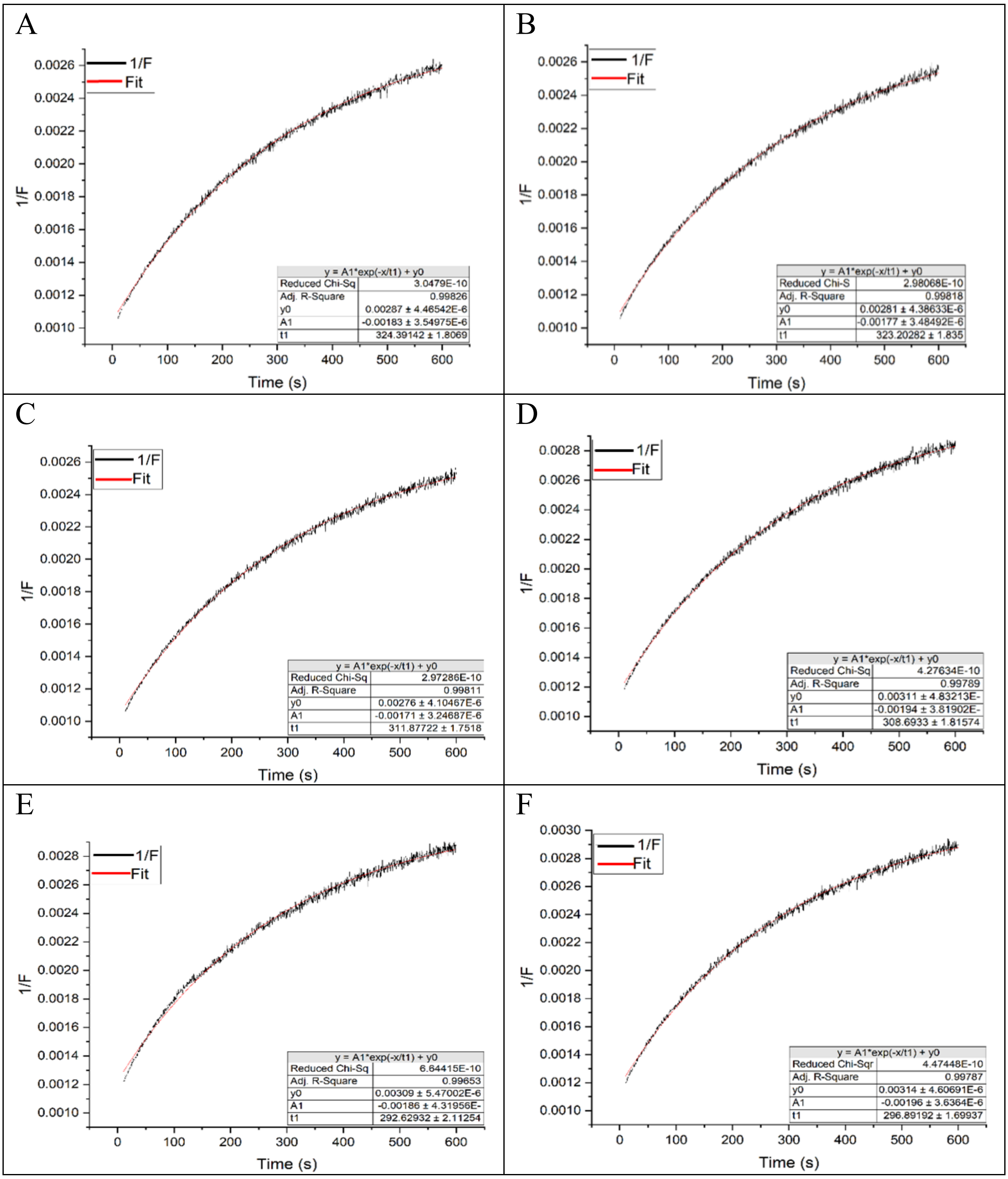

Figure S93. Changes in the reciprocated fluorescence (1/F) over time and corresponding single exponential decay fits for the transport of Cl^- ions into POPC LUVs by resorcin[4]arene **4**, pre-incorporated into the lipid bilayer at 0.25 mol% with respect to lipid.

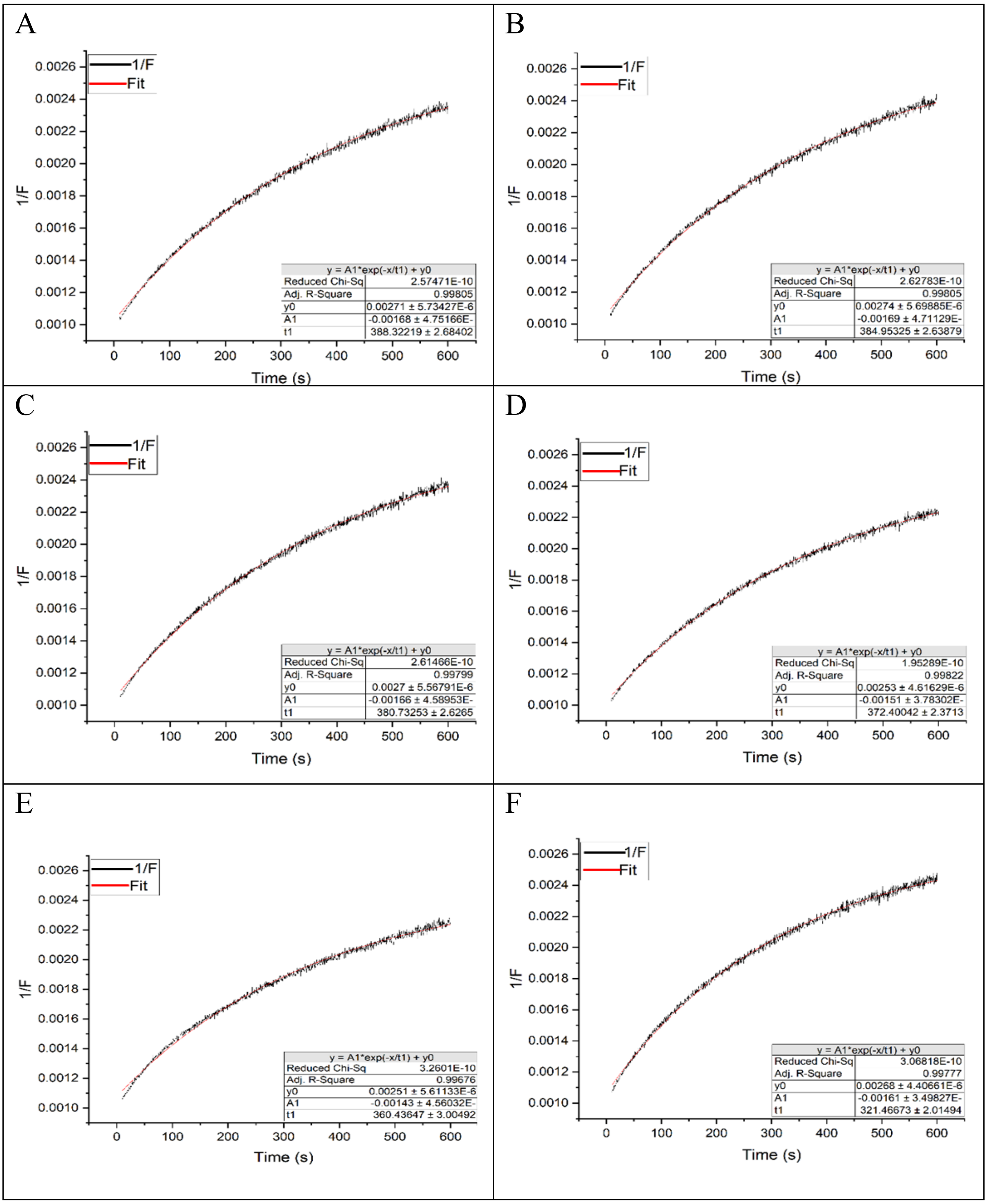

Figure S94. Changes in the reciprocated fluorescence (1/F) over time and corresponding single exponential decay fits for the transport of Cl^- ions into POPC LUVs by resorcin[4]arene **4**, pre-incorporated into the lipid bilayer at 0.2 mol% with respect to lipid.

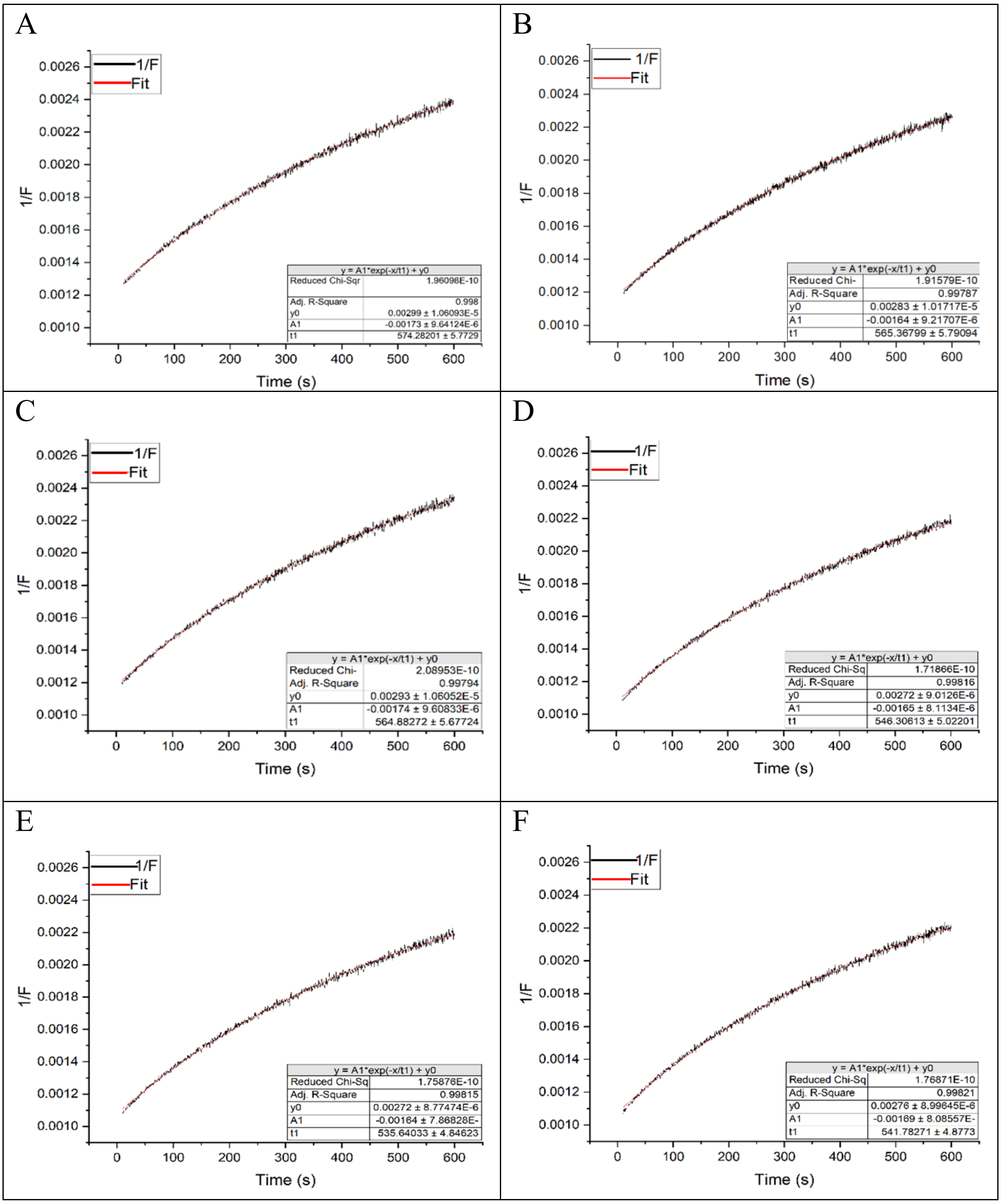

Figure S95. Changes in the reciprocated fluorescence (1/F) over time and corresponding single exponential decay fits for the transport of Cl^- ions into POPC LUVs by resorcin[4]arene **4**, pre-incorporated into the lipid bilayer at 0.15 mol% with respect to lipid.

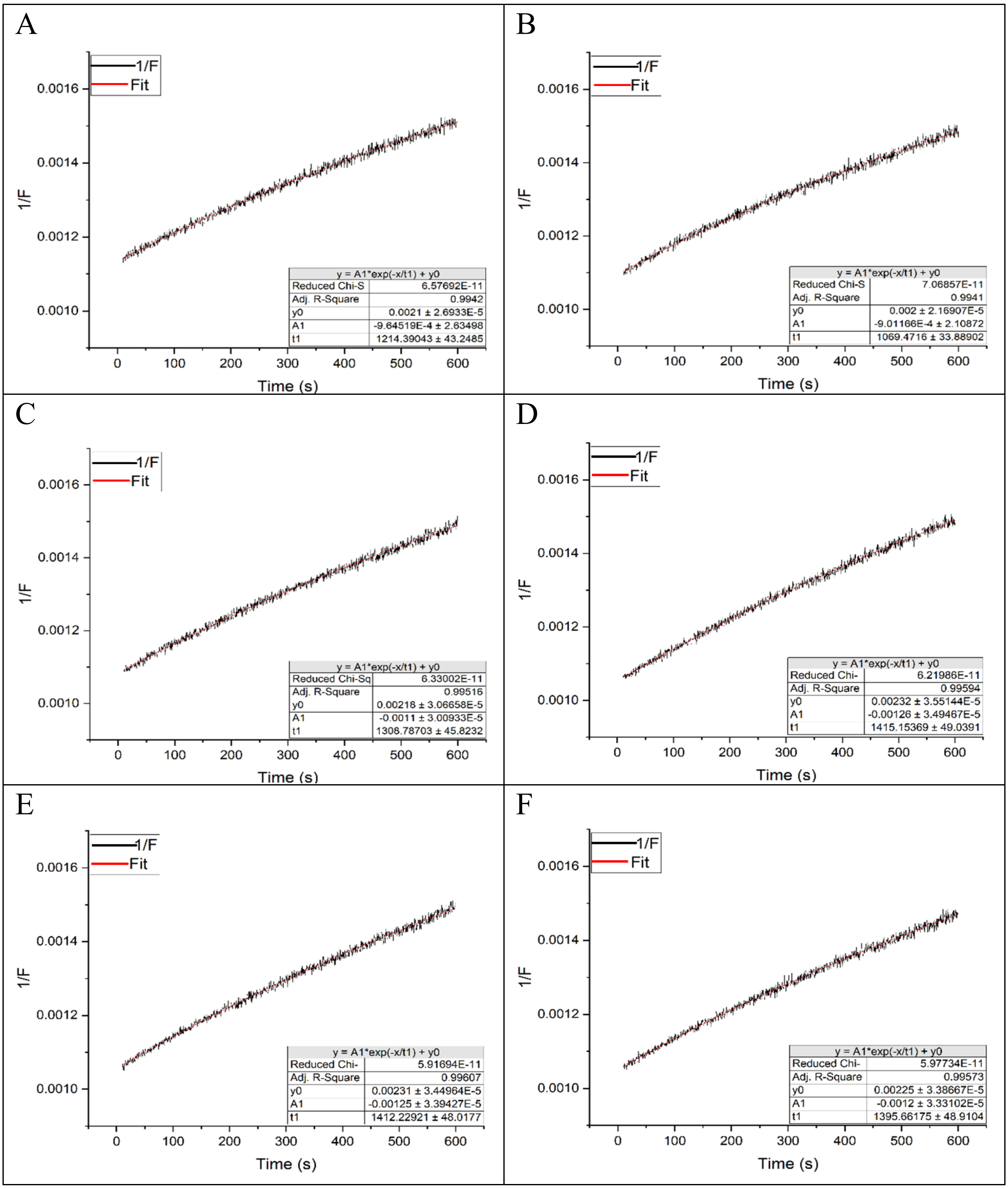

Figure S96. Changes in the reciprocated fluorescence (1/F) over time and corresponding single exponential decay fits for the transport of Cl^- ions into POPC LUVs by resorcin[4]arene **4**, pre-incorporated into the lipid bilayer at 0.05 mol% with respect to lipid.

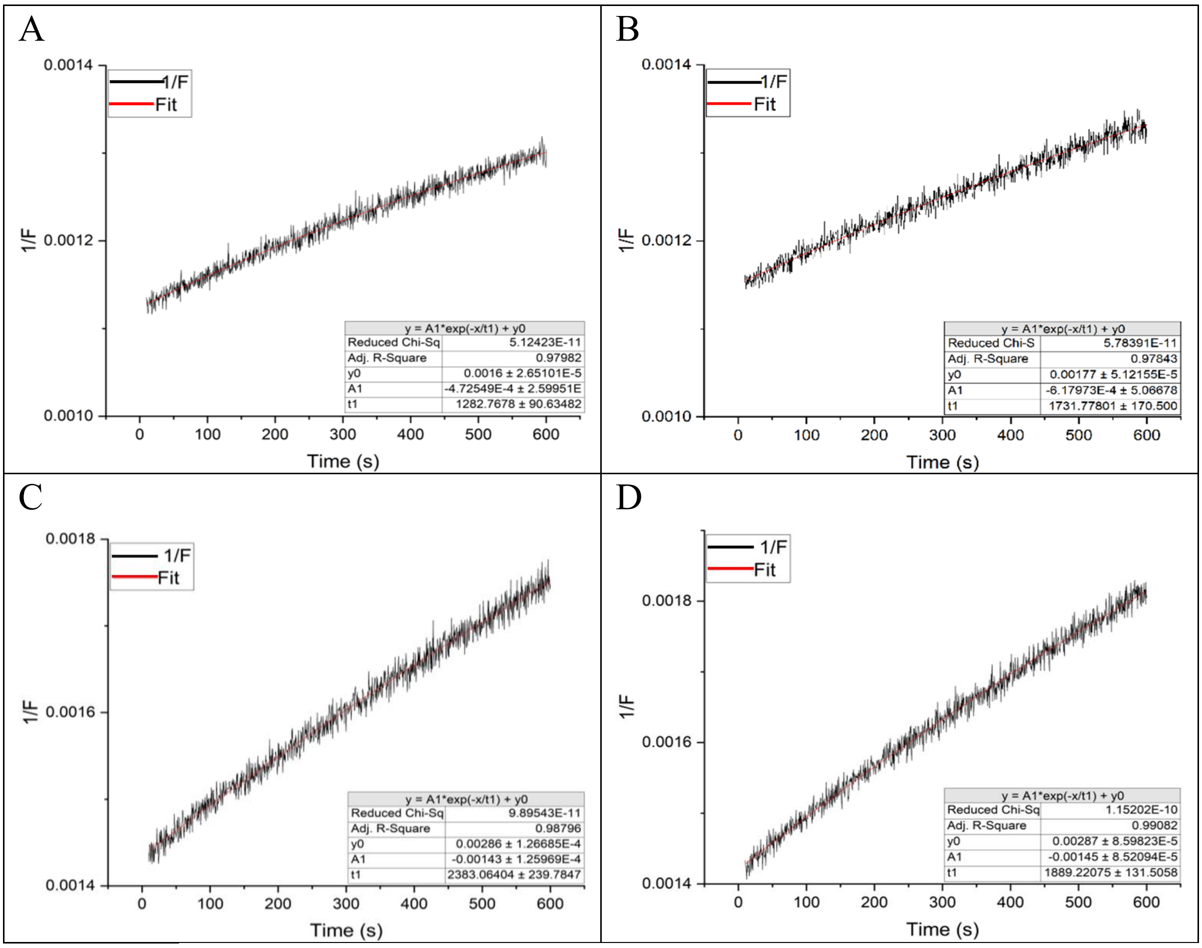

Figure S97. Changes in the reciprocated fluorescence (1/F) over time and corresponding single exponential decay fits for the transport of Cl^- ions into POPC LUVs by resorcin[4]arene **4**, pre-incorporated into the lipid bilayer at 0.025 mol% with respect to lipid.

Fitting Anion Transport Data for Resorcin[4]arene 1: Calculation of Half-Life Time Values:

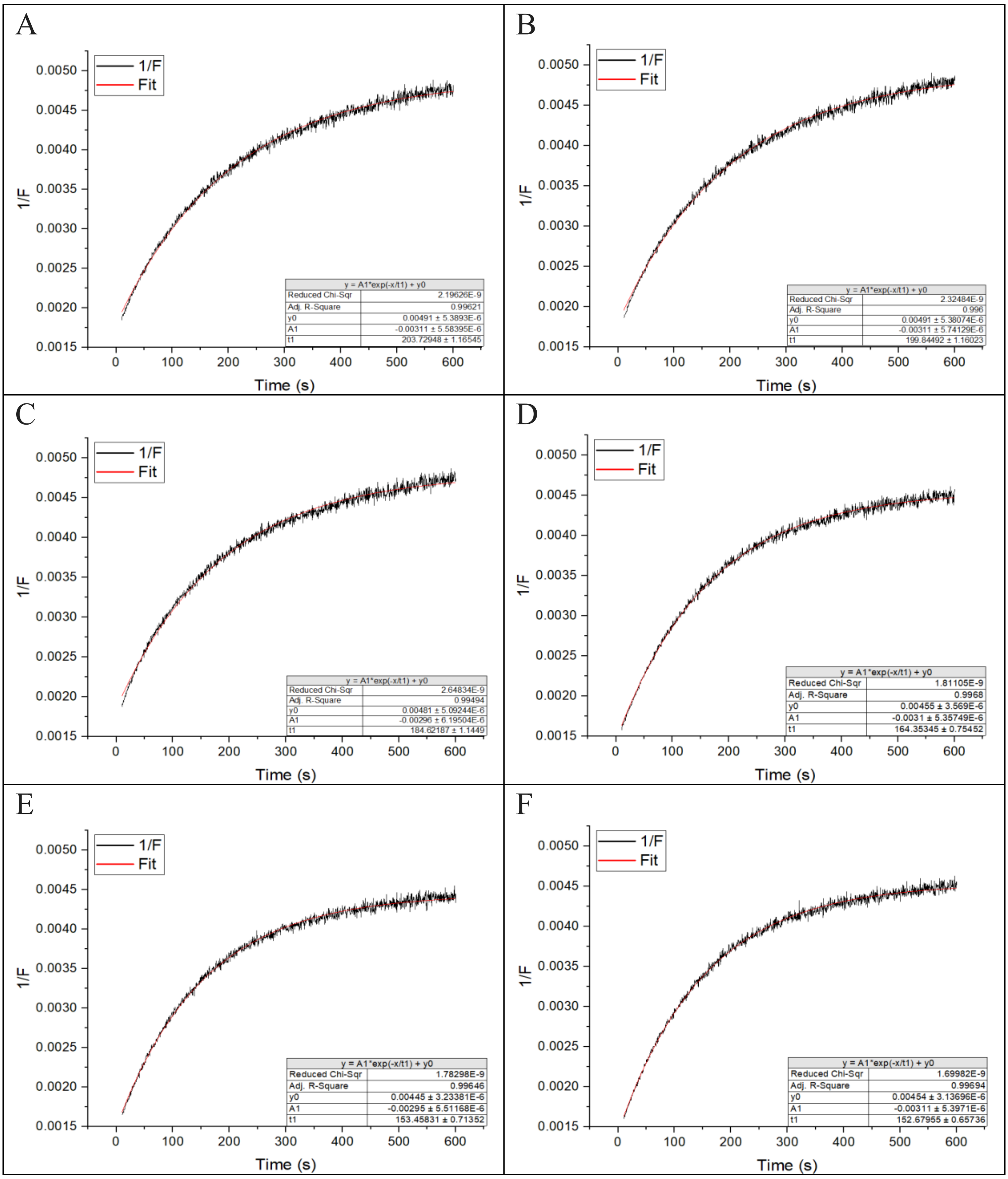

Figure S98. Changes in the reciprocated fluorescence (1/F) over time and corresponding single exponential decay fits for the transport of Cl^- ions into POPC LUVs by resorcin[4]arene **1**, pre-incorporated into the lipid bilayer at 0.025 mol% with respect to lipid.

Fitting Anion Transport Data for Resorcin[4]arene 4: Calculation of Initial Rate Values

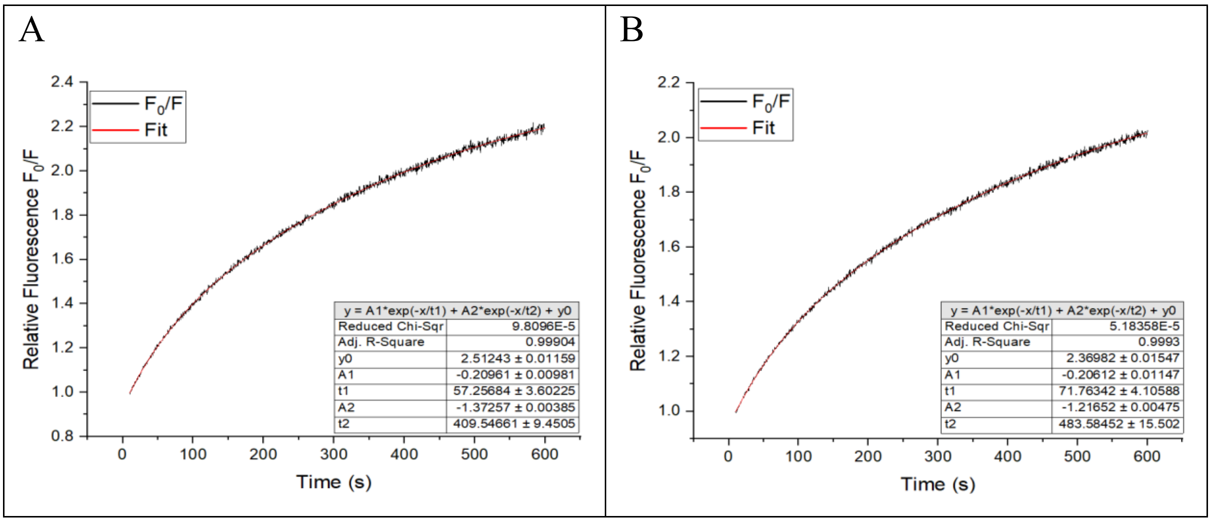

Figure S99. Changes in the normalized reciprocated fluorescence intensity (F_0/F) over time, along with the corresponding double exponential decay fit, for the transport of Cl^- ions into POPC LUVs by resorcin[4]arene **4** pre-incorporated into the lipid bilayer at 0.5 mol% with respect to lipid.

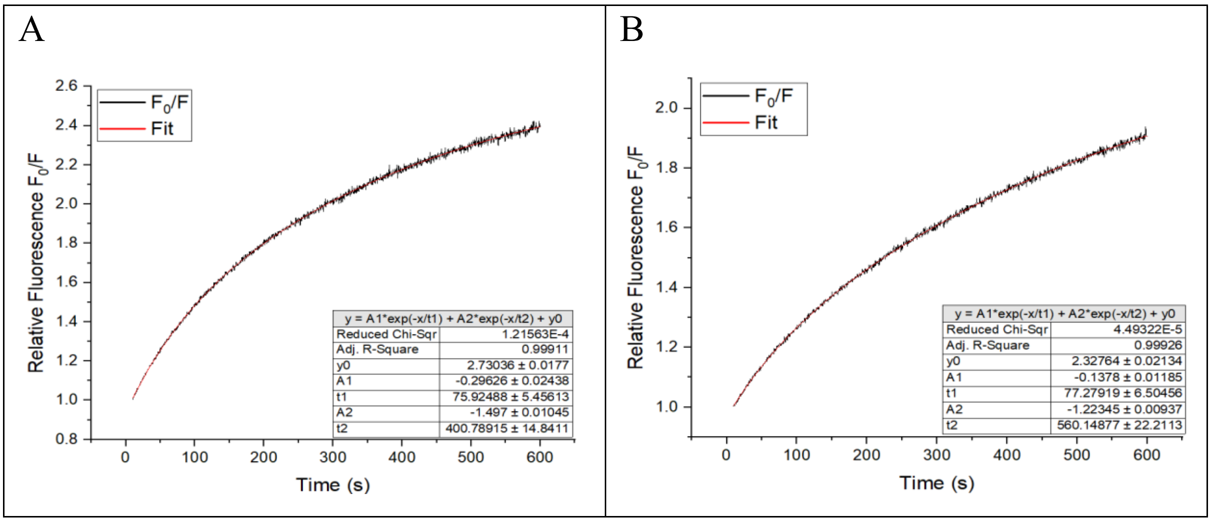

Figure S100. Changes in the normalized reciprocated fluorescence intensity (F_0/F) over time, along with the corresponding double exponential decay fit, for the transport of Cl^- ions into POPC LUVs by resorcin[4]arene **4** pre-incorporated into the lipid bilayer at 0.3 mol% with respect to lipid.

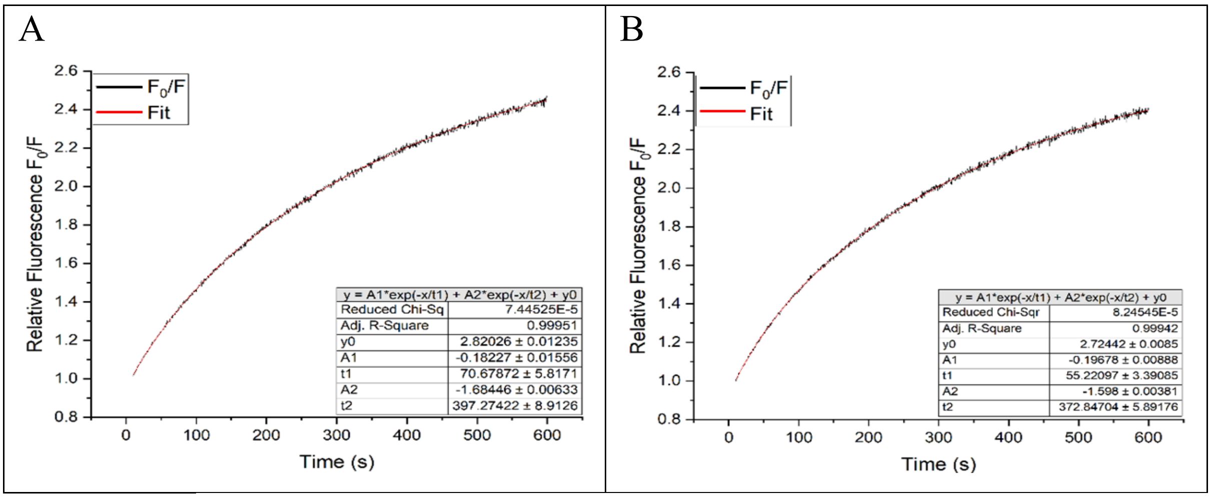

Figure S101. Changes in the normalized reciprocated fluorescence intensity (F_0/F) over time, along with the corresponding double exponential decay fit, for the transport of Cl^- ions into POPOC LUVs by resorcin[4]arene **4** pre-incorporated into the lipid bilayer at 0.25 mol% with respect to lipid.

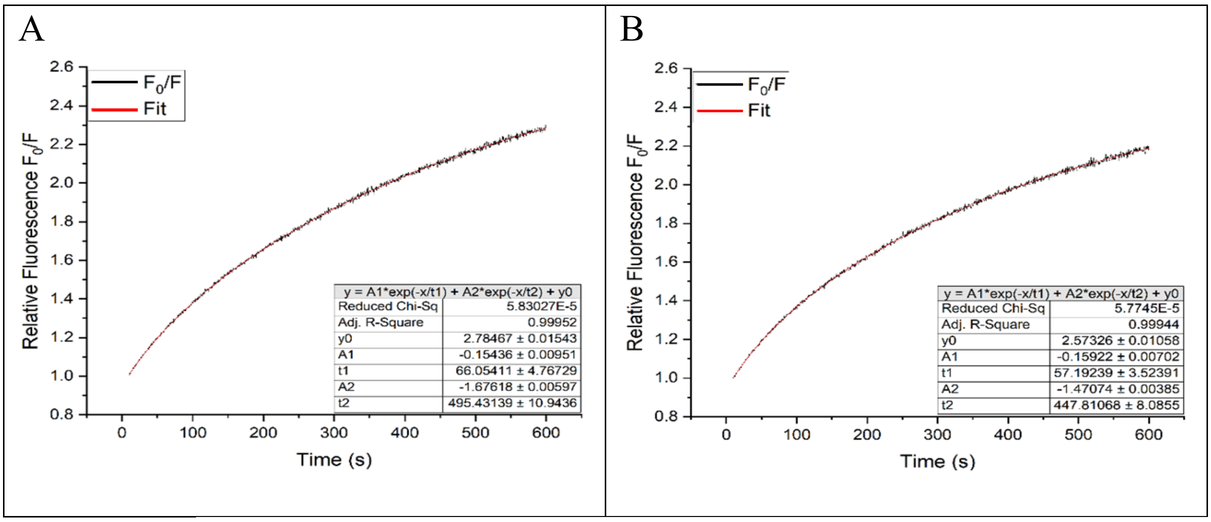

Figure S102. Changes in the normalized reciprocated fluorescence intensity (F_0/F) over time, along with the corresponding double exponential decay fit, for the transport of Cl^- ions into POPC LUVs by resorcin[4]arene **4** pre-incorporated into the lipid bilayer at 0.2 mol% with respect to lipid.

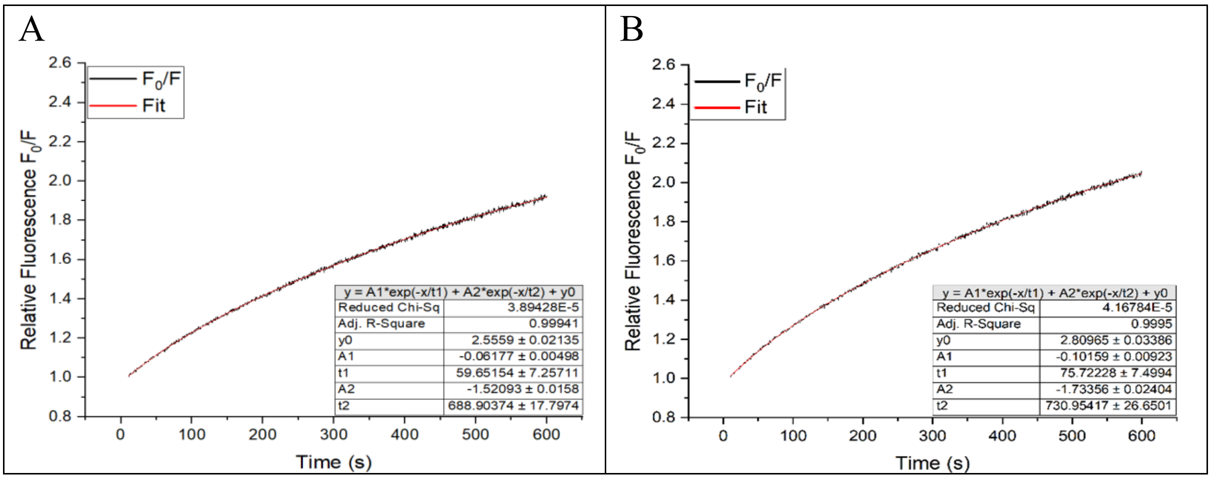

Figure S103. Changes in the normalized reciprocated fluorescence intensity (F_0/F) over time, along with the corresponding double exponential decay fit, for the transport of Cl^- ions into POPC LUVs by resorcin[4]arene **4** pre-incorporated into the lipid bilayer at 0.15 mol% with respect to lipid.

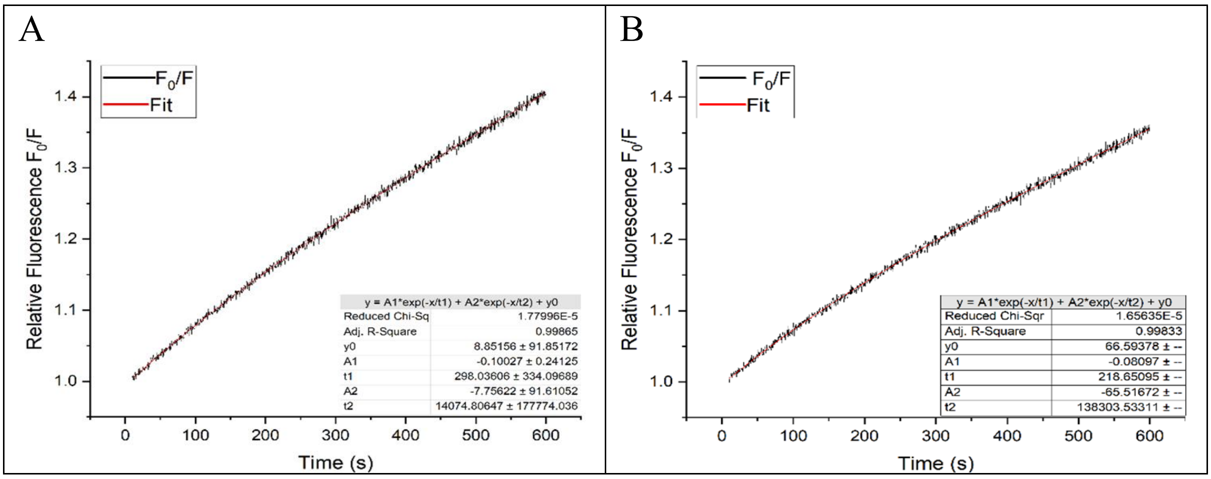

Figure S104. Changes in the normalized reciprocated fluorescence intensity (F_0/F) over time, along with the corresponding double exponential decay fit, for the transport of Cl^- ions into POPC LUVs by resorcin[4]arene **4** pre-incorporated into the lipid bilayer at 0.05 mol% with respect to lipid.

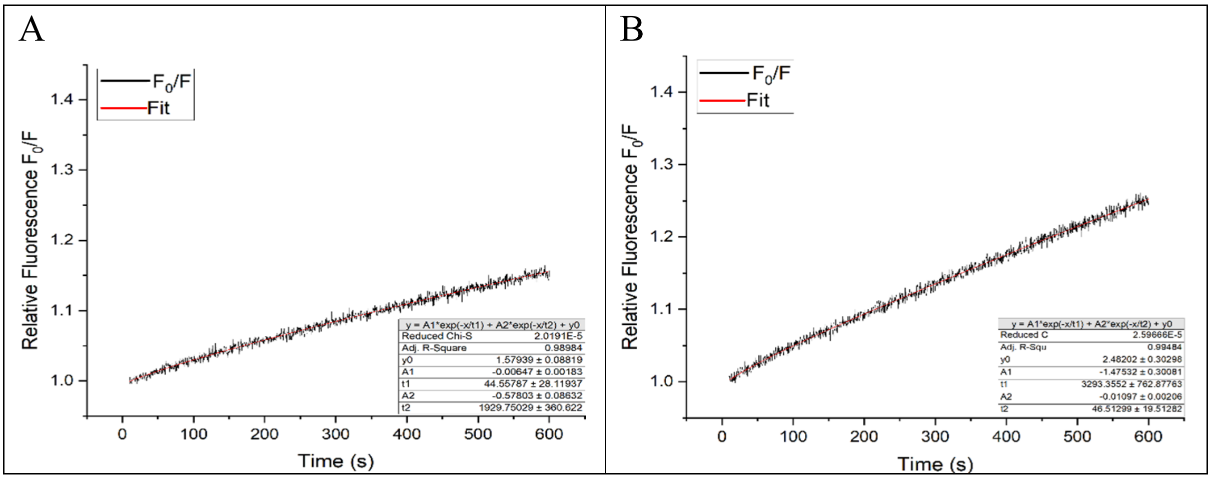

Figure S105. Changes in the normalized reciprocated fluorescence intensity (F_0/F) over time, along with the corresponding double exponential decay fit, for the transport of Cl^- ions into POPC LUVs by resorcin[4]arene **4** pre-incorporated into the lipid bilayer at 0.025 mol% with respect to lipid.

Fitting Anion Transport Data for Resorcin[4]arene 1: Calculation of Initial Rate Values

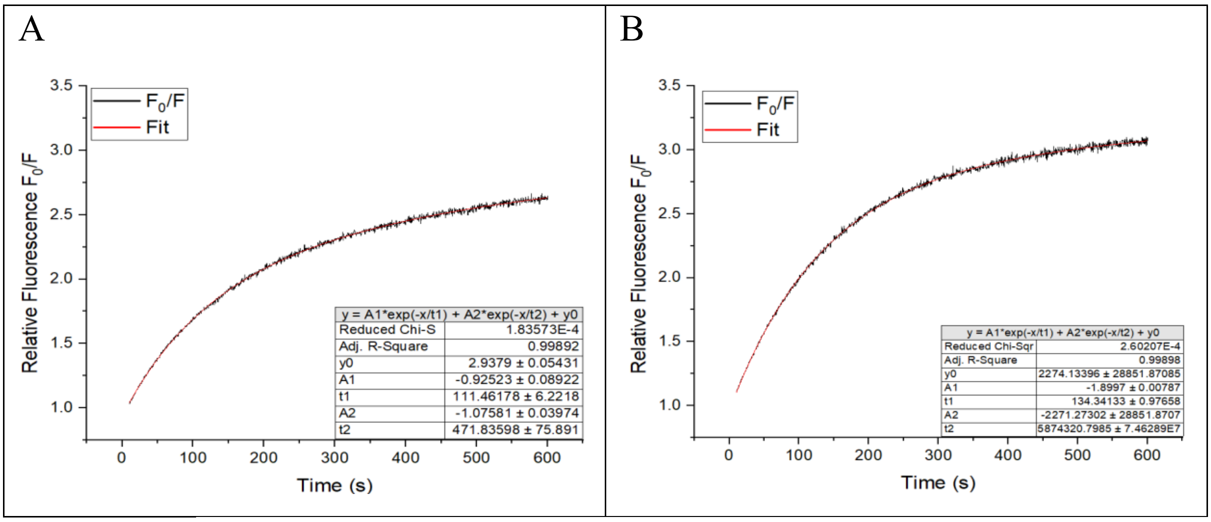

Figure S106. Changes in the normalized reciprocated fluorescence intensity (F_0/F) over time, along with the corresponding double exponential decay fit, for the transport of Cl^- ions into POPC LUVs by resorcin[4]arene **1** pre-incorporated into the lipid bilayer at 0.025 mol% with respect to lipid.

Estimation of log*P*

To estimate the log*P* values of resorcin[4]arenes **1 - 7**, we employed a retention time–based method using reversed-phase HPLC.[12] Seven reference compounds, selected from the OECD guideline list,[12] were used to construct a calibration curve.

Stock solutions (1 mM) of all reference compounds were prepared in THF, while stock solutions of the resorcinarenes were prepared under the conditions outlined in Table S3.

Note: Compound **7** was too insoluble to obtain a good chromatogram under these conditions.

Table S3: Solvents and concentrations of the stock solutions for the HPLC experiment.

**Compound**	**Solvent(s)**	**Concentration**
**1**	THF	1.0 mM
**2**	THF	0.5 mM
**3**	THF	0.5 mM
**4**	THF:acetonitrile (1:1, *v*:*v*)	0.3 mM
**5**	THF	1.0 mM
**6**	THF:water (9:1, *v*:*v*)	0.5 mM

HPLC measurements were performed on a C8 reversed-phase column (100 mm × 4.6 mm) under isocratic conditions, with acetonitrile/water (3:1, *v*:*v*) as the mobile phase. The injection volume was 1 µl, the flow rate was 1 ml/min, and the column temperature was maintained at 40 °C. UV detection was carried out

[12] OECD. *Test No. 117: Partition Coefficient (n-octanol/water), HPLC Method,* OECD,2022. https://doi.org/10.1787/9789264069824-en.

at 254 nm. Uracil (retention time = 1.01 min) was used as a void volume marker to estimate the dead time of the column (t_0).

Note: All the reported retention times in this section represent the average of at least two independent experimental repeats.

Retention times of the reference compounds were used to calculate their capacity factors (k), defined as:

$$k = \frac{t_r - t_0}{t_0}$$

where t_r is the retention time of the analyte and t_0 is the dead time of the column.

Table S4: Retention times (t_r), capacity factors (k), logarithms of k, and literature log*P* values for reference compounds.

**Compound**	**Retention time (minutes)**	**k**	**log k**	**log*P***
**Nitrobenzene**	1.357	0.344	-0.464	1.9
**Naphthalene**	1.747	0.730	-0.137	3.6
**Diphenyl ether**	1.927	0.908	-0.042	4.2
**Phenanthrene**	2.097	1.076	0.032	4.5
**Fluoranthene**	2.327	1.303	0.115	5.1
**Triphenylamine**	3.163	2.132	0.329	5.7
**DDT**	3.857	2.819	0.450	6.5

A linear correlation was observed between the log$P$ values and log k of the reference compounds (Figure S107):

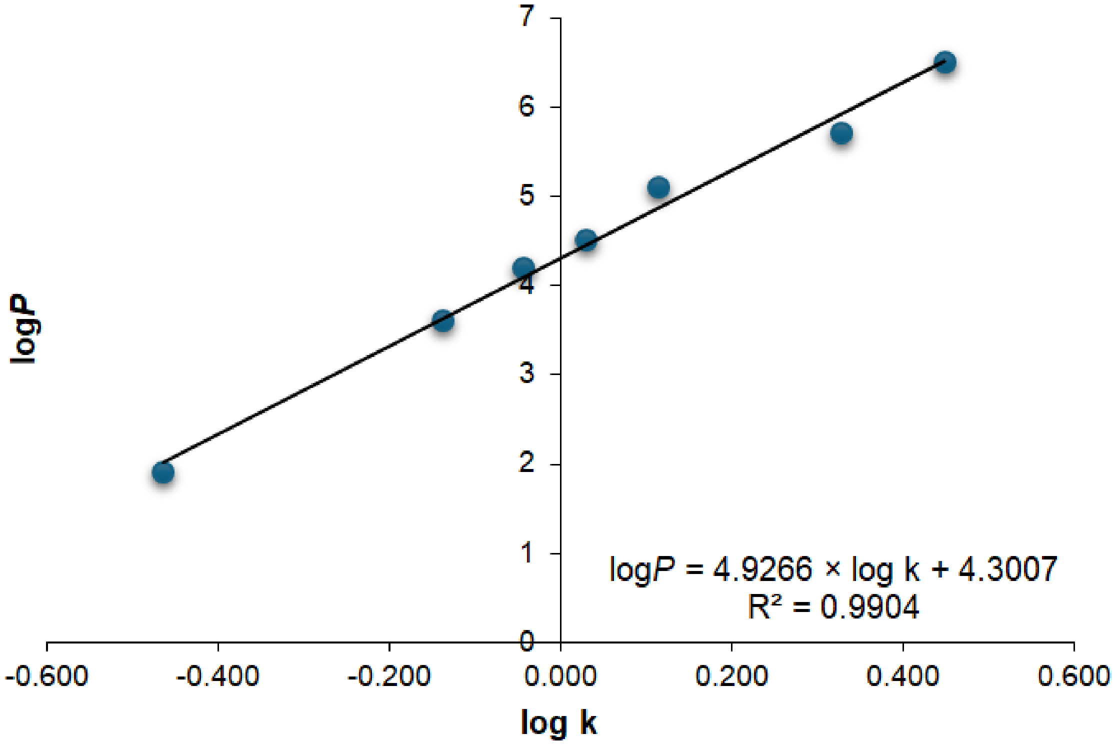

Figure S107. Calibration curve showing the linear relationship between log$P$ values and log k of the standards listed in
Table S4.

This yielded the following calibration equation:

$$\log P = 4.9266 \times \log \mathrm{k} + 4.3007$$

Using this equation, we estimated log$P$ values for compounds **1** - **6** based on their experimental retention times (Table S5).

Table S5: Retention times (t_r), capacity factors (k), logarithms of capacity factors (log k) and estimated log*P* values for resorcinarenes **1** - **6**.

**Resorcin[4]arene**	**Retention time (minutes)**	**k**	**log k**	**Estimated log*P* (from HPLC)**
**1**	84.925	83.084	1.920	13.8
**2**	>100	>98.009	>1.991	>14.1
**3**	16.849	15.6817	1.1954	10.2
**4**	28.047	26.769	1.428	11.3
**5**	4.495	3.451	0.538	7.0
**6**	1.331	0.345	-0.497	1.9

Note: The log*P* values shown in Table S5 exceed the upper range of precision for this method[12] and should be interpreted with caution.

In parallel, log*P* values for resorcinarenes **1** - **6** were estimated computationally using ChemDraw and ChemAxon's Playground[13]. These calculated values, alongside HPLC-derived estimates, are shown below.

[13] *Playground* v1.6.2 https://playground.calculators.cxn.io/

Table S6: Lipophilicity estimates for receptors **1**–**7**.

Receptor	clog*P* (ChemDraw)	clog*P* (Playground)	log*P* (RP-HPLC)
**1**	16.6	12.5	13.8
**2**	20.3	15.8	>14.1[a]
**3**	15.1	11.6	10.2
**4**	15.5	12.1	11.3
**5**	10.7	8.9	7.0
**6**	5.9	5.2	1.9
**7**	12.8	10.7	n.d.[b]

[a] Retention time > 100 min. [b] Not determined due to too low solubility.

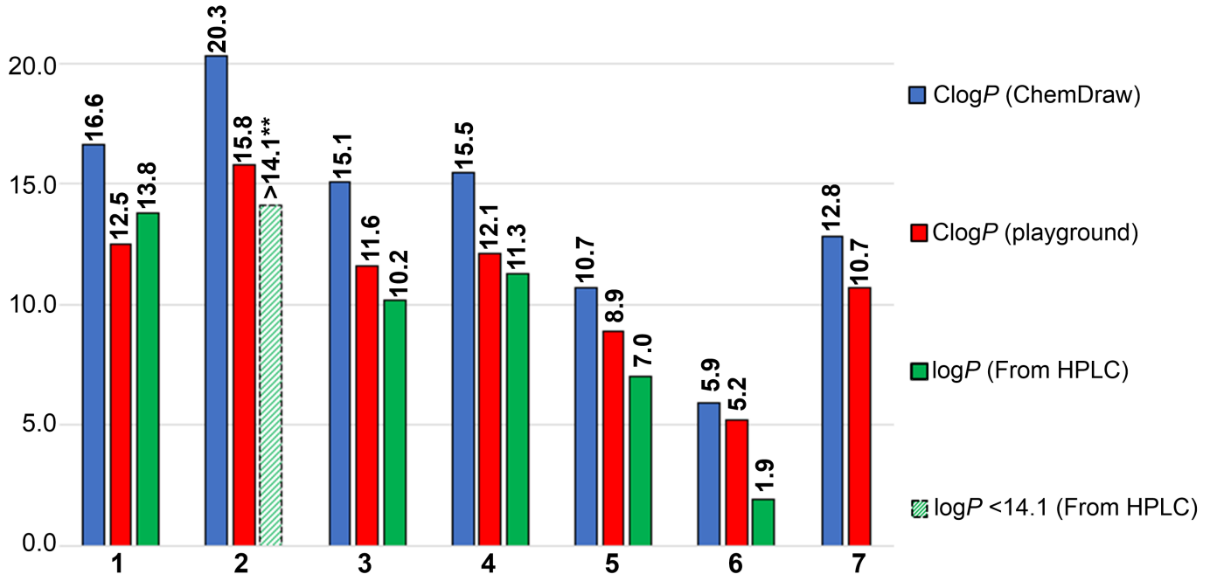

Figure S108. Comparison of log*P* values estimated from RP-HPLC measurements and clog*P* values calculated by ChemDraw and Playground. ** log*P* > 14.1.

8. THEORETICAL CALCULATIONS

All calculations performed within the density functional theory (DFT) approach using Gaussian 16 program suite.[14] Geometry was optimized with the B3LYP functional, employing the 6-31+G(d,p) basis set. For cavitands, solvent effects (THF) were considered within the SMD model. For complexes, calculations were done in a vacuum with D3 empirical dispersion. Atomic coordinates for optimized structures are listed below.

1a

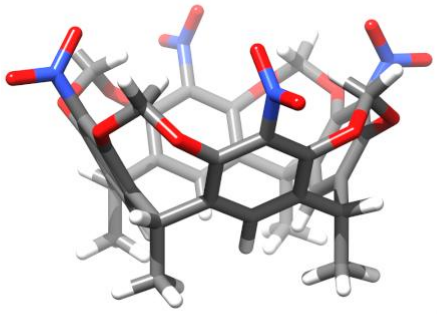

Energy = -2810.9700 Hartree

Number of imaginary frequencies 0

Symbol X Y Z

O 3.11394100 -3.53981800 -0.59530100
O -0.98525700 4.60517700 -0.59457000
O 3.53981800 3.11394100 -0.59530100
O 4.60517700 0.98525700 -0.59457000
O -4.60517700 -0.98525700 -0.59457000
O -3.11394100 3.53981800 -0.59530100
O -3.53981800 -3.11394100 -0.59530100
O 0.98525700 -4.60517700 -0.59457000
C -1.96900900 3.91192300 -1.36010100
H -1.52835200 3.03408600 -1.84041400
H -2.33146100 4.63572500 -2.08845300
C 3.91192300 1.96900900 -1.36010100
H 4.63572500 2.33146100 -2.08845300
H 3.03408600 1.52835200 -1.84041400
C -3.91192300 -1.96900900 -1.36010100
H -3.03408600 -1.52835200 -1.84041400
H -4.63572500 -2.33146100 -2.08845300
C 1.96900900 -3.91192300 -1.36010100
H 1.52835200 -3.03408600 -1.84041400
H 2.33146100 -4.63572500 -2.08845300
O 4.03100900 -1.03416900 -2.83308500
C 3.22017000 0.23332100 1.26205200
N 1.48864000 4.53268200 -1.80404500
C -0.83819000 -2.53262900 1.84464200
H -0.67337800 -2.03459400 2.79283400
C -2.45120100 2.10969100 1.26332700
C 3.24656000 1.63308500 1.87728500
H 4.17866700 2.10017000 1.54616800

[14] M. J. Frisch, G. W. Trucks, H. B. Schlegel, G. E. Scuseria, M. A. Robb, J. R. Cheeseman, G. Scalmani, V. Barone, G. A. Petersson, H. Nakatsuji, X. Li, M. Caricato, A. V. Marenich, J. Bloino, B. G. Janesko, R. Gomperts, B. Mennucci, H. P. Hratchian, J. V. Ortiz, A. F. Izmaylov, J. L. Sonnenberg, D. Williams-Young, F. Ding, F. Lipparini, F. Egidi, J. Goings, B. Peng, A. Petrone, T. Henderson, D. Ranasinghe, V. G. Zakrzewski, J. Gao, N. Rega, G. Zheng, W. Liang, M. Hada, M. Ehara, K. Toyota, R. Fukuda, J. Hasegawa, M. Ishida, T. Nakajima, Y. Honda, O. Kitao, H. Nakai, T. Vreven, K. Throssell, J. A. Montgomery, Jr., J. E. Peralta, F. Ogliaro, M. J. Bearpark, J. J. Heyd, E. N. Brothers, K. N. Kudin, V. N. Staroverov, T. A. Keith, R. Kobayashi, J. Normand, K. Raghavachari, A. P. Rendell, J. C. Burant, S. S. Iyengar, J. Tomasi, M. Cossi, J. M. Millam, M. Klene, C. Adamo, R. Cammi, J. W. Ochterski, R. L. Martin, K. Morokuma, O. Farkas, J. B. Foresman, and D. J. Fox, GAUSSIAN 16 (Revision C.01) Gaussian Inc., Wallingford, CT, 2016.

C -3.10435100 2.31677400 0.03900300
O 2.10969100 5.58992400 -1.75051600
C -0.23332100 3.22017000 1.26205200
C 3.10435100 -2.31677400 0.03900300
C 2.53262900 -0.83819000 1.84464200
H 2.03459400 -0.67337800 2.79283400
N 4.53268200 -1.48864000 -1.80404500
C -0.01180700 3.85925500 0.03363000
N -4.53268200 1.48864000 -1.80404500
C 2.31677400 3.10435100 0.03900300
O -2.10969100 -5.58992400 -1.75051600
C -2.31677400 -3.10435100 0.03900300
O 1.03416900 4.03100900 -2.83308500
C -2.53262900 0.83819000 1.84464200
H -2.03459400 0.67337800 2.79283400
C 3.85925500 0.01180700 0.03363000
N -1.48864000 -4.53268200 -1.80404500
C 0.23332100 -3.22017000 1.26205200
C -3.22017000 -0.23332100 1.26205200
C 0.83819000 2.53262900 1.84464200
H 0.67337800 2.03459400 2.79283400
O -4.03100900 1.03416900 -2.83308500
C -3.81143500 1.26046400 -0.53992500
C 1.63308500 -3.24656000 1.87728500
H 2.10017000 -4.17866700 1.54616800
C -2.10969100 -2.45120100 1.26332700
C 0.01180700 -3.85925500 0.03363000
C -1.26046400 -3.81143500 -0.53992500
C 2.10969100 2.45120100 1.26332700
C -1.63308500 3.24656000 1.87728500
H -2.10017000 4.17866700 1.54616800
O 5.58992400 -2.10969100 -1.75051600
C 3.81143500 -1.26046400 -0.53992500
C 2.45120100 -2.10969100 1.26332700
C -3.24656000 -1.63308500 1.87728500
H -4.17866700 -2.10017000 1.54616800
O -5.58992400 2.10969100 -1.75051600
C -3.85925500 -0.01180700 0.03363000
C 1.26046400 3.81143500 -0.53992500
O -1.03416900 -4.03100900 -2.83308500
C -1.63691300 3.26011600 3.41233400
H -1.08925500 4.13579600 3.77435400
H -2.66555100 3.32590100 3.77996400
H -1.17823500 2.37320900 3.85814900
C 3.26011600 1.63691300 3.41233400
H 4.13579600 1.08925500 3.77435400
H 3.32590100 2.66555100 3.77996400
H 2.37320900 1.17823500 3.85814900
C 1.63691300 -3.26011600 3.41233400
H 1.08925500 -4.13579600 3.77435400
H 2.66555100 -3.32590100 3.77996400
H 1.17823500 -2.37320900 3.85814900
C -3.26011600 -1.63691300 3.41233400
H -4.13579600 -1.08925500 3.77435400
H -3.32590100 -2.66555100 3.77996400
H -2.37320900 -1.17823500 3.85814900

2a

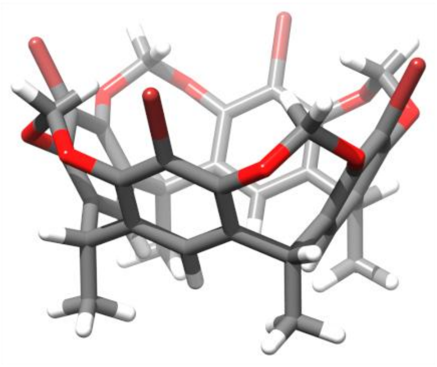

Energy = -12277.490 Hartree

Number of imaginary frequencies 0

Symbol X Y Z

O -2.39326100 -4.06722500 -0.30002500
O 4.06722500 2.39326100 -0.30002500
O 4.06722500 -2.39326100 -0.30002500
O 2.39326100 -4.06722500 -0.30002500
O -2.39326100 4.06722500 -0.30002500
O 2.39326100 4.06722500 -0.30002500
O -4.06722500 2.39326100 -0.30002500
O -4.06722500 -2.39326100 -0.30002500
C 3.11766000 3.11766000 -1.06946600
H 2.43093100 2.43093100 -1.57456700
H 3.69991100 3.69991100 -1.78312800
C 3.11766000 -3.11766000 -1.06946600
H 3.69991100 -3.69991100 -1.78312800
H 2.43093100 -2.43093100 -1.57456700
C -3.11766000 3.11766000 -1.06946600
H -2.43093100 2.43093100 -1.57456700
H -3.69991100 3.69991100 -1.78312800
C -3.11766000 -3.11766000 -1.06946600
H -2.43093100 -2.43093100 -1.57456700
H -3.69991100 -3.69991100 -1.78312800
C 1.22876400 -2.98499900 1.52649800
C -2.66927200 0.00000000 2.11502800
H -2.14224000 0.00000000 3.06178600
C 1.22876400 2.98499900 1.52649800
C 2.56495700 -2.56495700 2.14378700
H 3.30319400 -3.30319400 1.81842400
C 1.21836300 3.66153600 0.29616400
C 2.98499900 1.22876400 1.52649800
C -1.21836300 -3.66153600 0.29616400
C 0.00000000 -2.66927200 2.11502800
H 0.00000000 -2.14224000 3.06178600
C 3.66153600 1.21836300 0.29616400
C 3.66153600 -1.21836300 0.29616400
C -3.66153600 1.21836300 0.29616400
C 0.00000000 2.66927200 2.11502800
H 0.00000000 2.14224000 3.06178600
C 1.21836300 -3.66153600 0.29616400
C -2.98499900 -1.22876400 1.52649800
C -1.22876400 2.98499900 1.52649800
C 2.66927200 0.00000000 2.11502800
H 2.14224000 0.00000000 3.06178600
C 0.00000000 3.98834200 -0.31137800
C -2.56495700 -2.56495700 2.14378700
H -3.30319400 -3.30319400 1.81842400
C -2.98499900 1.22876400 1.52649800
C -3.66153600 -1.21836300 0.29616400
C -3.98834200 0.00000000 -0.31137800
C 2.98499900 -1.22876400 1.52649800
C 2.56495700 2.56495700 2.14378700
H 3.30319400 3.30319400 1.81842400
C 0.00000000 -3.98834200 -0.31137800
C -1.22876400 -2.98499900 1.52649800
C -2.56495700 2.56495700 2.14378700
H -3.30319400 3.30319400 1.81842400
C -1.21836300 3.66153600 0.29616400
C 3.98834200 0.00000000 -0.31137800
C -2.56892600 -2.56892600 3.67905100
H -3.56762900 -2.31423000 4.04790400
H -1.86128800 -1.86128800 4.11979300
C 2.56892600 -2.56892600 3.67905100
H 2.31423000 -3.56762900 4.04790400
H 1.86128800 -1.86128800 4.11979300
C -2.56892600 2.56892600 3.67905100
H -2.31423000 3.56762900 4.04790400
H -1.86128800 1.86128800 4.11979300
C 2.56892600 2.56892600 3.67905100
H 3.56762900 2.31423000 4.04790400
H 1.86128800 1.86128800 4.11979300

H -3.56762900 2.31423000 4.04790400

H -2.31423000 -3.56762900 4.04790400

H 3.56762900 -2.31423000 4.04790400

H 2.31423000 3.56762900 4.04790400

Br 0.00000000 -4.85075500 -2.00433700

Br 0.00000000 4.85075500 -2.00433700

Br -4.85075500 0.00000000 -2.00433700

Br 4.85075500 0.00000000 -2.00433700

3a

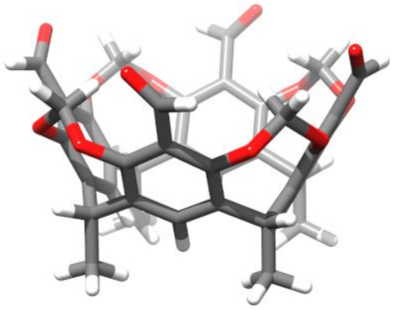

Energy = -2446.2716 Hartree

Number of imaginary frequencies 0

Symbol X Y Z

O 3.77355700 -2.85221300 -0.72064900

O -1.91646100 4.31787800 -0.68581900

O 2.85221300 3.77355700 -0.72064900

O 4.31787800 1.91646100 -0.68581900

O -4.31787800 -1.91646100 -0.68581900

O -3.77355700 2.85221300 -0.72064900

O -2.85221300 -3.77355700 -0.72064900

O 1.91646100 -4.31787800 -0.68581900

C -2.74031000 3.48416500 -1.47873900

H -2.14380900 2.73261400 -2.00414100

H -3.25625600 4.14550800 -2.17360500

C 3.48416500 2.74031000 -1.47873900

H 4.14550800 3.25625600 -2.17360500

H 2.73261400 2.14380900 -2.00414100

C -3.48416500 -2.74031000 -1.47873900

H -2.73261400 -2.14380900 -2.00414100

H -4.14550800 -3.25625600 -2.17360500

C 2.74031000 -3.48416500 -1.47873900

H 2.14380900 -2.73261400 -2.00414100

H 3.25625600 -4.14550800 -2.17360500

C 3.10698600 0.86933200 1.10080200

C -0.31676700 -2.64150500 1.68067200

H -0.25687300 -2.10839500 2.62279300

C -2.82654700 1.57717900 1.10087000

C 2.85182100 2.24637900 1.71805000

H 3.67393200 2.88736400 1.38871900

C -3.50959300 1.63330200 -0.12286400

C -0.86933200 3.10698600 1.10080200

C 3.50959300 -1.63330200 -0.12286400

C 2.64150500 -0.31676700 1.68067200

H 2.10839500 -0.25687300 2.62279300

C -0.77883700 3.77355700 -0.12872200

C 1.63330200 3.50959300 -0.12286400

C -1.63330200 -3.50959300 -0.12286400

C -2.64150500 0.31676700 1.68067200

H -2.10839500 0.25687300 2.62279300

C 3.77355700 0.77883700 -0.12872200

C 0.86933200 -3.10698600 1.10080200

C -3.10698600 -0.86933200 1.10080200

C 0.31676700 2.64150500 1.68067200

H 0.25687300 2.10839500 2.62279300

C -3.98657700 0.46588100 -0.76001000

C 2.24637900 -2.85182100 1.71805000

H 2.88736400 -3.67393200 1.38871900

C -1.57717900 -2.82654700 1.10087000

C 0.77883700 -3.77355700 -0.12872200

C -0.46588100 -3.98657700 -0.76001000

C 1.57717900 2.82654700 1.10087000

C -2.24637900 2.85182100 1.71805000

H -2.88736400 3.67393200 1.38871900

C 3.98657700 -0.46588100 -0.76001000

C 2.82654700 -1.57717900 1.10087000

C -2.85182100 -2.24637900 1.71805000

H -3.67393200 -2.88736400 1.38871900

C -3.77355700 -0.77883700 -0.12872200

C 0.46588100 3.98657700 -0.76001000

C 2.25638900 -2.86031000 3.25373900

H 1.87224700 -3.81665100 3.62271400

H 1.65440000 -2.06486300 3.70209800

C 2.86031000 2.25638900 3.25373900

H 3.81665100 1.87224700 3.62271400

H 2.06486300 1.65440000 3.70209800

C -2.86031000 -2.25638900 3.25373900

H -3.81665100 -1.87224700 3.62271400

H -2.06486300 -1.65440000 3.70209800

C -2.25638900 2.86031000 3.25373900

H -1.87224700 3.81665100 3.62271400

H -1.65440000 2.06486300 3.70209800

H -2.74353600 -3.28211500 3.61803900

H 3.28211500 -2.74353600 3.61803900

H 2.74353600 3.28211500 3.61803900

H -3.28211500 2.74353600 3.61803900

C -0.60140600 -4.69881900 -2.05386400

O 0.28101200 -4.82028700 -2.89336800

H -1.59976400 -5.13014200 -2.23753800

C 4.69881900 -0.60140600 -2.05386400

O 4.82028700 0.28101200 -2.89336800

H 5.13014200 -1.59976400 -2.23753800

C -4.69881900 0.60140600 -2.05386400

O -4.82028700 -0.28101200 -2.89336800

H -5.13014200 1.59976400 -2.23753800

C 0.60140600 4.69881900 -2.05386400

O -0.28101200 4.82028700 -2.89336800

H 1.59976400 5.13014200 -2.23753800

4a

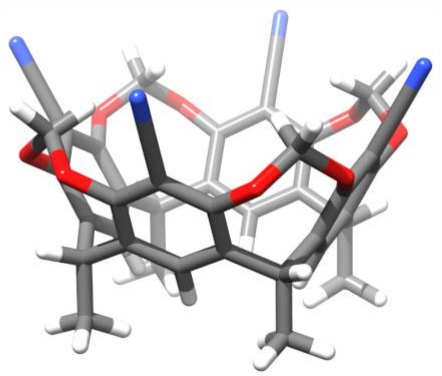

Energy = -2361.9521 Hartree

Number of imaginary frequencies 0

Symbol	X	Y	Z
O	-2.38684600	-4.06865000	-0.78727500
O	4.06865000	2.38684600	-0.78727500
O	4.06865000	-2.38684600	-0.78727500
O	2.38684600	-4.06865000	-0.78727500
O	-2.38684600	4.06865000	-0.78727500
O	2.38684600	4.06865000	-0.78727500
O	-4.06865000	2.38684600	-0.78727500
O	-4.06865000	-2.38684600	-0.78727500
C	3.11373400	3.11373400	-1.55486100
H	2.42715800	2.42715800	-2.05989700
H	3.69641500	3.69641500	-2.26643600
C	3.11373400	-3.11373400	-1.55486100
H	3.69641500	-3.69641500	-2.26643600
H	2.42715800	-2.42715800	-2.05989700
C	-3.11373400	3.11373400	-1.55486100
H	-2.42715800	2.42715800	-2.05989700
H	-3.69641500	3.69641500	-2.26643600
C	-3.11373400	-3.11373400	-1.55486100
H	-2.42715800	-2.42715800	-2.05989700
H	-3.69641500	-3.69641500	-2.26643600
C	1.23379600	-2.98933100	1.05132500
C	-2.67079100	0.00000000	1.63185900
H	-2.14473200	0.00000000	2.57970700
C	1.23379600	2.98933100	1.05132500
C	2.56705300	-2.56705300	1.67112100
H	3.30622900	-3.30622900	1.34933700
C	1.22109700	3.66829400	-0.17307500
C	2.98933100	1.23379600	1.05132500
C	-1.22109700	-3.66829400	-0.17307500
C	0.00000000	-2.67079100	1.63185900
H	0.00000000	-2.14473200	2.57970700
C	3.66829400	1.22109700	-0.17307500
C	3.66829400	-1.22109700	-0.17307500
C	-3.66829400	1.22109700	-0.17307500
C	0.00000000	2.67079100	1.63185900
H	0.00000000	2.14473200	2.57970700
C	1.22109700	-3.66829400	-0.17307500
C	-2.98933100	-1.23379600	1.05132500
C	-1.23379600	2.98933100	1.05132500
C	2.67079100	0.00000000	1.63185900
H	2.14473200	0.00000000	2.57970700
C	0.00000000	4.02330200	-0.78082700
C	-2.56705300	-2.56705300	1.67112100
H	-3.30622900	-3.30622900	1.34933700
C	-2.98933100	1.23379600	1.05132500
C	-3.66829400	-1.22109700	-0.17307500
C	-4.02330200	0.00000000	-0.78082700
C	2.98933100	-1.23379600	1.05132500
C	2.56705300	2.56705300	1.67112100
H	3.30622900	3.30622900	1.34933700
C	0.00000000	-4.02330200	-0.78082700
C	-1.23379600	-2.98933100	1.05132500
C	-2.56705300	2.56705300	1.67112100
H	-3.30622900	3.30622900	1.34933700
C	-1.22109700	3.66829400	-0.17307500
C	4.02330200	0.00000000	-0.78082700
C	4.73267700	0.00000000	-2.02172000
C	0.00000000	4.73267700	-2.02172000
C	-4.73267700	0.00000000	-2.02172000
C	0.00000000	-4.73267700	-2.02172000
N	-5.30343300	0.00000000	-3.03607500
N	0.00000000	5.30343300	-3.03607500
N	5.30343300	0.00000000	-3.03607500
N	0.00000000	-5.30343300	-3.03607500
C	-2.56573500	-2.56573500	3.20625100
H	-3.56299200	-2.31003200	3.57751100
H	-2.31003200	-3.56299200	3.57751100
H	-1.85627500	-1.85627500	3.64124600

C 2.56573500 -2.56573500 3.20625100
H 2.31003200 -3.56299200 3.57751100
H 3.56299200 -2.31003200 3.57751100
H 1.85627500 -1.85627500 3.64124600
C -2.56573500 2.56573500 3.20625100
H -2.31003200 3.56299200 3.57751100
H -3.56299200 2.31003200 3.57751100
H -1.85627500 1.85627500 3.64124600
C 2.56573500 2.56573500 3.20625100
H 3.56299200 2.31003200 3.57751100
H 2.31003200 3.56299200 3.57751100
H 1.85627500 1.85627500 3.64124600

6_{out}

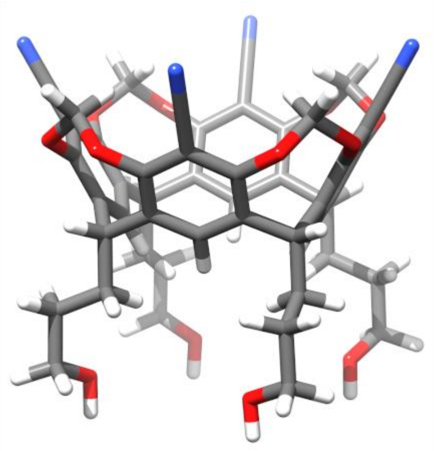

Energy = -2977.3765 Hartree

Number of imaginary frequencies 0

Symbol X Y Z

O 0.64577800 4.67529200 -1.85918700

O -2.84208100 -3.76033300 -1.86763300

O -4.67529200 0.64577800 -1.85918700

O -3.76033300 2.84208100 -1.86763300

O 3.76033300 -2.84208100 -1.86763300

O -0.64577800 -4.67529200 -1.85918700

O 4.67529200 -0.64577800 -1.85918700

O 2.84208100 3.76033300 -1.86763300

C -1.68059500 -4.07339300 -2.63107600

H -1.30624400 -3.17967600 -3.14033000

H -1.99715300 -4.83819800 -3.33860600

C -4.07339300 1.68059500 -2.63107600

H -4.83819800 1.99715300 -3.33860600

H -3.17967600 1.30624400 -3.14033000

C 4.07339300 -1.68059500 -2.63107600

H 3.17967600 -1.30624400 -3.14033000

H 4.83819800 -1.99715300 -3.33860600

C 1.68059500 4.07339300 -2.63107600

H 1.30624400 3.17967600 -3.14033000

H 1.99715300 4.83819800 -3.33860600

C -2.28162400 2.28162400 -0.02879600

C 2.45788800 1.01970800 0.55340600

H 1.97011200 0.81852600 1.50071000

C 0.00506800 -3.23317600 -0.02271900

C -3.35943700 1.39189000 0.59038600

H -4.31942200 1.79235600 0.24951900

C 0.27720400 -3.85548100 -1.24694300

C -2.28162400 -2.28162400 -0.02879600

C -0.27720400 3.85548100 -1.24694300

C -1.01970800 2.45788800 0.55340600

H -0.81852600 1.97011200 1.50071000

C -2.91494700 -2.52954200 -1.25294500

C -3.85548100 -0.27720400 -1.24694300

C 3.85548100 0.27720400 -1.24694300

C 1.01970800 -2.45788800 0.55340600

H 0.81852600 -1.97011200 1.50071000

C -2.52954200 2.91494700 -1.25294500

C 2.28162400 2.28162400 -0.02879600

C 2.28162400 -2.28162400 -0.02879600

C -2.45788800 -1.01970800 0.55340600

H -1.97011200 -0.81852600 1.50071000

C 1.53831100 -3.71200800 -1.85930500

C 1.39189000 3.35943700 0.59038600

H 1.79235600 4.31942200 0.24951900

C 3.23317600 0.00506800 -0.02271900

C 2.91494700 2.52954200 -1.25294500

C 3.71200800 1.53831100 -1.85930500

C -3.23317600 -0.00506800 -0.02271900

C -1.39189000 -3.35943700 0.59038600

H -1.79235600 -4.31942200 0.24951900

C -1.53831100 3.71200800 -1.85930500

C -0.00506800 3.23317600 -0.02271900

C 3.35943700 -1.39189000 0.59038600

H 4.31942200 -1.79235600 0.24951900

C 2.52954200 -2.91494700 -1.25294500

C -3.71200800 -1.53831100 -1.85930500

C -4.36662500 -1.80847200 -3.10077700

C 1.80847200 -4.36662500 -3.10077700

C 4.36662500 1.80847200 -3.10077700

C -1.80847200 4.36662500 -3.10077700

N 4.89402600 2.02558100 -4.11551100

N 2.02558100 -4.89402600 -4.11551100

N -4.89402600 -2.02558100 -4.11551100

N -2.02558100 4.89402600 -4.11551100

C 1.42210600 3.38209000 2.13140900

H 2.47317900 3.34643700 2.44305300

H 0.94566100 2.49350000 2.55551500
C -3.38209000 1.42210600 2.13140900
H -3.34643700 2.47317900 2.44305300
H -2.49350000 0.94566100 2.55551500
C 3.38209000 -1.42210600 2.13140900
H 3.34643700 -2.47317900 2.44305300
H 2.49350000 -0.94566100 2.55551500
C -1.42210600 -3.38209000 2.13140900
H -2.47317900 -3.34643700 2.44305300
H -0.94566100 -2.49350000 2.55551500
C -0.77224300 -4.64283300 2.71872000
H 0.27513000 -4.72118200 2.40108700
H -1.28767900 -5.53245300 2.33343800
C 0.77224300 4.64283300 2.71872000
H -0.27513000 4.72118200 2.40108700
H 1.28767900 5.53245300 2.33343800
C -4.64283300 0.77224300 2.71872000
H -4.72118200 -0.27513000 2.40108700
H -5.53245300 1.28767900 2.33343800
C 4.64283300 -0.77224300 2.71872000
H 4.72118200 0.27513000 2.40108700
H 5.53245300 -1.28767900 2.33343800
C -0.82097500 -4.69917500 4.23907800
H -1.85716700 -4.59136500 4.59215400
H -0.44370500 -5.67162800 4.58466400
C 0.82097500 4.69917500 4.23907800
H 1.85716700 4.59136500 4.59215400
H 0.44370500 5.67162800 4.58466400
C -4.69917500 0.82097500 4.23907800
H -4.59136500 1.85716700 4.59215400
H -5.67162800 0.44370500 4.58466400
C 4.69917500 -0.82097500 4.23907800
H 4.59136500 -1.85716700 4.59215400
H 5.67162800 -0.44370500 4.58466400
O -0.00632100 -3.63926600 4.77051100
H -0.07475700 -3.65579100 5.73521400
O 0.00632100 3.63926600 4.77051100
H 0.07475700 3.65579100 5.73521400
O -3.63926600 0.00632100 4.77051100
H -3.65579100 0.07475700 5.73521400
O 3.63926600 -0.00632100 4.77051100
H 3.65579100 -0.07475700 5.73521400

6in

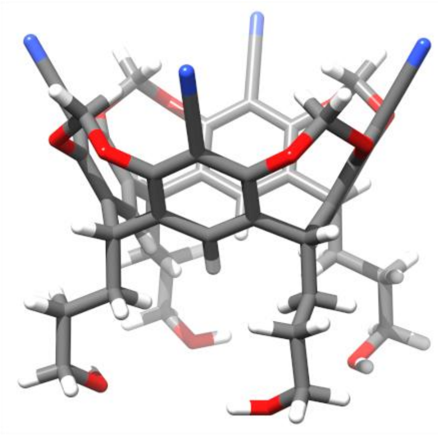

Energy = -2977.3755 Hartree

Number of imaginary frequencies 0

Symbol X Y Z

O 4.67573400 -0.64535700 -1.86556900

O -3.75988000 2.84125400 -1.87390200

O 0.64535700 4.67573400 -1.86556900

O 2.84125400 3.75988000 -1.87390200

O -2.84125400 -3.75988000 -1.87390200

O -4.67573400 0.64535700 -1.86556900

O -0.64535700 -4.67573400 -1.86556900

O 3.75988000 -2.84125400 -1.87390200

C -4.07300400 1.67984300 -2.63757800

H -3.17927000 1.30489700 -3.14618800

H -4.83752100 1.99636000 -3.34526300

C 1.67984300 4.07300400 -2.63757800

H 1.99636000 4.83752100 -3.34526300

H 1.30489700 3.17927000 -3.14618800

C -1.67984300 -4.07300400 -2.63757800

H -1.30489700 -3.17927000 -3.14618800

H -1.99636000 -4.83752100 -3.34526300

C 4.07300400 -1.67984300 -2.63757800

H 3.17927000 -1.30489700 -3.14618800

H 4.83752100 -1.99636000 -3.34526300

C 2.28089100 2.28089100 -0.03567300

C 1.01906100 -2.45707400 0.54640300

H 0.81568300 -1.96593300 1.49195200

C -3.23385900 -0.00526600 -0.02898200

C 1.39147000 3.35939900 0.58310400

H 1.79210800 4.31909200 0.24230800

C -3.85666100 -0.27757100 -1.25304300

C -2.28089100 2.28089100 -0.03567300

C 3.85666100 0.27757100 -1.25304300

C 2.45707400 1.01906100 0.54640300

H 1.96593300 0.81568300 1.49195200

C -2.52930000 2.91501600 -1.25952300

C -0.27757100 3.85666100 -1.25304300

C 0.27757100 -3.85666100 -1.25304300

C -2.45707400 -1.01906100 0.54640300

H -1.96593300 -0.81568300 1.49195200

C 2.91501600 2.52930000 -1.25952300

C 2.28089100 -2.28089100 -0.03567300

C -2.28089100 -2.28089100 -0.03567300

C -1.01906100 2.45707400 0.54640300

H -0.81568300 1.96593300 1.49195200

C -3.71297800 -1.53871000 -1.86525400

C 3.35939900 -1.39147000 0.58310400

H 4.31909200 -1.79210800 0.24230800

C 0.00526600 -3.23385900 -0.02898200

C 2.52930000 -2.91501600 -1.25952300

C 1.53871000 -3.71297800 -1.86525400

C -0.00526600 3.23385900 -0.02898200

C -3.35939900 1.39147000 0.58310400

H -4.31909200 1.79210800 0.24230800

C 3.71297800 1.53871000 -1.86525400

C 3.23385900 0.00526600 -0.02898200

C -1.39147000 -3.35939900 0.58310400

H -1.79210800 -4.31909200 0.24230800

C -2.91501600 -2.52930000 -1.25952300

C -1.53871000 3.71297800 -1.86525400

C -1.80929400 4.36839600 -3.10632900

C -4.36839600 -1.80929400 -3.10632900

C 1.80929400 -4.36839600 -3.10632900

C 4.36839600 1.80929400 -3.10632900

N 2.02629100 -4.89621300 -4.12075000

N -4.89621300 -2.02629100 -4.12075000

N -2.02629100 4.89621300 -4.12075000

N 4.89621300 2.02629100 -4.12075000

C 3.38146700 -1.43056700 2.12375400

H 3.37211500 -2.48624700 2.42302100

H 2.47771200 -0.98382000 2.54949300
C 1.43056700 3.38146700 2.12375400
H 2.48624700 3.37211500 2.42302100
H 0.98382000 2.47771200 2.54949300
C -1.43056700 -3.38146700 2.12375400
H -2.48624700 -3.37211500 2.42302100
H -0.98382000 -2.47771200 2.54949300
C -3.38146700 1.43056700 2.12375400
H -3.37211500 2.48624700 2.42302100
H -2.47771200 0.98382000 2.54949300
C -4.62102200 0.75864400 2.72862800
H -4.65202200 -0.30668600 2.46247400
H -5.52839300 1.21095100 2.30450800
C 4.62102200 -0.75864400 2.72862800
H 4.65202200 0.30668600 2.46247400
H 5.52839300 -1.21095100 2.30450800
C 0.75864400 4.62102200 2.72862800
H -0.30668600 4.65202200 2.46247400
H 1.21095100 5.52839300 2.30450800
C -0.75864400 -4.62102200 2.72862800
H 0.30668600 -4.65202200 2.46247400
H -1.21095100 -5.52839300 2.30450800
C -4.70210400 0.89608000 4.24860400
H -4.66306200 1.95223600 4.53697000
H -5.65667600 0.48846600 4.60893600
C 4.70210400 -0.89608000 4.24860400
H 4.66306200 -1.95223600 4.53697000
H 5.65667600 -0.48846600 4.60893600
C 0.89608000 4.70210400 4.24860400
H 1.95223600 4.66306200 4.53697000
H 0.48846600 5.65667600 4.60893600
C -0.89608000 -4.70210400 4.24860400
H -1.95223600 -4.66306200 4.53697000
H -0.48846600 -5.65667600 4.60893600
O -3.60708400 0.27577600 4.94049400
H -3.68954700 -0.68414300 4.84418100
O 3.60708400 -0.27577600 4.94049400
H 3.68954700 0.68414300 4.84418100
O 0.27577600 3.60708400 4.94049400
H -0.68414300 3.68954700 4.84418100
O -0.27577600 -3.60708400 4.94049400
H 0.68414300 -3.68954700 4.84418100

6$_{cmplx}$

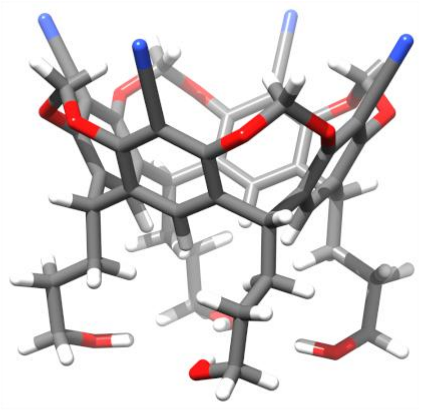

Energy = -2977.3643 Hartree

Number of imaginary frequencies 1

Symbol X Y Z

O -0.65522200 -4.68193200 -1.86075400

O 2.85267100 3.76082300 -1.87289100

O 4.68193200 -0.65522200 -1.86075400

O 3.76082300 -2.85267100 -1.87289100

O -3.76082300 2.85267100 -1.87289100

O 0.65522200 4.68193200 -1.86075400

O -4.68193200 0.65522200 -1.86075400

O -2.85267100 -3.76082300 -1.87289100

C 1.69065500 4.08049100 -2.63276300

H 1.31486500 3.19026900 -3.14696500

H 2.00918900 4.84795500 -3.33670600

C 4.08049100 -1.69065500 -2.63276300

H 4.84795500 -2.00918900 -3.33670600

H 3.19026900 -1.31486500 -3.14696500

C -4.08049100 1.69065500 -2.63276300

H -3.19026900 1.31486500 -3.14696500

H -4.84795500 2.00918900 -3.33670600

C -1.69065500 -4.08049100 -2.63276300

H -1.31486500 -3.19026900 -3.14696500

H -2.00918900 -4.84795500 -3.33670600

C 2.27690600 -2.28195200 -0.04259600

C -2.44981300 -1.01225900 0.53245900

H -1.95081200 -0.80884100 1.47109100

C 0.00124500 3.23035700 -0.03385500

C 3.34165700 -1.39050500 0.59717000

H 4.31462200 -1.79665300 0.30170700

C -0.27071600 3.86173600 -1.25245300

C 2.28195200 2.27690600 -0.04259600

C 0.27071600 -3.86173600 -1.25245300

C 1.01225900 -2.44981300 0.53245900

H 0.80884100 -1.95081200 1.47109100

C 2.92263200 2.52798100 -1.26093100

C 3.86173600 0.27071600 -1.25245300

C -3.86173600 -0.27071600 -1.25245300

C -1.01225900 2.44981300 0.53245900

H -0.80884100 1.95081200 1.47109100

C 2.52798100 -2.92263200 -1.26093100

C -2.28195200 -2.27690600 -0.04259600

C -2.27690600 2.28195200 -0.04259600

C 2.44981300 1.01225900 0.53245900

H 1.95081200 0.80884100 1.47109100

C -1.53474800 3.72285900 -1.86335800

C -1.39050500 -3.34165700 0.59717000

H -1.79665300 -4.31462200 0.30170700

C -3.23035700 0.00124500 -0.03385500

C -2.92263200 -2.52798100 -1.26093100

C -3.72285900 -1.53474800 -1.86335800

C 3.23035700 -0.00124500 -0.03385500

C 1.39050500 3.34165700 0.59717000

H 1.79665300 4.31462200 0.30170700

C 1.53474800 -3.72285900 -1.86335800

C -0.00124500 -3.23035700 -0.03385500

C -3.34165700 1.39050500 0.59717000

H -4.31462200 1.79665300 0.30170700

C -2.52798100 2.92263200 -1.26093100

C 3.72285900 1.53474800 -1.86335800

C -1.39866400 -3.27597500 2.13875900

H -2.44521600 -3.23121500 2.46324600

H -0.93915300 -2.34585400 2.48755700

C 3.27597500 -1.39866400 2.13875900

H 3.23121500 -2.44521600 2.46324600

H 2.34585400 -0.93915300 2.48755700

C -3.27597500 1.39866400 2.13875900

H -3.23121500 2.44521600 2.46324600

H -2.34585400 0.93915300 2.48755700

C 1.39866400 3.27597500 2.13875900

H 2.44521600 3.23121500 2.46324600

H 0.93915300 2.34585400 2.48755700
C 0.72008600 4.46683900 2.82741400
H -0.27807400 4.64599700 2.40852600
H 1.30941500 5.37695800 2.64743400
C -0.72008600 -4.46683900 2.82741400
H 0.27807400 -4.64599700 2.40852600
H -1.30941500 -5.37695800 2.64743400
C 4.46683900 -0.72008600 2.82741400
H 4.64599700 0.27807400 2.40852600
H 5.37695800 -1.30941500 2.64743400
C -4.46683900 0.72008600 2.82741400
H -4.64599700 -0.27807400 2.40852600
H -5.37695800 1.30941500 2.64743400
C 0.57374400 4.27670600 4.34153600
H 1.53906100 3.98786300 4.78389500
H 0.27583200 5.22739700 4.79718400
C -0.57374400 -4.27670600 4.34153600
H -1.53906100 -3.98786300 4.78389500
H -0.27583200 -5.22739700 4.79718400
C 4.27670600 -0.57374400 4.34153600
H 3.98786300 -1.53906100 4.78389500
H 5.22739700 -0.27583200 4.79718400
C -4.27670600 0.57374400 4.34153600
H -3.98786300 1.53906100 4.78389500
H -5.22739700 0.27583200 4.79718400
O -0.44502500 3.34165700 4.70841000
H -0.14008800 2.42310300 4.58837000
O 0.44502500 -3.34165700 4.70841000
H 0.14008800 -2.42310300 4.58837000
O 3.34165700 0.44502500 4.70841000
H 2.42310300 0.14008800 4.58837000
O -3.34165700 -0.44502500 4.70841000
H -2.42310300 -0.14008800 4.58837000
C 4.38632600 1.80614400 -3.09963400
N 4.92086800 2.02442400 -4.11056500
C -1.80614400 4.38632600 -3.09963400
N -2.02442400 4.92086800 -4.11056500
C 1.80614400 -4.38632600 -3.09963400
N 2.02442400 -4.92086800 -4.11056500
C -4.38632600 -1.80614400 -3.09963400
N -4.92086800 -2.02442400 -4.11056500

6$_{\mathbf{cryst}}$

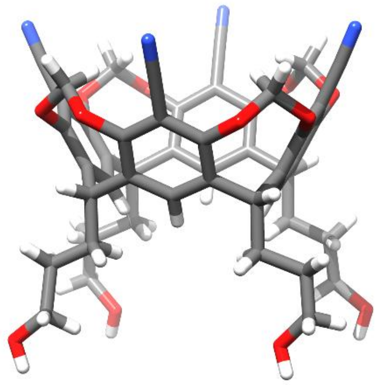

Energy = -2976.7545 Hartree

Number of imaginary frequencies 61

Symbol	X	Y	Z
C	-3.68852100	1.03704100	-1.22662500
C	-3.11987500	2.85269500	-2.66933700
H	-3.56251600	3.26792900	-3.45130900
H	-2.47272000	2.18445700	-3.00638700
C	-1.25651700	3.49471200	-1.29312700
C	-0.04361500	3.86588100	-1.87299600
C	1.16209300	3.62867500	-1.23595000
C	1.19456600	2.98481200	-0.01203600
C	-0.02479100	2.58428200	0.54182800
H	-0.01139000	2.11473900	1.36776500
C	-1.26859600	2.84089300	-0.06021800
C	2.52341200	2.67326200	0.64500800
H	3.16482300	3.38553400	0.35914800
C	3.00853300	1.36901200	0.03786000
C	3.69040200	1.38232300	-1.16909400
C	4.03880000	0.18448900	-1.80623600
C	3.00705300	-1.08206200	-0.02047900
C	2.66345900	0.11438300	0.57266400
H	2.16934400	0.09131700	1.38362800
C	3.13046200	3.22414700	-2.54687700
H	2.55023300	2.54314200	-2.97100900
H	3.59435700	3.71629700	-3.26977800
C	-0.06588900	4.51616200	-3.17490800
C	4.68444400	0.23438100	-3.08718700
C	2.58873200	-2.43099600	0.56030800
H	3.27036500	-3.09885100	0.26226100
O	-4.09360400	2.19740800	-1.87949200
O	-2.41492500	3.85875800	-1.94815200
O	2.32161300	4.12784200	-1.82480500
O	4.09053100	2.57200300	-1.75290400
C	2.52024000	2.66248800	2.17700600
H	2.14717600	3.51762500	2.50809900
H	1.93368300	1.93113600	2.49586700
C	3.91983800	2.46888600	2.74907100
H	4.21978600	1.54855400	2.54096400
H	4.53584900	3.09380200	2.28919700
C	4.02380100	2.65819100	4.04710400
H	4.16201700	3.63128400	4.17224700
H	4.87077200	2.22230900	4.32109900
O	3.06576500	2.27533600	4.98898800
H	3.30266500	2.53854000	5.75204000
C	2.55983100	-2.48010800	2.07929900
H	2.02300200	-1.71898300	2.41536900
H	2.11371900	-3.31582400	2.36615500
C	3.86638600	-2.42066600	2.66139300
H	4.17628000	-1.48393600	2.64654300
H	4.48328800	-2.95301600	2.10036000
C	3.94178100	-2.93124100	4.08032800
H	3.17783000	-2.53211700	4.55733300
H	3.76400100	-3.90429600	4.02918200
O	4.99342500	-2.80291500	4.90141400
H	4.73334000	-2.86279300	5.69838900
N	-0.10091200	5.02951500	-4.19383900
N	5.15499900	0.32681900	-4.12514400
C	3.68865700	-1.03743100	-1.22642800
C	3.12048000	-2.85172600	-2.66971500
H	3.56322300	-3.26674500	-3.45174300
H	2.47336800	-2.18339600	-3.00666500
C	1.25694200	-3.49412400	-1.29392600
C	0.04315500	-3.86485300	-1.87405600
C	-1.16195500	-3.62906200	-1.23710300
C	-1.19431000	-2.98457700	-0.01301600
C	0.02469500	-2.58516000	0.54111900
H	0.01146600	-2.11488400	1.36718300
C	1.26885900	-2.84064400	-0.06083500
C	-2.52324200	-2.67320800	0.64394000
H	-3.16461500	-3.38540000	0.35780000

C -3.00828300 -1.36879100 0.03708800
C -3.68999400 -1.38176900 -1.16995900
C -4.03926900 -0.18347900 -1.80681600
C -3.00679600 1.08229900 -0.02057400
C -2.66328000 -0.11430900 0.57228300
H -2.16955000 -0.09242700 1.38331900
C -3.12987400 -3.22321200 -2.54817600
H -2.54958900 -2.54209000 -2.97204400
H -3.59367400 -3.71516300 -3.27127400
C 0.06628000 -4.51601500 -3.17614500
C -4.68378500 -0.23329700 -3.08786500
C -2.58951100 2.43135300 0.56063900
H -3.27014400 3.09901100 0.26268700
O 4.09410500 -2.19665800 -1.87956100
O 2.41543500 -3.85798900 -1.94890000
O -2.32139900 -4.12806700 -1.82624800
O -4.09004600 -2.57128800 -1.75414900
C -2.52027100 -2.66285700 2.17594100
H -2.14725000 -3.51808500 2.50684800
H -1.93375500 -1.93159200 2.49508100
C -3.92022200 -2.47037300 2.74787600
H -4.22110400 -1.54970400 2.53998300
H -4.53589400 -3.09420100 2.28775000
C -4.02435600 -2.66003500 4.04584300
H -4.16230800 -3.63220200 4.17070000
H -4.87108300 -2.22326800 4.31984800
O -3.06740300 -2.27716100 4.98795900
H -3.30412400 -2.53961500 5.75090700
C -2.56012800 2.47880700 2.07964800
H -2.02306300 1.71854900 2.41557800
H -2.11377500 3.31540400 2.36679200
C -3.86772000 2.41948300 2.66155400
H -4.17637200 1.48343800 2.64640500
H -4.48330900 2.95266900 2.10058700
C -3.94302100 2.93062700 4.08062000
H -3.17941200 2.53041100 4.55761400
H -3.76427400 3.90341700 4.02976500
O -4.99381200 2.80179500 4.90153300
H -4.73383200 2.86145300 5.69855800
N 0.10171600 -5.02812600 -4.19521300
N -5.15420400 -0.32544900 -4.12590900

6$_{cryst\text{-}opt}$

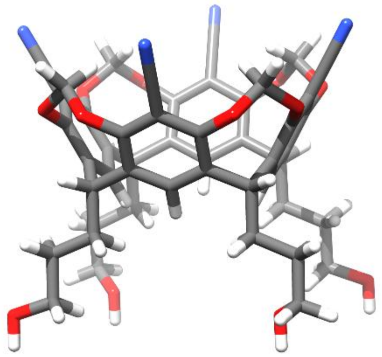

Energy = -2977.3755 Hartree

Number of imaginary frequencies 0

Symbol X Y Z

C -2.52247900 -2.95076500 -1.23750500

C -4.04583500 -1.72243000 -2.64006100

H -4.80530600 -2.04155100 -3.35198900

H -3.14379300 -1.36057800 -3.14349800

C -3.83372000 0.25076000 -1.27304500

C -3.69561500 1.51493000 -1.88036000

C -2.91060800 2.51137300 -1.26627400

C -2.27995800 2.26415900 -0.04072700

C -2.44831200 0.99849500 0.53529100

H -1.95912300 0.79665600 1.48160400

C -3.21611300 -0.01871200 -0.04539900

C -1.40728800 3.34727800 0.59467900

H -1.81748700 4.30604500 0.26242800

C -0.00462800 3.24486600 -0.00907300

C 0.26447600 3.87569800 -1.22988600

C 1.52733900 3.74489300 -1.84070300

C 2.27995800 2.31224800 -0.01505200

C 1.01654700 2.47793800 0.56648900

H 0.81551700 1.98546500 1.51156300

C -1.68582100 4.07925400 -2.62613900

H -1.29630600 3.19433000 -3.13916100

H -2.00649200 4.84544400 -3.33015400

C -4.34823400 1.78506600 -3.12300200

C 1.79330800 4.40493800 -3.08028900

C 3.35948600 1.40587700 0.58031100

H 4.31829900 1.79902100 0.22928600

O -3.75021800 -2.87604800 -1.85784600

O -4.64922600 -0.67260400 -1.88911500

O -2.84898300 3.74573300 -1.87426200

O -0.66523700 4.68815800 -1.84088700

C -1.45924300 3.34618300 2.13513900

H -2.51683400 3.31474700 2.42604300

H -1.00024100 2.44358400 2.54941600

C -0.80885300 4.58561900 2.76371100

H 0.26078900 4.62447000 2.52477700

H -1.26116600 5.49238400 2.34046800

C -0.97746100 4.65202200 4.27499700

H -2.04584800 4.64531500 4.53778300

H -0.54018100 5.58573100 4.65422500

O -0.31897700 3.52082300 4.87260100

H -0.40715800 3.59120400 5.83333500

C 3.42922300 1.39101400 2.12094300

H 2.46744500 1.11060400 2.56627500

H 4.13998100 0.60617600 2.40710200

C 3.89812200 2.73105200 2.70390900

H 3.18793600 3.52857700 2.45537000

H 4.86173300 3.00835200 2.25747800

C 4.05898800 2.67358200 4.21629700

H 3.09977100 2.42408800 4.69260300

H 4.78635400 1.89510000 4.49020200

O 4.51602300 3.95781000 4.66895000

H 4.60971100 3.92775000 5.63144300

N -4.87351200 2.00203000 -4.13876100

N 2.00628500 4.93624400 -4.09375800

C 2.52247900 2.95076500 -1.23750500

C 4.04583500 1.72243000 -2.64006100

H 4.80530600 2.04155100 -3.35198900

H 3.14379300 1.36057800 -3.14349800

C 3.83372000 -0.25076000 -1.27304500

C 3.69561500 -1.51493000 -1.88036000

C 2.91060800 -2.51137300 -1.26627400

C 2.27995800 -2.26415900 -0.04072700

C 2.44831200 -0.99849500 0.53529100

H 1.95912300 -0.79665600 1.48160400

C 3.21611300 0.01871200 -0.04539900

C 1.40728800 -3.34727800 0.59467900

H 1.81748700 -4.30604500 0.26242800

C 0.00462800 -3.24486600 -0.00907300
C -0.26447600 -3.87569800 -1.22988600
C -1.52733900 -3.74489300 -1.84070300
C -2.27995800 -2.31224800 -0.01505200
C -1.01654700 -2.47793800 0.56648900
H -0.81551700 -1.98546500 1.51156300
C 1.68582100 -4.07925400 -2.62613900
H 1.29630600 -3.19433000 -3.13916100
H 2.00649200 -4.84544400 -3.33015400
C 4.34823400 -1.78506600 -3.12300200
C -1.79330800 -4.40493800 -3.08028900
C -3.35948600 -1.40587700 0.58031100
H -4.31829900 -1.79902100 0.22928600
O 3.75021800 2.87604800 -1.85784600
O 4.64922600 0.67260400 -1.88911500
O 2.84898300 -3.74573300 -1.87426200
O 0.66523700 -4.68815800 -1.84088700
C 1.45924300 -3.34618300 2.13513900
H 2.51683400 -3.31474700 2.42604300
H 1.00024100 -2.44358400 2.54941600
C 0.80885300 -4.58561900 2.76371100
H -0.26078900 -4.62447000 2.52477700
H 1.26116600 -5.49238400 2.34046800
C 0.97746100 -4.65202200 4.27499700
H 2.04584800 -4.64531500 4.53778300
H 0.54018100 -5.58573100 4.65422500
O 0.31897700 -3.52082300 4.87260100
H 0.40715800 -3.59120400 5.83333500
C -3.42922300 -1.39101400 2.12094300
H -2.46744500 -1.11060400 2.56627500
H -4.13998100 -0.60617600 2.40710200
C -3.89812200 -2.73105200 2.70390900
H -3.18793600 -3.52857700 2.45537000
H -4.86173300 -3.00835200 2.25747800
C -4.05898800 -2.67358200 4.21629700
H -3.09977100 -2.42408800 4.69260300
H -4.78635400 -1.89510000 4.49020200
O -4.51602300 -3.95781000 4.66895000
H -4.60971100 -3.92775000 5.63144300
N 4.87351200 -2.00203000 -4.13876100
N -2.00628500 -4.93624400 -4.0937580

4⊃Cl⁻

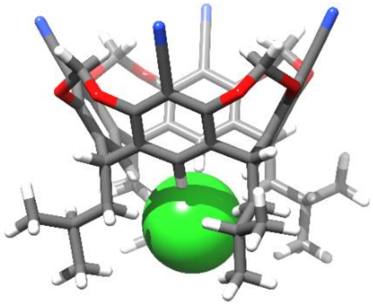

Energy = -3294.2453 Hartree

Number of imaginary frequencies 0

Symbol X Y Z

N -0.87832300 -5.14953800 -4.10722300

O 1.66932800 -4.40839300 -1.80888900

O -3.03898500 -3.61093700 -1.80643800

C 0.71100900 -3.13257200 0.01954500

C 0.58374500 -3.81084800 -1.19846700

C -0.67441100 -3.95287700 -1.81482000

C -1.81636400 -3.40641500 -1.19653800

C -1.71217800 -2.72342200 0.01890600

C -0.44229200 -2.58329900 0.59212300

H -0.34877300 -2.00648000 1.50822800

C -0.78862600 -4.62533800 -3.07172200

C -3.59228100 -2.54801300 -2.56849000

H -4.26211400 -3.02123200 -3.28470100

H -2.79492100 -1.98623500 -3.07038300

C -2.95594400 -2.09424100 0.63756500

H -3.80832100 -2.68979900 0.30013400

C -2.95904100 -2.07350800 2.17866300

H -2.16303100 -1.42792900 2.56139800

H -3.90705300 -1.61635100 2.49613400

N 5.15109300 -0.87874700 -4.10632300

O 4.40887300 1.66928900 -1.80863000

O 3.61140600 -3.03906600 -1.80602100

C 3.13248100 0.71106200 0.01943900

C 3.81137600 0.58368100 -1.19819900

C 3.95365300 -0.67453900 -1.81435500

C 3.40689400 -1.81641500 -1.19617800

C 2.72323300 -1.71209700 0.01887300

C 2.58255800 -0.44209400 0.59169100

H 2.00420400 -0.34814900 1.50680600

C 4.62653900 -0.78888800 -3.07101500

C 2.54842600 -3.59198500 -2.56830300

H 3.02155700 -4.26191000 -3.28448600

H 1.98699700 -2.79440100 -3.07023000

C 2.09406800 -2.95582000 0.63768600

H 2.68964400 -3.80821500 0.30033200

C 2.07361400 -2.95856400 2.17878200

H 1.42821600 -2.16237400 2.56144300

H 1.61646400 -3.90646600 2.49659600

N -5.15087700 0.87870300 -4.10660000

O -4.40890800 -1.66928400 -1.80885300

O -3.61129000 3.03903300 -1.80617700

C -3.13259900 -0.71117800 0.01934000

C -3.81137700 -0.58373300 -1.19836300

C -3.95356400 0.67448600 -1.81456600

C -3.40683700 1.81635300 -1.19637200

C -2.72332800 1.71198100 0.01875700

C -2.58275100 0.44199300 0.59163600

H -2.00463300 0.34824300 1.50692000

C -4.62635500 0.78882300 -3.07127900

C -2.54830700 3.59208300 -2.56836500

H -3.02146300 4.26195600 -3.28458000

H -1.98673700 2.79456900 -3.07024400

C -2.09414300 2.95567300 0.63754500

H -2.68972700 3.80809600 0.30027500

C -2.07346400 2.95843000 2.17864600

H -1.42808500 2.16217900 2.56121800

H -1.61613100 3.90627800 2.49635800

N 0.87858800 5.15027800 -4.10686900

O -1.66934200 4.40860900 -1.80889400

O 3.03902100 3.61106400 -1.80626200

C -0.71112600 3.13241700 0.01932500

C -0.58376000 3.81107200 -1.19845600

C 0.67444900 3.95321800 -1.81469500

C 1.81634800 3.40658600 -1.19644900
C 1.71202600 2.72323000 0.01877400
C 0.44206300 2.58271100 0.59169100
H 0.34827800 2.00483500 1.50711700
C 0.78875600 4.62590500 -3.07146700
C 3.59227200 2.54805200 -2.56826500
H 4.26212100 3.02121500 -3.28449800
H 2.79486900 1.98630500 -3.07012000
C 2.95571100 2.09410300 0.63760600
H 3.80813400 2.68967400 0.30031800
C 2.95849300 2.07347200 2.17870900
H 2.16219000 1.42818200 2.56131900
H 3.90630300 1.61605300 2.49641200
C -3.45249800 2.82159800 2.85070400
C -4.42608300 3.93911100 2.44723000
C -3.26978000 2.76670600 4.37513200
H -3.88970200 1.86418000 2.53586600
H -4.65355800 3.92180100 1.37603200
H -5.37568400 3.84240700 2.98653000
H -4.00442900 4.92468500 2.68613600
H -2.58999800 1.95596600 4.65790100
H -2.84546400 3.70846500 4.74816400
H -4.22913800 2.60880900 4.88236800
C 2.82183400 3.45254300 2.85072500
C 3.93943400 4.42599400 2.44717100
C 2.76698300 3.26988400 4.37516200
H 1.86445200 3.88984300 2.53591100
H 3.92210400 4.65342900 1.37596400
H 3.84285100 5.37562600 2.98643800
H 4.92497200 4.00424500 2.68605400
H 1.95618100 2.59019900 4.65798700
H 3.70871200 2.84547900 4.74817200
H 2.60920900 4.22927500 4.88237100
C -2.82211700 -3.45249000 2.85080200
C -3.93949500 -4.42620200 2.44726200
C -2.76740500 -3.26968800 4.37522800
H -1.86462800 -3.88962400 2.53607900
H -3.92206800 -4.65371300 1.37607300
H -3.84273400 -5.37577400 2.98660400
H -4.92513200 -4.00464400 2.68607000
H -1.95677100 -2.58980300 4.65805200
H -3.70925100 -2.84545500 4.74813500
H -2.60945700 -4.22900100 4.88253100
C 3.45273900 -2.82150100 2.85060400
C 4.42645500 -3.93885300 2.44700800
C 3.27025500 -2.76660900 4.37506200
H 3.88971200 -1.86400600 2.53566700
H 4.65381100 -3.92149900 1.37578500
H 5.37610000 -3.84199400 2.98620300
H 4.00499100 -4.92449800 2.68595500
H 2.59032900 -1.95602300 4.65792500
H 2.84621700 -3.70845700 4.74818800
H 4.22965700 -2.60847600 4.88213800
Cl 0.00004600 -0.00039800 3.21477500

4⊃Br⁻

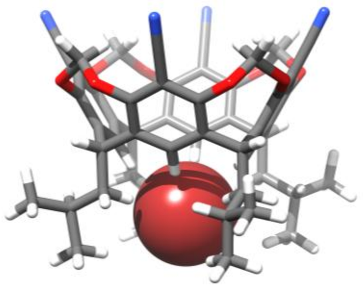

Energy = -5405.7978 Hartree

Number of imaginary frequencies 0

Symbol	X	Y	Z
Br	0.00000000	0.00000000	3.21063100
N	-4.82959500	1.99887200	-4.23758100
O	-4.66977900	-0.65202100	-1.94022800
O	-2.84952500	3.76379500	-1.93763900
C	-3.21297700	0.00000000	-0.11259500
C	-3.84749700	0.27483500	-1.32963700
C	-3.70894200	1.53411700	-1.94482100
C	-2.92177900	2.52645200	-1.32758400
C	-2.27732900	2.27284600	-0.11317000
C	-2.42072500	1.00281500	0.45821400
H	-1.87886400	0.78474400	1.37377100
C	-4.33905300	1.79466500	-3.20185200
C	-1.68989500	4.06737900	-2.69939600
H	-2.00288900	4.82487900	-3.41615700
H	-1.31846300	3.16510200	-3.20055700
C	-1.38782100	3.34574200	0.50643900
H	-1.78049400	4.30952200	0.17140000
C	-1.36158500	3.33930900	2.04715700
H	-0.92011900	2.41303000	2.42403500
H	-0.69184800	4.15036800	2.36645500
N	-1.99887200	-4.82959500	-4.23758100
O	0.65202100	-4.66977900	-1.94022800
O	-3.76379500	-2.84952500	-1.93763900
C	0.00000000	-3.21297700	-0.11259500
C	-0.27483500	-3.84749700	-1.32963700
C	-1.53411700	-3.70894200	-1.94482100
C	-2.52645200	-2.92177900	-1.32758400
C	-2.27284600	-2.27732900	-0.11317000
C	-1.00281500	-2.42072500	0.45821400
H	-0.78474400	-1.87886400	1.37377100
C	-1.79466500	-4.33905300	-3.20185200
C	-4.06737900	-1.68989500	-2.69939600
H	-4.82487900	-2.00288900	-3.41615700
H	-3.16510200	-1.31846300	-3.20055700
C	-3.34574200	-1.38782100	0.50643900
H	-4.30952200	-1.78049400	0.17140000
C	-3.33930900	-1.36158500	2.04715700
H	-2.41303000	-0.92011900	2.42403500
H	-4.15036800	-0.69184800	2.36645500
N	1.99887200	4.82959500	-4.23758100
O	-0.65202100	4.66977900	-1.94022800
O	3.76379500	2.84952500	-1.93763900
C	0.00000000	3.21297700	-0.11259500
C	0.27483500	3.84749700	-1.32963700
C	1.53411700	3.70894200	-1.94482100
C	2.52645200	2.92177900	-1.32758400
C	2.27284600	2.27732900	-0.11317000
C	1.00281500	2.42072500	0.45821400
H	0.78474400	1.87886400	1.37377100
C	1.79466500	4.33905300	-3.20185200
C	4.06737900	1.68989500	-2.69939600
H	4.82487900	2.00288900	-3.41615700
H	3.16510200	1.31846300	-3.20055700
C	3.34574200	1.38782100	0.50643900
H	4.30952200	1.78049400	0.17140000
C	3.33930900	1.36158500	2.04715700
H	2.41303000	0.92011900	2.42403500
H	4.15036800	0.69184800	2.36645500
N	4.82959500	-1.99887200	-4.23758100
O	4.66977900	0.65202100	-1.94022800
O	2.84952500	-3.76379500	-1.93763900
C	3.21297700	0.00000000	-0.11259500
C	3.84749700	-0.27483500	-1.32963700

C 3.70894200 -1.53411700 -1.94482100
C 2.92177900 -2.52645200 -1.32758400
C 2.27732900 -2.27284600 -0.11317000
C 2.42072500 -1.00281500 0.45821400
H 1.87886400 -0.78474400 1.37377100
C 4.33905300 -1.79466500 -3.20185200
C 1.68989500 -4.06737900 -2.69939600
H 2.00288900 -4.82487900 -3.41615700
H 1.31846300 -3.16510200 -3.20055700
C 1.38782100 -3.34574200 0.50643900
H 1.78049400 -4.30952200 0.17140000
C 1.36158500 -3.33930900 2.04715700
H 0.92011900 -2.41303000 2.42403500
H 0.69184800 -4.15036800 2.36645500
C 3.52191600 2.72925500 2.72995300
C 4.84999000 3.40675300 2.36110000
C 3.38718800 2.55482500 4.25072400
H 2.70520600 3.38526400 2.39815600
H 4.91627600 3.62981600 1.29078300
H 4.96926400 4.35277500 2.90221100
H 5.69901900 2.76158700 2.62390900
H 2.42596700 2.09729300 4.50779200
H 4.18321200 1.90407900 4.63673700
H 3.46328400 3.51918800 4.76725300
C 2.72925500 -3.52191600 2.72995300
C 3.40675300 -4.84999000 2.36110000
C 2.55482500 -3.38718800 4.25072400
H 3.38526400 -2.70520600 2.39815600
H 3.62981600 -4.91627600 1.29078300
H 4.35277500 -4.96926400 2.90221100
H 2.76158700 -5.69901900 2.62390900
H 2.09729300 -2.42596700 4.50779200
H 1.90407900 -4.18321200 4.63673700
H 3.51918800 -3.46328400 4.76725300
C -2.72925500 3.52191600 2.72995300
C -3.40675300 4.84999000 2.36110000
C -2.55482500 3.38718800 4.25072400
H -3.38526400 2.70520600 2.39815600
H -3.62981600 4.91627600 1.29078300
H -4.35277500 4.96926400 2.90221100
H -2.76158700 5.69901900 2.62390900
H -2.09729300 2.42596700 4.50779200
H -1.90407900 4.18321200 4.63673700
H -3.51918800 3.46328400 4.76725300
C -3.52191600 -2.72925500 2.72995300
C -4.84999000 -3.40675300 2.36110000
C -3.38718800 -2.55482500 4.25072400
H -2.70520600 -3.38526400 2.39815600
H -4.91627600 -3.62981600 1.29078300
H -4.96926400 -4.35277500 2.90221100
H -5.69901900 -2.76158700 2.62390900
H -2.42596700 -2.09729300 4.50779200
H -4.18321200 -1.90407900 4.63673700
H -3.46328400 -3.51918800 4.76725300

5$_2$⊃Cl^-

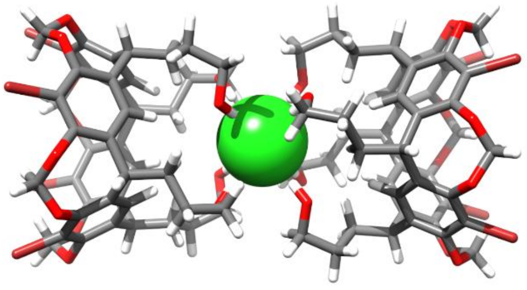

Energy = -26246.489 Hartree

Number of imaginary frequencies 0

Symbol	X	Y	Z
C	-5.79297100	0.14116700	3.21185800
C	-5.15478400	-1.23700100	3.39237600
C	-5.20994800	1.11877600	2.40206100
C	-3.61987900	-1.17615000	3.30383300
C	-5.79163600	-2.17840800	2.36792100
H	-5.43941600	-1.60059600	4.38572200
C	-7.02598700	0.43225500	3.81323400
C	-5.79150400	2.36853100	2.17846800
H	-4.24951900	0.91358200	1.95002000
C	-2.89668400	-2.51072400	3.52729300
H	-3.31633400	-0.79684100	2.32610600
H	-3.25927600	-0.42875900	4.01790400
C	-5.20988700	-2.40214700	1.11828000
C	-7.02347500	-2.78812000	2.64230400
C	-7.64193400	1.66747500	3.57961800
O	-7.61860300	-0.45470700	4.68858800
C	-5.15437300	3.39307600	1.23739300
C	-7.02347700	2.64296000	2.78788400
C	-1.52121200	-2.50512900	2.85856300
H	-3.47101400	-3.34084200	3.09926300
H	-2.78525900	-2.71967100	4.59957000
C	-5.79256300	-3.21226800	0.14074400
H	-4.24958900	-1.94985100	0.91304400
C	-7.64164500	-3.58013100	1.66685300
O	-7.61445700	-2.67251700	3.88392000
C	-8.38569200	-1.51352900	4.14455800
C	-3.61943300	3.30454100	1.17689200
C	-5.79227800	3.21258300	-0.14091200
H	-5.43909800	4.38637600	1.60102300
O	-7.61429600	3.88465800	2.67228800
O	-1.69364400	-2.47173200	1.43415200
H	-0.95898500	-3.40984900	3.12422900
H	-0.93385900	-1.64063400	3.19345800
C	-5.15415000	-3.39282800	-1.23729700
C	-7.02551600	-3.81380400	0.43172600
H	-9.09200400	-1.79717500	4.92432800
H	-8.90183700	-1.18427500	3.23473100
C	-2.89656300	3.52613100	2.51205300
H	-3.31583000	2.32734700	0.79631500
H	-3.25870400	4.01968000	0.43057400
C	-5.20932800	2.40242000	-1.11827000
C	-7.02513400	3.81415200	-0.43227000
C	-8.38522400	4.14556500	1.51316400
C	-3.61919800	-3.30456400	-1.17604300
C	-5.79090400	-2.36826300	-2.17865800
H	-5.43883100	-4.38613100	-1.60095800
O	-7.61794400	-4.68924300	-0.45527500
C	-1.52169600	2.85617900	2.50633900
H	-3.47167000	3.09798400	3.34157300
H	-2.78413900	4.59810000	2.72197700
C	-5.79075300	2.17868200	-2.36807600
H	-4.24906900	1.95014700	-0.91278700
C	-7.64091600	3.58048100	-1.66756800
O	-7.61782700	4.68960100	0.45455200
H	-9.09146800	4.92542000	1.79674700
H	-8.90145400	3.23586800	1.18367600
C	-2.89550800	-3.52775800	-2.51049900
H	-3.31556900	-2.32705200	-0.79626900
H	-3.25907400	-4.01904400	-0.42880000
C	-5.20930900	-1.11848300	-2.40205500
C	-7.02269200	-2.64267100	-2.78846900
C	-8.38493900	-4.14517800	-1.51417000
O	-1.69546800	1.43189800	2.47126800

H -0.95946000 3.11997100 3.41161900
H -0.93363800 3.19145800 1.64248600
C -5.15373500 1.23725600 -3.39241400
C -7.02250000 2.78842000 -2.64281400
C -1.52033000 -2.85845000 -2.50435100
H -3.46980700 -3.10010900 -3.34082700
H -2.78344400 -4.59997600 -2.71933500
C -5.79205300 -0.14088100 -3.21206300
H -4.24904700 -0.91325200 -1.94968500
C -7.64089400 -1.66717800 -3.58039900
O -7.61359300 -3.88434300 -2.67302900
H -9.09114300 -4.92498700 -1.79797700
H -8.90120800 -3.23543300 -1.18487700
H -1.18691400 1.05864000 1.73573700
C -3.61888200 1.17605900 -3.30352600
H -5.43812900 1.60093000 -4.38579800
O -7.61313300 2.67282400 -3.88460200
O -1.69350000 -1.43407700 -2.47061100
H -0.95747000 -3.12327700 -3.40893800
H -0.93311800 -3.19324900 -1.63972600
C -7.02487300 -0.43195700 -3.81383300
C -2.89520100 2.51085100 -3.52437700
H -3.31577400 0.79504400 -2.32635500
H -3.25823400 0.42977600 -4.01873200
C -8.38437000 1.51390000 -4.14549500
H -1.18553200 -1.06052200 -1.73481500
O -7.61720700 0.45503100 -4.68934800
C -1.52002100 2.50351600 -2.85502800
H -3.46949600 3.34046500 -3.09530600
H -2.78310300 2.72153000 -4.59623300
H -9.09043200 1.79761700 -4.92546500
H -8.90081400 1.18464500 -3.23583700
O -1.69306600 2.46763800 -1.43074700
H -0.95719600 3.40849100 -3.11853400
H -0.93280900 1.63945700 -3.19126400
O 7.61314000 0.87363000 4.63337500
O 7.61732900 1.48086400 -4.47292500
O 7.61299500 4.63336700 -0.87338700
O 7.61666500 4.47273400 1.48034700
O 7.61858300 -4.47198200 -1.48036700
O 7.61418600 -0.87288200 -4.63342400
O 7.61465300 -4.63267200 0.87336300
O 7.61729900 -1.48012200 4.47287900
C 8.38489300 0.29283000 -4.40305800
H 8.90058100 0.22631300 -3.43750500
H 9.09153800 0.34876200 -5.23063900
C 8.38398300 4.40301400 0.29214600
H 9.09056300 5.23065900 0.34796500
H 8.89974200 3.43751300 0.22546300
C 8.38571300 -4.40200600 -0.29206400
H 8.90110600 -3.43631600 -0.22527900
H 9.09260700 -5.22938900 -0.34780200
C 8.38436900 -0.29176400 4.40312500
H 8.90016200 -0.22503500 3.43764400
H 9.09092300 -0.34739200 5.23080400
C 5.79142700 2.88088200 1.42814000
C 5.20943900 -1.99466900 1.74482700
H 4.24918500 -1.62374300 1.41437700
C 5.79116300 -1.03466600 -3.04736700
C 5.15312000 3.60294400 0.24045000
H 5.43734900 4.65858600 0.30960200
C 7.02323200 -1.48079100 -3.54470000
C 5.79174400 1.42834800 -2.88143400
C 7.02212200 1.48130800 3.54456000
C 5.20877600 1.74462600 1.99442100
H 4.24864600 1.41403800 1.62322900
C 7.02471200 1.93791900 -3.31362700
C 7.02207700 3.54463700 -1.48132500
C 7.02324500 -3.54415800 1.48120300
C 5.20971500 -1.74484400 -1.99489500
H 4.24937600 -1.41466000 -1.62390200
C 7.02436300 3.31334200 1.93755400
C 5.79192700 -1.42831000 2.88112700
C 5.79274400 -2.88083000 -1.42846900

C 5.20885400 1.99447600 -1.74521400
H 4.24867200 1.62322000 -1.41489100
C 7.64183000 -2.59937400 -2.97274800
C 5.15337000 -0.24070600 3.60315800
H 5.43750900 -0.30973400 4.65882500
C 5.79127600 -3.04695300 1.03467000
C 7.02502400 -1.93741200 3.31349900
C 7.64131500 -2.97230200 2.60013200
C 5.79022000 3.04697600 -1.03498300
C 5.15378200 0.24048500 -3.60357500
H 5.43799700 0.30963800 -4.65921100
C 7.64037100 2.60013600 2.97271700
C 5.79031800 1.03469300 3.04701200
C 5.15449800 -3.60309000 -0.24087000
H 5.43895600 -4.65867400 -0.30999500
C 7.02593500 -3.31280500 -1.93767100
C 7.64052000 2.97303100 -2.60016700
C 3.61847200 -0.26054700 3.49697800
H 3.25788700 -1.23340700 3.84639200
H 3.31535700 -0.21086600 2.44930000
C 3.61821700 3.49698500 0.26019800
H 3.25761500 3.84694100 1.23285800
H 3.31492400 2.44933300 0.21098000
C 3.61956600 -3.49746500 -0.26069400
H 3.25899300 -3.84694100 -1.23353500
H 3.31606200 -2.44989300 -0.21092100
C 3.61888500 0.25966500 -3.49748100
H 3.25780800 1.23199900 -3.84785400
H 3.31578900 0.21085200 -2.44976300
C 2.89618400 -0.87078200 -4.24182000
H 3.47193200 -1.80232200 -4.18792700
H 2.78292200 -0.62721100 -5.30656500
C 2.89517900 0.86878300 4.24242600
H 3.47037300 1.80070700 4.18933800
H 2.78215000 0.62417400 5.30696000
C 2.89530800 4.24217900 -0.86955200
H 3.47082600 4.18878000 -1.80127000
H 2.78244700 5.30679800 -0.62524200
C 2.89681800 -4.24334600 0.86864900
H 3.47249900 -4.19058900 1.80030400
H 2.78384600 -5.30779900 0.62363600
C 1.52195200 -1.13841400 -3.62629400
H 0.93356600 -0.21305400 -3.58162300
H 0.95934300 -1.85879300 -4.23433400
C 1.52075900 1.13623400 3.62723600
H 0.93271000 0.21067600 3.58213400
H 0.95799200 1.85608400 4.23576000
C 1.52072500 3.62743900 -1.13745700
H 0.93228200 3.58222800 -0.21218500
H 0.95861100 4.23661500 -1.85726900
C 1.52241200 -3.62856700 1.13732300
H 0.93380300 -3.58242000 0.21220300
H 0.96037300 -4.23828600 1.85670900
O 1.69679100 -1.68444300 -2.31060200
H 1.18977400 -1.16275800 -1.67040900
O 1.69515800 1.68302700 2.31180800
H 1.18865200 1.16110000 1.67139600
O 1.69475300 2.31223800 -1.68484300
H 1.18823500 1.67171900 -1.16311700
O 1.69692200 -2.31385800 1.68569800
H 1.19135700 -1.67268500 1.16384300
Cl 0.00614800 -0.00180100 0.00109200
H -1.18522300 1.73117600 -1.05820500
H -1.18606900 -1.73559200 1.06065100
Br -9.37319300 -4.29425700 1.99941500
Br -9.37360300 2.00012500 4.29340500
Br -9.37238000 4.29458900 -2.00062000
Br -9.37235300 -1.99983300 -4.29470400
Br 9.37180700 3.56689200 -3.11881000
Br 9.37332000 -3.11779200 -3.56619800
Br 9.37157200 3.11922100 3.56644900
Br 9.37276100 -3.56548900 3.11902700

6⊃Cl⁻

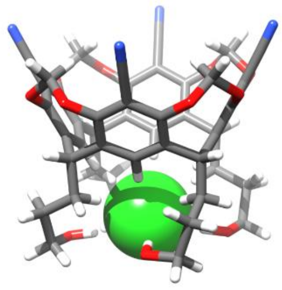

Energy = -3437.8707 Hartree

Number of imaginary frequencies 0

Symbol	X	Y	Z
O	1.18551300	-4.56661300	-1.94396800
O	1.18873000	4.56353400	-1.94853600
O	4.56661300	1.18551300	-1.94396800
O	4.56353400	-1.18873000	-1.94853600
O	-4.56353400	1.18873000	-1.94853600
O	-1.18551300	4.56661300	-1.94396800
O	-4.56661300	-1.18551300	-1.94396800
O	-1.18873000	-4.56353400	-1.94853600
C	0.00000000	4.40895900	-2.70888600
H	-0.00226300	3.43678700	-3.21699300
H	-0.00007700	5.23348500	-3.41991800
C	4.40895900	0.00000000	-2.70888600
H	5.23348500	0.00007700	-3.41991800
H	3.43678700	0.00226300	-3.21699300
C	-4.40895900	0.00000000	-2.70888600
H	-3.43678700	-0.00226300	-3.21699300
H	-5.23348500	-0.00007700	-3.41991800
C	0.00000000	-4.40895900	-2.70888600
H	0.00226300	-3.43678700	-3.21699300
H	0.00007700	-5.23348500	-3.41991800
C	2.97018900	-1.23175200	-0.12171800
C	-1.85992400	-1.85923900	0.45040600
H	-1.46800700	-1.46823100	1.38120200
C	-1.23039200	2.97154600	-0.11978900
C	3.61229300	-0.00267500	0.51436400
H	4.66791600	-0.00145300	0.22137300
C	-1.72416200	3.44989400	-1.33725600
C	1.23175200	2.97018900	-0.12171800
C	1.72416200	-3.44989400	-1.33725600
C	1.85923900	-1.85992400	0.45040600
H	1.46823100	-1.46800700	1.38120200
C	1.72602900	3.44712400	-1.34025500
C	3.44989400	1.72416200	-1.33725600
C	-3.44989400	-1.72416200	-1.33725600
C	-1.85923900	1.85992400	0.45040600
H	-1.46823100	1.46800700	1.38120200
C	3.44712400	-1.72602900	-1.34025500
C	-1.23175200	-2.97018900	-0.12171800
C	-2.97018900	1.23175200	-0.12171800
C	1.85992400	1.85923900	0.45040600
H	1.46800700	1.46823100	1.38120200
C	-2.83116100	2.83407500	-1.95505800
C	-0.00267500	-3.61229300	0.51436400
H	-0.00145300	-4.66791600	0.22137300
C	-2.97154600	-1.23039200	-0.11978900
C	-1.72602900	-3.44712400	-1.34025500
C	-2.83407500	-2.83116100	-1.95505800
C	2.97154600	1.23039200	-0.11978900
C	0.00267500	3.61229300	0.51436400
H	0.00145300	4.66791600	0.22137300
C	2.83116100	-2.83407500	-1.95505800
C	1.23039200	-2.97154600	-0.11978900
C	-3.61229300	0.00267500	0.51436400
H	-4.66791600	0.00145300	0.22137300
C	-3.44712400	1.72602900	-1.34025500
C	2.83407500	2.83116100	-1.95505800
C	-0.01796600	-3.52609300	2.05151600
H	-0.98002200	-3.90454500	2.41387200
H	0.01044700	-2.48096200	2.36540300
C	3.52609300	-0.01796600	2.05151600
H	3.90454500	-0.98002200	2.41387200
H	2.48096200	0.01044700	2.36540300
C	-3.52609300	0.01796600	2.05151600

H -3.90454500 0.98002200 2.41387200
H -2.48096200 -0.01044700 2.36540300
C 0.01796600 3.52609300 2.05151600
H 0.98002200 3.90454500 2.41387200
H -0.01044700 2.48096200 2.36540300
C -1.13177000 4.27955500 2.73387400
H -2.06601600 4.14899700 2.17433500
H -0.91631700 5.35739600 2.75168400
C 1.13177000 -4.27955500 2.73387400
H 2.06601600 -4.14899700 2.17433500
H 0.91631700 -5.35739600 2.75168400
C 4.27955500 1.13177000 2.73387400
H 4.14899700 2.06601600 2.17433500
H 5.35739600 0.91631700 2.75168400
C -4.27955500 -1.13177000 2.73387400
H -4.14899700 -2.06601600 2.17433500
H -5.35739600 -0.91631700 2.75168400
C -1.38231800 3.78062900 4.16314600
H -0.44006800 3.76819800 4.73190700
H -2.07227700 4.46084000 4.67580000
C 1.38231800 -3.78062900 4.16314600
H 0.44006800 -3.76819800 4.73190700
H 2.07227700 -4.46084000 4.67580000
C 3.78062900 1.38231800 4.16314600
H 3.76819800 0.44006800 4.73190700
H 4.46084000 2.07227700 4.67580000
C -3.78062900 -1.38231800 4.16314600
H -3.76819800 -0.44006800 4.73190700
H -4.46084000 -2.07227700 4.67580000
O -2.00669500 2.49825200 4.18802100
H -1.34230600 1.79621200 4.06227800
O 2.00669500 -2.49825200 4.18802100
H 1.34230600 -1.79621200 4.06227800
O 2.49825200 2.00669500 4.18802100
H 1.79621200 1.34230600 4.06227800
O -2.49825200 -2.00669500 4.18802100
H -1.79621200 -1.34230600 4.06227800
C 3.31600100 3.31102000 -3.21323300
N 3.69094300 3.68369500 -4.25022200
C -3.31102000 3.31600100 -3.21323300
N -3.68369500 3.69094300 -4.25022200
C 3.31102000 -3.31600100 -3.21323300
N 3.68369500 -3.69094300 -4.25022200
C -3.31600100 -3.31102000 -3.21323300
N -3.69094300 -3.68369500 -4.25022200
Cl 0.00000000 0.00000000 3.42207200

6⊃(Cl⁻)$_2$

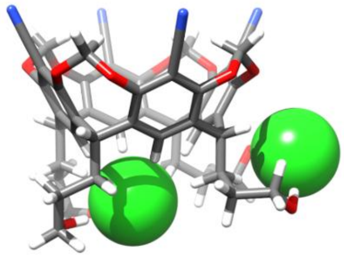

Energy = -3898.1249 Hartree

Number of imaginary frequencies 0

Symbol X Y Z

O 2.40198400 -3.49043300 -2.29800800
O -1.41376800 4.82413300 -1.41976800
O 3.02872900 3.18065700 -2.03432200
O 3.98045300 1.01425000 -2.28779200
O -5.15635000 -0.70529300 -1.44432000
O -3.54788300 3.78939400 -1.30590600
O -4.18313100 -2.86646300 -1.61453400
O 0.26645100 -4.51871700 -2.13937100
C -2.47282700 4.21549000 -2.13497400
H -2.09796800 3.37273900 -2.72882000
H -2.89096500 4.99731100 -2.76787700
C 3.26737400 2.06108100 -2.89427500
H 3.91541400 2.44839700 -3.67956400
H 2.31226800 1.70129600 -3.29917900
C -4.57511800 -1.68747300 -2.29369300
H -3.72366800 -1.25931300 -2.83700100
H -5.37322400 -1.98817200 -2.97146000
C 1.20812500 -3.82347500 -2.96135200
H 0.73590900 -2.92851100 -3.38656700
H 1.48200400 -4.53798900 -3.73680600
C 2.76424400 0.20453300 -0.34826900
C -1.21709900 -2.46829500 0.53122300
H -0.92703700 -1.98458900 1.45798000
C -2.72664000 2.21574800 0.34995000
C 2.94363500 1.54379800 0.35051600
H 3.86592900 1.96553500 -0.05635300
C -3.49605400 2.51325900 -0.77914200
C -0.50692700 3.28345400 0.22001000
C 2.46388300 -2.26959200 -1.64347600
C 2.07524200 -0.85453100 0.24723100
H 1.65159200 -0.70487900 1.23418600
C -0.38840000 4.00859800 -0.96778200
C 1.88172700 3.17549100 -1.27478400
C -2.88805400 -2.92885700 -1.12585600
C -2.76506900 0.90654000 0.84211000
H -2.14501300 0.65771100 1.69586200
C 3.27180000 0.02185500 -1.63659800
C -0.24284600 -3.18340200 -0.17610300
C -3.56222800 -0.09906800 0.28348900
C 0.58760100 2.51076800 0.63096900
H 0.49463300 1.91235500 1.53120900
C -4.29319600 1.52120200 -1.38644800
C 1.20499500 -3.26375600 0.30400500
H 1.63127900 -4.18425900 -0.10642100
C -2.54062700 -2.33727600 0.09037300
C -0.61613400 -3.76960900 -1.39266600
C -1.93643600 -3.64948300 -1.87330700
C 1.79185800 2.44784600 -0.08082900
C -1.83266300 3.28495500 0.97616600
H -2.31270900 4.25086900 0.78325000
C 3.12308300 -1.21179500 -2.29581200
C 1.94693100 -2.10761700 -0.35763800
C -3.57770200 -1.51495200 0.85214300
H -4.55872500 -1.94320900 0.61781800
C -4.31899400 0.21815700 -0.84887800
C 0.80013000 3.96312300 -1.72151300
C 1.31537400 -3.33849100 1.84270700
H 0.53188700 -4.02465900 2.18676500
H 1.07398300 -2.37738100 2.30717800
C 3.11727200 1.41700500 1.87710000
H 3.83482000 0.61015200 2.05071500
H 2.18428500 1.11645200 2.36308500
C -3.38105600 -1.53120200 2.37718000
H -4.09035200 -0.82877600 2.82957600

H -2.39491600 -1.13721200 2.62620600
C -1.65269600 3.12285300 2.49641000
H -0.93938100 3.88454500 2.83494500
H -1.18212300 2.16304700 2.72008900
C -2.95502800 3.24274000 3.30152900
H -3.78056000 2.74924200 2.77451300
H -3.23319600 4.30162100 3.41180000
C 2.66060100 -3.83961100 2.39835200
H 2.91162800 -4.81439200 1.95551400
H 2.51253100 -4.00701900 3.47387400
C 3.64794100 2.70661800 2.52130000
H 3.52474000 2.63567100 3.60906500
H 3.05293500 3.57111900 2.18635200
C -3.53403500 -2.91206500 3.02578000
H -3.05408900 -3.68445000 2.41232400
H -4.59910200 -3.17771800 3.10548900
C -2.84850500 2.58896200 4.68733500
H -1.93596700 2.93817500 5.19846300
H -3.70546800 2.89268600 5.30226800
C 3.87245300 -2.92022100 2.23179400
H 3.59394600 -1.88166100 2.46521200
H 4.64711600 -3.21227200 2.96105500
C 5.13422300 2.98125000 2.23904400
H 5.31866400 3.02283500 1.15423200
H 5.39622400 3.96733300 2.64897400
C -2.88198500 -2.95914600 4.41611700
H -3.21794900 -2.09740200 5.01505900
H -3.19655100 -3.87118900 4.93933200
O -2.88839500 1.17062300 4.63187500
H -2.02181000 0.81149400 4.33916800
O 4.37817400 -3.02460100 0.91082100
H 4.96249900 -2.24637500 0.75527400
O 5.99459000 2.03834400 2.85376100
H 6.07545000 1.25126200 2.26494900
O -1.46315300 -3.01494100 4.35477800
H -1.09032000 -2.13057300 4.15919100
C 0.89348200 4.69176000 -2.94861200
N 0.94631500 5.27348900 -3.95618300
C -5.06284900 1.83304500 -2.55066600
N -5.67578300 2.08284600 -3.50885300
C 3.60236200 -1.38062300 -3.63216100
N 3.94452200 -1.51077600 -4.73800200
C -2.30600500 -4.23622500 -3.12398600
N -2.61365900 -4.69298500 -4.15025600
Cl 6.05415000 -0.33058700 0.75117000
Cl -0.20715000 -0.12845400 3.43414600

6⊃HSO_4^-

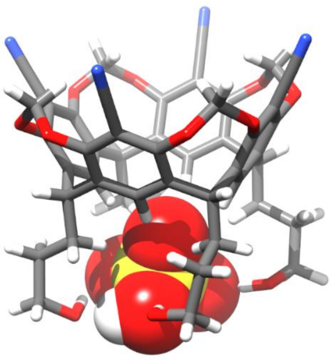

Energy = -3677.3318 Hartree

Number of imaginary frequencies 0

Symbol	X	Y	Z
O	-0.85767400	-3.65483500	-3.57525900
O	1.51752200	-1.25262700	4.82656200
O	-2.14336000	-3.68420900	2.96609700
O	-2.72336700	-4.34427800	0.76397500
O	5.20231200	0.71303000	-0.39995500
O	3.42812800	-0.14535100	3.94770000
O	4.57007400	0.11301900	-2.60404600
O	1.02335800	-2.49522800	-4.44867400
C	2.82476000	-1.38421700	4.28156600
H	2.80403500	-2.04603500	3.40703200
H	3.43246300	-1.79809200	5.08462200
C	-1.90083600	-4.58432200	1.89444600
H	-2.19224400	-5.56586800	2.26453600
H	-0.84219100	-4.55810200	1.60854900
C	5.19704800	-0.29452000	-1.39815400
H	4.72599700	-1.20804100	-1.01449800
H	6.24270800	-0.45931300	-1.65288400
C	0.54727500	-3.65738500	-3.78802800
H	1.07439300	-3.79625700	-2.83611300
H	0.73453800	-4.48108100	-4.47497400
C	-2.42863800	-2.06718800	-0.02136500
C	0.92544000	0.57276700	-2.41789900
H	0.19647700	1.25211600	-1.99091700
C	2.04399700	1.02992500	2.34080100
C	-2.97717400	-1.51133400	1.28978700
H	-3.64570600	-2.27704200	1.69755000
C	3.22330300	0.36110800	2.67867900
C	0.06227700	-0.12497400	3.24897700
C	-1.34073200	-3.09767700	-2.40903000
C	-2.01358100	-1.23090000	-1.06272200
H	-2.09180000	-0.15986100	-0.92616200
C	0.44438000	-1.26838800	3.95961000
C	-1.41902600	-2.50956500	3.00604400
C	3.21032900	-0.08721400	-2.73078000
C	1.92403300	1.54255700	1.04388300
H	1.00677800	2.04517500	0.76613900
C	-2.28302200	-3.44850000	-0.18989600
C	0.46304400	-0.51479400	-3.16552100
C	2.94304000	1.44832700	0.09180400
C	-1.05243800	-0.21499100	2.40324500
H	-1.32407900	0.64957800	1.80792100
C	4.27148100	0.23987800	1.74556000
C	-1.02000000	-0.77932000	-3.37823000
H	-1.10618600	-1.33800200	-4.31634100
C	2.28596000	0.81490500	-2.19353800
C	1.40385400	-1.41434200	-3.67773600
C	2.78030900	-1.20631900	-3.47039300
C	-1.81146000	-1.38527300	2.27042700
C	0.91187400	1.13717100	3.35900400
H	1.37476500	1.12283200	4.35138400
C	-1.73560700	-3.97230700	-1.37743400
C	-1.49091800	-1.71462500	-2.26685700
C	2.76990700	1.97733800	-1.32823400
H	3.76836000	2.24738300	-1.68932900
C	4.12364800	0.79124700	0.45771900
C	-0.29363100	-2.46218800	3.85073200
C	-1.87889600	0.49275800	-3.51519400
H	-1.36281100	1.19913300	-4.17532800
H	-1.97183600	1.00593500	-2.55374500
C	-3.80652100	-0.22813100	1.09256500
H	-4.55278400	-0.43085700	0.31455900
H	-3.18365800	0.57842400	0.70549700

C 1.87343500 3.22601000 -1.42352800
H 2.19180200 3.94676200 -0.66299500
H 0.83836700 2.98040500 -1.17269700
C 0.09990200 2.43226700 3.21632600
H -0.76532700 2.37910900 3.88659800
H -0.32058400 2.49898000 2.21626400
C 0.90638400 3.71114000 3.49968800
H 1.95864000 3.57220000 3.22208600
H 0.89044800 3.94236900 4.57349500
C -3.26834000 0.16584300 -4.08171500
H -3.66119500 -0.74542100 -3.61317800
H -3.18322800 -0.04029300 -5.15835300
C -4.50979600 0.23585400 2.37228900
H -3.76923000 0.59089700 3.10123700
H -5.02649000 -0.61166600 2.84540000
C 1.92006400 3.86801800 -2.81544500
H 1.80925500 3.10061800 -3.59193600
H 2.90042100 4.34186200 -2.97155800
C 0.38513300 4.90995900 2.70160500
H -0.70640300 4.98079800 2.79121700
H 0.82534100 5.83924600 3.09197000
C -4.30207000 1.26944000 -3.85128400
H -3.90775400 2.24281900 -4.18067900
H -5.20119100 1.05967400 -4.44342400
C -5.55260300 1.32892400 2.13189200
H -6.31204400 0.97974200 1.42416400
H -6.06007700 1.57375500 3.07626400
C 0.81374300 4.90383900 -3.03699600
H 0.83050200 5.65455100 -2.23201300
H 0.99206400 5.43073500 -3.98320300
O 0.76492900 4.75946500 1.33168000
H -0.01145700 4.83218400 0.75110300
O -4.71689600 1.32393600 -2.48884700
H -4.14879100 1.93666000 -1.99277900
O -5.02221300 2.52942200 1.55859100
H -4.20807700 2.81012500 2.02118000
O -0.47134900 4.29815200 -3.13344600
H -0.88616600 4.30330300 -2.25136500
C 0.11380600 -3.62939200 4.57002300
N 0.45815100 -4.58781800 5.13352600
C 5.46906900 -0.45907600 2.09534100
N 6.43187000 -1.05248900 2.37077700
C -1.56115400 -5.38412800 -1.52523100
N -1.39431900 -6.53102000 -1.63271800
C 3.73502200 -2.13883400 -3.98453800
N 4.51037000 -2.91247100 -4.37846700
S -2.11051400 3.20479600 0.10957200
O -1.39302500 1.94287000 -0.21115500
O -1.46823700 4.40462400 -0.49166600
O -2.42547000 3.35912200 1.55676900
O -3.57657400 3.10190500 -0.63958000
H -4.25202800 2.84007700 0.04716600

6⊃ClO_4^-

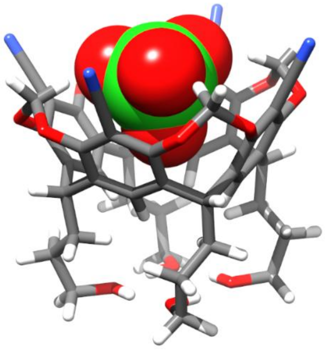

Energy = -3738.3974 Hartree

Number of imaginary frequencies 0

Symbol	X	Y	Z
O	3.84675600	2.67066900	1.76361700
O	-2.63288800	-3.75054200	1.88399100
O	2.11260900	-3.74788400	2.54893900
O	3.79086700	-2.06302700	2.52475100
O	-4.25777200	2.56050500	0.56953900
O	-4.31492300	-2.17028100	1.31779900
O	-2.54816900	4.21104700	0.61189600
O	2.19937500	4.30807400	1.25294100
C	-3.44022800	-2.66311200	2.33512000
H	-2.81513300	-1.85145900	2.72100000
H	-4.09230200	-3.08622900	3.09718700
C	2.72116400	-2.66608200	3.25456600
H	3.19018500	-3.12052900	4.12517400
H	1.97284700	-1.91591700	3.51886900
C	-3.36796300	3.33036600	1.38078100
H	-2.75181700	2.66867900	1.99808600
H	-4.00775700	3.97966800	1.97580000
C	2.79042000	3.49191400	2.26409300
H	2.02979000	2.87661200	2.74961600
H	3.27069000	4.18831700	2.94881900
C	2.96801700	-1.20972500	0.41976600
C	0.14325900	2.44975800	-1.14251800
H	0.24874600	1.71339900	-1.92549000
C	-2.93889400	-1.28561000	-0.45886900
C	2.61968800	-2.61472600	-0.07089400
H	3.32089000	-3.31204900	0.39995500
C	-3.85508900	-1.10183600	0.57812000
C	-1.20579600	-2.96396600	0.09603100
C	3.52027600	1.41114000	1.31071500
C	2.70465400	-0.08508400	-0.36043000
H	2.23918700	-0.21844600	-1.32565400
C	-1.37883200	-3.46026800	1.39138800
C	1.03924300	-3.46014000	1.73344300
C	-1.30259000	3.75813600	0.23374000
C	-2.46807700	-0.15342500	-1.11572300
H	-1.70874400	-0.29820800	-1.87037500
C	3.48908600	-1.00173300	1.69948900
C	1.28798400	2.91264900	-0.49565600
C	-2.90903900	1.14048300	-0.84345400
C	0.09648600	-2.69001400	-0.31925700
H	0.25017100	-2.24056800	-1.28895700
C	-4.31945500	0.18868800	0.89815200
C	2.67635300	2.39895600	-0.87583000
H	3.39619800	3.19147600	-0.64475600
C	-1.14577200	2.87673600	-0.83893100
C	1.11557300	3.80299300	0.56695900
C	-0.17509200	4.23210700	0.93428900
C	1.22772300	-2.95510600	0.44554000
C	-2.40504800	-2.66906700	-0.80493300
H	-3.18964900	-3.39075800	-0.55316500
C	3.76690400	0.30517100	2.15103400
C	2.99896700	1.21740500	0.02958100
C	-2.35047400	2.34297800	-1.60278900
H	-3.11573900	3.12643800	-1.58621200
C	-3.82414000	1.30569500	0.19860900
C	-0.26152700	-3.71752400	2.21106600
C	2.72806000	2.08258000	-2.38884300
H	2.54721300	3.02229000	-2.92704900
H	1.88812200	1.43635100	-2.64329700
C	2.77234900	-2.69046900	-1.60803800
H	3.82648400	-2.49352500	-1.84367600
H	2.21574900	-1.86902000	-2.05878200

C -2.05824000 1.94161500 -3.06868800
H -3.01396400 1.65110100 -3.52430000
H -1.44991100 1.03746600 -3.07025700
C -2.00513000 -2.84220400 -2.28976500
H -1.71836500 -3.89218700 -2.43377400
H -1.10074100 -2.26501800 -2.48035400
C -2.99745000 -2.42393800 -3.37749500
H -3.41542600 -1.43687000 -3.15664700
H -3.84500500 -3.11981100 -3.44126500
C 3.97105100 1.39422100 -2.95857700
H 4.26257200 0.54586500 -2.33161000
H 4.83177000 2.07611400 -2.98828700
C 2.30963800 -3.95400900 -2.33745500
H 1.31552700 -4.25501500 -1.99260300
H 2.98171900 -4.80107400 -2.14394600
C -1.33348600 2.93283600 -3.98308500
H -0.46932800 3.36852600 -3.47228800
H -1.98582300 3.76876700 -4.26995800
C -2.30269200 -2.36679000 -4.74857000
H -1.99111700 -3.37383100 -5.06021000
H -2.99495300 -1.98461600 -5.50656300
C 3.69948500 0.88167000 -4.38297900
H 3.53761400 1.72669200 -5.06722200
H 4.56248800 0.31701700 -4.75198600
C 2.25050200 -3.70915800 -3.85479000
H 3.26106000 -3.53897600 -4.25248300
H 1.83769400 -4.58719900 -4.36313200
C -0.84531200 2.22871200 -5.26057500
H -1.70214500 1.89885300 -5.86498100
H -0.25555400 2.92081800 -5.87126100
O -1.15630100 -1.49707800 -4.75927400
H -0.34600800 -2.01148800 -4.56896100
O 2.56661800 -0.00239900 -4.45319500
H 1.75829200 0.50790200 -4.66271600
O 1.41288400 -2.59444800 -4.20994400
H 1.95012900 -1.77915100 -4.27364800
O -0.00050400 1.09630900 -4.98704000
H -0.53677000 0.27862900 -4.95174300
C -0.44822900 -4.21751400 3.53746400
N -0.59726800 -4.63313100 4.61408900
C -5.23533200 0.37110800 1.98044600
N -5.98986600 0.51823200 2.85413800
C 4.29640700 0.50985200 3.46357000
N 4.73134100 0.67637000 4.53000800
C -0.34255300 5.12686000 2.03673400
N -0.47599200 5.86265000 2.92830800
Cl -0.39750900 0.38758800 2.38460600
O 0.16937500 -0.71101400 3.23586700
O 0.16721300 1.71176300 2.81015200
O -1.89317700 0.41604900 2.54707900
O -0.05819300 0.13529900 0.94709200
